# CAR T cells with dual targeting of CD19 and CD22 in adult patients with recurrent or refractory B cell malignancies: a phase 1 trial

**DOI:** 10.1038/s41591-021-01436-0

**Published:** 2021-07-26

**Authors:** Jay Y. Spiegel, Shabnum Patel, Lori Muffly, Nasheed M. Hossain, Jean Oak, John H. Baird, Matthew J. Frank, Parveen Shiraz, Bita Sahaf, Juliana Craig, Maria Iglesias, Sheren Younes, Yasodha Natkunam, Michael G. Ozawa, Eric Yang, John Tamaresis, Harshini Chinnasamy, Zach Ehlinger, Warren Reynolds, Rachel Lynn, Maria Caterina Rotiroti, Nikolaos Gkitsas, Sally Arai, Laura Johnston, Robert Lowsky, Robbie G. Majzner, Everett Meyer, Robert S. Negrin, Andrew R. Rezvani, Surbhi Sidana, Judith Shizuru, Wen-Kai Weng, Chelsea Mullins, Allison Jacob, Ilan Kirsch, Magali Bazzano, Jing Zhou, Sean Mackay, Scott J. Bornheimer, Liora Schultz, Sneha Ramakrishna, Kara L. Davis, Katherine A. Kong, Nirali N. Shah, Haiying Qin, Terry Fry, Steven Feldman, Crystal L. Mackall, David B. Miklos

**Affiliations:** 1 Division of Blood and Marrow Transplantation and Cellular Therapy, Stanford University School of Medicine, Stanford, CA USA; 2 Center for Cancer Cell Therapy, Stanford Cancer Institute, Stanford University School of Medicine, Stanford, CA USA; 3 Division of Hematology/Oncology, Loyola University Medical Center, Chicago, IL USA; 4 Department of Clinical Pathology, Stanford University School of Medicine, Stanford, CA USA; 5 Department of Biomedical Data Science, Stanford University School of Medicine, Stanford, CA USA; 6 Department of Pediatrics–Hematology/Oncology, Stanford University School of Medicine, Stanford, CA USA; 7 Adaptive Biotechnologies, Seattle, WA USA; 8 IsoPlexis, Brantford, CT USA; 9 BD Biosciences, San Jose, CA USA; 10 Pediatric Oncology Branch Center for Cancer Research, National Institutes of Health, Bethesda, MD USA; 11 Department of Pediatrics–Hematology/Oncology, University of Colorado Anschutz and Children’s Hospital Colorado, Denver, CO USA; 12 Present Address: Lyell Immunopharma, San Francisco, CA USA

**Keywords:** Cancer immunotherapy, Acute lymphocytic leukaemia, Lymphoma, Cancer immunotherapy, Phase I trials

## Abstract

Despite impressive progress, more than 50% of patients treated with CD19-targeting chimeric antigen receptor T cells (CAR19) experience progressive disease. Ten of 16 patients with large B cell lymphoma (LBCL) with progressive disease after CAR19 treatment had absent or low CD19. Lower surface CD19 density pretreatment was associated with progressive disease. To prevent relapse with CD19^−^ or CD19^lo^ disease, we tested a bispecific CAR targeting CD19 and/or CD22 (CD19-22.BB.z-CAR) in a phase I clinical trial (NCT03233854) of adults with relapsed/refractory B cell acute lymphoblastic leukemia (B-ALL) and LBCL. The primary end points were manufacturing feasibility and safety with a secondary efficacy end point. Primary end points were met; 97% of products met protocol-specified dose and no dose-limiting toxicities occurred during dose escalation. In B-ALL (*n* = 17), 100% of patients responded with 88% minimal residual disease-negative complete remission (CR); in LBCL (*n* = 21), 62% of patients responded with 29% CR. Relapses were CD19^−/lo^ in 50% (5 out of 10) of patients with B-ALL and 29% (4 out of 14) of patients with LBCL but were not associated with CD22^−/lo^ disease. CD19/22-CAR products demonstrated reduced cytokine production when stimulated with CD22 versus CD19. Our results further implicate antigen loss as a major cause of CAR T cell resistance, highlight the challenge of engineering multi-specific CAR T cells with equivalent potency across targets and identify cytokine production as an important quality indicator for CAR T cell potency.

## Abstract (web summary)

Bispecific CAR T cells targeting CD19 and CD22 exhibit clinical activity and low toxicity in patients with large B cell lymphoma and B cell acute lymphoblastic leukemia, with relapses associated with loss of CD19 but not CD22.

## Main

Impressive antitumor effects of chimeric antigen receptor-modified T cells^1–10^ and natural killer (NK) cells^11^ targeting CD19 (CAR19) have driven a paradigm shift in the treatment of relapsed or chemotherapy-refractory (relapsed/refractory) B cell malignancies. However, most patients treated with CAR19 experience disease progression. Clinical factors such as preCAR disease burden and serum lactate dehydrogenase (LDH)^5,8^ have been linked to response to CAR19 therapies. Disease progression associated with loss of cell surface CD19 has been reported in 30–95% of relapses after CAR19 therapy in B-ALL^12^, through a variety of mechanisms including splice mutations and retained intracellular CD19^12–16^. Several reports have also demonstrated that effective CAR T cell responses require high target antigen expression density^17–21^; resistance associated with diminished antigen density has been shown after treatment with a monospecific CD22-CAR^22^ and B cell maturation antigen CARs^23^. While CD19 is expressed at variable levels in LBCL^21^, the role of emergence of CD19^−/lo^ LBCL in CAR19 resistance has not been well studied^24,25^. Engineering next-generation therapeutics to overcome newly defined mechanisms of resistance is an important unmet goal.

CD22 is a sialic acid-binding adhesion molecule largely restricted to the B cell lineage and expressed on most B-lineage malignancies^26–30^. In a study of children and young adults with B-ALL enriched for patients with progressive disease after CD19-directed therapy, CD22 CAR T cells induced a 73% CR rate with equal effectiveness in CD19^+^ and CD19^−^ B-ALL^22,31^. However, relapse was associated with the emergence of CD22^lo^ B-ALL, illustrating the limitation of sequential targeting in B-ALL. In a phase I trial of adults with relapsed/refractory LBCL and B-ALL, we tested a bispecific CAR T targeting CD19 and/or CD22 (CD19-22.BB.z)^22^ and demonstrated the feasibility of manufacturing in a closed system, safety of bispecific CAR T cells and clinical activity in both B-ALL and LBCL. The observed relapses with CD19^−/lo^ and maintained CD22 expression could be due to reduced potency of the bispecific receptor toward CD22 versus CD19.

## Results

### Axicabtagene ciloleucel resistance is associated with CD19^−^ and CD19^lo^ LBCL

**Fig. 1:**
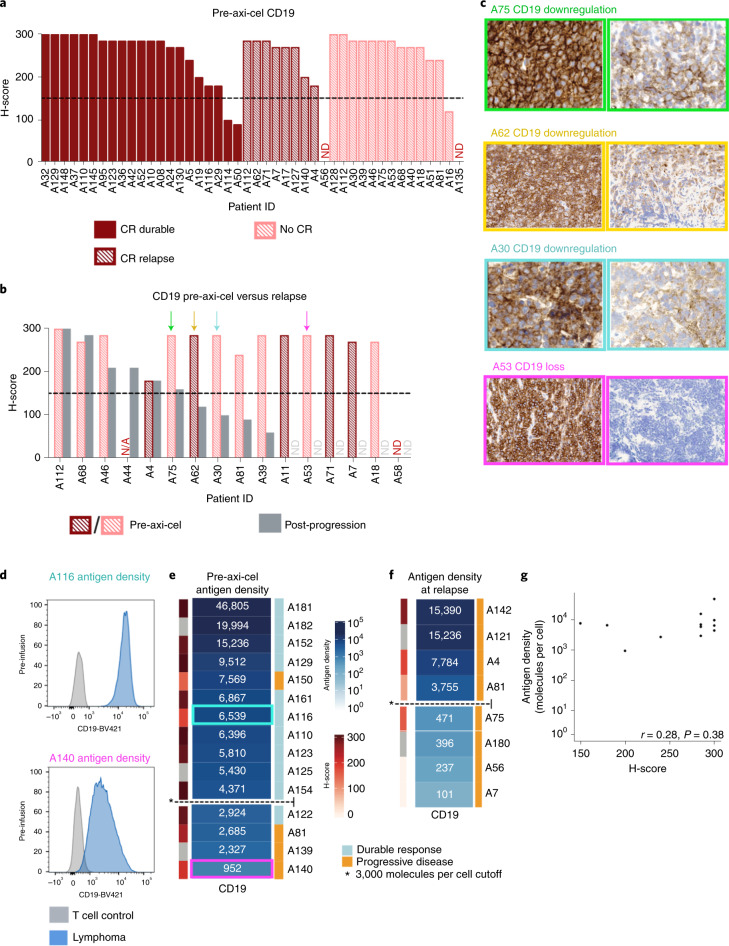
IHC demonstrates CD19^−/lo^ disease postaxi-cel and quantitative flow cytometry of LBCL preaxi-cel therapy is associated with disease progression. **a**, Preaxi-cel H-scores did not distinguish long-term responders and those with progression postaxi-cel (*P* = 0.32 by *t*-test, *P* = 1 Fisher’s exact test). Waterfall plot of CD19 IHC H-scores preaxi-cel therapy (*n* = 44 patients). The H-score was calculated by the percentage of positive tumor cells (0–100) × stain intensity (1–3). The dashed line denotes the H-score of 150, which was used to define antigen positivity. ND, not detectable. **b**, Paired CD19 H-scores preaxi-cel and at progression show significant differences (*P* = 0.003 by Wilcoxon signed-rank test). Using an H-score cutoff of 150, and the observed rate of CD19^−/lo^ progression (10 out of 16 patients), the estimated 95% binomial CI (Wilson score) for CD19^−/lo^ progression was 38–82%. N/A, no data point. **c**, Representative patients with IHC demonstrating decreased CD19 expression at the time of progression (A75, relapse H-score = 160; A62 relapse H-score = 120; A30, relapse H-score = 100; A53, relapse H-score = 0) **d**, Preaxi-cel site density by quantitative flow cytometry in a patient with ongoing response (A116) compared with a patient who experienced progression (A140) **e**, Preaxi-cel median CD19 site density by quantitative flow cytometry organized from highest (dark blue) to lowest (white) in 15 patients. Patients with lower site density were more likely to experience disease progression after axi-cel (*P* = 0.03 by Firth logistic regression). Based on the fit model, 3,000 molecules per cell was defined as the cutoff for CD19 positivity. **f**, Median site density at the time of axi-cel progression (*n* = 8). Four patients had a site density <3,000 molecules per cell. **g**, The preaxi-cel H-score did not correlate with antigen site density by quantitative flow cytometry (*n* = 12) (Spearman *r* = 0.28, *P* = 0.38). Source data

To assess whether resistance to CAR19 therapy in LBCL is associated with CD19^−/lo^ relapse, we used the semiquantitative immunohistochemistry (IHC) H-score to measure CD19 expression at baseline and at disease progression in patients treated with axicabtagene ciloleucel (axi-cel) at our institution. Our cohort comprised 44 consecutive patients treated with axi-cel with available pretreatment tissue biopsies for IHC. With a median follow-up of 21 months (95% confidence interval (CI) 11–24), median progression-free survival (PFS) was 6.1 months (95% CI 3.1–not estimable); 23 patients (52%) experienced progression. Sixteen of 23 patients with progression had postprogression biopsies available. Before axi-cel, the median CD19 H-score was 285 (interquartile range (IQR) = 240–285) (Fig. 1a and Supplementary Fig. 1). Using an H-score cutoff of 150 to indicate CD19 positivity, 39 (89%) patients were CD19^+^ pretherapy. CD19 positivity was not associated with durable response (Fisher’s exact test *P* = 1). In contrast, only 6 of 16 samples studied (37.5%) were CD19^+^ at disease progression. Among patients with paired pre- and posttherapy H-scores, 9 of 15 (60%) converted from CD19^+^ pretherapy to CD19^−^ at relapse (McNemar test *P* = 0.003) (Fig. 1b). Some postprogression biopsies showed complete loss of CD19, while others demonstrated diminished CD19 expression (Fig. 1c). Additionally, treating the pretreatment H-score as a continuous variable also demonstrated no difference between patients with durable disease control versus those who experienced disease progression (*t*-test *P* = 0.32). These data demonstrate that progression after axi-cel therapy for LBCL is associated with emergent CD19^−/lo^ disease in a high percentage of patients, but pretreatment semiquantitative IHC measurement of CD19 expression does not identify patients at risk of relapse.

**Extended Data Fig. 1:**
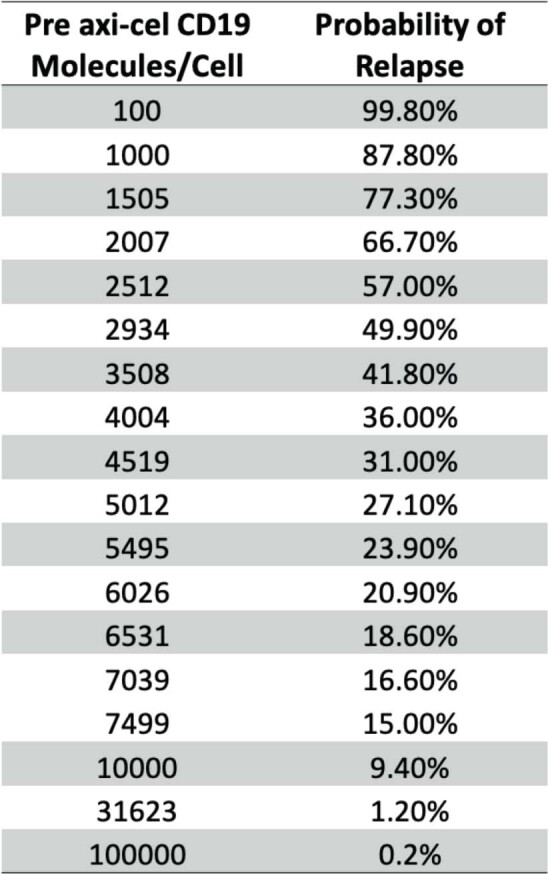
Model-based prediction of relapse after axi-cel. Estimated risk of relapse for a given pre-treatment CD19 antigen density based on a fitted penalized logistic regression model.

**Extended Data Fig. 2:**
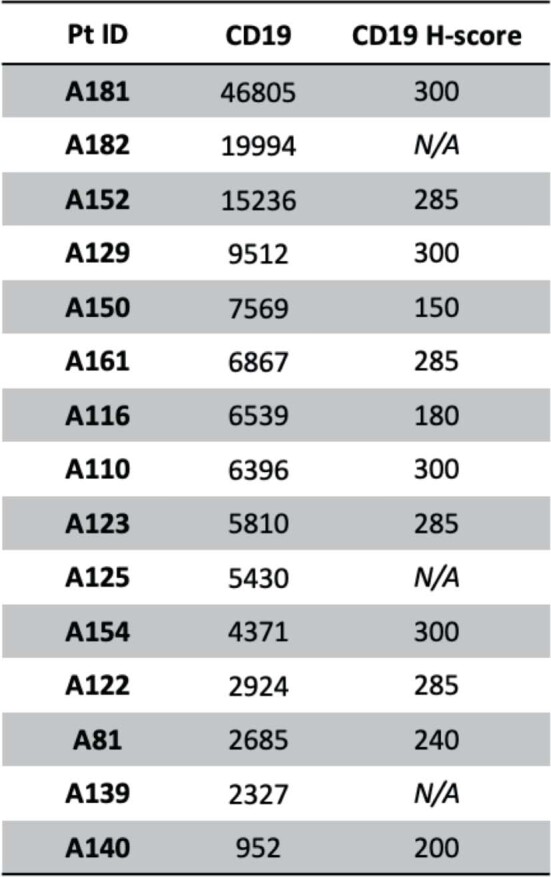
Evaluation of pre-axi-cel antigen density. CD19 IHC H-score and antigen density by quantitative flow cytometry in 15 patients prior to axi-cel. Spearman correlation for these values shown in 1G.

To evaluate whether quantitative assessment of CD19 cell surface density might predict outcomes after axi-cel and attempt to define a threshold level of antigen expression associated with relapse, we used quantitative flow cytometry to measure CD19 site density on viable single-cell suspensions obtained by fine needle aspiration. Preaxi-cel LBCL demonstrated substantial intra- and interpatient variability in median CD19 site density as illustrated in Fig. 1d, which shows LBCLs from two representative patients, both of whom were CD19^+^ by IHC H-score. Patient A116 had a median CD19 site density of 6,538 molecules per cell and experienced durable disease control; in contrast, patient A140 had a median CD19 site density of 952 molecules per cell and experienced disease progression at 3 months after initial CR. Median preaxi-cel CD19 site density (*n* = 15) using quantitative flow cytometry was 6,396 molecules per cell (IQR = 3647–8540) with a range of 952–46,805 molecules per cell (Fig. 1e). Using a penalized logistic regression model, patients with lower pretherapy median CD19 site density had significantly increased risk of progression after axi-cel (*P* = 0.03), with a 50% risk of progression for patients with 2,934 CD19 molecules per cell. Therefore, we selected 3,000 molecules per cell as a cutoff to define CD19 positivity (Extended Data Fig. 1). In our cohort, 3 of 4 patients with LBCL expressing ≤3,000 CD19 molecules per cell experienced progression while 1 of 11 patients with LBCL expressing >3,000 molecules per cell had disease progression. Of 8 patients studied after axi-cel progression, 4 demonstrated a CD19 site density ≤3,000 molecules per cell (Fig. 1f and Extended Data Fig. 2). The H-score did not correlate well with antigen site density (Spearman *r* = 0.28, *P* = 0.38; Fig. 1g and Extended Data Fig. 2). These data corroborate the emergence of CD19^−/lo^ LBCL as a major cause of resistance to CAR19 therapy, suggesting that the risk of relapse increases in LBCLs expressing a median of ≤3,000 molecules per cell before therapy, raising the prospect that quantitative flow cytometry, but not IHC, may identify LBCL patients a priori at risk of CD19^−/lo^ relapse after CAR19.

### CAR construct and clinical trial design

**Fig. 2:**
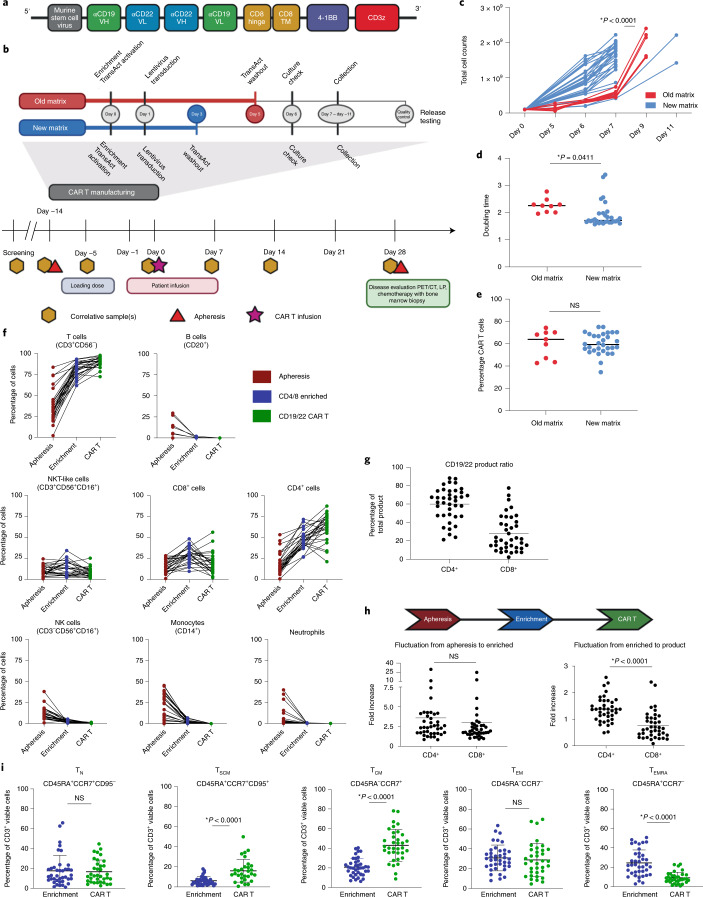
Characterization of CAR products throughout the manufacturing process reveals compositional and phenotypic changes. **a**, CD19-22-CD8.BB.z-CAR contained the CD19 FMC63 and CD22 M971 scFvs, CD8α hinge and transmembrane domains, a 4-1BB costimulatory domain and a CD3ζ domain. The unique bispecific structure shows FMC63 heavy chain proximal, followed by M971 light chain, M971 heavy chain and FMC63 light chain distal. **b**, CAR T manufacturing and clinical trial schema. The manufacturing schema shows the TransAct process change from old to new matrix. The clinical trial schema shows the screening, lymphodepletion, CAR T cell infusion and disease evaluation time points. LP, lumbar puncture. **c**, Improved culture expansion resulting from the new matrix manufacturing process compared to the old matrix (*P* < 0.0001, two-tailed *t*-test). **d**, Significant reduction in doubling time with the new matrix process compared to the old matrix (*P* = 0.0411, two-tailed *t*-test). **e**, No significant difference in transduction efficiency between old and new matrix (*P* = not significant (NS), two-tailed *t*-test). Overall average transduction efficiency was 60.1% (*n* = 39 individual products). **f**, Composition of apheresis, CD4/8-enriched and CD19-22.BB.z product over time, looking at T cell (CD3^+^CD56^−^), B cell or leukemic cell (CD20^+^), CD4^+^, CD8^+^, NKT-like (CD3^+^CD56^+^CD16^+^), NK, monocyte and neutrophil subsets. **g**, Phenotyping of CAR T cell product reveals a skewing toward CD4^+^ cells (*n* = 39 individual products). **h**, Comparing the fold increase from apheresis to enrichment to final product reveals the skewing toward CD4^+^ cells that occurred during culture between enrichment and final product (*P* < 0.0001, two-tailed *t*-test). **i**, Phenotyping of T cell memory subsets revealed an enrichment in T_SCM_ (*P* < 0.0001, two-tailed *t*-test) and T_CM_ (*P* < 0.0001, two-tailed *t*-test) cell subsets and a depletion of the T_EMRA_ (*P* < 0.0001, two-tailed *t*-test) subset. There was no significant change in T_N_ or T_EM_ cell subsets between enrichment and CD19-22.BB.z product. Source data

Previous data demonstrated that CD19 loss is an important mechanism of CAR19 failure in B-ALL. The data presented in this study similarly implicate absent or decreased cell surface CD19 as a mechanism of resistance after CAR19 in LBCL, providing a rationale for dual antigen targeting in B-ALL and LBCL. As reported previously^22,32^, we generated a CD19-22.BB.z-CAR comprising a single cistron encoding the anti-CD19 murine FMC63 single-chain variable fragment (scFv) and fully human anti-CD22 m971 scFv (ɑCD19 vH-ɑCD22 vL-linker-ɑCD22 vH-ɑCD19 vL), followed by human CD8 hinge and transmembrane domains, 4-1BB costimulation and CD3ζ activation domains. The CD19-22.BB.z-CAR was encoded by a self-inactivating lentiviral vector under control of an murine stem cell virus internal promoter (Fig. 2a and Supplementary Fig. 2). We conducted a phase I clinical trial of CD19-22.BB.z-CAR in patients with relapsed/refractory B-ALL and LBCL, evaluating the feasibility of manufacture and safety of CD19-22.BB.z-CAR as the primary end points. Eligible patients had disease relapsed or refractory after two or more lines of therapy and had measurable disease that expressed CD19. Patients received conditioning chemotherapy followed by CAR T cell infusion at 2 dose levels: 1 × 10^6^ CAR^+^ cells kg^−1^ (DL1) and 3 × 10^6^ CAR^+^ cells kg^−1^ (DL2) using a 3 + 3 dose escalation phase that enrolled LBCL and B-ALL in one cohort. While a maximal tolerated dose was not identified, a third dose level at 1 × 10^7^ cells kg^−1^ was not pursued due to the efficacy seen at DL2 and concern regarding the possible toxicity of cell doses higher than 3 × 10^6^ cells kg^−1^ seen in other clinical trials. Therefore, the two disease-specific expansion cohorts received the recommended phase 2 dose of 3 × 10^6^ CAR T cells kg^−1^ (DL2).

### Patient characteristics

**Extended Data Fig. 3:**
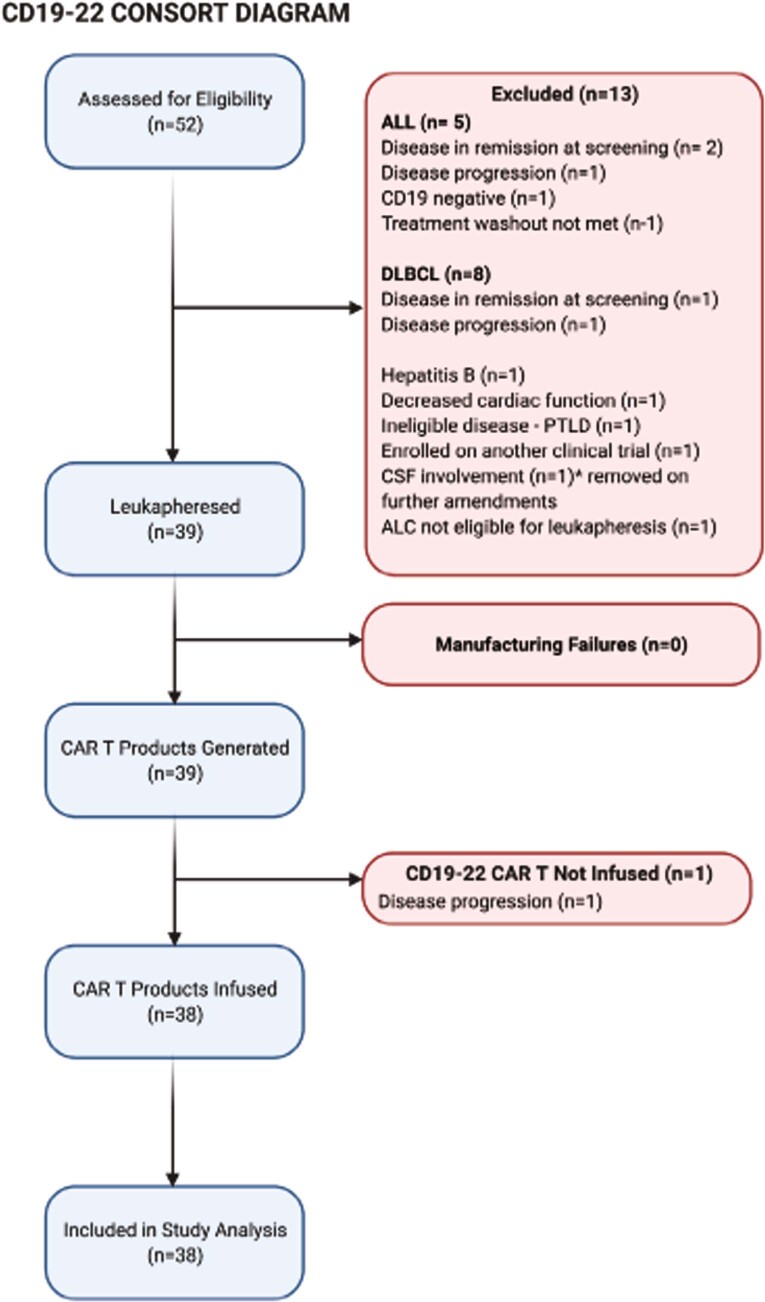
CONSORT Diagram. Consort diagram of CD19-22-CD8.BB.z clinical trial.

Thirty-nine patients were enrolled in two cohorts (B-ALL, *n* = 17, LBCL, *n* = 22); 38 patients received the CD19-22.BB.z-CAR infusion while one patient with LBCL died during lymphodepletion due to progressive disease and sepsis (Extended Data Fig. 3). The median age for the cohort with LBCL who received the infusion was 69 years (range 25–78) (Table 1); 15 had double expressor (expression of c-MYC and BCL2) LBCL, 3 had high-grade B cell lymphoma with translocation of c-MYC and BCL2 and/or BCL6 (double-hit) and 4 had previous autologous stem cell transplant. All patients with LBCL were CAR-naive. In the cohort with B-ALL, median age was 47 years (range 26–68); 71% had progressed after allogeneic hematopoietic cell transplantation (HCT), 65% had previous CD19-directed therapy (including 1 patient receiving previous CAR T) and 29% had previous CD22-directed therapy (Table 2). Sixty-three percent had previous central nervous system (CNS) involvement and 12% had active CNS disease at the time of enrollment.

**Table 1:** Patient characteristics, responses and outcomes for enrolled patients with LBCL

Patient ID	Dose level^a^	Age/sex	Disease	DEL/DHL	Lines of therapy	SPD^b^	Pre-lymphodepletion LDH	Maximum CRS	Maximum neurotoxicity	Best response (PFS)^c^	CD19 H-score at progression	CD19 site density at progression^d^	CD22 H-score at progression	CD22 site density at progression
SL1	1	71/M	DLBCL	DEL + DHL	3	141.08	788	1	2	PD (1)	>90% flow	–	95% flow	–
SL2	1	54/M	TFL	DEL	3	129.9	614	0	0	PR (5)	150	–	0	–
SL3	1	67/M	DLBCL	DEL	4	5.5	254	0	0	PD (1)	210	–	240	–
SL5	1	75/M	DLBCL	–	2	8.35	223	3	0	CR (22+)	–	–	–	–
SL6	1	67/M	DLBCL	–	2	16.12	196	1	1	PR (5)	>95% flow	–	>60% flow	–
SL10	2	70/M	DLBCL	DEL	2	5.6	222	1	0	SD (2)	270	8682	285	38,002
SL11	2	25/F	PMBCL	DEL	2	96.48	335	0	0	PR (5)	180	1150	270	1,046
SL12	2	70/F	DLBCL	DEL	2	36.39	326	1	0	PR (3)	285	–	285	
SL14	2	64/M	TFL	DHL	2	60.68	291	1	0	CR (14+)	–	–	–	–
SL15	2	77/M	DLBCL	DHL	4	93.07	1563	1	1	SD (1)	>90% flow	5446	26% flow	1,942
SL16	2	66/F	DLBCL	–	2	14.35	201	1	0	PD (1)	270	–	0	–
SL17	2	71/M	TFL	N/A	3	61.36	312	N/A	N/A	Not infused	N/A	N/A	N/A	N/A
SL18	2	75/M	DLBCL	DEL	4	47.63	274	2	3	PR (3)	Not biopsied	–	–	–
SL19	2	67/F	DLBCL	DEL	2	4.93	177	1	2	PD (1)	0	–	90	–
SL20	2	69/F	Richter^e^	DEL	7	2.72	130	1	0	PD (1)	30	–	150	–
SL21	2	59/F	DLBCL^e^	DEL	4	0.6	250	1	0	CR (6)	0	110	100	2,888
SL22	1	34/F	PMBCL	DEL	2	29.96	336	0	0	PR (9)	300	–	300	–
SL23	2	70/M	Richter	DEL	3	39.1	220	1	1	SD (3)	300	21092	300	12,492
SL25	2	78/M	DLBCL^e^	DEL	4	5.18	200	2	1	CR (11+)	–	–	–	–
SL27	2	76/M	DLBCL	DEL	3	6.6	246	1	1	PR (3)	Not biopsied	–	–	–
SL28	2	73/M	DLBCL^e^	N/A	4	9.07	200	0	0	CR (10+)	–	–	–	–
SL31	2	60/M	TFL	DEL	4	35.94	197	2	2	CR (10+)	–	–	–	–

^a^DL1 was 1 × 10^6^ cell kg^−1^; DL2 was 3 × 10^6^ cells kg^−1^. ^b^SPD was calculated by summing the products of the cross-sectional diameters of the preinfusion index lesions in cm^2^. ^c^Lymphoma responses were assessed according to Cheson et al.^65^. PFS was determined from the date of infusion until progression or death. The + symbol indicates ongoing response. ^d^CD19 positivity by site density was >3,000 molecules per cell. ^e^Previous autologous stem cell transplant. DEL, double expressor lymphoma; DHL, double-hit lymphoma; DLBCL, diffuse large B cell lymphoma; PMBCL, primary mediastinal B cell lymphoma; SPD, sum product of diameters; TFL, transformed follicular lymphoma.

**Table 2:** Patient characteristics, responses and outcomes for enrolled patients with B-ALL

Patient ID	Dose level	Age/sex	Previous allo-HCT	Previous anti-CD19/CD22	Marrow disease burden	Extra-medullary disease	CNS disease	Maximum CRS	Maximum NE	Best response (PFS)	CD19^+^ blast percentage at progression^a^	CD19 site density at progression^b^	CD22^+^ blast percentage at progression^a^	CD22 site density at progression
SA4	1	50/F	Matched related donor	–	–	Yes	Active	2	0	CRi (7+)	–	–	–	–
SA7	1	35/F	Haplo-identical	–	MRD^c^	–	Previous	1	0	CR (3)	95%^d^	–	95%^d^	–
SA8	2	68/M	MUD	Blinatumomab	–	Yes	Previous	1	2	CRi (12)	–	–	–	–
SA9	2	47/M	MUD	JCAR15^e^	MRD^c^	–	Previous	0	0	CRi (1)	100%		NA	
SA13	2	58/M	–	Blinatumomab/inotuzumab	>5%	Yes	–	4	4	CRi (5)	0%	64	90	6,016
SA24	2	35/M	–	Blinatumomab	>5%	–	–	2	0	CRi (3)	0%	0	90	5,915
SA26	2	26/M	Matched related donor	Blinatumomab/inotuzumab	–	Yes	–	1	0	CR (10+)	–	–	–	–
SA29	2	27/M	MUD	Blinatumomab/inotuzumab	–	Yes	Previous	1	0	PR (3)	N/A^d^	–	–	–
SA30	2	59/M	MUD	Blinatumomab/inotuzumab	MRD^c^	–	–	2	0	CR (9+)	–	–	–	–
SA32	2	36/F	First: matched related donor Second: MUD	–	>5%	–	Previous	2	0	CRi (6)	20%	–	90	–
SA33	2	26/M	–	–	>5%	–	Previous	1	3	CRi (2)	0%	82	90	9,031
SA34	2	48/F	Matched related donor	Blinatumomab	>5%	Yes	–	0	0	CR (6)	100%	17,944	90	1,726
SA35	2	31/M	–	Blinatumomab/inotuzumab	MRD^c^	–	Previous	2	1	CRi (9+)	–	–	–	–
SA36	2	53/F	MMURD	–	–	Yes	Active	0	0	PR (3)	80%	94	90	7,112
SA37	2	33/M	First: matched related donor Second: MUD	Blinatumomab/inotuzumab	–	Yes	Previous	0	0	CRi (2)	>90%	16,074	90	3,815
SA38	2	48/M	MUD	–	>5%	–	–	2	0	CR (6+)	–	–	–	–
SA39	2	48/M	–	Blinatumomab	>5%	–	–	2	3	CR (6+)	–	–	–	–

^a^Antigen status for leukemia determined by standard flow cytometry. ^b^CD19 positivity by site density was >3,000 molecules per cell. ^c^MRD at 10^−4^. ^d^For patients S7 and S29, antigen status at relapse was determined by IHC. Patient S29 was determined CD19^+^ by an outside pathologist. ^e^JCAR15, investigational CAR19. The + symbol indicates an ongoing response. MMURD, mismatched unrelated donor; MUD, matched unrelated donor.

### Feasibility of closed-system manufacturing

**Extended Data Fig. 4:**
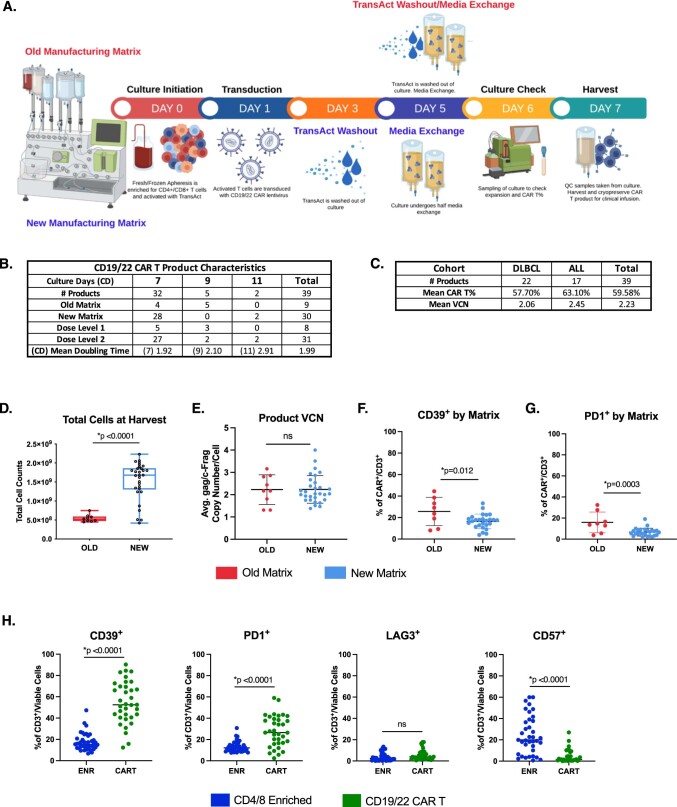
CD19/22 CAR T manufacturing flowchart and product breakdown by culture days, manufacturing matrix, dose level, and disease. **a**, Manufacturing schematic detailing difference in timing of TransAct washout between the Old and New Matrix. **b**, Table of Infusion Product Characteristics, categorized by the number of culture days. **c**, Table of Infusion Product Characteristics, categorized by disease cohort. **d**, Significant difference in product expansion between the old and new matrix at harvest (p < 0.0001, t-test, two tailed). Box plot center line corresponds to the median; hinges correspond to the 25^th^ and 75^th^ percentiles with the whiskers signifying minimum and maximum values **e**, No significant difference in vector copy number based on manufacturing matrix. **f**,**g**, Significant decreases in CD39+ (p = 0.012, t-test, two-tailed) and PD1+ (p = 0.0003, t-test, two-tailed) CAR T cells manufactured using the new matrix versus the old matrix. **h**, Significant increases in CD39+ and PD1+ and a significant decrease in CD57+ expression on CD3+ cells from CD4/8 enriched to final CAR T product (p < 0.0001, t-test, two-tailed; for all 3 tests). No difference in LAG3 expression. Source data

A primary objective of this clinical trial was to assess the feasibility of cell production using closed-system manufacturing in the CliniMACS Prodigy (Miltenyi Biotec), defined as >80% of cell products meeting the protocol-specified cell dose. The manufacturing schema is illustrated in Fig. 2b and Extended Data Fig. 4a. Initially, a 7–9 d manufacturing process (old matrix) utilized washout of the T cell activator (TransAct) on day 5. At DL1, 57% (4 out of 7) of products met the prescribed cell dose within 7 d and 43% (3 out of 7) of products within 9 d. To reduce manufacturing time while increasing dose level, we incorporated a process change to remove the TransAct on day 3 (new matrix), significantly shortening the culture time required to meet the dose, which is reflected by the increased total cell count by day 7 (*P* < 0.0001; Fig. 2c) and decreased product doubling time (*P* = 0.04; Fig. 2d). Among 39 patients who underwent apheresis, CAR T products meeting predefined release criteria were successfully manufactured in 100%. Ninety-seven percent of products met the protocol-specified dose; 82% (32 out of 39) met the protocol-specified dose in 7 d (4 old matrix, 28 new matrix) (Extended Data Fig. 4b–d). Mean transduction efficiency was 60.1% (range 34.6–75.2%; Fig. 2e), with an average vector copy number of 2.23 (range 1.31–4.0; Extended Data Fig. 4e).

### Characterization of CD19-22.BB.z-CAR T infusion products

Samples were obtained from apheresis, postCD4/CD8 T cell enrichment and final CAR T product collection for cell subset analysis and to confirm enrichment in T cell populations and removal of leukemic cells. Compared to apheresis products, manufactured products demonstrated increased T cell and CD4^+^ populations, no change in CD8^+^ and natural killer T (NKT)-like subsets and depletion of B or leukemic cells (defined by CD20^+^), NK cells, monocytes and neutrophils (Fig. 2f).

CD19-22.BB.z products showed a CD4^+^ predominance (Fig. 2g). To determine whether this was due to T cell enrichment postapheresis or the manufacturing process, we assessed the fold increase in CD4^+^ and CD8^+^ populations over the manufacturing process (Fig. 2h). While there was no difference in fold increase from apheresis to enrichment, there was a significant difference in fold increase between CD4^+^ and CD8^+^ T cell subsets from enrichment to final product (*P* < 0.0001), implicating the manufacturing process in enriching the proportion of CD4^+^ cells in the manufactured product. Compared to enriched apheresis samples, products demonstrated enrichment in stem cell memory T (T_SCM_) (*P* < 0.0001) and central memory T (T_CM_) (*P* < 0.0001) cell subsets, no change in naive T (T_N_) and effector memory T (T_EM_) cell populations and a decrease in terminally differentiated effector memory (T_EMRA_) cell subsets (*P* < 0.0001; Fig. 2i).

### Toxicity

**Extended Data Fig. 5:**
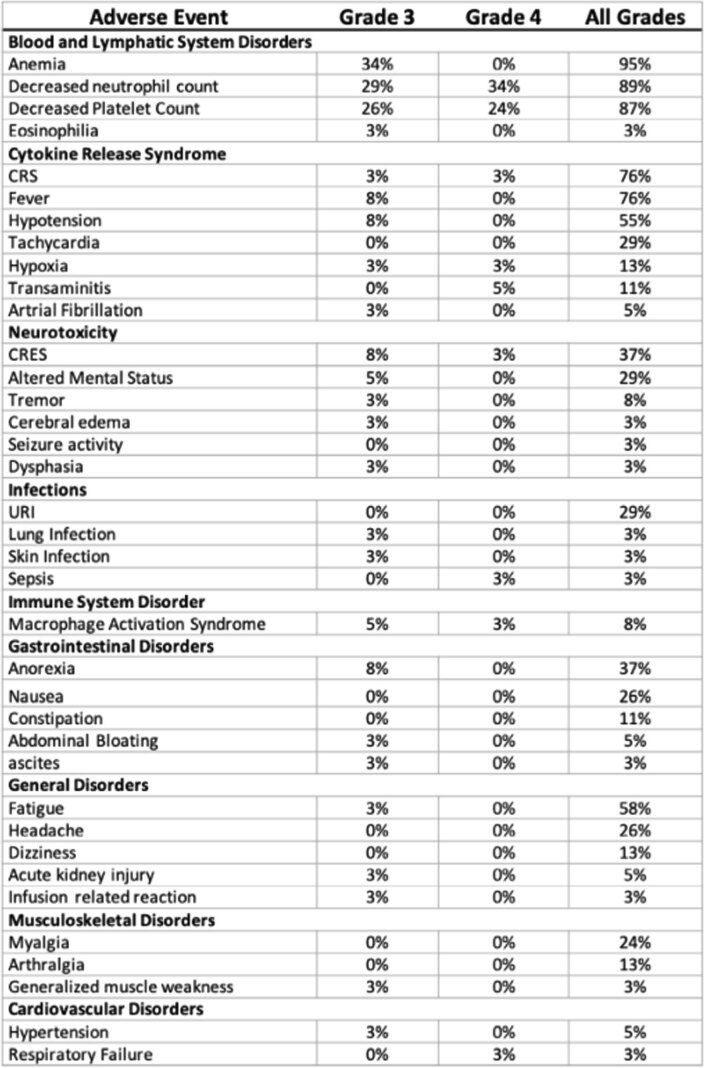
Adverse events summary. Adverse events with a Grade≥3 incidence rate of 5% or higher were included. CAR specific toxicities were included regardless of incidence.

No dose-limiting toxicity (DLT) occurred during dose escalation and one DLT total occurred during the clinical trial. Cytokine release syndrome (CRS) of any grade occurred in 29 patients (76%) with median onset 1 d after infusion (range 0–8) and lasted a median of 4 d (range 1–12) (Tables 1 and 2 and Extended Data Fig. 5). Grade ≥3 CRS occurred in 2 patients (5%). Neurological toxicity occurred in 14 patients (37%; 9 with LBCL and 5 with B-ALL); 4 experienced ≥grade 3 neurotoxicity. Onset of neurotoxicity occurred a median of 5 d after infusion (range 3–9) and lasted a median of 4 d (range 1–11). Both CRS and neurotoxicity were treated according to institutional guidelines, with 15 patients (39%) receiving ≥1 dose of tocilizumab (range 1–3) and 45% of patients receiving corticosteroids. All episodes of CRS and neurotoxicity resolved. Two patients had laboratory evidence of macrophage activation syndrome with hyperferritinemia and hypofibrinogenemia concurrent with grade ≥3 neurotoxicity and received high-dose corticosteroids and anakinra^31^.

### Response

**Fig. 3:**
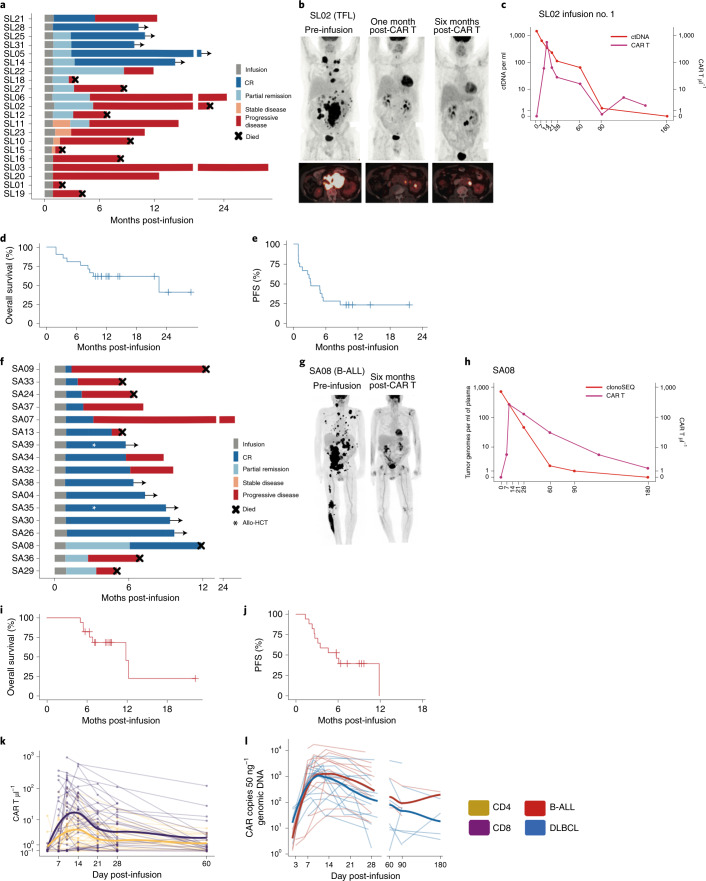
CD19-22.BB.z-CAR is active in both LBCL and B-ALL. **a**, Swimmer plot showing the duration of remission and ongoing responses in patients with lymphoma (*n* = 21). Five patients had an increasing depth of response from 1 to 3 months postinfusion **b**, PET scans for patient S2 showing partial remission at 1 month postinfusion with subsequent progression 6 months after infusion. **c**, Lymphoma disease monitoring using ctDNA. After infusion of CD19-22.BB.z for patient SL02, disease burden continued to decrease; this was coincident with prolonged persistence of CD19-22.BBZ**. d**, Overall survival for 21 infused patients with LBCL. **e**, PFS for the cohort with lymphoma. **f**, Swimmer plot for the cohort with B-ALL (*n* = 17). Two patients received a consolidative allogeneic stem cell transplant (white star) **g**, PET scans for patient SA8, with large bulk disease preinfusion that improved to a CR 6 months postinfusion. **h**, Disease monitoring of patient SA8 using cellular-based NGS with sensitivity of 10^−6^ demonstrates increasing disease control over time coinciding with improving PET response and ongoing persistence of CD19-22.BB.z. **i**, Overall survival of 17 infused patients with B-ALL. **j**, PFS for the cohort with B-ALL. **k**, Absolute number of circulating CD4 and CD8 CD19-22.BB.z CAR T cells after infusion as measured by flow cytometry (*n* = 38 autologous infused products). **l**, Number of circulating CD19-22.BB.z copies per 50 ng of genomic DNA as measured by qPCR (*n* = 33 autologous infused products) showing initial expansion and persistence of CD19-22.BB.z as measured at 1 and 2 months (days 35–75 postinfusion), 3 months (days 76–120) and 6 months after infusion (days 120–200). Source data

**Extended Data Fig. 6:**
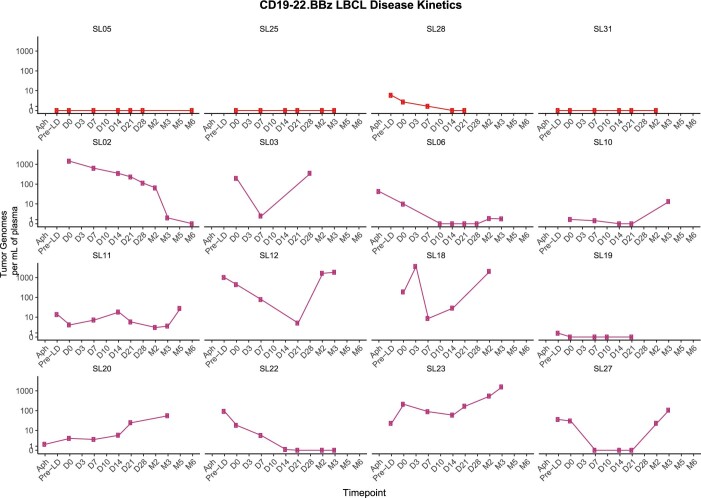
LBCL disease monitoring by ctDNA. Patients with baseline tumor sample were assessed for a dominant clone to allow for disease tracking by cell free tumor DNA in the peripheral blood. Measurements were performed at pre-specified timepoints from pre-infusion to the time of disease progression. Source data

The primary response assessment for LBCL was undertaken at three months after infusion. For patients with LBCL treated at the recommended phase II dose (*n* = 15), the overall response rate (ORR) and CR rate at 3 months were 40% (95% CI 16–68%) and 33% (95% CI 12–62%), respectively. For the entire patient cohort with LBCL (*n* = 21), the best ORR at any time point was 62% (95% CI 38–82%) and the CR rate was 29% (95% CI 11–52%). Five of 13 responders had improvement in response from month 1 to month 3 after infusion (Fig. 3a–c). With a median follow-up of 10 months (95% CI 8.7–21.5), median overall survival was 22.5 months (95% CI 8.3–not estimable; Fig. 3d), which could change with a longer follow-up. Median PFS was 3.2 months (95% CI 1.2–5.5; Fig. 3e). To understand the kinetics of response in patients with LBCL treated with CD19-22.BB.z, we assessed change in lymphoma burden over time by cell-free circulating tumor DNA (ctDNA) in 16 patients with available diagnostic tumor samples (Extended Data Fig. 6)^33^. Four patients with ongoing clinical response had no detectable ctDNA at the time of the last assessment. Among 12 patients with disease progression, we observed an initial reduction in ctDNA that nadired 14–21 d postinfusion, with 9 patients demonstrating a rise in ctDNA at or before clinical progression. These findings suggest that progressive disease after CD19-22.BB.z-CAR in LBCL is associated with a robust early response followed by early acquired resistance.

**Extended Data Fig. 7:**
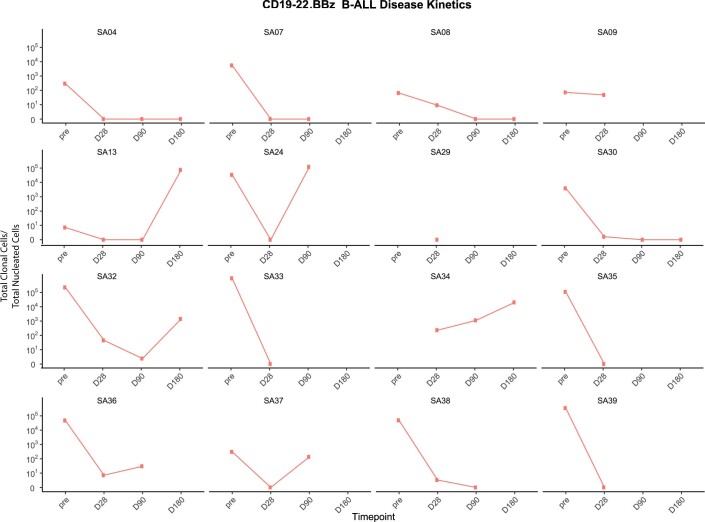
B-ALL disease monitoring by ClonoSeq. Patients with baseline tumor sample were assessed for a dominant clone to allow for disease tracking by cell free tumor DNA in the peripheral blood. Measurements were performed at pre-specified timepoints from pre-infusion to the time of disease progression. Source data

Primary response for B-ALL was evaluated at 28 days postinfusion. All (*n* = 17) patients with B-ALL achieved response; 14 with CR (82%) and 3 with partial remission (Fig. 3f). One patient’s response (SA8) improved to CR 6 months after infusion (Fig. 3g,h), leading to an overall CR rate of 88%, all of whom were negative for minimal residual disease (MRD) at 10^−4^ bone marrow sensitivity (Extended Data Fig. 7) or by positron emission tomography (PET)/computed tomography (CT) for patients with extramedullary disease. After a median follow-up of 9.3 months (95% CI 7.2–NE), median overall survival was 11.8 months (95% CI 5.5–NE) (Fig. 3i) and PFS was 5.8 months (95% CI 2.6–NE) (Fig. 3j). Two patients proceeded to allogeneic stem cell transplant in CR and are in ongoing remission. Serial MRD analyses by next-generation sequencing (NGS) of immunoglobulin receptors from bone marrow revealed that 100% (5 out of 5) of patients with ongoing CR achieved persistent MRD-negative response, while 70% (7 out of 10) of progressors had persistence or rise in MRD before or at the time of morphological relapse (Extended Data Fig. 7).

### In vivo quantification of CD19-22.BB.z CAR T cells

**Extended Data Fig. 8:**
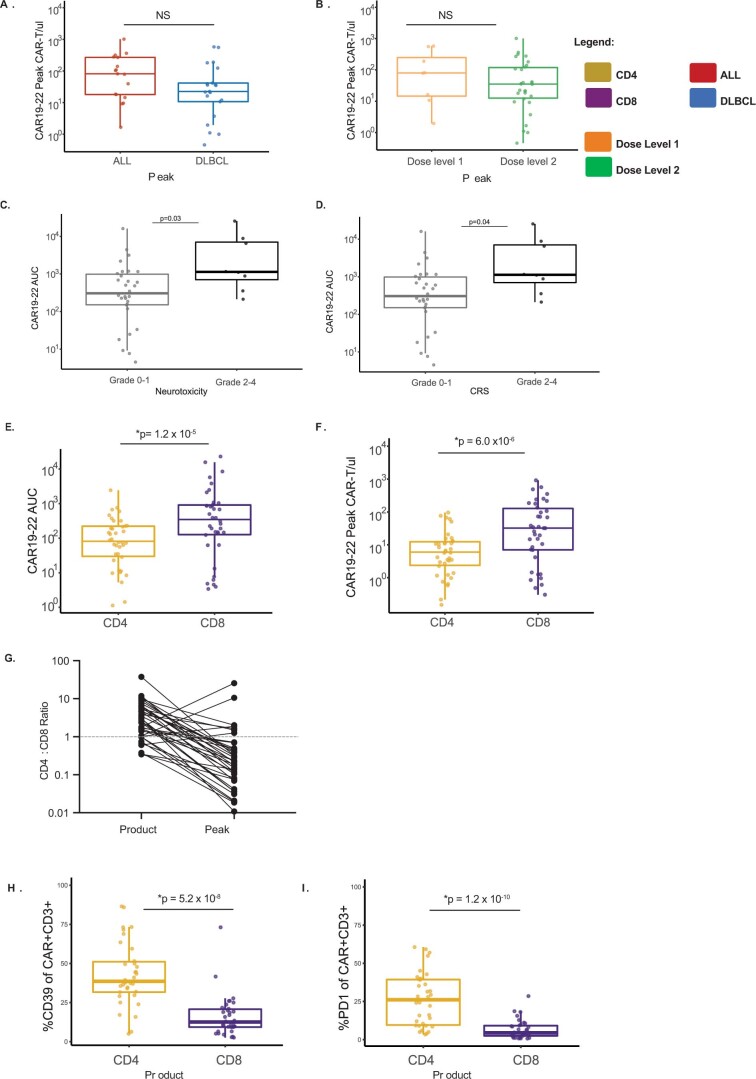
In vivo CAR-T cell expansion is CD8 predominant and relates to exhaustion phenotype of CAR-T products. **a**,**b** Peak CD19-22.BBZ cells as measured by flow cytometry compared by (**a**) disease type and (**b**) dose level. No significant difference observed. **c**,**d**. CD19-22.BB.z AUC was measured by the trapezoidal method from infusion until Day 60 post infusion.Grades 2-4 (**d**) CRS (p = 0.04) and (**c**) neurotoxicity (p = 0.03) associated with higher CD19-22.BBZ AUC. Comparisons by Wilcoxon rank sum. **e**, Area under the curve calculated by trapezoidal integration of CD4 and CD8 CD19-22.BB.z cells from infusion to 2 months post infusion. The AUC for CD8 was greater than that of CD4 (p = 1.2 × 10^−5^) **f**, Peak expansion of CD4 and CD8 CD19-22.BB.z-CAR T cells measured by flow cytometry. Peak CD8 CD19-22.BB.z-CAR was greater than that of CD4 (p = 6.0 × 10^−^^6^). Comparison in **e** and **f** performed by paired Wilcoxon signed rank test. **g**, Change in the CD4:CD8 ratio from CD19-22.BB.z infusion product to peak expansion, shows the outgrowth of CD4 cells during manufacturing is not seen in the patients at time of peak CAR expansion, where CD8 CAR T+ cells are more abundant. N = 38 patients for a-g. **h**,**i** Significantly higher percentage of CD4 CAR T cells in the infusion product express CD39 (p = 5.2 × 10^−8^) and PD-1 (p = 1.2 × 10^−^^10^) compared to CD8 CAR-T cells (Wilcoxon signed rank) (n = 34 patients). Gating on CD3+ CAR+ cells. Box plot center line corresponds to the median; hinges correspond to the 25^th^ and 75^th^ percentiles with the whiskers extending to the smallest or largest value at most 1.5 x IQR from the hinge. All tests were two-sided and not adjusted for multiple comparisons. Source data

CD19-22.BB.z-CAR cells were detected in the blood by both flow cytometry and quantitative PCR (qPCR) and peaked between days 10–14 postinfusion (Fig. 3k,l). The median peak number of circulating CD19-22.BB.z cells measured by flow cytometry was 36 CAR µl^−1^ (IQR = 13–136); by qPCR, it was 1,794 (IQR = 509–4,315) copies of the CD19-22.BB.z transgene per 50 ng of genomic DNA. Peak expansion did not significantly differ between LBCL and B-ALL or dose level (Extended Data Fig. 8a,b). Higher expansion as measured by area under the curve (AUC) was associated with increased CRS and neurotoxicity (Extended Data Fig. 8c,d). Despite a predominance of CD4^+^ cells in the manufactured CD19-22.BB.z-CAR products, CD8 CD19-22.BB.z cells demonstrated greater expansion relative to CD4 as measured by both AUC and peak levels (Extended Data Fig. 8e,f) and as seen by a peak CD4:CD8 ratio <1 in most patients (Extended Data Fig. 8g). Analysis of exhaustion markers on CAR^+^ cells within the product showed that CD4^+^ cells expressed higher levels of CD39 (ref. ^34^) and programmed cell death protein 1 (PD-1) (Extended Data Fig. 8h,i), potentially providing a basis for diminished in vivo expansion of CD4^+^ compared to CD8^+^ CAR T cells.

### Antigen expression in patients with progressive disease after CD19-22.BB.z-CAR

**Fig. 4:**
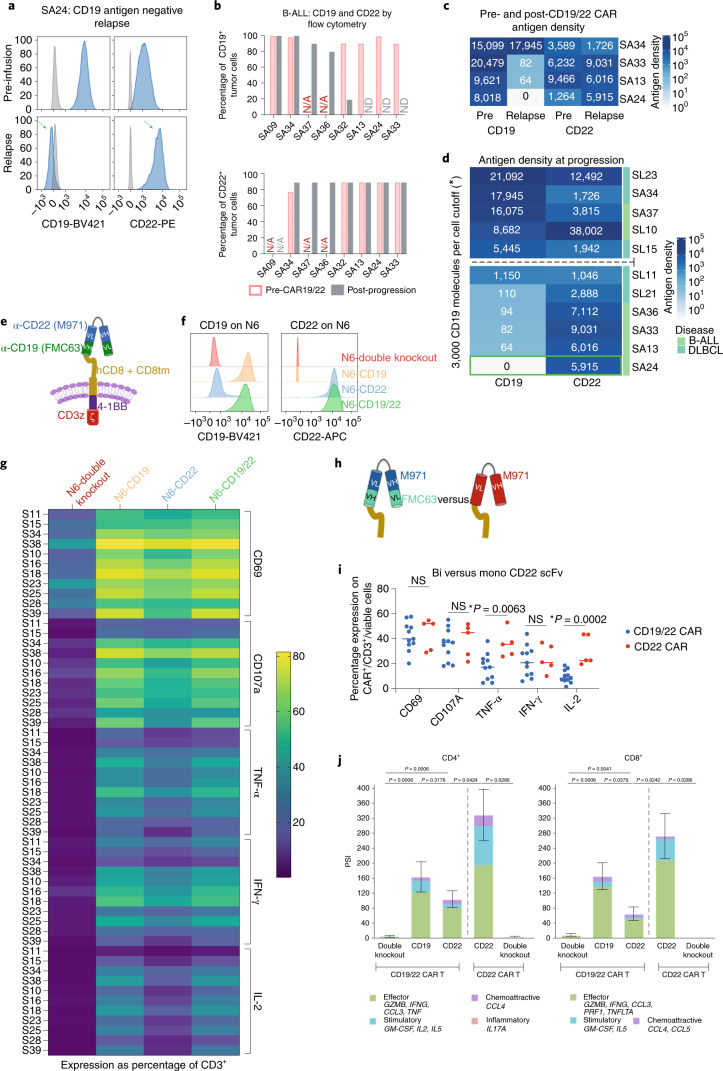
CD19 negative relapse with preserved CD22 site density after CD19-22.BB.z-CAR and diminished CAR T functionality against CD22. **a**, Antigen density of patient S24 demonstrating both CD19 and CD22 expression preCD19-22.BB.z (top) with loss of CD19 and preservation of CD22 at progression (bottom, green arrows). **b**, CD19 and CD22 assessment by conventional flow cytometry in patients with B-ALL pretreatment and postprogression demonstrates CD19 loss with CD22 preservation. **c**, In B-ALL, 3 of 4 patients with antigen quantification pre- and postCD19-22-CD.BB.z demonstrated loss of CD19 expression associated with preserved CD22 expression. **d**, CD19 and CD22 antigen density at progression (*n* = 11 patients) after CD19-22.BB.z, with patient S24 highlighted in green. Six patients had <1,150 CD19 molecules per cell with a median CD22 of approximately 6,000 molecules per cell. Dashed line denotes the cutoff at 3,000 CD19 molecules per cell. **e**, Schematic of CD19-22.BB.z bispecific CAR, displaying the loop structure. **f**, Histogram of CD19 and CD22 expression on NALM6 lines tested in **g**–**j**. **g**, ICS heatmap representing the secretion or expression of CD69, CD107a, TNF-α, IFN-γ and IL-2 from CD19-22.BB.z infusion products (*n* = 11 individual products) stimulated with the NALM6 tumors lines from **f**. The heatmap shows greater activation and secretion of cytokines with stimulation with N6-CD19 and N6-CD19/22 lines versus N6-CD22 stimulation. **h**, Schematic of bispecific CD19-22.BB.z versus monospecific CD22.BB.z. **i**, ICS stimulation of CD19-22.BB.z (*n* = 11) versus monospecific CD22.BB.z (*n* = 5) CAR products against the CD22^high^ cell line shows increased cytokine secretion of IL-2 and TNF-α through the monospecific CAR22.BB.z (two-tailed *t*-test). **j**, Using the single-cell IsoPlexis platform, stimulation of clinical products (*n* = 7 individual products) with N6-CD19 showed a higher PSI compared to N6-CD22. The CD22 scFV on the bispecific CAR had lower PSI compared to the monospecific CAR22.BB.z (*n* = 4 individual products) when stimulated with N6-CD22 (Mann–Whitney *U*-test). Source data

**Extended Data Fig. 9:**
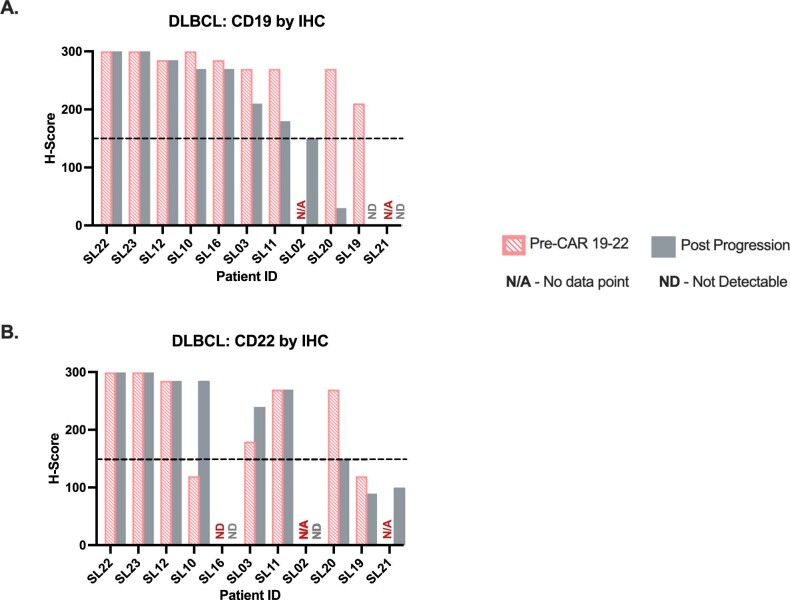
Site density change after CD19-22.BB.z. **a**, Waterfall plot of assessment of CD19 for patients with LBCL by IHC H-score pre-CD19-22.BB.z and post progression. Cutoff for CD19 positivity was 150. **b**, Waterfall plot of assessment of CD22 in patients with LBCL by IHC H-score pre-CD19-22-CD8.BB.z and post progression (n = 11 patients for a-b). Cutoff for CD22 positivity was 150. Source data

We next quantified CD19 and CD22 antigen expression at the time of progression after CD19-22.BB.z-CAR. Flow cytometry, as illustrated by patient SA24 (Fig. 4a), demonstrated that 5 of 10 patients with B-ALL with progression had negative or low CD19 expression with preserved CD22, using a 90% threshold (Fig. 4b and Table 2). Paired pre- and posttherapy antigen quantification in four patients with B-ALL confirmed decreased CD19 expression in three patients with no change in CD22 expression density (Fig. 4c). In 14 patients with LBCL biopsied at progression, 3 were CD19^−/lo^ (Extended Data Fig. 9a) by IHC. CD22 expression was not required for trial enrollment and pretreatment CD22 expression was heterogenous (Extended Data Fig. 9b); three patients had CD22^−/lo^ LBCL and two were undetermined. All 6 patients with a pretreatment CD22 H-score >150 maintained CD22 positivity. Eleven patients with B-ALL or LBCL had quantitative flow cytometry at progression; 6 patients had low CD19 with ≤3,000 molecules per cell (Fig. 4d), including 1 patient with LBCL with a CD19 H-score >150. The median CD22 expression in patients with low CD19 was approximately 6,000 molecules per cell. Overall, 4 out of 14 (29%) patients with LBCL were CD19^−/lo^ at progression after therapy with CD19-22.BB.z-CAR (Extended Data Fig. 9a and Fig. 4d).

### CD19-22.BB.z-CAR T secretes less cytokine when stimulated through the CD22 scFv

**Extended Data Fig. 10:**
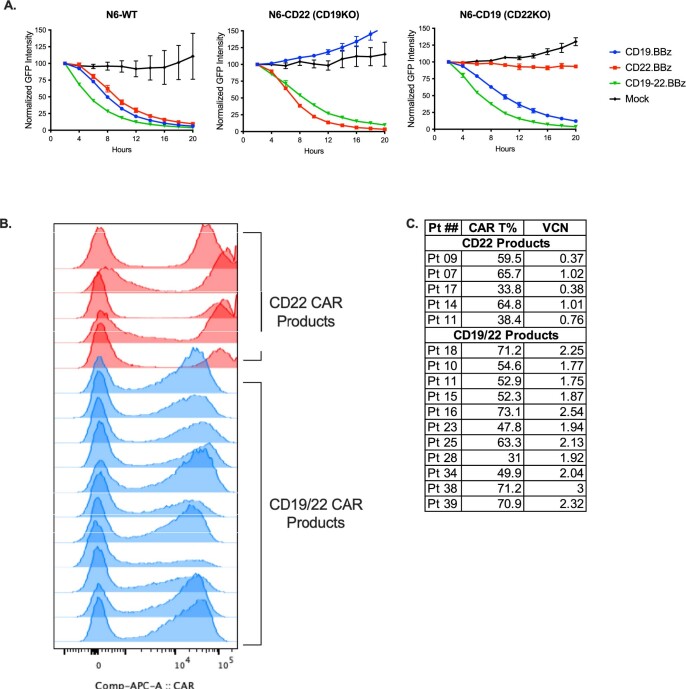
Functional assessment of CD19-22 scFvs by Incucyte and comparison of MFI, transduction efficiency and VCN of CD19-22.BB.z-CAR and CD22-BB.z.CAR. **a**, Incucyte shows tumor killing after co-culture with CD19-22, CD19, CD22, or Mock T cells, is mediated successfully through either the CD19 scFv or CD22 scFv (n = 1 donor, technical triplicates) **b**, Mean fluorescence intensity measured via a fluorescently tagged CD22 molecule.**c**, CAR transduction efficiency and vector copy number. For b-c, n = 5 CD22-BB.z products, 11 CD19-22.BB.z. Source data

The pattern of CD19^−/lo^ relapse with CD22 preservation suggested that T cells expressing the CD19-22.BB.z-CAR exert significant immune pressure against CD19 but not against CD22. In vitro models demonstrated that CD19-22.BB.z-CAR was active against CD19/CD22^+^ cell lines (Extended Data Fig. 10a). To address this, we compared the relative potency of the signal delivered via the CD19 scFv versus the CD22 scFv within the CD19-22.BB.z-CAR (Fig. 4e) using single-cell assays. Using samples from 11 manufactured cell products, we measured the intracellular cytokine secretion (ICS) of CD19-22.BB.z-CAR T cells after coculture with double-positive NALM6 (N6-CD19/22, approximately 20,000 CD19 molecules per cell and approximately 50,000 CD22 molecules per cell), NALM6 where CD22 had undergone knockout via CRISPR–Cas9 (N6-CD19, approximately 20,000 CD19 molecules per cell, 0 CD22 molecules per cell), NALM6 where CD19 had undergone knockout via CRISPR–Cas9 (N6-CD22, approximately 40,000 CD22 molecules per cell, 0 CD19 molecules per cell) or NALM6 with both CD19 and CD22 genetically deleted (N6-double knockout) (Fig. 4f). These site densities were higher than those seen in patient samples^21,28,35^ and have been associated with high levels of cytokine secretion in in vitro models^21^.

Using ICS, CD19-22.BB.z-CAR manufactured products appeared activated as measured by CD69 expression after coculture with N6-CD19 and less activated with N6-CD22 (*P* < 0.0001). Higher expression with N6-CD19 compared with N6-CD22 was seen with CD107 (*P* < 0.0001), tumor necrosis factor-α (TNFα) (*P* < 0.0001), interleukin-2 (IL-2) (*P* < 0.0001) and interferon-γ (IFN-γ) (*P* < 0.0001) (Fig. 4g).

Decreased PSI and ICS resulting from CD22 as opposed to CD19 stimulation suggested decreased potency of the CD22 scFV. We next compared the activity of the CD22 scFv of CD19-22.BB.z-CAR against the identical scFV in a monospecific CD22.BB.z-CAR (Fig. 4h)^22,31,36^. We tested good manufacturing practice (GMP)-manufactured CD22.BB.z-CAR T cell products (*n* = 5) from patients with relapsed/refractory LBCL or B-ALL enrolled on an ongoing clinical trial (NCT04088890) against N6-double knockout and N6-CD22. The mean fluorescence intensity of monospecific CAR on T cells was higher than that of the bispecific CAR (Extended Data Fig. 10b,c). We found that CD19-22.BB.z-CAR and CD22.BB.z-CAR T cells manifested similar levels of activation as measured by CD69 expression and IFN-γ and CD107 secretion (Fig. 4i). However, CD22.BB.z-CAR T cells demonstrated significantly higher levels of TNF-α (*P* = 0.0063) and IL-2 (*P* = 0.0002) secretion compared to CD19-22.BB.z-CAR T cells. Similarly, single-cell cytokine secretion using the IsoPlexis platform showed a higher PSI for CD22.BB.z-CAR T cells compared to CD19-22.BB.z-CAR in the presence of N6-CD22 (*n* = 4; Fig. 4j). Single-cell cytokine secretion was assessed with the 32-plex human panel^27^ after stimulation of CD4 and CD8 CAR^+^ cells with the N6-CD19/22, N6-double knockout, N6-CD19 or N6-CD22 N6 lines (*n* = 7). Overall, CD19 stimulation with N6-CD19 yielded a higher polyfunctional strength index (PSI) testing CD8CAR^+^ products (*P* = 0.04) compared to N6-CD22 stimulation (Fig. 4j). Together, these data demonstrate that both CD19 and CD22 scFvs contained within CD19/22.BB.z-CAR can signal in response to target recognition but cytokine levels induced after CD22 scFv ligation in the bispecific CAR are lower compared to CD19 scFv ligation. In contrast, CD22 scFv ligation in the monospecific CAR resulted in higher levels of cytokines than the bispecific CD22 scFV. The reduced potency of the CD22 scFV provides a mechanism for relapse with CD19^−/lo^ disease with preserved CD22 expression in the population studied here.

## Discussion

CD19 loss has been frequently identified after CAR19 in B-ALL across numerous studies incorporating variable scFvs and costimulatory domains^5–7,15,16,37,38^. In LBCL, case reports have also described CD19 loss after CAR19^35,39–41^ but a systematic analysis of CD19 expression after CAR19 in LBCL has not been conducted. In this study, we evaluated CD19 expression in 44 patients with LBCL treated with axi-cel at our institution and found that pretreatment quantitative flow cytometry was more sensitive than conventional IHC in identifying lower antigen levels associated with future progression. Quantitative flow cytometry could serve as a predictive biomarker in characterizing antigen modulation as a mechanism of resistance to CAR therapeutics.

Several studies have demonstrated the efficacy of simultaneous targeting multiple antigens in preclinical models using a variety of CAR configurations to endow multi-specific antigen recognition^22,42–46^. Early clinical trial results involving tandem CAR T cells targeting CD19 and CD20 have shown promising results with low reported rates of CD19 loss at the time of progression^47,48^. The CD19-22.BB.z-CAR used in this trial is a single molecule consisting of two scFvs engineered in a loop orientation^22^, which demonstrated activity in vitro and in xenograft models^32^. The clinical data presented in this study demonstrated that CD19-22.BB.z-CAR T cells were clinically active in both B-ALL, with 82% achieving an MRD^−^ CR, and in LBCL with an ORR of 62%. Toxicity was low: 5 and 11% experienced grade ≥3 CRS or neurotoxicity, respectively. Although we observed relapses with absent or low CD19 expression, a definitive determination of whether these rates are reduced compared to those observed with monospecific CAR19 therapeutics cannot be determined in this single-arm trial given the limited data available regarding antigen^−/lo^ relapse and the wide variability in antigen loss reported across studies.

However, the antigen expression pattern observed at relapse in B-ALL was consistent with CD19-22.BB.z-CAR delivering significant immune pressure on the CD19 antigen, whereas the lack of decrease or loss of CD22 expression suggests more limited immune pressure on the CD22 target. Experience with monospecific CD22-CAR T cells clearly demonstrated resistance associated with CD22^lo^ B-ALL, which was not observed in this trial^22^. In LBCL, CD22^lo^ disease at relapse was seen; however, CD22 expression before therapy was heterogeneous and absent in some. Our data show that CD22 scFv ligation in the bispecific CAR provided less cytokine secretion compared to CD19 scFv. These results suggest cytokine production could be a clinically meaningful product quality attribute to predict CAR potency in vivo and illustrate the challenges of delivering equivalent potency across targets in the context of multi-specific chimeric antigen receptors.

At present, much of CAR engineering is empiric and multiple approaches to simultaneous targeting of CD19 and CD22 are currently under study^32,49–52^. Engineering an optimal monospecific CAR is dependent on numerous factors, including prevention of tonic signaling,^53^ optimizing hinge length,^54,55^ hinge/transmembrane domain^21^ and optimizing the distance between the target epitope and tumor cell membrane^27,56,57^. In human trials, numerous chimeric antigen receptors targeting CD22 have demonstrated that small alterations in structure can result in loss of activity^49,58^. In the context of a tandem CAR (linked scFvs), optimal engineering also requires engineering an optimal bispecific receptor^59,60^, which may be particularly challenging for the CD22 target. When compared to a monospecific CAR with the same scFV, the CD19-22.BB.z-CAR demonstrated decreased TNF-α and IL-2 secretion. The threshold for CAR T cell IL-2 secretion has previously been shown to be higher than that of IFN-γ^20^ and may better discriminate CAR efficacy. Our results suggest that engineering iterations should be guided by careful studies of single-cell CAR polyfunctionality incorporating cytokine production as a critical quality attribute.

We also observed a significant incidence of CD19^+^ relapse, as reported in many previous trials of CAR19 for B-ALL and LBCL^61^, suggesting that potency toward the CD19 target may also be insufficient in some patients. Improvements in CAR manufacturing could prevent antigen^+^ resistance, which likely results from T cell failure. Defined composition of CD4:CD8 CAR cells and preferential administration of T cell subsets, such as T_CM_^62,63^, have been hypothesized to mediate optimal CAR activity^1^. We found our manufacturing process selectively enriched for T_CM_ and T_SCM_ with low T_EMRA_ but biased the final product toward CD4 predominance. Furthermore, CD4 cells produced with our process expressed higher levels of CD39 and PD-1, which are associated with exhaustion, suggesting that the manufacturing process may not generate an optimal final product composition. Due to these findings, we paused trial enrollment to modify our manufacturing process to attain a more balanced CD4:CD8 ratio, which may also allow CD4 CAR cells to maintain a less exhausted phenotype.

In summary, this work provides evidence that antigen^−/lo^ escape is a major pathway of resistance after CAR19 therapy for LBCL and quantitative antigen density in LBCL correlates with outcomes after CAR T cell therapy. Using a bispecific CAR capable of simultaneously recognizing CD22 and CD19, we demonstrated safety and impressive clinical activity in B-ALL. The 6-month PFS in LBCL (29%, 95% CI 12–48%) in this trial was similar to tisagenlecleucel^64^. Therefore, the lymphoma arm was closed in this study with enrollment ongoing in patients with B-ALL. Resistance to the bispecific CAR was associated with CD19^+^CD22^+^ relapse, probably reflecting intrinsic limitations of CAR T cells, as well as CD19^−/lo^ but CD22^+^ relapse, implying inadequate immune pressure on the CD22 target. Our data also illustrate the value of cytokine production as a key product quality attribute for credentialing the potency of CAR T cells. Future work is needed to optimize multi-specific targeting by CAR T cells to improve the efficacy of this class of therapeutics both in B cell malignancies and other solid and liquid cancers.

## Methods

### Trial design

We conducted a single-center phase I clinical trial of CD19-22.BB.z-CAR in adult patients with relapsed/refractory LBCL and B-ALL at Stanford University Medical Center. The study was approved by the internal review board and registered with ClinicalTrials.gov (NCT03233854). A trial-specific safety monitoring committee was chartered for safety and trial conduct; it consisted of internal Stanford and external CAR T expert members. In addition, data monitoring was overseen by Stanford’s institutional Data Safety Monitoring Committee. Informed written consent was provided by all patients in accordance with the Declaration of Helsinki (2013). The full clinical trial protocol is included in the Supplementary Information. The dose escalation phase enrolled both histologies in a single arm and permitted enrollment on three dose levels using a 3 + 3 design: DL1 of 1 × 10^6^ cells kg^−1^; DL2 of 3 × 10^6^ cells kg^−1^; and DL3 of 1 × 10^7^ cells kg^−1^. Primary outcomes were feasibility of manufacture of CD19-22.BB.z-CAR as well as safety of CD19-22.BB.z-CAR. Feasibility of manufacture was defined as greater than 80% of products meeting the protocol-specified dose. Safety was defined by incidence and severity of DLTs at each of the dose levels tested; the maximal tolerated dose was defined as the dose level immediately below the dose level where incidence of DLTs was >30%. After dose escalation, disease-specific dose expansion cohorts could be enrolled at either the maximum tolerated dose or highest dose level tested. Secondary outcomes included efficacy of CD19-22.BB.z-CAR to induce clinical response at three months for LBCL and one month for B-ALL. A futility analysis was conducted after 15 individuals were enrolled at the recommended phase 2 dose. In LBCL, futility was defined as a ≤33% ORR at the 3-month time point. In B-ALL, futility was defined as a ≤40% ORR at the 1-month time point. Exploratory objectives included the rate of CD19^−^ and/or CD22^−^ relapse after CD19-22.BB.z-CAR, site density of CD19 and CD22 of malignant cells and expansion and persistence of CD19-22.BB.z-CAR after infusion.

### Patient enrollment and eligibility

Patients eligible for leukapheresis were ≥18 with LBCL or B-ALL relapsed or refractory after ≥2 lines of therapy. If applicable, previous autologous or allogeneic HCT were considered as a line of therapy. Patients with LBCL must have received an anthracycline and an anti-CD20 monoclonal antibody as part of previous therapy. Transformed indolent lymphomas, including Richter transformation, were eligible. Measurable disease by PET/CT was required. For B-ALL, either morphological disease (including extramedullary disease) or detectable MRD with <5% marrow blasts were acceptable; CNS involvement was allowed. Expression of CD19 on malignant cells was required by either flow cytometry or IHC. Previous anti-CD19-directed therapy, including previous CAR T, was allowed provided <5% of circulating T cells expressed the previous CAR. At least two weeks or five half-lives, whichever was shorter, must have elapsed since any previous systemic therapy at the time of leukapheresis and all previous toxicities must be stable or recovered to grade 1 or lower. Adequate marrow function was required unless cytopenias were felt to be due to underlying leukemia/lymphoma: an absolute neutrophil count ≥750 µl, platelet count ≥50,000 µl and absolute lymphocyte count ≥150 µl. Adequate organ function was defined as creatinine ≥2 mg ml^−1^, serum aspartate aminotransferase or alanine aminotransferase less than ten times the upper limit of normal, total bilirubin ≤1.5 mg dl^−1^, cardiac ejection fraction ≥45%, no clinically significant electrocardiogram findings, no clinically significant pleural effusion and baseline oxygen saturation ≥92% on room air. Exclusion criteria included: history of previous malignancy within 3 years; presence of uncontrolled bacterial, viral or fungal infection or infection requiring intravenous antibiotics; known infection with human immunodeficiency virus, hepatitis B (HBsAg^+^) or hepatitis C (anti-hepatitis C virus^+^); CNS disorder impairing one’s ability to evaluate neurotoxicity; history of autoimmune disease requiring systemic immunosuppression within previous 2 years; history of clinically significant cardiac disease within 12 months of enrollment.

After leukapheresis, bridging therapy was permitted at the investigator’s discretion. Conditioning chemotherapy, consisting of fludarabine 30 mg m^−2^ and cyclophosphamide 500 mg m^−2^, was administered on day −5 through day −3 before infusion. Patients were enrolled to receive CD19-22.BB.z-CAR between 12 September 2017 and 19 November 2019. Data were locked as of 15 June 2020. Dose escalation and production feasibility were determined by consecutive patients irrespective of disease type. After dose escalation, separate LBCL and B-ALL cohorts were expanded to treat 15 patients in each cohort at the recommended phase II dose.

### Toxicity assessment

In the dose escalation phase, CRS was graded according to the Lee criteria^66^ and neurotoxicity-graded by the Common Terminology Criteria for Adverse Events v.4.0.3. Patients in the cohort expansion were graded according to the American Society for Transplantation and Cellular Therapy (ASTCT) criteria for CRS and ASTCT immune effector cell-associated neurotoxicity syndrome criteria for neurotoxicity^67^. For uniform reporting in the article, patients in the dose escalation group were regraded according to the ASTCT criteria. Adverse events were captured for all treated patients until disease relapse or death.

### Response assessment

Response for patients with LBCL and patients with B-ALL with extramedullary disease without concurrent bone marrow or CNS involvement was assessed using the Lugano PET/CT criteria^67^. For all other patients with B-ALL, CR was defined as <5% bone marrow blasts by morphology. MRD negativity was defined as a bone marrow blast percentage <10^−4^ by multiparameter flow cytometry.

### Axi-cel patients

Consecutive patients with LBCL treated with standard-of-care axi-cel between 27 December 2017 and 9 April 2020 were identified. Patients were consented for collection of clinical data as well as blood and lymph node sampling on a clinical outcomes biorepository protocol. Patients with available tissue samples for IHC and/or quantitative flow cytometry were included in the analysis. The protocol was approved by the Stanford Internal Review Board (no. 43375). Clinical data were obtained retrospectively from the chart review. Treatment response was assessed radiographically according to the Lugano criteria.

### CD19-22.BB.z production

CD19-22.BB.z products were manufactured in the automated closed-system CliniMACS Prodigy in a 7–11 d manufacturing process. All days provided in this CAR T production section are reflective of the manufacturing schema (Extended Data Fig. 2). The frozen patient apheresis was washed on the Lovo (Fresenius Kabi) and rested overnight in low-dose IL-2 before loading on the CliniMACS Prodigy. On day 0, the apheresis was enriched for CD4 and CD8 T cells before T cell activation with TransAct (Miltenyi Biotec). On day 1, T cells were transduced with CD19-22.BB.z lentiviral vector (Fig. 2a) at a multiplicity of infection of 40. TransAct was subsequently washed out on either day 3 (new matrix) or day 5 (old matrix), followed by a series of media exchanges. On days 7, 9 or 11, when target dose was achieved, the final product was collected, sampled for quality control testing and cryopreserved. The product release criteria are listed in Supplementary Table 1. Iterative improvements to the manufacturing process were implemented to shorten the vein-to-vein time during the course of the trial.

### IHC

Tissue microarrays were constructed with duplicate 0.6-mm cores of formalin-fixed paraffin-embedded tissue from diagnostic biopsies^68^. Additional whole-tissue sections were evaluated from cases when available.

Immunohistochemical studies were performed on 4-mm-thick sections of the formalin-fixed paraffin-embedded tissue in tissue microarray or whole-section form. Automated immunostaining was performed using the Leica BOND-III (Leica Biosystems). Slides were stained with antibodies against CD19 (clone BT51E; prediluted mouse monoclonal antibody; Leica Biosystems) and CD22 (clone FPC1; prediluted mouse monoclonal antibody; Leica Biosystems).

The intensity of staining (0, negative; 1, weak; 2, moderate; 3, strong) and percentage of tumor cells showing staining (0–100%) were evaluated independently then jointly scored by 2 pathologists (S.Y. and Y.N.). An H-score of 0–300 was generated by multiplying the intensity of positivity by the percentage of tumor cells with staining.

### Detection of CD19-22.BB.z cells

CD19-22.BB.z cells were detected using the CD19 anti-idiotype antibody developed at the MD Anderson Cancer Center^69^. The CD19 anti-idiotype antibody was conjugated to DyLight 650 (Thermo Fisher Scientific) using an antibody labeling kit and stored at −80 °C for downstream flow cytometry use.

### Phenotyping of manufacturing samples at apheresis, enrichment and final product collection

This section describes the methods for the phenotyping shown in Fig. 2. All samples were washed in FACS buffer (1× PBS, 2% FCS), stained for a minimum of 30 min at 4 °C before additional washes and running flow cytometry on the MACSQuant Analyzer 10 (Miltenyi Biotec). The MACS Comp bead kit (catalog no. 130-097-900; Miltenyi Biotec) was used for compensation controls. The antibodies used for these experiments are listed in Supplementary Table 2.

### Flow cytometry for phenotyping and exhaustion profiling

All samples were washed in FACS buffer (1× PBS, 3% FCS), stained for a minimum of 30 min at 4 °C before additional washes and running flow cytometry. UltraComp eBeads (catalog no. 01-2222-41; Invitrogen) were used for compensation controls and stained with the respective antibody from the antibody index below. Samples were run on the LSRFortessa X-20 (BD Biosciences) and stained using the antibodies from antibody index shown below.

### Exhaustion and T cell subset phenotyping panel

This section describes the methods for the intracellular cytokine panel in Figs. 2 and 4 and Extended Data Fig. 2. Samples were stained with a panel backbone (CD3, CD4, CD8, CAR, viability) first. Subsequently, samples were split into two for either phenotyping of T cell subsets (CD45RA, CD45RO, CCR7, CD62L, CD95) or exhaustion markers (CD39, LAG3, PD-1); all samples were prepared and washed as described above. Analysis for this and the previous two sections were performed in FlowJo v10.5.3 (FlowJo LLC).

### NALM6 tumor line antigen density check by flow cytometry

This section describes the methods used for the intracellular cytokine panel in Fig. 4. All NALM6 lines were divided into two and stained with either CD19 or CD22 antibody and prepared and washed as described above.

### Coculture of CD19-22.BB.z/CD22.BB.z product with NALM6 target cells

This section describes the methods used for the intracellular cytokine panel in Fig. 4. Clinical CAR T products were thawed and rested overnight before incubation in coculture assays with the NALM6 lines described above. During this 6–7 h coculture at 37 °C, cells were coincubated with monensin (Thermo Fisher Scientific) and CD107a. A phorbol myristate acetate/ionomycin (Sigma-Aldrich) condition was included as a positive control, along with no monensin, no CD107a or no tumor cells as negative controls. After coculture, cells were washed with FACS buffer and resuspended in an extracellular antibody cocktail consisting of CD3, CD4, CD8, CD69, CAR, LIVE/DEAD and CD107a and incubated for 30 min at 4 °C. Cells were subsequently washed out with FACS buffer before intracellular cytokine staining using the Fixation/Permeabilization Solution Kit (BD Biosciences). The kit protocol was followed to fix and permeabilize CAR T products, followed by resuspension in an intracellular antibody cocktail consisting of IFN-γ and TNF-α and incubated overnight at 4 °C. Cells were washed the following morning in FACS buffer before proceeding with flow cytometry run on the LSRFortessa X-20. The full panel of antibodies is shown in Supplementary Table 3.

### Real-time peripheral blood CAR T phenotyping assay

A high-dimensional immunophenotyping flow cytometry panel was designed for immune profiling of CAR T and B cells in real time on the LSR II (BD Biosciences) and analyzed in Cytobank. Peripheral blood mononuclear cells (PBMCs) were isolated from fresh whole blood by gradient centrifugation on Ficoll (Ficoll-Paque PLUS; Sigma-Aldrich). Between 2–5 million PBMCs were stained with fixable Live/Dead aqua (Invitrogen) amine-reactive viability. Cells were then preincubated with Fc block (TruStain FcX; BioLegend) for 5 min before staining at room temperature with the panel of antibodies listed in Supplementary Table 4. CD19-22.BB.z cells were used as the positive batch control for the daily staining experiments. At least 10^6^ cells were acquired unless restricted by the number of cells isolated from 8 ml of whole blood. The assay limit of detection for CAR T cells was calculated as 1 in 10^4^ of total acquired PBMCs.

### qPCR measurement of in vivo CD19-22.BB.z-CAR expansion

DNA was extracted from whole blood (2 × 10^6^–5 × 10^6^ PBMCs) using the QIAamp DNA Mini Kit (catalog no. 51306; QIAGEN) at baseline and on days 7, 14, 21, 28, 90 and 180 postCAR infusion. CAR presence was measured by qPCR using the primer and probe sequences provided in Supplementary Table 5. For the standard curve, a custom Minigene plasmid (Integrated DNA Technologies) was designed containing a partial CD19-22.BB.z sequence and a partial albumin sequence, which served as a control for normalization. The standard curve contained a tenfold serial dilution of plasmid between 5 × 10^6^ and 5 × 10^0^ copies. Both plasmid and patient DNA from each time point were run in triplicate, with each reaction containing 5 µl of DNA (50 ng total), 100 nM of forward and reverse albumin primers (or 100 nM of forward and 200 nM reverse CD19-22.BB.z primers), a 150-nM probe suspended in 10 µl of TaqMan Fast Universal PCR Master Mix (2×), No AmpErase UNG or equivalent (Thermo Fisher Scientific) and 5 µl of TE buffer (catalog no. AM9935; Invitrogen). The Bio-Rad CFX96 Touch Real-Time PCR Detection System was used for qPCR with 20 µl per reaction. The quality metrics of all qPCR standard curve results were *R*^2^ > 0.99, −3.38 > slope > −3.71 and efficiency >86%.

### IsoPlexis for 32-plex cytokines

CD19-22.BB.z or CD22.BB.z products were magnetically selected into CD4 or CD8 T cells using microbeads (Miltenyi Biotec). CD4^+^ and CD8^+^ populations were subsequently cocultured with NALM6-double knockout, NALM6-CD19 or NALM6-CD22 at a 1:2 ratio (T cell:tumor) for 20 h. After incubation and a viability check, tumor cells were depleted from the coculture and the remaining T cells were stained with CD22-Fc-AF647 for CD19-22.BB.z detection. Subsequently, 30,000 viable cells were loaded onto the 32-plex human polyfunctional strength single-cell IsoCode chips (IsoPlexis); duplicate chips were run when sample was available. Chips were loaded into the IsoLight machine for scanning and data were analyzed using the IsoSpeak software v.2.8.0.0 (IsoPlexis). Statistical significance between stimulation conditions for single-cell polyfunctionality, percentage and PSI was done by Mann–Whitney *U*-test.

### Tumor-killing IncuCyte proliferation assay

The tumor-killing ability of bispecific CD19-22.BB.z and single CD19 and CD22 CAR T cells was assessed in a coculture assay with either NALM6-wild type, NALM6-CD19 knockoutor NALM6-CD22 knockout at an effector:target ratio of 3:1. CD19-22.BB.z cells, all from the same donor, were grown on the CliniMACS Prodigy and CD19 and CD22 CAR T cells were grown using small-scale plate-based processes. All NALM6 cell lines were green fluorescent protein (GFP)^+^; untransduced T cells were included as a mock condition. Tumor killing was measured as a decrease in GFP overtime using the IncuCyte System (Sartorius) and normalized.

### Antigen density assessment

Specimens were processed within 24 h of collection (mean ± h), stained using the antibody combination listed in Supplementary Table 6 and analyzed on the FACSLyric system (BD Biosciences). The median fluorescence intensities of CD19, CD20 and CD22 were determined under saturating antibody conditions for antigen density measurements; the antibody bound per cell was calculated by calibration with Quantibrite phycoerythrin beads (BD Biosciences) and custom BD Biosciences quantitation beads for allophycocyanin and Brilliant Violet 421 (BD Biosciences) after controlling for the fluorophore to antibody ratio.

### Measurement of cell-free tumor DNA

Tumor DNA was extracted from archival paraffin-embedded tissue; PCR amplification of the IgH-VDJ, IgH-DJ and Igκ/Igλ regions using universal consensus primers was performed by NGS to determine the tumor clonotype(s) (Adaptive Biotechnologies)^70^. ctDNA was measured from plasma extracted from blood obtained in EDTA tubes at pre-, 0, 7, 14, 21, 56 and 90 d postCD19-22.BB.z-CAR infusion.

### Statistical analysis

Descriptive statistics were enumerated by median and IQR for continuous variables and counts and percentages for categorical variables. Fisher’s exact (independent) and McNemar’s (dependent) tests evaluated the association between categorical variables and Spearman correlation was used for the association between two random continuous variables. Between-group comparisons for continuous variables were performed using either a Student’s *t*-test or Wilcoxon signed-rank test. All tests were two-sided. To associate CD19 antigen density with risk of progression, Firth’s penalized logistic regression was used due to the small sample size. Overall survival was defined as the time from infusion to death from any cause. PFS was defined as the time from infusion to either disease progression or death from any cause. Patients were censored at the time of the last follow-up. Overall survival and PFS were estimated by Kaplan–Meier method. Response rates were summarized along with 95% Clopper–Pearson CIs. Analyses were exploratory and not adjusted for multiple comparisons. The statistical software packages used include R v.3.6.2 and Prism 8 (GraphPad Software). *P* values generated in Prism 8 did not go below *P* < 0.0001.

### Reporting Summary

Further information on research design is available in the Nature Research Reporting Summary linked to this article.

## Online content

Any methods, additional references, Nature Research reporting summaries, source data, extended data, supplementary information, acknowledgements, peer review information; details of author contributions and competing interests; and statements of data and code availability are available at 10.1038/s41591-021-01436-0.

## Supplementary information

Supplementary Information Supplementary Tables 1–6, Supplementary Figs. 1–4 and CD19-22.BB.z Trial Protocol.

Reporting Summary

## Data Availability

All requests for raw and analyzed data will be reviewed by the corresponding author to determine if the request if subject to any intellectual property or confidentiality considerations. Patient-related data not included in the paper were generated as part of clinical trials and may be subject to patient confidentiality. Any data and materials that can be shared will be released via a material transfer agreement. The source data for Figs. 1–4 and Extended Data Figs. 1–7 are provided with the paper. The CD19-22 bispecific CAR sequence is in the patent application (U.S. Provisional Patent Application no. 62/135442) filed on 19 March 2015, titled Dual Specific Anti-Cd22-Anti-Cd19 Chimeric Antigen Receptors; the amino acid sequence is provided in Supplementary Fig. 2.

## Source data

Source Data Fig. 1 Statistical source data.
Source Data Fig. 2 Statistical source data.
Source Data Fig. 3 Statistical source data.
Source Data Fig. 4 Statistical source data.
Source Data Extended Data Fig. 4 Statistical source data.
Source Data Extended Data Fig. 6 Statistical source data.
Source Data Extended Data Fig. 7 Statistical source data.
Source Data Extended Data Fig. 8 Statistical source data.
Source Data Extended Data Fig. 9 Statistical source data.
Source Data Extended Data Fig. 10 Statistical source data.
