# Supplementary material for: CAR T cells with dual targeting of CD19 and CD22 in adult patients with recurrent or refractory B cell malignancies: a phase 1 trial
Source: Nat Med. 2021 Jul 26;27(8):1419–31. doi: 10.1038/s41591-021-01436-0 (PMC8363505; doi:10.1038/s41591-021-01436-0)
Supplement: Supplementary file 1 — Supplementary Tables 1–6, Supplementary Figs. 1–4 and CD19-22.BB.z Trial Protocol. [file 41591_2021_1436_MOESM1_ESM.pdf]

nature portfolio

https://doi.org/10.1038/s41591-021-01436-0

Supplementary information

CAR T cells with dual targeting of CD19 and CD22 in adult patients with recurrent or refractory B cell malignancies: a phase 1 trial

In the format provided by the authors and unedited

Supplemental Data Table 1:

**Product Rapid-Release for Infusion Criteria Table**	
**Release Test**	**Acceptance Criteria**
Cell viability	≥ 70%
Cell number	± 20% of planned dose level
% CD3+ Cells	≥ 70%
% CD19-22.BB.z+ cells	≥10%
Endotoxin	< 5 EU/kg body weight
Mycoplasma	Negative
Gram Stain	Negative
Preliminary Sterility (3-5 day)	Negative
Preliminary Fungal (3-5 day)	Negative
qPCR-based Replication Competent Lentivirus (RCL)	Negative
VCN	FIO
Product Phenotyping/Composition	FIO
**Product Final Release and Lot Disposition Table**	
Sterility (14 days)	Negative
Fungal (42 days)	Negative
Cell-based RCL	Negative

Supplemental Data Table 2:

**Panel 1**			
**Manufacturer**	**Part Number**	**Antibody & Fluorochrome**	**Antibody Dilution**
Miltenyi	130-110-637	CD45-VioBlue (REA747)	1:100
Miltenyi	130-113-138	CD3-FITC (REA613)	1:100
Miltenyi	130-113-221	CD4-VioGreen (VIT4)	1:100
Miltenyi	130-113-155	CD8-APC-Vio770 (BW135/80)	1:100
Miltenyi	130-113-312	CD56-PE (REA196)	1:100
Miltenyi	130-113-393	CD16-PE (REA423)	1:100
Miltenyi	130-110-520	CD14-APC (REA 599)	1:100
Miltenyi	130-113-375	CD20-PE-Vio770 (LT20)	1:100

Supplemental Data Table 3:

**Antibody Index for Phenotyping and Exhaustion Profiling**			
**Manufacturer**	**Part Number**	**Antibody & Fluorochrome**	**Antibody Dilution**
BD Biosciences	564809	CD3-BUV496	1:100
Biolegend<br>Biolegend	317418<br>564305	CD4-APC-Cy7<br>CD4-BUV737	1:100<br>1:100
Biolegend	564912	CD8-BUV805	1:100
B. Jena, L. Cooper, *et al.*	Referenced above	CD19 Anti-Id-DL650/APC	1:600
Thermo<br>BD Biosciences	L34964<br>565388	Live/Dead Viability-BV450<br>Live/Dead Viability-BV786	1:100<br>1:100
BD Biosciences	563733	CD45RA-BV711	1:100
BD Biosciences	561271	CCR7-FITC	1:100
Biolegend	305624	CD95-BV421	1:100
Biolegend	328206	CD39-FITC	1:100
Biolegend	369320, 369318	LAG3-BV711	1:100
BD Biosciences	561272	PD-1-PE-Cy7	1:100
Biolegend	393304	CD57-BV605	1:100
Biolegend	328634	CD107a-BV605	1:100
Biolegend	310930	CD69-BV421	1:100
Biolegend<br>Biolegend	502509<br>563563	IFNg-PE<br>IFNg-BUV395	1:100<br>1:100
Biolegend	502940	TNFa-BV711	1:100
Biolegend	392504	CD19-APC	1:100
Biolegend	302510	CD22-APC	1:100
Biolegend	500326	IL-2-PE-Cy7	1:100

Supplemental Data Table 4:

**Panel 2**			
**Manufacturer**	**Part Number**	**Antibody & Fluorochrome**	**Antibody Dilution**
BioLegend	300406	CD3-FITC	1:50
BioLegend	363004	CD19-PE	1:50
BioLegend	367112	CD14-PE-Cy7	1:50
BD Pharmingen	565310	CD8-PerCP Cy5.5	1:50
B. Jena, L. Cooper, *et al.*	Referenced above	CD19 Anti-Id-DL650/APC	1:100
BioLegend	362554	CD56-APC-Fire750	1:50
Invitrogen	L-34965	L/D Fixable Aqua-BV405	1:50
BioLegend	302524	CD22-BV421	1:50
BioLegend	302334	CD20-BV605	1:50
BioLegend	300558	CD4-BV711	1:50
BioLegend	368528	CD45-BV785	1:50

Supplemental Data Table 5:

**qPCR Reagent**	**Sequence**
Albumin FAM Probe	5’ - CCT GTC ATG CCC ACA CAA ATC TCT CC - 3’
Forward Primer Albumin	5’ - GCT GTC ATC TCT TGT GGG CTG T - 3’
Reverse Primer Albumin	5’ - ACT CAT GGG AGC TGC TGG TTC - 3’
CD19-22 FAM Probe	5’ - TGT GAT TTC CAG CTT GGT GCC TCC - 3’
Forward Primer CD19-22	5’ - TCT GTC AGC AAG GCA ACA - 3’
Reverse Primer CD19-22	5’ - GGT CTG GCT AGG CTT CAC - 3’
CD19-22 Albumin Minigene® Plasmid	TCTGTCAGCAAGGCAACACCCTGCCCTACACCTTCGGCGGA<br>GGCACCAAGCTGGAAATCACAGGCGGCGGAGGATCCCAGGT<br>GCAGCTGCAGCAGTCTGGACCCGGCCTCGTGAAGCCTAGCC<br>AGACCCTGTCTCTGACCTGCGCCATCAGCGGGCTGGCCTTT<br>TGCTCACAAGCTTGGGGTTGCTGTCATCTCTTGTGGGCTGTA<br>ATCATCGTCTAGGCTTAAGAGTAATATTGCAAAACCTGTCATG<br>CCCACACAAATCTCTCCCTGGCATTGTTGTCTTTGCAGATGT<br>CAGTGAAAGAGAACCAGCAGCTCCCATGAGTCCCAAGCTAT<br>GTTCTTTCCTGCGTT

Supplemental Data Table 6:

**Panel 3**			
**Manufacturer**	**Part Number**	**Antibody & Fluorochrome**	**Antibody Dilution**
BD Biosciences	346586	Lambda-FITC	1:18.5
BD Biosciences<br>BD Biosciences	340708<br>340932	CD22-PE<br>CD22-APC	1:9.25<br>1:23
BD Biosciences	347213	CD34-PERCP	1:18.5
BD Biosciences	341102	CD10-PE-CY7	1:62
BD Biosciences	340940	CD20-APC	1:18.5
BD Biosciences	565121	CD5-APC-R700	1:62
BD Biosciences	653314	CD38-APC-H7	1:62
BD Biosciences	659477	CD19-BV421	1:18.5
BD Biosciences	647450	CD45-V500-C	1:62
BD Biosciences	663192	Kappa-BV605	1:62

Supplemental Figure 1: H-score components, % of tumor cells positive and staining intensity

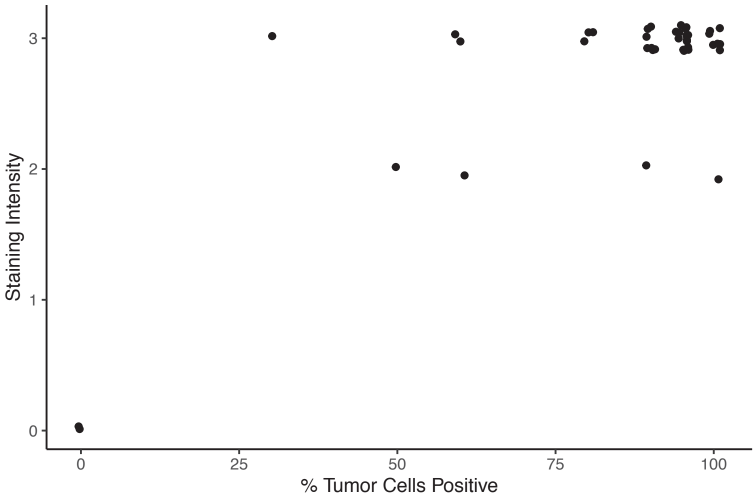

Supplemental Figure 2: 19-22.BB.z Amino Acid sequence

MLLLVTSLLLCELPHPAFLLIPDIQMTQTTSSLSASLGDRVTISCRASQDISKYLNWYQQ
KPDGTVKLLIYHTSRLHSGVPSRFSGSGSGTDYSLTISNLEQEDIATYFCQQGNTLPYT
FGGGTKLEITGGGGSQVQLQQSGPGLVKPSQTLSLTCAISGDSVSSNSAAWNWIRQS
PSRGLEWLGRTYYRSKWYNDYAVSVKSRITINPDTSKNQFSLQLNSVTPEDTAVYYCA
REVTGDLEDAFDIWGQGTMVTVSSGSTSGSGKPGSGEGSTKGDIQMTQSPSSLSASV
GDRVTITCRASQTIWSYLNWYQQRPGKAPNLLIYAASSLQSGVPSRFSGRGSGTDFTL
TISSLQAEDFATYYCQQSYSIPQTFGQGTKLEIKGGGGSEVKLQESGPGLVAPSQSLS
VTCTVSGVSLPDYGVSWIRQPPRKGLEWLGVIWGSETTYYNSALKSRLTIIKDNSKSQ
VFLKMNSLQTDDTAIYYCAKHYYYGGSYAMDYWGQGTSVTVSSASSGTTTPAPRPPT
PAPTIASQPLSLRPEACRPAAGGAVHTRGLDFACDIYIWAPLAGTCGVLLLSLVITLYCK
RGRKKLLYIFKQPFMRPVQTTQEEDGCSCRFPEEEEGGCELRVKFSRSADAPAYKQG
QNQLYNELNLGRREEYDVLDKRRGRDPEMGGKPRRKNPQEGLYNELQKDKMAEAYS
EIGMKGERRRGKGHDGLYQGLSTATKDTYDALHMQALPPR

Supplemental Figure 3

CAR-FACS flow assay validation

CAR-FACS FMC63	
**Precision: Inter-assay**	4.0 %and 2.2 % CV for CD4- and CD8-CART; n=50
**Precision: Intra-assay**	<2% CV (n=12; replicates)
**Analytical sensitivity**	<u>Cell frequency</u>: 1 in 10,000 acquired cells depending on number of cells collected<br><u>Signal intensity</u>: 500 copies per cell to get 10 counts per cell
**Analytical specificity including interfering substances**	Antibody specificity described Jena B, et al. PLoS One. 2013; 8(3): e57838.<br>No cross reactivity on untraduced cells
**Reportable range**	~0.01%-100%, based on collection of 10^6 PBMC events acquisition
**Reference interval (normal range)**	Not applicable
**Quality control and improvement procedures**	1. Positive and negative Control cells run<br>2. Single stained beads<br>3. Multicolor-197 beads calibrating the lasers and PMTs across each instrument set up, normalizing for instrument variability across years of trial
**Sample robustness**	100% robust ness on viably frozen cells at > 65% recovery and >75% viability when thawed

Reproducibility of the same positive control cells run on multiple occasions followed through one year. Aliquots of CAR-T cells viably frozen; Cells thawed, stained and included in each experiment

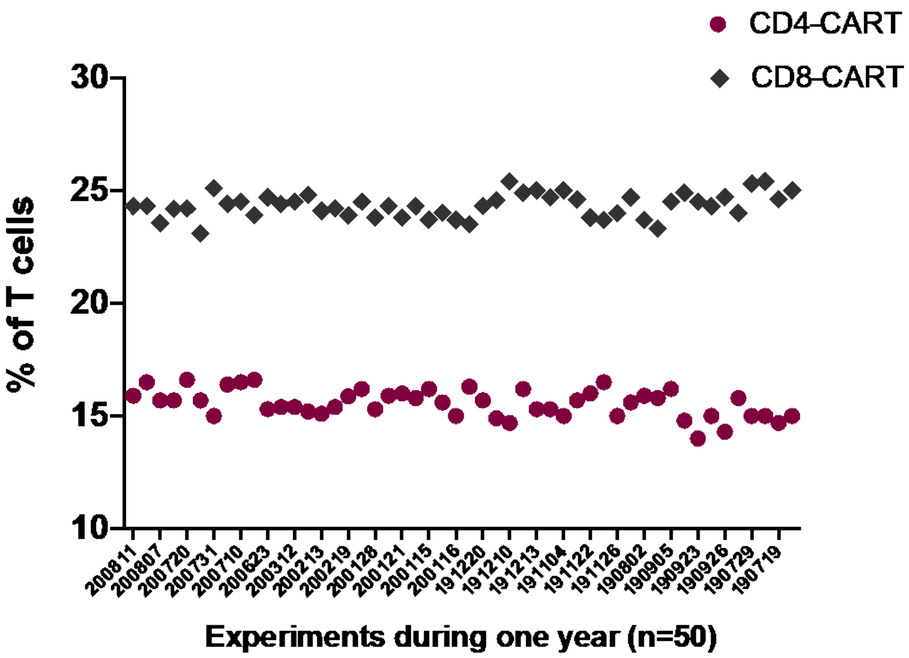

Supplemental Figure 4:CD19-22.BB.z-CAR Gating Strategy

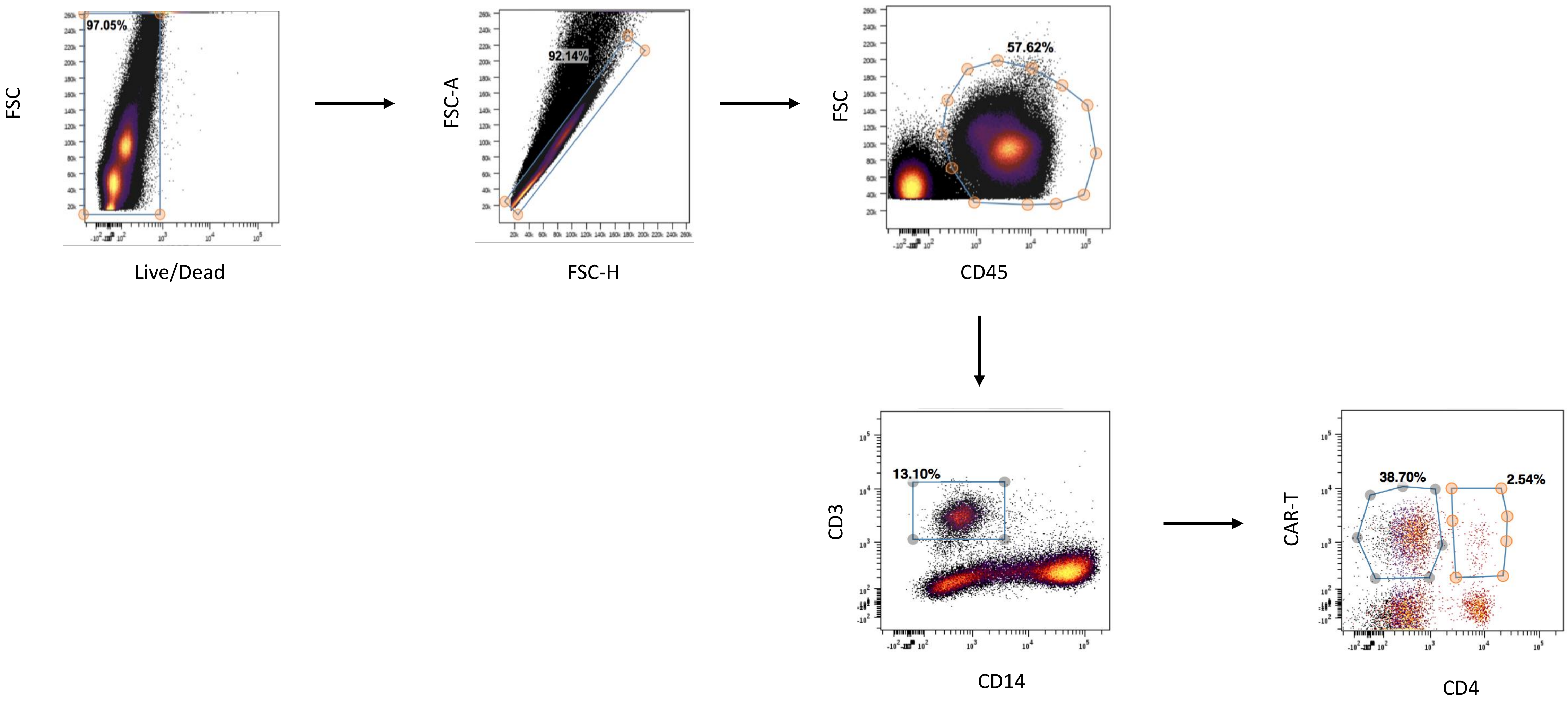

Phase 1 Dose Escalation Study of CD19/CD22 Chimeric Antigen Receptor (CAR) T cells in Adults with Recurrent or Refractory B Cell Malignancies

DEPARTMENT OF MEDICINE-BLOOD & MARROW TRANSPLANTATION

STANFORD CANCER INSTITUTE

Principal Investigator: **David Miklos, MD, PhD**

IND-holder: **Crystal L Mackall, MD**

Study Agent: **Autologous T cells Transduced with bivalent lentiviral vector (CD19/CD22.BB.z) chimeric antigen receptor (CAR); following fludarabine and cyclophosphamide**

Protocol Version: **Amd 4, dated 20 May 2019**

Document History	Notes
Amd 0, Version Date: May 04, 2017	Initial IRB and FDA submission
Amd 0, Version Date: May 05, 2017	Initial FDA submission, with administrative corrections
Amd 0, Version Date: June 15, 2017	Revision in response to FDA comments
Amd 1, Version Date: December 18, 2017	Shortened cell culture duration and added 3 additional subjects to cohort 1, Revision of Correlative Labs Collection Dates and Table. Revision of formatting and structure of protocol. Addition of specific criteria for definition of DLTs. Clarified several eligibility criteria.

Amd 2, Version Date: March 20, 2018	Separate the expansion cohorts to evaluate DCBL and ALL in separate analyses. Revise the particulars for patients with ALL to be consistent with CCT5007 on the same IND. Administrative corrections for consistency throughout the study. Administrative updates in response to DSMC audit. Added stipulation from Safety Monitoring Committee re: wait times between patients in Dose level 1 receiving cells with shortened culture times.
Amd 3, Version Date: January 2, 2019	Adds Miltenyi Biotec Inc (MBI) Sunnyvale Facility as a manufacturing site for the investigational product.
Amd 4, Version Date: March 21, 2019	Updated assessment and grading of CRS and neurotoxicity to ICANS system proposed by ASBMT working group. Define enrollment as date of eligibility confirmation, rather than date of apheresis or manufacture. Updates to eligibility criteria for CD19 expression, performance status, CNS inclusion, prior malignancy exclusion, cardiovascular exclusion and autoimmune exclusion. Timing windows added to study procedures. Remove hematologic toxicity from DLT definition. Revise AE reporting guidelines and windows to capture relevant events. Updates to correlative sample collection calendar.

Confidentiality Statement

This document contains confidential information of Stanford University School of Medicine.
Do not copy or distribute without written permission of the sponsor.

PRINCIPAL INVESTIGATOR'S SIGNATURE PAGE

David Miklos, MD, PhD

Associate Professor of Medicine (Blood and Marrow Transplantation)
Clinical Director Cancer Cell Therapy
Medical Director of Stanford Cellular Therapeutics and Transplantation Laboratory
269 West Campus Dr, CCSR 2205, M/C 5623
Stanford, California, 94305-5623
650-452-8155

Signature **Date**

SPONSOR DETAILS

**Name of IND Sponsor:**	Crystal L. Mackall, M.D.
Address:	265 Campus Dr G3141A, MC5456<br>Stanford, California, 94305
Telephone Number:	1-650-725-9670

STANFORD CO-INVESTIGATORS

Lori Muffly, M.D.<br>300 Pasteur Drive, H0101<br>Stanford, CA 94305<br>T: 650-723-0822<br>F: 650-736-8092<br>lmuffly@stanford.edu	Sally Arai, M.D.<br>300 Pasteur Drive, H0101<br>Stanford, CA 94305<br>T: 650-723-0822<br>F: 650-736-8092<br>sarai1@stanford.edu
Laura Johnston, M.D.<br>300 Pasteur Drive, H0101<br>Stanford, CA 94305<br>T: 650-723-0822<br>F: 650-736-8092<br>korb@stanford.edu	Robert Lowsky, M.D.<br>300 Pasteur Drive, H0101<br>Stanford, CA 94305<br>T: 650-723-0822<br>F: 650-736-8092<br>rlowsky@stanford.edu
Everett Meyer, M.D., Ph.D.<br>300 Pasteur Drive, H0101<br>Stanford, CA 94305<br>T: 650-723-0822<br>F: 650-736-8092<br>evmeyer@stanford.edu	Robert Negrin, MD<br>300 Pasteur Drive, H0101<br>Stanford, CA 94305<br>T: 650-723-0822<br>F: 650-736-8092<br>negrs@stanford.edu
Andrew Rezvani, MD<br>300 Pasteur Drive, H0101<br>Stanford, CA 94305<br>T: 650-723-0822<br>F: 650-736-8092<br>arezvani@stanford.edu	Judith Shizuru, MD, PhD<br>300 Pasteur Drive, H0101<br>Stanford, CA 94305<br>T: 650-723-0822<br>F: 650-736-8092<br>jshizuru@stanford.edu
Wen-Kai, Weng, MD, PhD<br>300 Pasteur Drive, H0101<br>Stanford, CA 94305<br>T: 650-723-0822<br>F: 650-736-8092<br>wkweng@stanford.edu	Praveen Shiraz, MD<br>300 Pasteur Drive, H0115<br>Stanford, CA 94305<br>T: 650-723-0820<br>F: 650-725-8950<br>pshiraz@stanford.edu
Matthew Frank, MD, PhD<br>300 Pasteur Drive, H0101<br>Stanford, CA 94305<br>T: 650-723-0822<br>F: 650-736-8092<br>mjfrank@stanford.edu	
**BIOSTATISTICIAN**<br>John Tamaresis<br>Room X369, MSOB M/C 5464<br>1265 Welch Rd<br>Stanford, CA 94305<br>T: 650-498-6426<br>jtamares@stanford.edu	**STUDY COORDINATORS**<br>Juliana Craig, BA<br>300 Pasteur Drive E-1 anteroom<br>Stanford CA 94305<br>Phone# (650) 736-0912<br>Email: jkcraig@stanford.edu
**Coordinating Center/Participating Sites:**	Single Site

SYNOPSIS

**Protocol Number**	IND#17484/ e-Protocol # IRB-41382 / CCT5001
**Protocol Title**	Phase 1 Dose Escalation Study of Autologous CD19/CD22 Chimeric Antigen Receptor (CAR) T cells in Adults with Recurrent or Refractory B Cell Malignancies
**Sponsor**	Crystal L. Mackall, M.D.
**Protocol Director**	David Miklos, M.D., Ph.D.
**Name of Investigational Product**	Autologous T cells transduced with bivalent lentiviral vector (CD19/CD22.BB.z) Chimeric Antigen Receptor (CD19/CD22 CAR); following Fludarabine and Cyclophosphamide
**Clinical Phase**	Phase 1/1b
**Background and Rationale**	Relapsed/refractory B-cell acute lymphoblastic leukemia (ALL) is a predominantly CD19+/CD22+ hematologic malignancy that is associated with dismal survival in adults. Complete response (CR) to conventional salvage chemotherapy occurs in < 30% and long-term survival following salvage therapy with or without allogeneic stem cell transplant (SCT) has been reported to be < 25% in younger adults and < 10% in adults ≥ 50 years[1],[2], [3].<br><br>Diffuse Large B-cell Lymphoma (DLBCL) is a CD19+/CD22+ non-Hodgkin lymphoma malignancy that represents the most common aggressive B-cell non-Hodgkin lymphoma (NHL) in adults. Subjects who are not cured with up-front rituximab-containing therapy regimens are typically offered salvage therapy followed by autologous SCT. Subjects with chemorefractory disease, or those who relapse within one year of autologous SCT have very poor outcomes with long-term event-free survival (EFS) of < 20%[4].<br><br>CARs are non-native receptors that link an antigen-binding domain to cell signaling domain(s). When expressed in T cells, CARs endow MHC-unrestricted antigen specificity. Most commonly, CARs utilize a single chain variable fragment (scFv) coupled to TCR zeta and CD28 or 4-1BB endodomains. CAR T cells targeting CD19 in subjects with relapsed and refractory B-ALL have demonstrated dramatic activity, with complete responses rates of ≥ 70%[5],[6],[7],[8]. Over 750 subjects have now been treated with CD19-CAR T cells for B cell malignancies. Numerous clinical and scientific insights have been drawn from this emerging experience and serves as the basis for the current approach to CAR therapy.<br><br>In brief, these insights include:<br>• Dramatic and rapid expansion of CD19-CAR T cells is often associated with toxicity, including cytokine release syndrome and neurotoxicity.<br>• CD28 costimulatory facilitates more rapid and higher peak T cell expansion[7],[6] but it also predisposes T cells to early exhaustion, leading to poor long-term T cell persistence from activation induced cell death[9]. 4-1BB costimulatory domains on the other hand are associated with a slower expansion rate, lower peak level, a diminished risk of T cell exhaustion and more prolonged persistence following adoptive transfer[9].<br>• Treatment with CD19-CAR therapy causes prolonged B cell aplasia that is an expected consequence of effective therapy, which can be effectively managed with immunoglobulin replacement therapy.

<table>
<tr><td></td><td>

- CD19 immune escape has been observed by several groups following CD19-CAR therapy for B-ALL[10],[7],[11]. Investigation of this phenomenon reveals a complex biology responsible for loss or downregulation of CD19 expression observed in these cases.

CD22 is a sialic acid binding adhesion molecule, which belongs to the Siglec superfamily. Signaling via CD22 mediates inhibitory signals on B cell receptor signaling via immunoreceptor tyrosine-based inhibitory motifs, which are essential for maintenance of B cell tolerance[12-14]. CD22 expression is restricted to cells of the B-lineage. It is expressed on the surface of mature B cells, but is not expressed on pluripotent hematopoietic stem cells[15],[16]. The vast majority of B-lineage malignancies express CD22 including B-ALL and B cell NHL[17],[18],[19],[20],[21],[22, 23]. Several therapeutics targeting CD22 have been studied in clinical trials with some success and no evidence for on target, off-tissue toxicity has been observed.

The Pediatric Oncology Branch of the National Cancer Institute developed a chimeric antigen receptor targeting CD22. Clinical testing of this first chimeric antigen receptor targeting CD22 is under study in children and young adults with B-ALL. Results from the first 16 subjects enrolled have been encouraging with clinical responses (MRD negative CR) and acceptable toxicities.

Sequential treatment with CD19-CAR followed by CD22-CAR for subjects who develop CD19 neg/dim resistance provides one approach to immune targeting of B-ALL. However there is concern that a similar loss of CD22 expression might also occur as a result of an immune response directed solely toward one target. Furthermore, we noted that in one subject treated with CD22-CAR who developed recurrence at 3 months showed increased expression of CD19 at that time point. Therefore we posit that the long-term effectiveness of CARs could be enhanced if the CAR could simultaneously target two antigens, such as CD19 or CD22.

To this end, we developed a chimeric antigen receptor with transmembrane and signaling domains were essentially identical to the CD22-CAR, but with scFvs identical to those incorporated into the original CD19-CAR (fmc63) and the CD22-CAR described above (m971). This bivalent, bispecific CAR is capable of recognizing cells CD19+CD22+ cells, as well as CD19+CD22– and CD19–CD22+ cells. The receptor has equivalent activity against NALM-6 B-ALL cells in xenografts compared to CD19-CARs and CD22-CARs.

</td></tr>
<tr><td>Objectives</td><td>

Primary Objectives:

✓ Determine the feasibility of producing CD19/CD22-CAR T cells meeting the established release criteria.

✓ Assess the safety of administering escalating doses of autologous CD19/CD22-CAR T cells that meet established release specifications in adults with B-cell hematologic malignancies following a cyclophosphamide/fludarabine conditioning regimen. The following dose escalation will be used:

(a) Dose Level -1: 3×10^5 transduced T cells/kg (± 20%)
(b) Dose Level 1: 1×10^6 transduced T cells/kg (± 20%)
(c) Dose Level 2: 3×10^6 transduced T cells/kg (± 20%)
(d) Dose Level 3: 1×10^7 transduced T cells/kg (± 20%)

Secondary Objectives:

✓ Evaluate the ability of CD19/CD22-CAR T cells to mediate clinical activity in adults with DLBCL and adults with ALL.

Exploratory Analyses:

</td></tr>
</table>

	✓ Evaluate the frequency of CD22+ expression on lymphoma cells, and determine site density when possible.<br>✓ Analyze alterations in early B cell development induced by immune pressure exerted via anti-CD19/CD22-CAR T cells.<br>✓ Evaluate whether subjects receiving CD19/CD22-CAR T cells relapse with loss or diminished expression of CD19 and/or CD22, when feasible.<br>✓ Measure persistence of CD19/CD22-CAR T cells in the blood, bone marrow and CSF, and explore correlations between CD19/CD22-CAR T cell properties and CAR T cell efficacy and persistence.<br>✓ Establish the utility of chromatin structure and epigenomic technology to characterize CAR T cell therapies.
**Primary Endpoint(s)**	The primary endpoints for this study are:<br>- Feasibility defined by the rate of successful manufacture of the CD19/CD22-CAR T cells to satisfy the targeted dose level and meet the required release specifications.<br>-Safety of CD19/CD22-CAR T cells as evidenced by the incidence and severity of dose limiting toxicities (DLT), adverse events, serious adverse events, laboratory abnormalities, changes in vital signs, and changes in physical examination following infusion of CD19/CD22-CAR T cells, recorded and graded according to the Common Terminology Criteria for Adverse Events (CTCAE) Version 4.0 (see link) at three dose levels.
**Secondary and Exploratory Endpoints**	The secondary endpoint for this study is:<br>-Clinical response as measured by Response Criteria for ALL (see **Section 14.5.2**)<br>- Complete Response (CR)<br>- Partial Response (PR)<br>- Hematological Activity<br>- Stable Disease (SD)<br>-Progressive Disease (PD)<br>-The response rate as measured by Response Criteria for Lymphoma (see **Section 14.5.1**):<br>- Complete Response (CR)<br>- Partial Response (PR)<br>- Stable Disease (SD)<br>-Progressive Disease (PD)
**Study Centers**	This is a Stanford University, investigator initiated protocol conducted at Stanford Health Care (SHC).
**Sample Size**	The minimum accrual will be 24 subjects and the maximum accrual will be 90 subjects: Up to 6 subjects will be enrolled in 3 dose cohorts (18 subjects) to determine maximum tolerated dose (MTD), and up to 3 subjects may be replaced in each dose cohort (9 subjects) due to inability to achieve target dose of cells, and up to 6 subjects may be replaced for inevaluable subjects (subjects enrolled who cannot receive cells) for a maximum of 33 subjects. An additional 3 patients will be enrolled in dose cohort #1 to evaluate the safety of CD19/CD22-CAR T cells manufactured with shortened culture duration. An expansion phase will enroll up to a total of 30

	subjects in each of two cohorts: 1) Adults with DLBCL and 2) Adults with ALL (for a total of 60 subjects in both cohorts) may be enrolled at the MTD or highest dose tested (including those treated in dose escalation) to conduct a preliminary assessment of efficacy. Therefore a maximum of 90 subjects (18 + 9 + 6 + 3 + 24 + 30).
**Overall Duration of the Study**	The recruitment period for this study is expected to be 3-4 years. Subjects will be followed for 5 years post treatment. The total duration of this study is expected to be 8-9 years of active treatment and short term follow up, and a total of 15 years of long term follow up after the last subject completes study therapy.
**Duration of Study per Subject**	Subject's active participation in this study is expected to be 5 years, with a total of 15 years of long term follow up from the time of cell infusion.
**Subject Population**	Subjects ≥ 18 years of age, with B-ALL or DLBCL who have relapsed or have refractory disease after standard therapies, who meet the eligibility criteria.
**Eligibility criteria**	✓ CD19 positive expression on malignant cells by immunohistochemistry or flow cytometry<br>✓ Age: ≥ 18 years of age.<br>✓ All subjects must have evaluable or measurable disease; subjects with lymphoma must have evaluable or measurable disease according to the revised IWG Response Criteria for Malignant Lymphoma[24]. Lesions that have been previously irradiated will be considered measurable only if progression has been documented following completion of radiation therapy.<br>✓ Performance status of ECOG 0, 1 or 2 or Karnofsky ≥60%<br>✓ Adequate organ and marrow function as defined by (supportive care is allowed per institutional standards, i.e. filgrastim, transfusion):<br>o ANC ≥ 750/uL*<br>o Platelet count ≥ 50,000/uL*<br>o Absolute lymphocyte count ≥ 150/uL*<br>✓ Adequate renal, hepatic, pulmonary and cardiac function defined as:<br>o SaO2 ≥ 92% on room air<br>o Creatinine ≤ 2 mg/dL or Creatinine Clearance ≥ 60 mL/min (as estimated by Cockcroft Gault)<br>o Total bilirubin ≤ 1.5 mg/dL (except in subjects with Gilbert's disease) (Elevations related to leukemia or lymphoma involvement of the liver will not disqualify a subject)<br>o Serum ALT/AST ≤ 10 times the ULN (institutional normal) (Elevations related to leukemia or lymphoma involvement of the liver will not disqualify a subject)<br>o Cardiac ejection fraction (LVEF) ≥ 45%<br>✓ Subjects with CNS involvement or a history of CNS involvement are eligible only in the absence of neurologic symptoms that may mask or interfere with neurological assessment of toxicity<br>✓ Subjects with history of allogeneic SCT must be at least 100 days from SCT, have no evidence of Graft versus Host Disease (GvHD), and no longer taking immunosuppressive agents for at least 30 days prior to enrollment.<br>✓ Females of child-bearing potential and males of child-fathering potential must be willing to practice birth control during and for 4 months post therapy.<br>✓ Females of child-bearing potential must have negative pregnancy test.<br>✓ Must meet wash-out period since prior therapies.<br>✓ Must have recovered from acute side effects from prior therapy to meet eligibility.<br>✓ If had prior CAR therapy, 30 days must have elapsed prior to apheresis; may not have evidence of persistence of CAR T cells in blood samples (circulating levels

	of genetically modified cells of ≥ 5% by flow cytometry)<br>✓ May NOT have uncontrolled infection. Simple UTI or uncomplicated bacterial pharyngitis is permitted if responding to active treatment.<br>✓ May NOT have ongoing HIV, HBV or HCV infection. History of HBV or HCV is permitted if viral load is undetectable by qPCR and/or nucleic acid testing.<br>✓ May NOT have history of other malignancies, apart from non-melanoma skin cancer or carcinoma in situ, unless disease free for at least 3 years, or in remission 1-2 years and Principal Investigator assesses other malignancy as unlikely to return within 1 year or interfere with CAR T cell safety<br>✓ May NOT be receiving anticoagulation therapy<br>✓ May NOT have active CNS disorder, or autoimmune disease with CNS involvement that in investigator's judgement impair ability to evaluate neurotoxicity.<br>✓ May NOT have history of MI, cardiac angioplasty or stenting, unstable angina or other clinically significant cardiac disease with 12 months of enrollment.<br>✓ May NOT have severe, immediate hypersensitivity reaction attributed to compounds of similar chemical or biologic composition to any agents used in study.<br>✓ May NOT be pregnant or breastfeeding.<br>✓ May NOT have primary immunodeficiency or history of autoimmune disease (e.g. Crohns, rheumatoid arthritis, systemic lupus) requiring systemic immunosuppression/systemic disease modifying agents within the last 2 years.<br>✓ May NOT, in investigator's judgment, have any medical condition likely to interfere with assessment of safety or efficacy, or be unlikely to complete all protocol-required visits and procedures.<br>* A subject will not be excluded because of pancytopenia ≥ Grade 3 if it is felt by the investigator to be due to underlying leukemia/lymphoma.
**Investigational Product, Dose, and Mode of Administration**	Autologous CD19/CD22-CAR T cells in escalating doses administered intravenously after conditioning lymphodepletion chemotherapy regimen with cyclophosphamide and fludarabine<br>Dose escalation:<br>▪ Dose Level -1: 3×10^5 transduced T cells/kg (± 20%)<br>▪ Dose Level 1: 1×10^6 transduced T cells/kg (± 20%)<br>▪ Dose Level 2: 3×10^6 transduced T cells/kg (± 20%)<br>▪ Dose Level 3: 1×10^7 transduced T cells/kg (± 20%)<br>Fludarabine 30 mg/m^2 per day IV for days -5, -4, -3<br>Cyclophosphamide 500 mg/m^2 per day IV for days -5, -4, -3
**Study Design and Methodology**	This is a phase 1 dose escalation study in adult subjects with B-ALL or DLBCL who have relapsed or refractory disease after standard therapies. Autologous PBMC will be obtained by leukapheresis and transduced with CD19/CD22-CAR lentiviral vector. Cryopreserved PBMC stored from participation in other institutional cell therapy or cell collection studies may be used to generate the cellular product on this study as long as they meet the criteria established in this IND.<br>Subjects will receive a conditioning lymphodepletion chemotherapy regimen of fludarabine and cyclophosphamide followed by infusion of CD19/CD22-CAR T cells. Subjects will be evaluated sequentially after treatment for toxicity, antitumor effects and for persistence of CAR in blood samples and functionality of transduced T cells.<br>Three planned dose levels of CD19/CD22-CAR T cells will be administered in escalating cohorts. With Amendment 1 the culture time for generating the CD19/CD22-CAR T cells was shortened to approximately 7 days. To ensure safety, 3 additional subjects will be treated at Dose level 1. If 2/6 subjects experience DLT at dose level 1, dose -1 will be explored (3×10^5 transduced T cells/kg (± 20%)). The

	MTD, or highest cell dose studied if MTD is not reached, will be expanded to further explore the safety, feasibility and clinical response activity.
Statistical Methodology	Feasibility will be defined by the successful manufacturing and expansion of CD19/CD22-CAR T cells to satisfy the targeted dose level and meet the requirements of the COA. If after the first 6 subjects have been enrolled, more than 3 are unable to have adequate CD19/CD22-CAR T cells produced (that meet COA for infusion), accrual to that dose level in the study will be terminated, since the upper 90% one-sided confidence interval about 3/6 is 79.9%; thus, it would be unlikely that the true feasibility rate is 80% or greater, which would be desirable.<br><br>Safety of CD19/CD22-CAR T cells as evidenced by the incidence and severity of dose limiting toxicities (DLT), adverse events, serious adverse events, laboratory abnormalities, changes in vital signs, and changes in physical examination following infusion of CD19/CD22-CAR T cell will be evaluated at three dose levels: 1) 1 x 10^6 transduced T cells/kg (± 20%), 2) 3 x 10^6 transduced T cells/kg (± 20%), and 3) 1 x 10^7 transduced T cells/kg (± 20%).<br><br>Up to 6 evaluable subjects may be enrolled in cohorts 1 to 3 in order to determine a safe and feasible dose. In addition, the study will allow for up to 3 subjects to be replaced in each of the dose cohorts (9 additional subjects) due to inability to achieve target doses. An additional 3 subjects will be enrolled in dose level 1 at the time of approval of Amendment 1; the additional subjects will receive CD19/CD22-CAR T cells manufactured with shortened culture duration. In addition, the study will allow for 6 total inevaluable subjects (subjects enrolled but who cannot receive cells, either due to physical deterioration or withdrawn consent during cell growth).<br><br>After determining the MTD (or highest dose tested if MTD is not achieved), two expansion cohorts (DLCBL and ALL) will each enroll 15 subjects, including subjects treated at the MTD in the dose escalation phase, to further define safety, persistence, efficacy and duration of response. If 5 or fewer subjects have an OR (in subjects with DLBCL) or CR (in subjects with ALL), further enrollment to the study will stop, with the upper 80% Confidence Limit of 0.476 not reaching the 0.48 minimum overall response rate expected (40%), otherwise enrollment will proceed to a maximum of 30 subjects in each group treated at MTD. With 0 DLT out of 30 subjects, based on the one-sided upper Confidence Limits, we can rule out true DLT rates as high as 6%, 8% and 10% with 80%, 90% and 95% confidence (respectively). The precision of estimate of the probability of dichotomous outcomes (as measured by 90% half-widths of two-sided 90% confidence intervals) relating to evidence for efficacy is no worse than 0.17, under the above conditions.<br><br>Thus, 3+3+3+3 +12 yields a minimum of 24 subjects while 18+ 9 + 6 + 30 + 24 yields a maximum of 90 subjects who may be enrolled.

SCHEMA

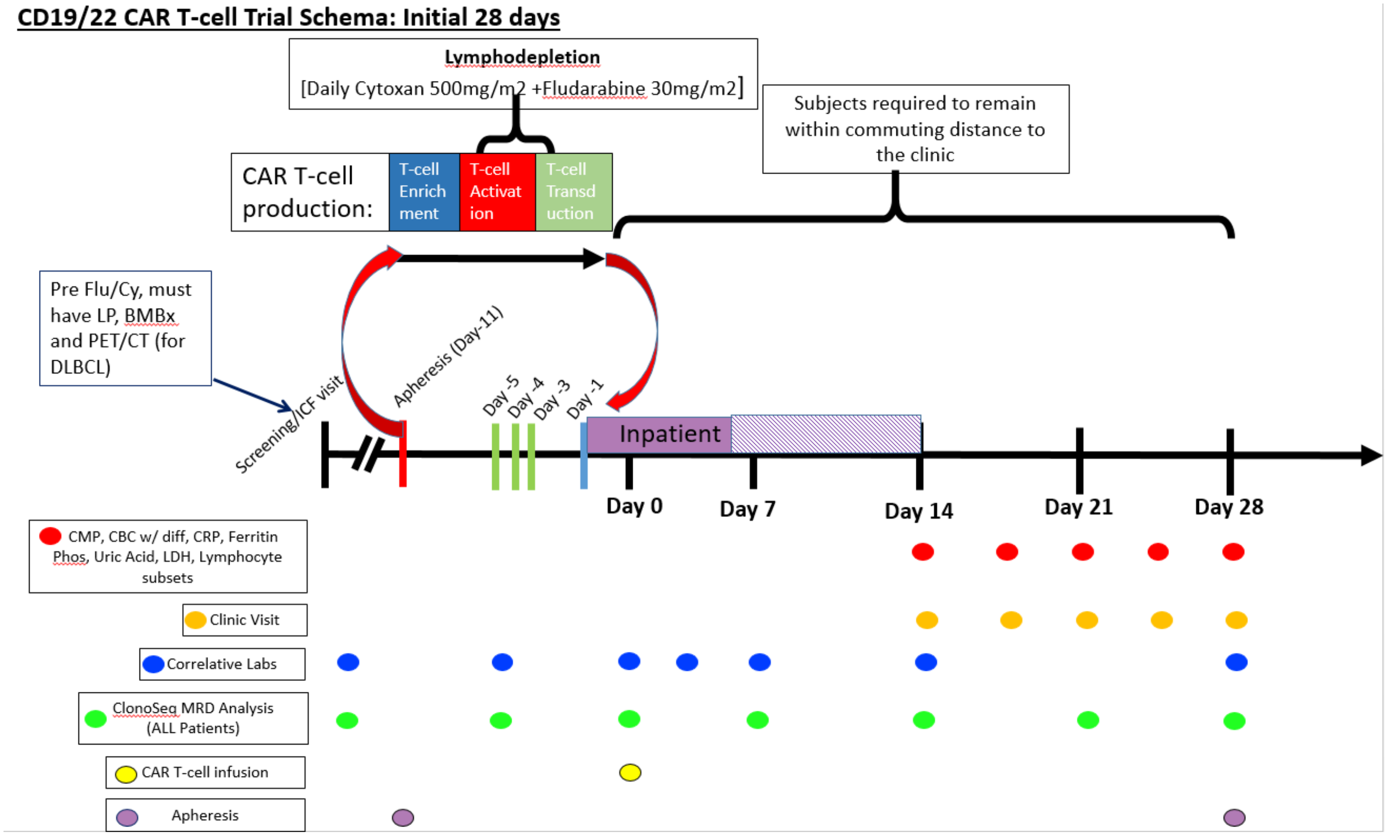
CD19/22 CAR T-cell Trial Schema: Initial 28 days
Lymphodepletion
[Daily Cytoxan 500mg/m2 +Fludarabine 30mg/m2]
Subjects required to remain within commuting distance to the clinic
CAR T-cell production:
T-cell Enrichment
T-cell Activation
T-cell Transduction
Pre Flu/Cy, must have LP, BMBx and PET/CT (for DLBCL)
Screening/ICF visit
Apheresis (Day-11)
Day -5
Day -4
Day -3
Day -1
Inpatient
Day 0
Day 7
Day 14
Day 21
Day 28
CMP, CBC w/ diff, CRP, Ferritin Phos, Uric Acid, LDH, Lymphocyte subsets
Clinic Visit
Correlative Labs
ClonoSeq MRD Analysis (ALL Patients)
CAR T-cell infusion
Apheresis

TABLE OF CONTENTS

PRINCIPAL INVESTIGATOR'S SIGNATURE PAGE 3
SPONSOR DETAILS 4
STANFORD CO-INVESTIGATORS 4
SYNOPSIS 5
SCHEMA 11
TABLE OF CONTENTS 12
TABLE OF FIGURES 15
LIST OF ABBREVIATIONS AND DEFINITION OF TERMS 16
1 OBJECTIVES 18
1.1 PRIMARY OBJECTIVE 18
1.2 SECONDARY OBJECTIVES 18
1.3 EXPLORATORY ANALYSIS 18
2 BACKGROUND AND RATIONALE 18
2.1 B CELL MALIGNANCIES IN ADULTS 18
2.2 CHIMERIC ANTIGEN RECEPTOR (CAR) THERAPY 20
2.3 DEVELOPMENT OF A BI-SPECIFIC CD19/CD22-CAR FOR B-CELL MALIGNANCIES 27
2.4 PRODUCTION OF CD19/CD22 CAR EXPRESSING T CELLS 32
2.5 SAFETY CONSIDERATIONS FOR BISPECIFIC CD19/CD22 CAR THERAPY 34
2.6 CORRELATIVE STUDIES BACKGROUND 37
2.7 PROTOCOL RATIONALE AND SUMMARY 39
2.8 STUDY DESIGN 40
3 PARTICIPANT SELECTION AND ENROLLMENT PROCEDURES 42
3.1 SCREENING PARTICIPANT ELIGIBILITY CHECKLIST 43
3.2 INFORMED CONSENT PROCESS 50
3.3 SUBJECT SCREENING ASSESSMENTS AND REGISTRATION 50
4 TREATMENT PLAN 51
4.1 OVERVIEW 51
4.2 GENERAL CONCOMITANT MEDICATION AND SUPPORTIVE CARE GUIDELINES 51
4.3 CRITERIA FOR REMOVAL FROM PROTOCOL THERAPY AND OFF STUDY CRITERIA 53
5 SUBJECT STUDY PROCEDURES 54
5.1 SUBJECT SCREENING 54

5.2 LEUKAPHERESIS FOR CELL ACQUISITION: 56
5.3 CONDITIONING CHEMOTHERAPY 57
5.4 INVESTIGATIONAL AGENT ADMINISTRATION AND RESEARCH PROCEDURES 59
5.5 EVALUATIONS AND FOLLOW UP 62
5.6 OPTION FOR ADDITIONAL DOSE(S) OF CD19/CD22-CAR T CELLS 69
5.7 PHASE 1 DOSING PROCEDURES 71
6 INVESTIGATIONAL AGENT AND COMMERCIAL DRUG INFORMATION 75
6.1 AUTOLOGOUS T CELLS TRANSDUCED WITH LENTIVIRAL VECTOR (CD19/CD22.BB.Z) CHIMERIC ANTIGEN RECEPTOR (CD19/CD22-CAR); FOLLOWING FLUDARABINE AND CYCLOPHOSPHAMIDE 75
6.2 FLUDARABINE 76
6.3 CYCLOPHOSPHAMIDE 77
6.4 ACETAMINOPHEN (TYLENOL): 78
6.5 DIPHENHYDRAMINE (BENADRYL): 79
6.6 ANTIMICROBIAL PROPHYLAXIS 79
6.7 LEVETIRACETAM (KEPPRA): 79
6.8 ALLOPURINOL 79
7 ADVERSE EVENTS AND REPORTING PROCEDURES 79
7.1 POTENTIAL ADVERSE EVENTS 79
7.2 ADVERSE EVENT DEFINITIONS 80
7.3 ADVERSE EVENT REPORTING 83
7.4 IND SPONSOR REPORTING CRITERIA 83
7.5 FDA REPORTING CRITERIA 85
8 CORRELATIVE/SPECIAL STUDIES 86
8.1 SAMPLE COLLECTION SCHEDULE 87
8.2 BLOOD DRAWING LIMITS FOR RESEARCH PURPOSES 89
9 STUDY CALENDAR 90
10 MEASUREMENTS 93
10.1 PRIMARY OUTCOME MEASURES 93
10.2 SECONDARY OUTCOME MEASURES 93
10.3 EXPLORATORY OBJECTIVES 94
11 REGULATORY CONSIDERATIONS\ 94
11.1 INSTITUTIONAL REVIEW OF PROTOCOL 94

11.2 DATA AND SAFETY MONITORING PLAN 94
11.3 DATA MANAGEMENT PLAN 95
12 COLLABORATIVE AGREEMENTS 95
13 STATISTICAL CONSIDERATIONS 95
13.1 STATISTICAL DESIGN 95
13.2 SAFETY ENDPOINTS 96
13.3 PROTOCOL SAFETY RULES 98
13.4 FEASIBILITY ENDPOINT 98
13.5 EFFICACY ENDPOINT 98
13.6 EXPLORATORY ANALYSIS 100
13.7 SAMPLE SIZE 100
14 APPENDICES 102
14.1 APPENDIX A: PERFORMANCE STATUS CRITERIA 102
14.2 APPENDIX B: GUIDELINES FOR TOXICITY ASSESSMENT AND MANAGEMENT – DOSE EXPANSION COHORT 103
14.3 APPENDIX C: GUIDELINES FOR GRADING AND MANAGEMENT OF SUSPECTED CYTOKINE RELEASE SYNDROME AND NEUROTOXICITY – DOSE ESCALATION COHORT 108
14.4 APPENDIX D: CALCULATION OF WEIGHT FOR CELL DOSE CALCULATION IN MORBIDLY OBESE CANDIDATES 111
14.5 APPENDIX E: RESPONSE CRITERIA 112
14.6 APPENDIX F: MONITORING GENE THERAPY TRIALS: REPLICATION COMPETENT LENTIVIRUS (RCL) 116
14.7 APPENDIX G: DRAFT LETTER AND QUESTIONNAIRE TO SUBJECTS FOR LONG TERM FOLLOW-UP FOR DELAYED ADVERSE EVENTS 117
14.8 APPENDIX H: PHYSICIAN (LOCAL MEDICAL PROVIDER) LETTER 119
14.9 APPENDIX I: CORRELATIVE SAMPLE SCHEDULE 120
15 REFERENCES 121

TABLE OF FIGURES

Figure 1: CD22 Site Density with CD22-CAR T cell Therapy 25

Figure 2: Loop CAR Construction Using CD19 & CD22 scFv from Single CAR Constructs 28

Figure 3: Potent in vivo Activity of Bispecific CAR T cells against CD19+CD22+ Leukemia..... 28

Figure 4: Cytokine production by ELISA in vitro..... 30

Figure 5: IFN-gamma production with CARs 30

Figure 6: Relapsed Subject Sample Cured in Xenograft Model After Treatment with Bispecific Loop CAR T cells...... 31

Figure 7: Transduction Efficiencies of CAR Strategy Options..... 31

Figure 8: Effectiveness of CAR Therapy Administration Options 32

LIST OF ABBREVIATIONS AND DEFINITION OF TERMS

ADL	Activities of daily living
AE	Adverse event
ALL	Acute lymphoblastic leukemia
ASBMT	American Society for Blood and Marrow Transplantation
BID	Twice daily
BMT-CTF	Stanford Bone Marrow Transplant – Cellular Therapeutics Facility
BSA	Body surface area
CAR	Chimeric Antigen Receptor
CBC	Complete blood count
CI	Confidence interval
CMAX	Maximum concentration of drug
CMV	cytomegalovirus
CNS	Central nervous system
CRF	Case report/Record form
CR	Complete response
CRS	Cytokine release syndrome
CSF	cerebral spinal fluid
CTCAE	Common Terminology Criteria for Adverse Events
DLBCL	Diffuse large B cell lymphoma
DLCO	Diffusing capacity of the lungs for carbon monoxide
DLT	Dose Limiting Toxicity
DMSO	Dimethyl Sulfoxide
DSMB	Data Safety Monitoring Board
EBV	Epstein-Barr virus
ECG	Electrocardiogram
ELISA	enzyme-linked immunosorbent assay
FL	Follicular lymphoma
GI	Gastrointestinal
GMP	Good Manufacturing Practices
GvHD	Graft versus Host Disease
HbsAG	Hepatitis B surface antigen
Hgb	Hemoglobin
HIV	Human Immunodeficiency Virus
HLH	Hemophagocytic lymphohistiocytosis
HPF	High-power field
HSV	herpes simplex virus
HTN	Hypertensions
ICANS	Immune effector cell-associated neurotoxicity syndrome
ICE	Immune Effector Cell-associated encephalopathy
IDB	ideal body weight
IRB	Institutional Review Board
IV	Intravenous

LCGM	Stanford's Laboratory for Cell and Gene Medicine
LLN	Lower limit of normal
MAS	macrophage activation syndrome
MBI	Miltenyi Biotec Inc.
MRD	Minimal residual disease
MRI	Magnetic resonance imaging
MTD	Maximum tolerated dose
MUGA scan	multigated acquisition scan
OR	Overall response
OS	Overall survival
PBMC	Peripheral blood mononuclear cells
PCR	polymerase chain reaction
PD	Progressive diseased
PFS	Progression free survival
PFTs	pulmonary function tests
PLT	Platelet
PR	Partial response
QD	Once daily
RECIST	Response evaluation criteria in solid tumors
RR	Response rate
SAE	Serious adverse event
SCT	stem cell transplant
SD	Stable disease
TCR	T-cell Receptor
TLS	Tumor lysis syndrome
TTP	Time to progression
ULN	Upper limit of normal
UNK	Unknown
VZV	varicella zoster virus
WBC	White blood cell
WHO	World Health Organization

1 OBJECTIVES

1.1 Primary Objective

- ✓ Determine the feasibility of producing CD19/CD22-CAR T cells meeting the established release criteria.
- ✓ Assess the safety of administering escalating doses of autologous CD19/CD22-CAR T cells that meet established release specifications in adults with hematologic malignancies following a cyclophosphamide/fludarabine conditioning regimen.

1.2 Secondary Objectives

- ✓ Evaluate the ability of CD19/CD22-CAR T cells to mediate clinical activity in adults with DLBCL and/or adults with ALL.

1.3 Exploratory Analysis

- ✓ Evaluate the frequency of CD22+ expression on lymphoma cells, and determine site density when possible.
- ✓ Analyze alterations in early B cell development induced by immune pressure exerted via CD19/CD22-CAR T cells.
- ✓ Evaluate whether subjects receiving CD19/CD22-CAR T cells relapse with loss or diminished expression of CD19 and/or CD22, when feasible.
- ✓ Measure persistence of CD19/CD22-CAR T cells in the blood, bone marrow and CSF, and explore correlations between anti-CD19/CD22-CAR T cell properties and correlations between immune responses directed toward the CD19/22-CAR T cells and CAR T cell efficacy and persistence.
- ✓ Establish the utility of chromatin structure and epigenomic technology to characterize CAR T cell therapies.

2 BACKGROUND AND RATIONALE

2.1 B Cell Malignancies in Adults

2.1.1 Acute Lymphoblastic leukemia (ALL) in Adults

The incidence of ALL follows a bimodal distribution with the first peak occurring in early childhood, and a second occurring in older adults. Although survival rates for pediatric ALL have consistently improved over the last 20 years with most recent estimates of long-term disease free survival of 90% in children with B-cell ALL[25] survival in adult ALL has remained flat at 30-40%[26],[27]. Outcomes for adults with relapsed/refractory B-cell have been particularly poor. For these subjects, CR to conventional salvage chemotherapy occurs in < 30% and disease-term survival following salvage therapy with or without allogeneic SCT has been reported to be < 25% in younger adults and < 10% in adults ≥ 50 years[1],[2],[28],[3]. Further, toxicity associated with chemotherapy and SCT are substantial, and adult subjects who relapse following SCT rarely experience long-term survival[29].

Therapeutic advances in adult B-cell ALL have been achieved in recent years. Notably,

the development and now approval of the bi-specific anti-CD19/CD3 T-cell engaging (BITE) antibody blinatumomab has resulted in complete response rates in subjects with relapsed and refractory B-cell ALL of 50-80%[30],[31]; however, the responses are often not durable and many subjects relapse without consolidative allogeneic stem cell transplant. Further, approximately 20% of subjects treated with BITE therapy will experience CD19 escape, relapsing with CD19 negative leukemia[32]. Additional agents are thus needed in order to improve the outcomes of adults with B-cell ALL.

2.1.2 Diffuse Large B-cell Lymphoma (DLBCL) in Adults

Diffuse Large B-Cell Lymphoma (DLBCL) is the most common lymphoid malignancy in the United States and Europe, accounting for approximately 30-40% of all non-Hodgkin lymphoma (NHL)[33],[34]. There are approximately 25,000 new cases of DLBCL in the US each year[35]. DLBCL is a heterogeneous and despite recent advancements in combination immunotherapy and chemotherapy regimens, event-free survival (EFS) beyond 5 years only occurs in 30% to 50% of subjects.

Therapeutic advances have been achieved in standard first-line treatment of DLBCL with the addition of the anti-CD20 monoclonal antibody rituximab to cyclophosphamide, doxorubicin, vincristine, and prednisone (R-CHOP)[36], [37]. Despite this, about one-third of subjects with DLBCL do not achieve durable remission and develop primary relapsed/refractory disease with an overall survival rate measured in months[38],[39]. Most relapses of DLBCL occur within the first 3 years, but some still occur after 5 years of remission[40].

Subjects with primary refractory DLBCL represent the highest proportion of subjects in need of new therapies. The current approach to primary relapsed/refractory DLBCL is salvage chemotherapy followed by high dose platinum based therapy (HDT) and autologous stem cell transplantation (ASCT)[41]. Unfortunately, half of subjects with primary relapsed/refractory disease are not eligible for HDT-ASCT right from the start because of age (typically older than 70-75 years), comorbidities, or lack of adequate social support to help with the care needed after transplantation [42]. Of the remaining 50% initially eligible, an additional 50% do not receive HDT-ASCT mainly because their disease shows no sensitivity to salvage chemotherapy[4]. Thus, only approximately 10% of those with relapsed DLBCL will be cured with HDT followed by ASCT[39].

The large SCHOLAR-1 retrospective study highlights the poor outcomes across all subgroups of chemorefractory DLBCL. For these subjects, expected median overall survival (OS) is only 6-7 months, with the lowest OS rates observed in subjects with refractory to second or later line therapy and those who relapsed less than 12 months after an ASCT. In aggregate, response to therapy in refractory DLBCL was 26%, with a CR rate of only 8%[43].

Similarly, the recently reported REFINE collaborative retrospective study analyzed the outcomes of subjects with primary refractory DLBCL. They reported a response rate (CR + PR) of approximately 40% to salvage therapy. For the subgroup undergoing ASCT, the 2-year OS rate was only 54.9%, with the subgroup with germinal center B type DLBCL with positive c-myc expression (by FISH) having a far worse OS rate of 23.7%[44].

Taking into account the observations reported from experience with ASCT, the results of the SCHOLAR-1 study and the REFINE collaborative study, it can be surmised that the overall outlook for subjects with primary relapsed/refractory DLBCL is dismal. A large portion are unable to undergo further aggressive therapy with HDT and ASCT and for even those who are able to pursue such an approach the outcomes are disappointing. Thus, there is a pressing need to develop and incorporate novel therapeutic agents into the treatment of subjects with primary refractory/relapsed DLBCL.

2.2 Chimeric Antigen Receptor (CAR) Therapy

CARs are non-native receptors that link an antigen-binding domain to cell signaling domain(s). When expressed in T cells, they endow MHC-unrestricted antigen specificity. Most commonly, CARs utilize a single chain variable fragment (scFv) coupled to TCR zeta and CD28 or 4-1BB endodomains. Beginning in 2010, several groups published clinical results using CAR T cells targeting CD19 for B cell malignancies.

2.2.1 CD19 CAR Therapy

CD19 is a 95 kD transmembrane protein with expression restricted to the B cell lineage. Expression of CD19 is present from the time of immunoglobulin rearrangement through B cell development and maturation until it is lost with terminal plasma cell differentiation[45]. It is not expressed on primitive hematopoietic stem cells. CAR T cells targeting CD19 first demonstrated impressive clinical efficacy in small case series in adults with B cell lymphomas and chronic lymphocytic leukemia [46-48]. The first pediatric report followed shortly after and demonstrated striking responses in two children with refractory and relapsed B-ALL[10]. The demonstration of exquisite sensitivity of acute B cell leukemias inspired multiple centers to advance translation of CD19 targeting CARs. Despite utilizing varying strategies, clinical trials from multiple institutions have consistently shown dramatic activity of CD19-CAR T cells in subjects with relapsed and refractory B-ALL with complete responses rates of ≥ 70% across trials[5],[6],[7],[8]. Kochenderfer et al.[8] demonstrated CAR efficacy in aggressive lymphomas and reported 4 out of 7 complete responses in adults with refractory DLBCL treated with anti-CD19 CAR T cells.

2.2.1.1 CD19-CAR T cell dose level

Over 750 subjects have now been treated with CD19-CAR T cells for B cell malignancies. As detailed below, numerous clinical and scientific insights have been drawn from this emerging experience and serve as the basis for the current novel approach to CAR therapy. Responses following CD19-CAR therapy for B-ALL occur rapidly, with maximal response observed within 28 days following infusion of the engineered T cells. The relationship between CAR T cell dose and efficacy is less direct than that of a non-replicating drug, however there does appear to be a threshold dose of CAR T cells required for efficacy. In the phase 1 study of anti-CD19 CAR therapy by Lee et al, the dose level of 1×10^6 cells/kg was found to be safe and effective, but the higher dose level of 3×10^6 cells/kg while effective, was found to exceed the maximum tolerated dose[7]. Similarly, the work by Kochenderfer et al., the $1\text{-}2 \times 10^6$ cells/kg dose has been similarly safely and effectively employed, while higher doses have been associated with toxicity[8].

In contrast, the lower starting cell dose of 3 x 10^5 cells/kg tested in the ongoing phase 1 study of anti-CD22 CAR T cells at the NCI (PI-Fry) was largely ineffective in the majority of subjects treated (1 responder amongst 6), while the 1 x 10^6 cells/kg dose was, as in the anti-CD19 CAR experience, both safe and effective, with the higher cell dose of 3 x 10^6 cells/kg associated with potentially more severe toxicity (Personal Communication—Shah/Fry). In most active clinical studies, the recommended dose of CD19-CAR T cells is 1 x 10^6 cells/kg. The inclusion of endogenous costimulation within CAR constructs permits lower dosing than that required to achieve efficacy when using adoptive T cell methods lacking co-stimulatory signals.

2.2.1.1 CD19-CAR T cell efficacy

Independent of the infused T cell dose, anti-leukemic effects generally do not occur in the absence of expansion of the CD19-CAR T cells *in vivo*. Dramatic CD19-CAR T cell expansion can be observed, with > 1000-fold expansion occurring within 1-2 weeks in subjects with high tumor burden. CAR expansion can be quantified using polymerase chain reaction to measure the number of cells with integrated virus or by flow cytometry, where is it not uncommon to observe that > 70% of all circulating T cell transiently express the engineered CD19-CAR receptor.

Expansion of CAR T cells is antigen driven, but is greatly enhanced following treatment with a lymphodepleting preparative regimen. Extensive preclinical work has demonstrated that lymphopenia induces elevations in the availability of cytokines that drive T cell expansion, notably IL-7 and IL-15, and that increased availability of such factors lead to enhanced expansion and improved efficacy[49]. Based upon this paradigm, the vast majority of clinical trials using CAR T cell incorporate a pre-infusion preparative regimen, comprising cyclophosphamide and fludarabine, agents known to induce profound lymphocyte depletion.

2.2.2 CD19-CAR T cell Therapy Associated Toxicities

2.2.2.1 Cytokine release syndrome (CRS)

Dramatic and rapid expansion of CD19-CAR T cells is often associated with toxicity, including cytokine release syndrome and neurotoxicity. Cytokine release syndrome (CRS) has been the topic of several reports and reviews[5],[50]. In brief, CRS comprises a febrile, sepsis-like picture that results from hemodynamic and organ effects of supraphysiologic levels of inflammatory cytokines produced directly or indirectly by the activated T cells. Among the most important of these cytokines are IL-6 and IFN-gamma[7],[6],[5]. CRS can be safely managed with supportive care and in some cases, immunosuppression using anti-IL6R mAbs therapy and corticosteroids[50]. Notably, CRS is limited in subjects with low tumor burdens, as the CAR T cell expansion appears to be largely driven by the magnitude of the leukemia burden with contributions from any existing normal B cell population, and CRS severity correlates with the degree of CAR T cell expansion.

2.2.2.2 Neurotoxicity

Neurotoxicity is also observed in a significant minority (20-30% in most series) of subjects[7],[6],[5] with toxicity ranging from mild to severe. The pathobiology of

CD19-CAR associated neurotoxicity is not fully understood, but the clinical syndrome is associated with increased expansion of CD19-CAR T cells and identification of CD19-CAR T cells in the cerebrospinal fluid. Neurotoxicity may be seen more frequently in subjects with CNS leukemia[7], however subjects without documented CNS leukemia or lymphoma can also develop symptoms of neurotoxicity that range from mild to severe. Hence, any direct association of CNS disease burden to neurotoxicity severity is unclear[7] The syndrome typically manifests clinically as confusion, aphasia and/or dysmetria, and occasionally seizures. Radiographic changes are typically not observed and the syndrome typically resolves in 1-2 weeks and appears fully reversible. The prevailing hypothesis regarding the pathophysiology of this syndrome is that it reflects non-specific neurotoxic effects of cytokines and/or activated T cells, rather than a direct on-target effect of CD19-CAR T cells. Indeed, the dose limiting toxicity of IL-6 when administered in a Phase 1 trial was neurotoxicity and transient aphasia was observed[51]. In further support of this hypothesis, the Jensen laboratory at Seattle Children's Hospital has recently developed a rhesus model of neurotoxicity that utilizes a CD20-CAR rather than the CD19-CAR platform and the model appears to model the human syndrome well (ASH presentation, unpublished). This provides further evidence against a direct, on-target effect involving CD19 in brain tissue.

In the summer of 2016, three deaths were reported as a result of severe neurotoxicity in subjects treated with CD19-CAR T cells. Given that more than 750 subjects have been treated with these therapeutics, and only one previous death was reported and attributed to neurotoxicity associated with status epilepticus, these events were unexpected and work is underway to understand the basis for this phenomena. Subsequently, two additional deaths were noted in the same trial utilizing high dose cyclophosphamide (in the absence of fludarabine) and CD19-CAR T cells incorporating a CD28 costimulatory domain. Notably, CAR T cells that incorporate a 4-1BB costimulatory domain do not typically undergo as rapid expansion as those using a CD28 costimulatory domain, so the risk of fatal or very severe neurotoxicity may be reduced with a 4-1BB containing CAR.

Cerebral edema is another potential cause of death associated with CD19-CAR T cells. In our own experience at Stanford, we had one case of fatal cerebral edema associated with CD19-CAR T cells incorporating a CD28 costimulatory domain in April 2017. Additionally, with respect to 4-1BB costimulation, a fatality occurred in February 2017 (personal communication - Seattle) in which a patient died from cerebral edema associated neurotoxicity using a CAR incorporating the 4-1BB costimulatory domain. The CAR being utilized in this study will incorporate a 4-1BB costimulatory domain.

2.2.2.3 Co-stimulatory Signaling

Another major insight gleaned from the substantial clinical experience with CD19-CAR T cells for B cell malignancies is the differential impact of distinct costimulatory domains on T cell function. It has become evident that CD28 costimulation facilitates more rapid and higher peak T cell expansion[6, 7], it also predisposes T cells to early exhaustion, which leads to poor long-term T cell persistence as a result of activation induced cell death[9]. In contrast, 4-1BB costimulation is associated with a slower expansion rate, lower peak level, a diminished risk of T cell exhaustion and more prolonged persistence following adoptive transfer[9]. Although remission induction rates do not appear to differ

between CD28 and 4-1BB containing CD19-CARs, sustained remission likely requires T cell persistence and thus 4-1BB CARs may confer improved long-term outcomes.

2.2.2.4 B-Cell Aplasia

The cumulative CD19-CAR experience affirms that prolonged B cell aplasia is an expected consequence of effective, persistent CD19-CAR therapy. Similar to the management of subjects with congenital absence of B cells, and subjects treated with chronic rituximab, CAR-mediated B cell aplasia can be managed effectively with immunoglobulin replacement therapy[52],[53]. Thus far, significant increase in infection susceptibility or other toxicity related to chronic B cell depletion has not been noted[6].

2.2.3 CD19 Immune Escape Following CD19 Directed Immunotherapy

CD19 is universally expressed at high levels on B-ALL at diagnosis and levels of expression do not change appreciably following cytotoxic therapy. Indeed, CD19-based flow cytometry provides powerful prognostications by measuring minimal residual disease (MRD) following standard cytotoxic therapy in B-ALL[54],[55],[56]. However, with the introduction of CD19-based immunotherapies, relapse with diminished or absent surface CD19 has been increasingly observed. CD19 immune escape was first reported following blinatumumab therapy[57], and has been observed by several groups following CD19-CAR therapy for B-ALL[10],[7, 11]. In a recent report from Children’s Hospital of Philadelphia of 50 subjects rendered into remission with CD19-CAR therapy, 40% of subjects had relapsed with a median follow-up of 10.5 months, and loss of the CD19 target accounted for 65% of the total relapses[58]. While the true incidence of relapse due to CD19 immune escape has not yet been established, our CD19-CAR experience to date implicated CD19 escape as the most common cause of post-CAR relapse in B-ALL.

Investigation into the biology of CD19 immune escape has identified a complex biology guiding the loss or downregulation of CD19 expression. The two distinct patterns of tumor remodeling that have been recognized are categorized as the “isoform switch” and “lineage switch”. The majority of cases fall into the isoform switch category, wherein the cells retain all other characteristics of B-ALL and there is no clear evidence for alteration in fitness. In the majority of “isoform switch” cases, mRNA specific for CD19 is retained, but is enriched for CD19 isoforms that preferentially remain intracellular, lack a transmembrane domain, and/or lack the epitopes targeted by all CD19-CARs currently under study as well as blinatumumab[59]. Such B-ALL cells resultantly express reduced total surface levels of wild-type CD19 or express an aberrant CD19 that lack the epitope targeted by CD19-CARs and blinatumumab. An alternative pathway for loss or diminution of CD19 expression involves tumor cells undergoing switching from the lymphoid to the myeloid lineage with a resultant population with myeloid markers and features that clonally relates to the parental B cell leukemia[11].

2.2.4 CD22-CAR Therapy for B-ALL

The emergence of CD19 immune escape has prompted interest in the development of immunotherapies targeting alternative B-ALL cell surface molecules. CD22 is a 130 kD sialic acid binding transmembrane glycoprotein, which belongs to the Siglec superfamily.

Signaling via CD22 mediates inhibitory signals on B cell receptor signaling via immunoreceptor tyrosine-based inhibitory motifs, which are essential for maintenance of B cell tolerance[12],[13],[14]. CD22 expression is restricted to cells of the B-lineage. It is expressed on the surface of mature B cells, but is not expressed on pluripotent hematopoietic stem cells[15],[16]. The vast majority of B-lineage malignancies express CD22 including B-ALL and B cell NHL[17],[18],[19],[20],[21],[22],[23].

Several therapeutics targeting CD22 have been studied in clinical trials with some success and no evidence for on target, off-tissue toxicity. An unconjugated anti-CD22 mAb (Eptratuzumab), mediated modest clinical activity against B-ALL alone or when combined with chemotherapy in single arm studies[60],[61],[62]. CD22 is rapidly internalized following antigen binding, leading to development of CD22 antibody drug conjugates and immunotoxins. Inotuzumab, an anti-CD22 mAb conjugated to calicheamicin, mediated complete responses in 81% of subjects with relapsed/refractory B-ALL[63]. Moxetumumab, an anti-CD22 genetically linked to pseudomonas exotoxin mediated clinical activity in hairy cell leukemia[64] and B-ALL[65], but benefits were transient due to the agent's short half-life, and substantial immunogenicity, which precluded repetitive cycles. ^{90}Y-conjugated epratuzumab mediated complete responses in 3 of 17 B-ALL subjects treated, but this was associated with pancytopenia due to radiotoxicity[66].

The Pediatric Oncology Branch of the National Cancer Institute (POB NCI) sought to develop a chimeric antigen receptor targeting CD22. A variety of binding domains, hinge regions and costimulatory domains were compared for efficacy based upon killing of cell lines *in vitro* and the capacity to eradicate human B-ALL xenografts in immunodeficient mice. The lead candidate incorporates the fully human scFv m971, which recognizes a membrane proximal epitope on CD22[67],[19]. Among other therapeutics targeting CD22 currently or previously in clinical trials, none have utilized this binding domain. The lead candidate also has a very short hinge region, and incorporates the TCR zeta signaling domain and the 4-1BB endodomain, which was selected based upon superior functionality compared to the CD28 endodomain[9].

Clinical testing of this first chimeric antigen receptor targeting CD22 is under study at the POB NCI in children and young adults with B-ALL. Results from the first sixteen subjects enrolled are encouraging. The first dose level tested of 3 x 10^5/kg was chosen to be lower than the current standard CD19-CAR dosing because this was a first in human study. Six subjects received this dose with one experiencing an MRD negative CR. A second subject demonstrated signs of clinical activity as evidenced by a substantial reduction in circulating blasts, but the subject subsequently developed rapid disease progression and did not achieve a CR. At the second dose level, 1 x 10^6/kg, eight of ten subjects for whom results are available thus far experienced an MRD negative CR. Each of the remissions induced at the 1 x 10^6 dose level were associated with persistent CD22-CAR T cells in the peripheral blood. One of the subjects from this initial cohort remains in an ongoing remission at 18 months post CAR therapy with ongoing detection of CAR T cells. At dose level three, 3 x 10^6/kg, the first subject developed Grade 4 hypoxia following CAR T cell infusion. A second subject was safely treated at this dose level without a DLT. Based on the single DLT at the third dose level and clinical activity appreciated at dose level 2 (1 x 10^6 cells/kg), a decision was made to expand dose level 2 to further explore activity

and safety. Eight additional subjects have since been treated at dose level 2, 1 x 10^6/kg. Nine subjects (56%) achieved a complete remission, with 8 out of 10 responders (80%) receiving dose levels ≥ 1 x 10^6 CD22 CAR/kg. Of the 16 subjects enrolled, 9 had CD19 neg/dim B-ALL as a result of previous CD19 directed immunotherapy, and 6 of these subjects achieved complete remission following CD22-CAR.

From this small series, the investigators conclude that the CD22-CAR shows many characteristics that are similar to the CD19-CAR. Response occurs rapidly within 28 days and correlates with CD22-CAR expansion. Cytokine release syndrome did occur, but the maximal severity was Grade 2. The CD22-CAR T cells traffic to the spinal fluid at levels similar to that observed with CD19-CAR. Although a cyclophosphamide/fludarabine based preparative regimen was used, neurotoxicity was limited to Grade 1 and observed in only 2 subjects. No evidence of unexpected toxicity was seen. As anticipated, since the CD22-CAR incorporates a 4-1BB costimulatory domain, subjects maintained CAR persistence and persistent B cell aplasia at 90 days, with some evidence for persistent CAR cells out to 9 and 18 months seen in those subjects who are in the longest remission. This is the first data to demonstrate that a chimeric antigen receptor targeting a molecule other than CD19 can also mediate potent antileukemic activity. This CD22 CAR is the first alternative to CD19 for CAR based immunotherapy for B-ALL. Given that CD22 is expressed on numerous other B cell malignancies, the results raise the prospect that this CD22-CAR may also provide a novel therapeutic for application across the spectrum of B cell malignancies.

The CD22-CAR also provides an option for salvage immunotherapy, in the event of relapsed disease or resistance to front line immunotherapy.

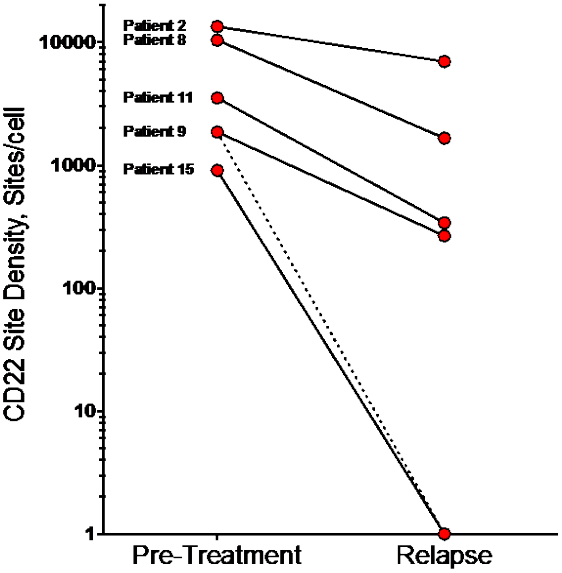

Figure 1: CD22 Site Density with CD22-CAR T cell Therapy

2.2.5 CD22 Immune escape following CD22 targeted immunotherapy

In the current CD22-CAR T cell trial being conducted by POB NCI, alterations in CD22 site density have been observed in a few of the relapsed subjects. One notable example is being seen, following CD22 directed antibody therapy (inotuzumab), where several subjects have emerged with partial loss of CD22 on leukemia blasts after this therapy. Similarly, following anti-CD22 CAR therapy, one subject, at the 6-month restaging evaluation revealed a decrease in CD22 site density from over 10,000 sites/cell prior to CD22-CAR therapy, to approximately 1000 sites/cell at relapse in the presence of circulating CD22 CAR T-cells (see ***Figure 1*.** Subject #8). Another subject, known to be CD19 negative at the time of CD22-CAR infusion, developed relapsed leukemia at 5 months post-CD22 CAR therapy that was partially positive for both CD19 and CD22 (see ***Figure 1*.** Subject #9). Another subject who was known to be CD19 negative prior

to CD22 CAR therapy and had received inotuzumab prior to receiving her CD22 CAR T cells, had a bimodal CD22 population noted in her peripheral blood flow with 89% of blasts expressing CD22; bone marrow flow cytometry showed complete CD22+ population at that time. Despite this, at 1-month post CD22 CAR therapy, she had a complete MRD negative remission, with 41% circulating CD22 CAR T cells in her bone marrow. At her 2-month restaging visit, despite a high level of circulating CD22 CAR T cells, she relapsed with CD19-/CD22- ALL (see ***Figure 1*** Subject #15). Additionally, another subject who was previously refractory to blinatumomab and CD19 CAR, attained a complete remission but had a decrease in CD22 site density, from approximately 3,500 sites per cell prior to CD22 CAR therapy to less than 500 sites/cell at 1 month post infusion (see ***Figure 1.*** Subject #11). The mechanism for CD22 surface expression alteration is unknown at this time, however constant CAR immune pressure as seen in CD19-CAR treated subjects may contribute to this phenomena.

In summary, the reported clinical trial experience using CAR-T cells for management of B cell malignancies and the toxicity profile, supports use of this treatment strategy for children and young adults with no alternative curative options, or has declined to pursue alternative therapy. We believe the lower CRS and neurologic toxicity rates observed using 4-1BB costimulation along with evidence that 4-1BB provides persistent CAR-T expression with durable responses supports the use of 4-1BB costimulation in our CAR19/CD22 construct.

2.2.6 CD19-CAR T cell Therapy for Diffuse large B cell lymphoma (DLBCL)

A number of recent trials illustrate the promise of targeting CD19 in DLBCL. Kite Pharma reported Phase 2 interim analysis with an overall response rate of 76% (47% CR rate) with a majority of responses happening within the first month[68]. Furthermore the side effect profile was tolerable with 29% of subjects experiencing neurologic events and 20% experiencing cytokine release syndrome[68]. Similarly the Penn group recently presented their own experience with CD19 CAR T cell therapy in relapsed/refractory DLBCL, including double hit lymphoma and transformed lymphoma (n=13). At 3 months they reported an ORR of 53% (38% CR rate) with median progression free survival of 5.8 months. Furthermore, at median follow up of 23.3 months for those subjects with a response, 85.7% maintained a response. With regards to safety, 69% experienced cytokine release syndrome, 15% experienced transient neurotoxicity and 7.5% experienced a cognitive disturbance[69]. These recent trial results highlight that CD19 targeted CAR T cell therapy can be effective and safe in subjects with relapsed/refractory DLBCL lymphoma.

In addition to the chemotherapy relapsed/refractory subjects described above, subjects who relapse after hematopoietic stem cell transplantation have also been treated with CD19 CAR T cells. The NCI group led by Jim Kochenderfer, reported using allogenic CD19 CAR T cells in subjects with aggressive B-cell lymphomas (including 5 subjects with DLBCL) who experienced disease progression following an allogeneic stem cell transplant (n=20). They reported an overall event-free survival (EFS) of 39% at 6 months and in the DLBCL subgroup, one subject had a CR which lasted greater than 6 months, 3 had stable disease and 1 had progression of disease. They also reported that there was no increase in the rate of GVHD or worsening in the degree of pre-existing GVHD in the

study participants[70]. A series of studies looking at the use of CD19 central memory enriched CAR T cells following autologous stem cell transplant has been primarily reported by City of Hope and the Fred Hutchinson Cancer Research Center. The first trial (using CD19 devoid of a costimulatory domain) enrolled 7 subjects with DLBCL and one with MCL; 5 of 8 (63%) achieved CR or preserved previously achieved CR status (4 of the DLBCL subjects and 1 MCL subject), and 50% were progression free at 1-yr and 2-yrs of follow up. In the second trial (using CD19 in addition to a CD28 costimulatory domain), 8 subjects (4 DLBCL, 4 MCL); all achieved or maintained previously attained CR status and 75% where progression free at 1-yr follow up. The group did not appreciate any increase in rates of toxicities or adverse impact on transplant course or engraftment outcomes due to the CAR T cell therapy[71].

It is widely believed that antitumor efficacy of CAR-T cells requires efficient CAR-T expansion and persistence. An additional hypothesized attribute of CAR-T efficacy may depend upon the specific T-cell subpopulations that express CD19 CAR. Wang et al. showed that CAR-T cell products containing both $CD4^+$ and CD8+ enriched T cells had higher rates of expansion in DLBCL subjects compared to cell products containing only $CD8^+$ enriched T cells. Six out of 8 subjects received $CD4^+/CD8^+$ enriched T cells were progression free at 1 year compared to 4 out of 8 subjects who received $CD8^+$ enriched cells. Turtle et al., showed similar responses in DLBCL subjects who were infused with CAR-T cell products consisting of a 1:1 ratio of $CD4^+/CD8^+$ T cells. The CR rate in subjects treated with a 1:1 ratio of $CD4^+/CD8^+$ T cells was 64% (82% ORR, $n = 11$)[72]. As already described in pediatric ALL studies, we hypothesize that CAR-T persistence relates to the specific costimulatory domain utilized. For instance, studies in mice have shown that CAR-T cells incorporating the 4-1BB domain reduces CAR-T cell exhaustion whereas the CD28 domain augments exhaustion[9]. In human studies of DLBCL, the KITE pharma and NCI group have used CD28 costimulation whereas the Juno, Fred Hutchinson and UPENN groups have primarily incorporated 4-1BB costimulation domains. In a recent ASH abstract from MGH, Investigator Dr. Abramson reported early results of subjects with relapsed/refractory DLBCL receiving CD19 CAR-T cells utilizing the 4-1BB domain. They reported an ORR of 89% with 73% CR, 9% PR and 18% PD approximately 29 days after CAR T cell infusion. In this study, 21% experienced low-grade cytokine release syndrome, 7% experienced encephalopathy and 7% experienced seizures[73]. The MGH investigators believe the lower CRS and neurotoxicity they observed was due to less rapid CAR-T proliferation using 4-1BB costimulation in contrast to CD28 costimulation KITE pharma utilizes.

In summary, the reported clinical trial experience using CAR-T cells for management of non-Hodgkin lymphoma (NHL) is less than the B cell acute lymphoblastic leukemia (ALL) experience, but CD19-CAR -T therapy shows overall response rates in subjects with chemotherapy/relapsed DLBCL and manageable toxicities. We believe the lower CRS and neurologic toxicity rates observed using 4-1BB costimulation along with evidence that 4-1BB provides persistent CAR-T expression with durable responses supports the use of 4-1BB costimulation in our CAR19/CD22 construct.

2.3 Development of a Bi-specific CD19/CD22-CAR for B-cell Malignancies

Sequential treatment with CD19-CAR followed by CD22-CAR reserved for subjects who

develop CD19 neg/dim resistance provides one approach to immune targeting of B-ALL. However there is concern that a similar loss of CD22 expression might also occur as a result of an immune response directed solely toward one target. Furthermore, one subject treated with CD22-CAR developed recurrence at 3 months with increased expression of CD19 at that time point. Therefore we posit that the long-term effectiveness of CARs could be enhanced if the CAR could simultaneously target two antigens, such as CD19 or CD22.

To this end, we developed a chimeric antigen receptor with transmembrane and signaling domains that are essentially identical to the CD22-CAR, but in place of an scFv targeting singular CD22, we incorporate scFvs identical to those incorporated into the original CD19-CAR (fmc63) and the CD22-CAR described above (m971)(***Figure 2***).

Figure 2: Loop CAR Construction Using CD19 & CD22 scFv from Single CAR Constructs

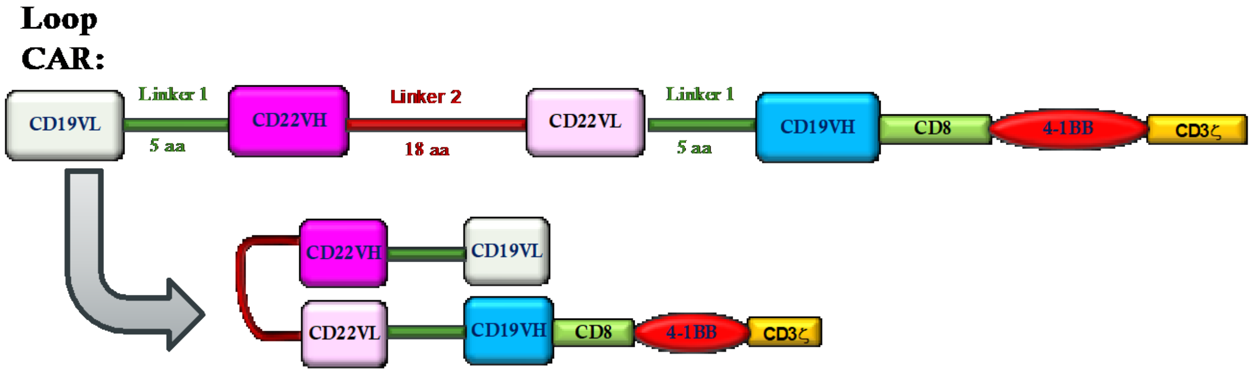

The anti-leukemia activity of bispecific loop CAR T cells was established and compared to CARs targeting the singular CD19 and CD22 antigens. Lentiviral vectors containing supernatants were generated the CD19, CD22, and bispecific CAR constructs and T cells were transduced to express respective CARs. Negative controls using mock (non-transduced) T cells were cultured and activated in parallel. We then tested *in vivo* CAR cytotoxicity. NSG immunodeficient mice were injected with the NALM6 leukemia cell line that has been permanently transfected to express the luciferase gene (termed NALM6-GL, gift of S. Grupp and D. Barret, Children's Hospital of Pennsylvania). NALM6-GL leukemia growth within a xenograft model can be detected by imaging the mice with a sensitive CCD camera that can measure photons produced by the luciferase enzyme following injection of the mice with the substrate, luciferin substrate. On day 3, upon establishment of detectable leukemia, mice were injected with CAR T cells transduced with either CD19, CD22, bispecific loop CAR, or control. The bispecific loop CAR had comparable activity in xenografts compared to CD19-CARs and CD22-CARs (***Figure 3***). This bivalent, bispecific CAR has the advantage of being able to recognize CD19+CD22+ cells, as well as CD19+CD22– and CD19–CD22+ cells.

Figure 3**: Potent *in vivo* Activity of Bispecific CAR T cells against CD19+CD22+ Leukemia**

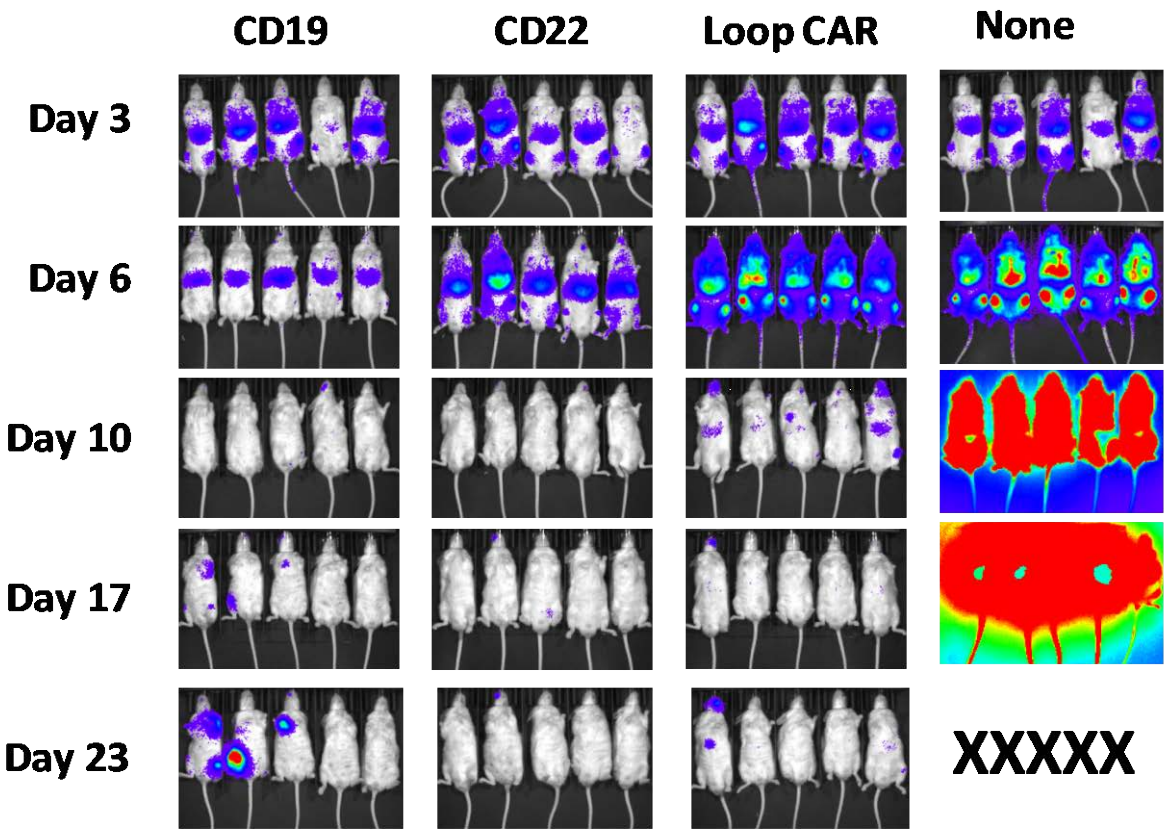

Figure 3: NSG (immunodeficient) mice were injected with 1 x 10^6 NALM6-GL cells on day 0 and images taken on day 3 demonstrate, with luciferin substrate, the presence of leukemia in all animals. On day 3, each mouse group was injected with 3 x 10^6 CAR T cells transduced with either CD19, CD22, Bispecific, or control. Mice were re-imaged weekly and equivalent anti-leukemic activity was seen in Bispecific Loop CAR.

CML cell line K562 was artificially transduced with CD19 or CD22 or both to express the target antigens. K562 cells served as the negative control. 1E5 CAR T cells were washed 3 times and then co-incubated with 1E5 target cells in RPMI media at 37^0C. After 14 hours, culture supernatant was harvested and the production of the cytokines was measured with ELISA kits. IL-2 production by the Loop CAR was comparable to that seen with the CD19 CAR (***Figure 4***).

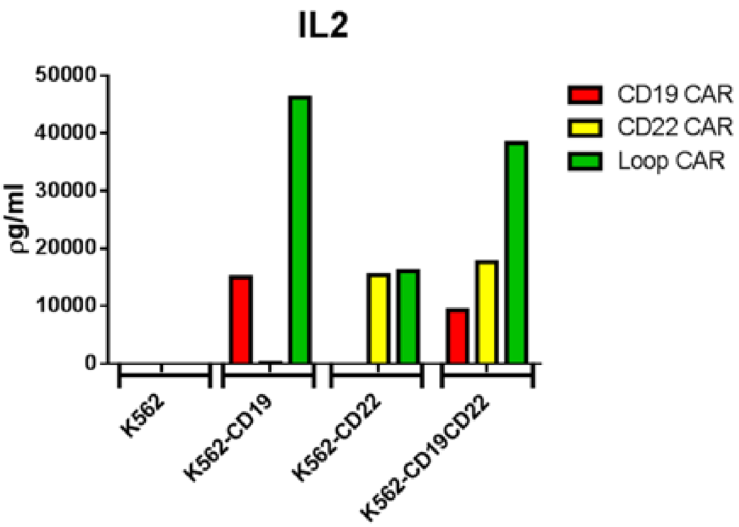

Figure 4: Cytokine production by ELISA *in vitro*

Interferon-gamma production was also seen with use of the bispecific CAR against both CD19 negative and CD22 negative samples (***Figure 5***).

Figure 5: IFN-gamma production with CARs

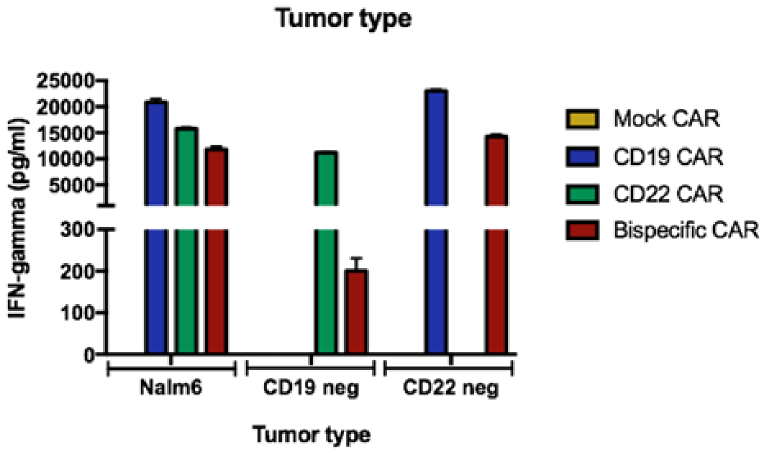

Additional testing was performed pre-clinically to determine if the bispecific loop CAR had activity against CD19 dim populations. After treatment with blinatumomab, a subject relapsed with a $CD19^{low}$ and $CD19^{high}$ leukemia. A subject derived xenograft model (PDX) was created with this leukemia phenotype. At day 0, 1 x 10^6 leukemia cells where injected into two NSG mouse groups, experiment vs control. At day 9, when leukemia was detected by flow cytometry, bispecific loop CAR T cells and non-transduced mock T cells were injected into the experimental and control groups respectively. The bispecific loop CAR demonstrated excellent activity and cured this post CD19 directed immunotherapy relapse.

Subject samples post CD19-directed immunotherapy shows $CD19^{low}$ and $CD19^{high}$ population in addition to CD22 population. NSG mice were injected with 1 x 10^6 leukemia

cells and treated on day 9 with 3 x 10^6 CAR T cells of bispecific loop CAR or non-transduced T cells. The bispecific loop CAR was able to eradicate PDX sample and mice remained disease free up to 49 days (***Figure 6***).

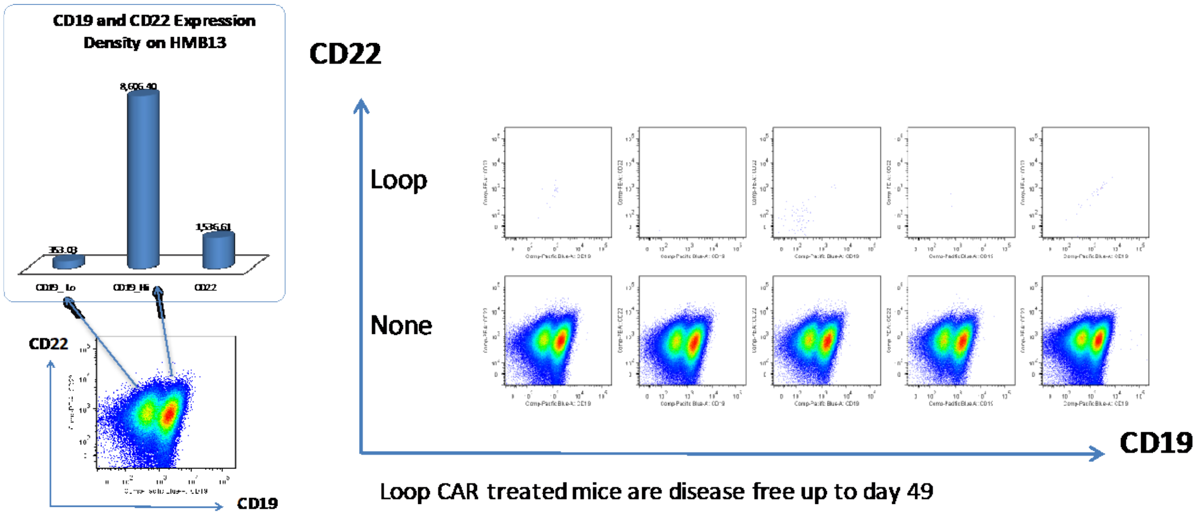

Figure 6: Relapsed Subject Sample Cured in Xenograft Model After Treatment with Bispecific Loop CAR T cells.

Other strategies that could provide a dual-targeting strategy include either 1. Co-transduction of both CARs into one T cell or 2. Co-infusion strategy by which both anti-CD19 CAR and anti-CD22 CAR T cells would be simultaneously infused. Both were considered as alternative approaches to the currently proposed bi-specific CAR strategy. In regards to the first strategy of co-transduction, although a reasonable concept, preclinical studies performed in the Fry lab (POB, NCI) demonstrate that this approach leads to a much lower transduction efficiency than desired and is not a clinically feasible approach at this time. (***Figure 7***, Personal communication-Fry lab)

Figure 7: Transduction Efficiencies of CAR Strategy Options

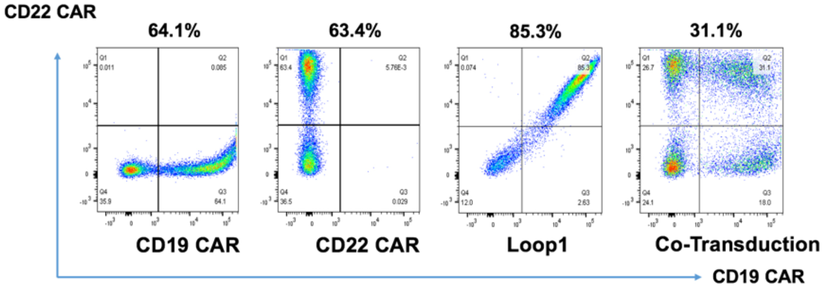

Figure 7: The vectors for CD19, CD22, and the bispecific Loop1 CAR were produced by transient transfection of the 293T lenti packaging cell line. Human PBMCs from a healthy donor were activated with CD3/CD28 microbeads for 24 hours. Activated T cells were then transduced with the vector individually or co-transduced with both CD19 and CD22 vectors together. Surface expression of the CD19 CAR and CD22 CAR were analyzed on day 8. Co-transduced T cells have much lower expression of both CD19 and CD22 CARs compared to the bispecific CAR. The expression of CD19 and CD22 CARs on co-transduced T cells are not at an equal molar ratio. In contrast, Loop1 CAR has an almost 1:1 ratio in expression of CD19 and CD22 CAR, which [illegible]

In regards to a co-infusion model (with either simultaneous or sequential infusion of anti-CD19 and anti-CD22 directed CAR cells), several concerns emerge. First, a sequential infusion approach, which may be preferred to avoid cumulative infusion related toxicity, is ineffective *in vivo*, as demonstrated below (***Figure 8***, Personal Communication-Fry lab).

Figure 8: Effectiveness of CAR Therapy Administration Options

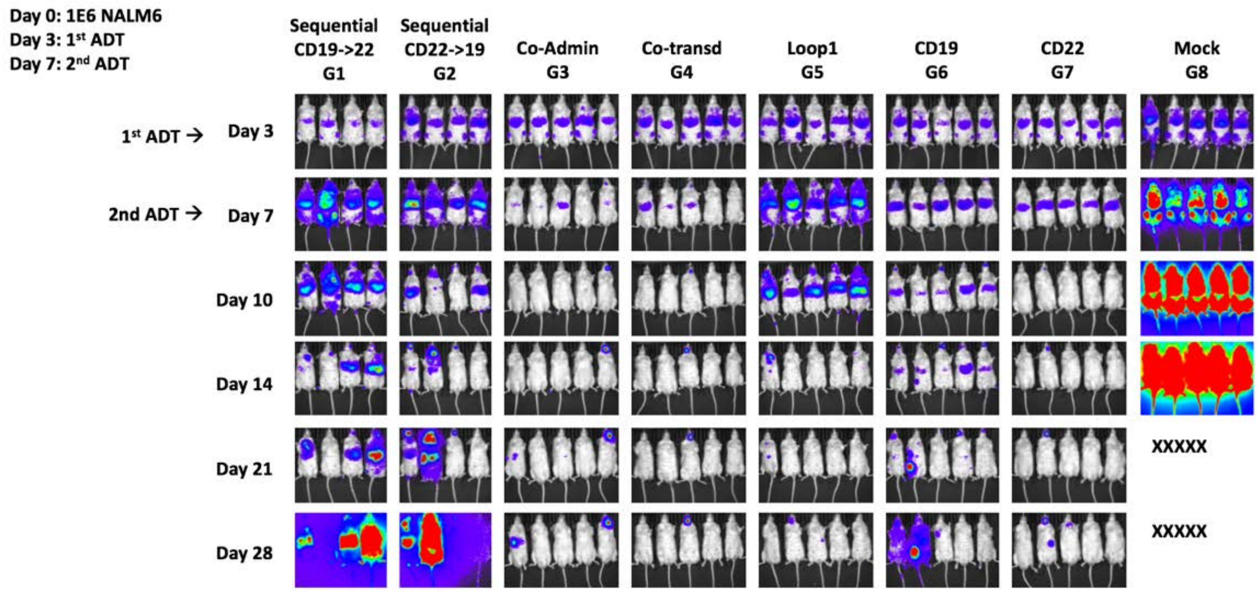

Figure 8: NSG mice were challenged with 1E6 of NALM6 leukemia on day 0. On 3 day, mice in group 4 to 7 received 3E6 of CAR+ T cells. Mice in group 1 and group 2 received sequential treatment with 1E6 CD19 or CD22 CAR+ T cells on day 3 and 3E6 CAR+ T cells of the other CAR on day 7. Mice in group 3 received co-administration of a total of 6E6 CAR T cells with 3E6 of CD19 CAR and 3E6 of CD22 CARs on day 3. Mice in group 4 received almost 10E6 of total T cells due to the low expression on the co-transduced T cells. Bioluminescent intensity represent tumor burden.

Simultaneous administration, while effective, in this model, may have implications in the clinical setting with cumulative toxicity of two different CAR cell expansions—but certainly could be explored further. The co-transduction model, shown above, while effective is notably being given at much higher doses to achieve this efficacy and is not yet a clinically feasible strategy.

Thus in proceeding forward with a dual-targeted approach, to explore the safety of a single CAR with dual targeting capabilities avoids concern for cumulative toxicity and demonstrates *in vivo* clinical activity.

2.4 Production of CD19/CD22 CAR Expressing T cells

This protocol will test the safety and activity of the bi-specific CD19/CD22-CAR T cells in subjects with relapsed, refractory B cell malignancies. An Investigational New Drug application (IND) #17484 has been approved by the U.S. Food and Drug Administration (FDA) for administration of the bi-specific CD19/CD22-CAR. Major elements of the

treatment regimen, including the processing of the cells, the lymphodepleting preparative regimen and dose escalation scheme will closely mimic that clinical development of CD19-CAR and CD22-CARs. Briefly, peripheral blood mononuclear cells collected via apheresis will be enriched for T cells using a CD4/CD8 immunomagnetic bead enrichment (Miltenyi). They will then be activated by co-culture with immunomagnetic particles expressing anti-CD3 and anti-CD28 mAb either at Stanford's Laboratory for Cell and Gene Medicine (LCGM) or at Miltenyi Biotec Inc.'s (MBI) Sunnyvale Facility. One day following activation, replication incompetent lentiviral vector particles containing the CD19/CD22.BB.z construct will be added to the culture for transduction. Cells will be incubated for approximately 9 days with IL-2 for the first 3 subjects, and then for approximately 7 days for subsequent subjects, then harvested and administered fresh or frozen for subsequent infusion. Cells will be required to meet standard release criteria including transduction efficiency ≥ 10%, T cell content ≥ 70%, sterility and minimum levels of LPS as well as no evidence for replication competent lentivirus. All procedures will take place using good manufacturing process guidelines in 3 facilities LCGM, or MBI Sunnyvale Facility and Stanford Bone Marrow Transplant – Cellular Therapeutics Facility (BMT-CTF).

2.4.1 Culture Timing of Cellular Products

2.4.2 Culture Timing of Cellular Products

Manufacturing of CAR T cells for the first three patients treated according to IND **#17484 (**Autologous T-cells transduced with bivalent lentiviral vector (CD19/CD22.BB.z) chimeric antigen receptor (CAR); following fludarabine and cyclophosphamide) has been successful with all three products meeting the release criteria set forth in the IND and clinical protocols. Two adults and one child have been treated with these products at Dose level 1 (1×10^6 CD19/22-CAR T cells/kg) without any dose limiting toxicity.

The protocol allows harvest on Day 9, which can be extended to Day 13 if needed to obtain adequate cell numbers. In each case, the protocol directed dose was met at Day 9, and we noted that cell numbers greatly exceeded the specified dose level. Furthermore, analysis on Day 5 during the culture period demonstrated that adequate dose was already available by Day 5 for all three cases. Therefore, the current culture conditions are generating substantially greater numbers than needed to meet the current and planned clinical trial dose levels.

Culture duration has been studied in the context of adoptive cellular immunotherapy using tumor infiltrating lymphocytes, where conclusions have consistently demonstrated that shorter culture generate T cells that have characteristics that predict greater potency[74], [75] and clinical trials of tumor infiltrating lymphocytes generated with "short" culture durations have demonstrated no evidence for inferior activity or increased toxicity[76], [77]. With regard to CAR T cells, Ghassemi and colleagues compared efficacy of CD19.BB.z-CAR T cells cultured at 3 vs 9-days and observed that those administrated after 3-days of culture demonstrated increased in vivo as measured using dose titrations in a murine model[78]. Therefore, available evidence demonstrates that shortening the culture duration of CAR T cells will not adversely affect their function.

We propose therefore to shorten the culture duration of the CD19/22-CAR T cells

generated according to IND #17484 from 9 days to 7 days. Because of the possibility that shortened culture duration CAR T cells could result in increased potency and therefore increased toxicity, the current dose level which has already been demonstrated to be safe will be utilized for three additional patients treated on the adult protocol and three additional patients treated on the pediatric protocol (1 x 10^6/kg) to ensure that any potential increased toxicity is not confounded by a simultaneous dose escalation. A 14 day window will be incorporated between patients following cell administration. Following enrollment of three additional patients using the modified manufacturing schema at dose level 1 (1 x 10^6/kg), dose escalation will proceed as originally described in the protocol.

In addition, in order to address challenges related to the time involved in completing release testing for a same day fresh infusion, where possible, the 7d culture CD19/22-CAR T cells will undergo cryopreservation on Day 7 and release assays will be completed on Day 7 and Day 8. For patients whose clinical condition mandates infusion on Day 9, samples will be thawed and infused on Day 9 according to the original manufacturing and infusion timeline. For patients whose clinical condition allows greater delay, we will wait for the results of the 14 day culture results prior to infusion.

2.5 SAFETY CONSIDERATIONS FOR BISPECIFIC CD19/CD22 CAR THERAPY

2.5.1 Risk of chemotherapy:

Toxicities resulting from fludarabine and cyclophosphamide in the doses proposed in the current study are well known and are what have been used in the prior anti-CD19 CAR and anti-CD22 CAR protocols conducted at the Pediatric Oncology Branch (POB) of the NCI. Such a preparative regimen is designed to decrease the number of endogenous T cells, including T regulatory cells that may otherwise suppress anti-CAR T cell cytotoxicity, and to induce increased availability of homeostatic cytokines thereby allowing for better engraftment of the transferred CAR T cells. The dose limiting toxicity for both fludarabine and cyclophosphamide is myelosuppression, however myelosuppressive effects are expected to be transient using the doses proposed. Other toxicities including fever, nausea, vomiting, stomatitis, diarrhea, anorexia, edema, skin rashes, myalgias, headache, agitation, and fatigue should be easily managed with appropriate supportive care. Sterile hemorrhagic cystitis occurs in about 20% of subjects who receive cyclophosphamide, but is unlikely given the relatively low dose administered in this trial and given that continuous intravenous infusion of Normal saline will be used prophylactically as a uroprotective agent. Tumor lysis syndrome (TLS) following fludarabine and cyclophosphamide administration can occur in subjects with advanced bulky disease. To prevent this, subjects who are at high risk of TLS, at the investigator's discretion, will be prescribed allopurinol and appropriately hydrated with close monitoring, by uric acid testing, for the development of TLS. Finally, opportunistic infections (protozoan, viral, fungal, and bacterial) have been observed post-fludarabine and cyclophosphamide, especially in heavily pre-treated individuals. Subjects will receive appropriate antimicrobial prophylaxis (e.g., Bactrim for PCP and acyclovir for HSV and VZV prophylaxis) during treatment and for a minimum of 6 months following treatment.

2.5.2 Risk of Autoimmunity:

Autoimmune toxicity is a risk of adoptive cell therapy trials for cancer and can occur if the transferred populations recognize the target antigen on normal tissues. Thus far, on-target, off-tumor toxicity of both CD19-CAR and CD22-CAR T cells has been restricted to B cell aplasia, which can be managed with immunoglobulin replacement therapy. Given that the CD19/CD22-CAR T cells tested in this study incorporates scFvs that have already been tested in clinical trials, it is unlikely that unexpected autoimmune toxicity will occur, but subjects will be monitored closely for the occurrence of unexpected toxicity and if it is observed, appropriate supportive care and modification to the clinical trial will be instituted.

2.5.3 Risks of Gene Therapy:

Risks of gene therapy include insertional mutagenesis or emergence of replication-competent lentivirus (RCL). While insertional mutagenesis is theoretically possible using retroviral vectors, this has only been observed in the setting of infants treated for X-SCID using retroviral vector-mediated gene transfer into CD34+ bone marrow stem cells. In the case of retroviral or lentiviral vector-mediated gene transfer into mature T-cells, there has been no evidence of long-term toxicities associated with these procedures since the first NCI sponsored gene transfer study in 1989. Despite the fact that clinical data currently available suggest that the introduction of lentiviral vectors transduced into mature T-cells is a safe procedure, continued follow-up of all gene therapy subjects will be performed as required. The proposed protocol follows all current FDA guidelines regarding testing and follow up of subjects receiving gene transduced cells. Similarly, the viral vectors used have been engineered to minimize the risk of emergence of replication competent lentivirus, but subjects will be monitored according to Recombinant DNA Advisory Committee guidelines for several years following receipt of this therapy and in the case of the development of second malignancy, all efforts will be made to determine whether replication competent lentivirus has emerged.

2.5.4 Risk of Cytokine Release Syndrome:

Cytokine release syndrome occurs in the setting of a variety of immunotherapies including following antibody infusions and also following adoptive cell therapy. The clinical picture, now described by many groups, closely resembles infection/sepsis, with fever being the hallmark sign. The pathophysiology is related to excess cytokine levels, released either directly from the adoptively transferred cells or indirectly induced by the immune activation. Severe cytokine release syndrome can be associated with signs and symptoms of hemophagocytic lymphohistiocytosis and macrophage activation syndrome (HLH-MAS).

Grading and management of CRS for subjects in dose escalation cohort will follow the guidelines set forth by Lee et al[7] (see **Section 14.3, Appendix C**), which includes diligent supportive care and search for infection, with immunosuppression using anti-IL6R mAbs and/or corticosteroids reserved for more severe cases. Using such a risk adapted system for management of CRS has diminished morbidity and mortality associated with this syndrome.

Recently forty-nine experts from experts in immune effector cell therapies, including CAR T therapy met at a meeting supported by the American Society for Blood and Marrow Transplantation (ASBMT) and reached consensus on the grading definitions for immune effector cell-associated CRS [85] contained in **Section 14.2**, **Appendix B**.

2.5.5 Risk of Neurotoxicity

2.5.5.1 Immune Effector Cell-Associated Neurotoxicity Syndrome (ICANS)

Neurotoxicity is also observed in a significant minority (20-30% in most series) of subjects[7],[5],[6] with toxicity ranging from mild to severe. Symptoms tend to be more diverse than observed with CRS[85]. The pathobiology of CD19-CAR associated neurotoxicity is not fully understood, but the clinical syndrome is associated with increased expansion of CAR T cells and identification of CAR T cells in the cerebrospinal fluid. Neurotoxicity may be seen more frequently in subjects with CNS leukemia[7], however subjects without documented CNS leukemia or lymphoma can also develop symptoms of neurotoxicity that range from mild to severe. Hence, no direct association between CNS disease burden and neurotoxicity severity has been observed in the context of CD19-CAR therapies for leukemia[7]. The syndrome typically manifests clinically as tremors, dysgraphia, impaired attention, confusion, aphasia and/or dysmetria, and occasionally seizures. Expressive aphasia appears to be a characteristic feature. Radiographic changes are variable and the syndrome typically resolves in 1-2 weeks and appears fully reversible in the vast majority of patients. The prevailing hypothesis regarding the pathophysiology of this syndrome is that it reflects non-specific neurotoxic effects of cytokines and/or activated T cells, rather than a direct on-target effect of CAR T cells. Indeed, the dose limiting toxicity of IL-6 when administered in a Phase 1 trial was neurotoxicity and transient aphasia was observed[51]. Lethal neurotoxicity has occurred in <1% of patients treated with CD19-CAR T cells and is associated with cerebral edema and herniation in patients with severe cytokine release syndrome.

In summary, the ASBMT consensus group convened in 2017 defined ICANS as “a disorder characterized by a pathologic process involving the central nervous system following any immune therapy that results in the activation or engagement of endogenous or infused T cell and/or other immune effector cells. Symptoms or signs can be progressive and may include aphasia, altered level of consciousness, impairment of cognitive skills, motor weakness, seizure and cerebral edema”[85].

While immunosuppression appears to effectively reverse cytokine release syndrome, treatment with anti-IL6R for neurotoxicity does not appear to be effective and may even lead to transient worsening due to elevations in circulating IL-6 induced by the mAb. Corticosteroids may have some beneficial effects, but controlled trials have not been undertaken and therefore the true effectiveness of corticosteroids for management of severe neurotoxicity remains unclear.

Furthermore, as described in section **2.2.2**, we postulate that the risk of fatal or very severe neurotoxicity may be reduced with a 4-1BB based on the experience thus far that suggests that 4-1BB costimulatory domain based CARS do not typically undergo as rapid expansion as those using a CD28 costimulatory domain. The CAR utilized in this study will incorporate a 4-1BB costimulatory domain.

Subjects will be monitored carefully for neurotoxicity throughout this study. Additionally, we will also have prospective neurologic evaluations performed on this protocol to provide additional input and insight into the treatment and management of the subjects who do develop neurotoxicity. All subjects will be treated prophylactically with levetiracetam (Keppra), unless contraindicated, and subjects with significant neurotoxicity will be monitored closely and treated aggressively as outlined in section **4.2.7**.

2.5.6 Risk of Cerebral Edema

Cerebral edema is a condition where there is an excessive amount of fluid build-up in the inner and/or outer spaces of the brain, interfering with its function. In its mildest form, symptoms of cerebral edema include nausea, vomiting, blurred vision, and faintness. However, in severe cases people can experience seizures, coma, permanent brain damage, respiratory arrest and death. Cerebral edema is a medical emergency, managed in the ICU and therapies may include: diuretics, specially formulated salt solutions, steroids to reduce inflammation or surgery. Thus far, only a handful of CAR-T patients have experienced sever cerebral edema

2.6 Correlative Studies Background

CAR T cell therapy targeting a single tumor associated antigen has mediated striking remissions in B cell leukemia and lymphoma. The clinical experience to date has demonstrated that anti-leukemic effects are associated with CAR T cell expansion[7],[79] and that sustained remission is associated with CAR T cell persistence[73]. In subjects who have relapsed in the presence of persistent singular CD19 specific or CD22 specific CAR T cells, relapse has often been characterized by CD19 dim/negative or CD22 dim/negative phenotypes respectively. The complex interplay of tumor, T cell and intrinsic CAR properties that influence these outcomes are not well understood. We aim to utilize this safety study as an opportunity to collect correlative data that will permit extensive study of both the B cell and T cell compartment prior to and following CAR T cell therapy. We aim to integrate multi-dimensional technologies to permit complex analyses of the apheresis product, the CAR T cell product pre-infusion and *in vivo* expanded CAR T cells following antigen encounter. We additionally aim to investigate properties of B cell tumors that render them susceptible to CAR T cell cytotoxicity and study physiologic and malignant B cell remodeling under the pressure of multi-targeted CAR therapy.

2.6.1 Factors Impacting Loss of CAR T cells

Persistence of CAR T cells is associated with superior anti-leukemia effects in the setting of CD19-CAR T cell therapy for leukemia. We hypothesize that there are three distinct causes of T cell loss following CD19/CD22-CAR T cell therapy. First, in a subset of subjects, there is poor CAR T cell expansion following infusion of a product that appears to meet appropriate release criteria. We hypothesize that such products can be identified by the presence of exhausted or terminally differentiated cells that lack progenitor capacity. To explore the potential to identify such products *a priori*, we will analyze apheresis samples and manufactured products to enumerate the frequency of naïve, T stem cell memory, T central memory and T effective memory subsets. We will also analyze apheresis and manufactured products for the presence of markers of T cell

exhaustion including PD-1, Tim3 and LAG-3. Phenotypes will be correlated with the degree of expansion following CAR T cell infusion.

Second, we hypothesize that a subset of subjects will undergo efficient early T cell expansion but will demonstrate loss of CAR-T cell persistence associated with the acquisition of a terminally differentiated phenotype and/or characteristics of T cell exhaustion. To test this, we will evaluate CAR expressing T cells *in vivo* following adoptive transfer to identify characteristics associated with poor persistence. These phenotypic analyses will include flow cytometry and mass cytometry with a goal of identifying T cell phenotypes associated with poor persistence *in vivo*. We will also undertake epigenetic analyses to analyze correlations between T cell phenotype, persistence and enhancer profiles. Where possible, we will also compare the phenotype of cells harvested from blood, bone marrow, CSF and lymph nodes to analyze whether the tissue microenvironment has substantial impact on T cell exhaustion, phenotype or function.

Finally, we hypothesize that a subset of subjects lose their CAR T cell populations as a result of immune rejection. To test this, we will analyze for the presence or development of anti-CAR mediated T cell responses using cell based assays, as well as overlapping peptide libraries to localize the targets of anti-CAR reactivity.

2.6.2 Impact of Persistent CD19/CD22 Immune Pressure on Malignant and Normal B cell Differentiation

CD19 and CD22 are acquired during the process of normal and malignant B cell differentiation. Persistent immune based pressure targeting these two antigens has the potential to induce profound changes on both normal and malignant B cell differentiation. In this study, we will use mass cytometry, flow cytometry and ATAC-Seq to monitor changes in normal and malignant B cell differentiation in the presence of persistent CAR T cells targeting these antigens. We will explore whether this dual targeted therapeutic is capable of enabling emergence of malignancy that demonstrates loss of either or both antigens and whether such antigen loss is associated with a particular differentiation state, a particular genotype and/or are associated with increased or diminished fitness as evidenced by clinical behavior, or where possible growth patterns *ex vivo*. Similar analyses will focus on subjects with remission marrows, analyzing whether normal B cell development can proceed in the absence of CD19 and/or CD22 subpopulations. This will provide a novel opportunity to characterize the impact of CD19 and/or CD22 loss on normal lymphoid and/or myeloid cells development.

2.6.3 Utility of minimal residual disease assessment

A reliable biomarker that detects disease before it is evident clinically or on imaging offers potential to improve long-term disease control and survival. Necrosis and apoptosis of lymphoma cells lead to release of tumor DNA in blood circulation[80]. Next-generation sequencing and high-throughput technology can non-invasively detect and quantify the circulating free DNA (cfDNA)[81]. The VDJ immunoglobulin genes contain unique sequences that are markers of clonality[82]. These clonal markers can be used to assess MRD after treatment and have been shown to have prognostic value in DLBCL post-treatment surveillance[81] and other lymphoid malignancies[83],[84].

The role of MRD testing and cfDNA assessment in subjects treated with CAR-T cells has not been well studied. In this study we will correlate cfDNA assessment with marrow flow cytometry to identify the timing of relapse identification using these two approaches.

2.7 PROTOCOL RATIONALE AND SUMMARY

Most aggressive cancers eventually relapse or become refractory to current conventional treatments, necessitating the immediate development of new treatment modalities. The most recent advances in cancer therapy involve the development of new immune stimulating agents designed to target T-cells to cancer cells and initiated cell death signaling pathways. However, limitations to this type of immunotherapy include lack of tumor specificity, insufficient T-cell recruitment/numbers, and the existence of endogenous immunosuppressive agents which significantly attenuate or altogether block T-cell activity.

Chimeric Antigen Receptor (CAR) expressing T-cells is a new therapy wherein a subject's own T-cells are harvested and subsequently genetically modified in order to target cell surface antigens on specific cancer cells. In addition to their specificity, these CAR T-cells can be modified to be highly proliferative and possess the ability to negate immunosuppressive mechanisms making them ideal agents against highly aggressive cancers.

Specific to this study, bivalent CARs expressing both CD19 and CD22 will be utilized in order to avoid the immune escape that occurs when only a single antigen is targeted.

The primary goal of this trial is to evaluate the feasibility of generating CD19/CD22-CAR T cells to meet the manufacturing specifications and to assess the safety of administering CD19/CD22-CAR T cells after a conditioning lymphodepletion chemotherapy regimen in one treatment group: adults with B cell malignancies. Secondary aims will assess in a preliminary fashion, the clinical activity of this regimen. The preparative regimen cyclophosphamide and fludarabine is one that has been used successfully in many clinical trials with acceptable toxicity profile.

Subjects with B-ALL or DLBCL are eligible. Previous immunotherapy will not render subjects ineligible and subject eligibility requires relapsed or refractory disease after standard therapies. Subjects who have previously received CAR T cell therapy, the level of circulating CAR T cells must be < 5% of T cells at the time of enrollment. Subjects who have undergone allogeneic SCT will be eligible if, in addition to meeting other eligibility criteria, they are at least 100 days post-transplant, have no evidence of active GVHD and have been without immunosuppressive agents for at least 30 days. CD19 expression is required for enrollment based upon evidence that the CD19/CD22-CAR shows diminished reactivity toward CD19– blasts compared to the monovalent CD22-CAR already in clinical trials. In contrast, the CD19/CD22-CAR shows essentially equivalent reactivity toward CD19+CD22+ and CD19+CD22– lymphoblasts, therefore CD22 expression will not be an eligibility requirement. However where possible CD22 expression will be monitored and evaluated retrospectively for potential associations with outcome.

2.7.1 Dose Escalation

As described in section 2.2, the experience to date with anti-CD19 CAR and the POB NCI experience thus far with CD22-CAR trials is that dose level 3 x 10^5/kg is not effective for adequate CAR T cell expansion and that the dose that is both safe and efficacious is 1 x 10^6 transduced CAR T cells/kg. Given that both the CD19 and the CD22-CAR have been clinically tested at the 1 x 10^6 cells/kg dose, our proposal is to initiate this protocol at this dose level with a limited dose escalation and/or de-escalation if needed, utilizing a 3+3 Phase 1 design, utilizing a biologically active and well tolerated dose for both CD19 and CD22-CAR trials. With Amendment 1, the CD19/CD22-CAR T cell culture will be reduced from approximately 9 days to approximately 7 days. To ensure safety with the younger cell product, 3 additional subjects will be enrolled in the current enrolling dose cohort. A seven day window will be incorporated between subjects following the shortened cultured cell administration to allow for safety evaluation. If tolerated and safety has been assessed, we will have a very limited dose escalation to 3 x 10^6/kg. If tolerated, consideration of 1 x 10^7/kg may be given if there are concerns for inefficacy or limited persistence in low burden disease with manageable tolerability of a higher CAR dose.

This study will be conducted at Stanford by the principal investigator David Miklos, MD, PhD. The Sponsor of this study will be Crystal Mackall, MD, Professor Pediatrics & Medicine, Associate Director, Stanford Cancer Institute.

2.8 Study Design

2.8.1 Short Title for Study

CD19/CD22-CAR T-cells in adults with B-cell malignancies

2.8.2 Interventional model

Single arm, open label dose escalation model

2.8.3 Primary Outcome Type

Safety and feasibility

2.8.4 Investigational Agent

Autologous T cells transduced with bivalent lentiviral vector (CD19/CD22.BB.z) Chimeric Antigen Receptor (CD19/CD22-CAR); following Fludarabine and Cyclophosphamide

2.8.5 IND number:

017484, Sponsor: Crystal L. Mackall, M.D.

2.8.6 Primary outcome measures:

1. Rate of successful manufacture and expansion of CD19/CD22-CAR T cells to satisfy the target dose level and meet release specifications
2. Dose limiting toxicities (DLT) and maximum tolerated dose (MTD) of CD19/CD22-CAR T cells in adults with B cell malignancies

2.8.7 Primary outcome timeframe:

1. 7-14 days after apheresis or thawing of cryopreserved PBMCs
2. 28 days after infusion of CD19/CD22-CAR T cells

2.8.8 General Study Design

This is a phase 1 study in adult subjects with B cell hematologic malignancies who have relapsed or refractory disease after standard treatment options. Autologous PBMC will be obtained by leukapheresis and transduced with CD19/CD22-CAR lentiviral vector. Cryopreserved PBMC stored from participation in other institutional cell therapy or cell collection studies may be used to generate the cellular product on this study as long as they meet the criteria established in this IND.

Subjects will receive a lymphodepleting preparative regimen of fludarabine and cyclophosphamide followed by infusion of CD19/CD22-CAR T-cells. Subjects will be evaluated sequentially after treatment for toxicity, antitumor effects and for persistence of CAR in blood samples and functionality of transduced T cells. Additional blood and bone marrow samples will be collected to complete correlative study analysis.

Initially subjects will receive 1 x 10^6 transduced T cells/kg (± 20%) as outlined in section **5.4.2.** If 2/6 subjects experience DLT at dose level 1, dose -1 will be explored (3 x 10^5 transduced T cells/kg (± 20%)) as described in section **5.7.3**. The MTD, or highest cell dose studied if MTD is not reached, will be expanded for at least 15 and up to 30 evaluable subjects (including those treated in dose escalation at MTD) in two groups: 1) adults with DLBCL and 2) adults with ALL, to further explore the safety, feasibility and clinical response activity.

2.8.9 Number of Subjects

Up to 6 evaluable subjects may be enrolled in cohorts 1 to 3. With Amendment 1 the culture time for generating CD19/CD22-CAR T cells is shortened to approximately 7 days. To ensure safety, 3 additional subjects will be treated at dose level 1 with cells generated with the shortened culture time (21 total in order to determine a safe and feasible dose), allowing at least 14 days between each infusion. The study will allow for up to 3 subjects to be replaced in each of the dose cohorts (9 additional subjects) due to inability to achieve target doses. In addition, the study will allow for 6 total inevaluable subjects (subjects enrolled but who cannot receive cells, either due to physical deterioration or withdrawn consent during cell growth). Thus, 21 + 9 + 6 yields a maximum of 36 subjects who may be enrolled to determine a safe and feasible dose. In the dose expansion, two cohorts of additional subjects will be enrolled at the MTD (or highest dose tested) for a minimum of 15 evaluable subjects (including those tested in dose escalation) per cohort 1) Adults with DLBCL and 2) Adults with ALL. If > 5 of 15 subjects at MTD achieve an overall response (OR) for DLBCL or complete response (CR) for ALL, enrollment at MTD will be expanded to a total of 30 subjects per cohort. Therefore, a minimum of 24 and a maximum of 90 subjects may be enrolled on this trial.

2.8.10 Study Duration

2.8.10.1 Primary Completion:

Anticipate 2 subjects per month, with future increase in production for expansion cohorts, and therefore this study may require up to 3 to 4 years to complete accrual. Subjects will be followed for 5 years after treatment to evaluate toxicities, disease progression, and evaluate for gene therapy effects. Therefore, the total duration of this study to meet the primary objective is expected to be –8 - 9 years of active treatment and follow up.

2.8.10.2 Study Completion:

In addition, follow up for gene therapy according to the U.S. Food and Drug Administration (FDA) ***Guidance for Industry: Gene Therapy Clinical Trials – Observing Participants for Delayed Adverse Events*** will be conducted on this study or a long-term follow up study for subjects participating in genetic research for the required 15 years, for a total of 18 - 19 years until study completion.

3 PARTICIPANT SELECTION AND ENROLLMENT PROCEDURES

All subjects must sign and date the IRB/IEC approved consent form before initiating any study specific procedures or activities that are not part of a subject's routine care.

Each subject who enrolls in the study will receive a unique subject identification number before any study specific procedures or activities are initiated. This number will be used to identify the subject throughout the study and must be used on all study documentation related to the subject. Furthermore, the subject identification number must remain constant throughout the entire clinical study, it must not be changed if the subject is rescreened or retreated.

Subjects with B cell malignancies who have relapsed or are treatment refractory may enroll as defined by the following inclusion and exclusion criteria in the Screening Participant Eligibility Checklist. The Screening Participant Eligibility Checklist on the following page must be completed in its entirety for each subject prior to registration. The completed, signed, and dated checklist must be retained in the subject's study file and the study's Regulatory Binder or an electronic version completed within the subject's medical record.

The study coordinator, treating physician, and an independent reviewer must verify that the participant's eligibility is accurate, complete, and legible in source records, as required by the CCTO SOP 'Confirmation of Participant Eligibility in Clinical Trials'. A description of the eligibility verification process should be included in the EPIC or other Electronic Medical Record progress note.

The protocol-specific checklist is **required** by the SRC and must be approved by the IRB.

3.1 SCREENING PARTICIPANT ELIGIBILITY CHECKLIST

Protocol Title:	**Phase 1 Dose Escalation Study of CD19/CD22-Chimeric Antigen Receptor (CAR) T cells in Adults with Recurrent or Refractory B Cell Malignancies**
Protocol Number:	**CCT5001/IRB-41382**
Principal Investigator:	**David Miklos, M.D., Ph.D.**

II. Subject Information:

Subject Name/ID:
Gender: ☐ Male ☐ Female

III. Study Information:

SRC Approved ☐ IRB Approved ☐ Contract signed ☐

IV. Inclusion/Exclusion Criteria

Inclusion Criteria	Yes	No	Supporting Documentation*
1) <u>For DLBCL only:</u><br>A. Histologically confirmed aggressive B cell NHL including the following types defined by WHO 2008:<br>• DLBCL not otherwise specified; T cell/histiocyte rich large B cell lymphoma; DLBCL associated with chronic inflammation; Epstein-Barr virus (EBV)+ DLBCL of the elderly; OR<br>• primary mediastinal (thymic) large B cell lymphoma<br>• transformation of follicular lymphoma, marginal zone lymphoma or chronic lymphocytic leukemia to DLBCL will also be included	☐	☐	
B. Subjects with DLBCL must have progressed, had SD, or recurred after initial treatment regimens that include an anthracycline and an anti-CD20 monoclonal antibody. Subjects who relapse ≥12 months after therapy should have progressed after autologous transplant or been ineligible for autologous transplant.	☐	☐	

Inclusion Criteria	Yes	No	Supporting Documentation*
1) For B-ALL only:			
A. Confirmed diagnosis of relapsed or refractory B-cell ALL of one of the following types:<br>• Chemotherapy refractory disease in subjects with B-ALL, defined as progression or stable disease after two lines of therapies<br>• Recurrence of disease after achieving CR.	☐	☐	
B. Subjects with persistent or relapsed minimal residual disease (MRD, by flow cytometry, PCR, FISH, or next generation sequencing) require verification of MRD positivity on two occasions at least 4 weeks apart.	☐	☐	
C. Subjects with Philadelphia Chromosome positive acute lymphoblastic leukemia (Ph+ALL) subjects are eligible if they progressed, had stable disease or relapsed after two lines of therapy, including tyrosine kinase inhibitors (TKIs).	☐	☐	
D. Subjects with recurrence of isolated CNS relapse after achieving complete remission (CR); if relapsed with MRD, will require verification of MRD positivity on two occasions at least 4 weeks apart.	☐	☐	
2) CD19 positive expression<br>CD19 expression is required any time since diagnosis. If patient has received anti-CD19 targeted therapy (i.e. Blinatumomab or CD19-CAR T cell), the CD19 expression must be subsequently demonstrated. CD19 expression may be detected by immunohistochemistry or by flow cytometry. The choice of whether to use flow cytometry or immunohistochemistry will be determined by what is the most easily available tissue sample in each subject. In general, immunohistochemistry will be used for lymph node biopsies, flow cytometry will be used for peripheral blood and bone marrow samples.	☐	☐	

Inclusion Criteria	Yes	No	Supporting Documentation*
3) Subjects who have undergone autologous SCT with disease progression or relapse following SCT are eligible. Subjects who have undergone allogeneic SCT will be eligible if, in addition to meeting other eligibility criteria, they are at least 100 days post-transplant, they have no evidence of GVHD and have been without immunosuppressive agents for at least 30 days.	☐	☐	
4) Subjects who have undergone prior anti-CD19 or anti-CD22 CAR therapy will be eligible if < 5% of circulating levels of CD3+ cells express the previous CAR by flow cytometry.	☐	☐	
5) Must have evaluable or measurable disease; subjects with lymphoma must have evaluable or measurable disease according to the revised IWG Response Criteria for Malignant Lymphoma (Cheson 2014). Lesions that have been previously irradiated will be considered measurable only if progression has been documented following completion of radiation therapy.	☐	☐	
6) At least 2 weeks or 5 half-lives, whichever is shorter, must have elapsed since any prior systemic therapy at the time the subject is planned for leukapheresis, except for systemic inhibitory/stimulatory immune checkpoint therapy, which requires 5 half-lives.<br>*Exceptions:*<br>a. There is no time restriction with regard to prior intrathecal chemotherapy (incl. steroids) provided there is complete recovery from any acute toxic effects;<br>b. Subjects receiving hydroxyurea may be enrolled provided there has been no increase in dose for at least 2 weeks prior to starting apheresis;<br>c. Subjects who are on standard ALL maintenance type chemotherapy (vincristine, 6-mercaptopurine or oral methotrexate) may be enrolled provided that chemotherapy is discontinued at least	☐	☐	

Inclusion Criteria	Yes	No	Supporting Documentation*
1 week or 5 half-lives (whichever is shorter) prior to apheresis.<br>d. Subjects receiving steroid therapy at physiologic replacement doses (≥5 mg/day of prednisone or equivalent doses of other corticosteroids) only are allowed provided there has been no increase in dose for at least 2 weeks prior to starting apheresis;<br>e. For radiation therapy: Radiation therapy must have been completed at least 3 weeks prior to enrollment, with the exception that there is no time restriction if the volume of bone marrow treated is less than 10% and also the subject has measurable/evaluable disease outside the radiation port.			
7) Toxicities due to prior therapy must be stable and recovered to ≤ Grade 1 (except for clinically non-significant toxicities such as alopecia)	☐	☐	
8) Age 18 or older	☐	☐	
9) Eastern cooperative oncology group (ECOG) performance status of 0, 1, or 2; or Karnofsky ≥ 60% (See **Section 14.1**).	☐	☐	
10) Normal Organ and Marrow Function (supportive care is allowed per institutional standards, i.e. filgrastim, transfusion)<br>• ANC ≥ 750/uL*<br>• Platelet count ≥ 50,000/uL*<br>• Absolute lymphocyte count ≥ 150/uL*<br>Adequate renal, hepatic, pulmonary and cardiac function defined as:<br>• Creatinine ≤ 2 mg/dL or creatinine clearance (as estimated by Cockcroft Gault Equation) ≥ 60 mL/min<br>• Serum ALT or AST ≤ 10x ULN (Elevated ALT/AST associated with leukemia or lymphoma involvement of the liver will not disqualify a subject; only one value required for eligibility)<br>• Total bilirubin ≤ 1.5 mg/dl, except in subjects with Gilbert's syndrome.<br>• Cardiac ejection fraction ≥ 45%, no evidence of physiologically significant	☐<br>ANC<br>Platelet<br>ALC<br>Creatinine<br>AST/ALT<br>Bilirubin<br>LVEF	☐	

Inclusion Criteria	Yes	No	Supporting Documentation*
pericardial effusion as determined by an ECHO, MUGA or Cardiac MRI [performed **within 180 days or after most recent anthracycline based treatment or mediastinal radiation therapy** (whichever is most recent)]<br>• No clinically significant ECG findings<br>• No clinically significant pleural effusion<br>• Baseline oxygen saturation > 92% on room air<br>* A subject will not be excluded because of pancytopenia ≥ Grade 3 if it is felt by the investigator to be due to underlying leukemia/lymphoma.	ECG<br>Pleural<br>O_2 Sat		
11) CNS Status<br>Subjects with CNS involvement are eligible as long as there are no overt signs or symptoms that in the evaluation of the investigator would mask or interfere with the neurological assessment of toxicity.	☐	☐	
12) Females of childbearing potential must have a negative serum or urine pregnancy test (females who have undergone surgical sterilization or who have been postmenopausal for at least 2 years are not considered to be of childbearing potential)	☐	☐	
13) Contraception<br>Subjects of child-bearing or child-fathering potential must be willing to practice birth control from the time of enrollment on this study and for four (4) months after receiving the preparative lymphodepletion regimen.	☐	☐	
14) Ability to give informed consent.<br>Must be able to give informed consent. Subjects unable to give informed consent will not be eligible for this study.	☐	☐	

Exclusion Criteria	Yes	No	Supporting Documentation
1) History of other malignancy, unless disease free for at least 3 years. At the discretion of the Principal Investigator, subjects in	☐	☐	

Exclusion Criteria	Yes	No	Supporting Documentation
remission for 1-2 years prior to enrollment may be deemed eligible after considering the nature of other malignancy, likelihood of recurrence during one year following CAR therapy, and impact of prior treatment on risk of CD19/CD22-CAR T cells. Subjects in remission <1 year are not eligible.<br>• Exception: Nonmelanoma skin cancer or carcinoma in situ (e.g. cervix, bladder, breast) is eligible.<br>• Hormonal therapy in subjects in remission >1 year will be allowed.			
2) Presence of fungal, bacterial, viral, or other infection that is uncontrolled or requiring IV antimicrobials for management. Simple UTI and uncomplicated bacterial pharyngitis are permitted if responding to active treatment.	☐	☐	
3) Known history of infection with any of the following:<br>• HIV<br>• Hepatitis B (HBsAg positive)<br>• Hepatitis C virus (anti-HCV positive)<br>A history of hepatitis B or hepatitis C is permitted if the viral load is undetectable per quantitative PCR and/or nucleic acid testing.	☐<br>HIV<br>HBV<br>HCV	☐	
4) CNS disorder such as seizure disorder, cerebrovascular ischemia/hemorrhage, dementia, cerebellar disease, or any autoimmune disease with CNS involvement that in the judgment of the investigator may impair the ability to evaluate neurotoxicity.	☐	☐	
5) History of myocardial infarction, cardiac angioplasty or stenting, unstable angina, or other clinically significant cardiac disease within 12 months of enrollment.	☐	☐	
6) Any medical condition that in the judgement of the investigator is likely to interfere with assessment of safety or efficacy of study treatment	☐	☐	
7) History of severe immediate hypersensitivity reaction to any of the agents used in this study	☐	☐	

Exclusion Criteria	Yes	No	Supporting Documentation
8) Women who are pregnant or breastfeeding.	☐	☐	
9) In the investigators judgment, the subject is unlikely to complete all protocol-required study visits or procedures, including follow-up visits, or comply with the study requirements for participation	☐	☐	
10) Has primary immunodeficiency or history of autoimmune disease (e.g. Crohns, rheumatoid arthritis, systemic lupus) requiring systemic immunosuppression/systemic disease modifying agents within the last 2 years	☐	☐	

*****All subject files must include supporting documentation to confirm subject eligibility. The method of confirmation can include, but is not limited to, laboratory test results, radiology test results, subject self-report, and medical record review.

IV. Statement of Eligibility

By signing this form of this trial I verify that this subject is [☐ **eligible** / ☐ **ineligible**] for participation in the study. This study is approved by the Stanford Cancer Institute Scientific Review Committee, the Stanford IRB, and has finalized financial and contractual agreements as required by Stanford School of Medicine's Research Management Group.

Treating Physician Signature:	Date:
Printed Name:	
Secondary Reviewer Signature:	Date:
Printed Name:	
Study Coordinator Signature:	Date:
Printed Name:	

3.2 Informed Consent Process

All participants must be provided a consent form describing the study with sufficient information for participants to make an informed decision regarding their participation. The investigational nature and research objectives of this trial, the procedures and treatments involved and their attendant risks and discomforts and potential benefits, and alternative therapies will be carefully explained to the subject, and asked to review it and to ask questions prior to agreeing to participate in this protocol. The subject is reassured that participation on trial is entirely voluntary and that he/she can withdraw or decide against treatment at any time without adverse consequences.

The original signed copy of the consent document must be retained in the medical record or research file.

3.3 Subject Screening Assessments and Registration

3.3.1 General considerations for Subject Screening

The screening period begins on the date the subject signs the IRB/IEC approved ICF and continues through confirmation of enrollment (the date triple review eligibility sign off is completed to verify that the participant's eligibility is accurate, complete, and legible in source records). Informed consent must be obtained before completion of any non-standard of care study specific procedures. Procedures that are part of standard of care are not considered study specific procedures and may be performed prior to obtaining consent and used to confirm eligibility. Confirmation of these data must occur within 28 days of enrollment unless otherwise specified. CD19 expression may be tested anytime since diagnosis. If the subject has received anti-CD19 targeted therapy (i.e. Blinatumomab or CD19-CAR T cells), then CD19 expression must be subsequently demonstrated. In addition, viral and pregnancy testing must be performed prior to apheresis, according to the timing requirements established by the FDA (21 CFR 1271 Subpart C), the Stanford Apheresis Unit, and the state of California.

After written informed consent has been obtained, subjects will be screened to confirm study eligibility and participation. Only subjects who meet the eligibility criteria listed in sections **3.1** and **3.2** will be enrolled in the study. If at any time prior to enrollment the subject fails to meet the eligibility criteria, the subject should be designated as a screen failure on the subject screening log with the reasons for failing screening.

All subjects will undergo the screening procedures, which includes a comprehensive history and physical exam performed by a study physician or nurse practitioner. Imaging, bone marrow biopsy and aspiration, and organ specific studies will be performed as per institutional guidelines. The screening tests in Section 5.1 must be performed within 28 days of enrollment unless specified otherwise.

3.3.2 Study Enrollment

Before enrollment of a subject into the study, the responsible physician must ensure the subject meets all eligibility criteria using the Study Screening Procedures outlined in section **5.1**. Eligibility criteria will be reviewed and confirmed by the Investigator prior to any subject being enrolled into the study as described in **Section 3.1**.

Enrollment will be defined as the date triple review eligibility sign off is completed to verify that the participant's eligibility is accurate, complete, and legible in source records. At the time of enrollment, the subject will be assigned a unique study number.

4 TREATMENT PLAN

4.1 Overview

This is a phase 1 dose escalation study in adult subjects with B cell malignancies who have relapsed or refractory disease. Autologous PBMC will be obtained by leukapheresis and transduced with CD19/CD22-CAR lentiviral vector. Cryopreserved PBMC stored from participation in other institutional cell therapy, cell collection studies, or standard of care may be used to generate the cellular product on this study as long as they meet the criteria established in this IND.

Subjects will receive a lymphodepleting preparative regimen of fludarabine and cyclophosphamide followed by infusion of CD19/CD22-CAR transduced T-cells. Subjects will be evaluated sequentially after treatment for toxicity, antitumor effects and for persistence of CAR in blood samples and functionality of transduced T cells.

Three dose levels of CD19/CD22-CAR transduced T-cells will be administered in escalating cohorts. Dose escalation will proceed as described in section **5.7.3**. If 2/6 subjects experience DLT at dose level 1, dose -1 will be explored (3×10^5 transduced T cells/kg (± 20%)). The MTD, or highest cell dose studied if MTD is not reached, will be expanded in two groups (subjects with DLBCL and subjects with ALL) to a total of at least 15 and up to 30 evaluable subjects in each group to further explore the safety, feasibility and clinical response activity.

4.2 General Concomitant Medication and Supportive Care Guidelines

4.2.1 Infection Prophylaxis

Any temperature of > 38° C will require initiation of the fever work-up and treatment according to institutional standards.

4.2.1.1 Viral Prophylaxis

All subjects will be treated as high risk and will receive viral prophylaxis according to institutional standards.

4.2.1.2 Fungal Prophylaxis

All subjects will receive fungal prophylaxis according to institutional standards.

4.2.2 B-cell Depletion

Since B cell antigen-targeted immunotherapy has been demonstrated in other studies to also eliminate normal B cells, serum IgG levels will be monitored before cell infusion and every 3-12 weeks after infusion. If IgG < 500, IVIG will be administered with the appropriate premedications as per institutional guidelines.

4.2.3 Blood Product Support for Anemia and Thrombocytopenia

Using daily CBC's as a guide, the subject will receive platelets and packed red blood cells (PRBC's) as needed. Attempts will be made to keep Hb > 8.0 gm/dl, and plts > 10,000/mm3. All blood products with the exception of the lymphocyte product will be irradiated. Leukocyte filters will be utilized for all blood and platelet transfusions to decrease sensitization to transfused WBC's and decrease the risk of CMV infection.

In subjects with coagulopathy, attempts will be made to keep platelets > 50,000/mm3 and fibrinogen ≥ the lower limit of normal.

4.2.4 Tumor Lysis Syndrome

Subjects at risk for tumor lysis syndrome, as per the investigator's discretion, will receive allopurinol and hydration prophylactically as per the institutional guidance/managing team's determination.

4.2.5 Anti-Neoplastic Therapy

Patients must have evaluable disease at the time of protocol enrollment. PBMC will be collected and every effort will be made to initiate cell culture within 30 days so minimal time transpires for a patient's disease to progress significantly. In the event that circumstances external to this study arise and a patient develops significant disease progression, patients may receive disease-directed therapy (standard or investigational) prior to moving to the treatment phase of this study. Patients who require therapy will have the apheresis product stored for use in the future. Since the toxicity of CAR based therapy is related to disease burden (more disease associated with more CAR related toxicity) and because the time to initiation of conditioning lymphodepletion regimen may be delayed due to multiple reasons, our experience to date indicates that patients may have rapid progression of their disease in this interval risking increased toxicity from either CAR therapy or disease progression. Such patients may be treated with standard of care therapy (non-investigational) according to institutional guidelines and treating physician preference. Treatment data and unresolved toxicities will be captured in baseline assessment CRFs. In such circumstances, in order to initiate the conditioning lymphodepletion regimen**,** there must be 14 days from the last dose of standard chemotherapy (7 days from ALL maintenance therapy with the exclusion of steroids which may be continued until starting conditioning lymphodepletion regimen). In order to proceed, patient must meet the criteria established in Section **5.3** in addition to limited eligibility criteria based on the following evaluations: general blood tests (CBC, chemistries including sodium, potassium, chloride, total CO2, creatinine, glucose, urea nitrogen, albumin, alkaline phosphatase, ALT/SGPT, AST/SGOT, total bilirubin,) and disease evaluation (bone marrow aspirate/biopsy and lumbar puncture per PI discretion).

4.2.6 Cytokine Release Syndrome

Cytokine release syndrome, a toxicity associated with infusion of CAR T cell therapy, has been described in section **2.5.4.** Grading and management of CRS for the dose escalation cohort followed the guidelines in section **14.3** Appendix C[7]. New guidance for scoring of CRS and neurotoxicity was published in 2018 by the ASBMT [85].

Retroactive to all participants in the dose expansion cohort, CRS will be graded based on the revised CRS criteria proposed by ASBMT (see **Section 14.2.1**). Proper management of CRS includes diligent supportive care and search for infection, with immunosuppression using anti-IL6R mAbs and/or corticosteroids reserved for more severe cases. Symptom management recommendations in **Section 14.2.2** should serve as guidance for toxicity management, but deviation from the guidance will not be considered a protocol deviation, as all toxicity therapy should be at the treating physician's discretion.

4.2.7 Neurotoxicity

Neurotoxicity (e.g., encephalopathy, somnolence, aphasia) has been observed with CAR T cell therapies. All subjects will receive levetiracetam 500mg or 750mg twice daily (approximately 10 mg/kg/dose orally twice a day) beginning the day before cell infusion, which should continue until investigator judges neurotoxicity risk is resolved. For the dose escalation cohort, neurotoxicity was assessed using the CRES score and graded using CARTOX criteria [86] (see **Section 14.3**, Appendix C). Retroactive to all participants in the dose expansion cohort, neurotoxicity will be graded based on the Immune effector cell-associated neurotoxicity syndrome (ICANS) criteria (see **Section 14.2.3**). Clinicians and staff will evaluate the Immune Effector Cell-Associated Encephalopathy (ICE) score every 8 hours (± 2 hr), or at every shift change, while hospitalized up to Day 14; with daily documentation of the maximum score for each day. Neurologic evaluations and ICANS grading will be assessed daily and with a drop in ICE/CRES score below 7 out of 10. The neurotoxicity management recommendations in **Section 14.2.4** should serve as guidance for toxicity management, but deviation from the guidance will not be considered a protocol deviation, as all toxicity therapy should be at the treating physician's discretion.

Medications with sedative properties should be avoided if possible unless required to manage seizures, i.e. benzodiazepines. Leukoencephalopathy has been observed on MRI in the setting of neurotoxicity. Follow-up MRI is recommended to monitor the course of leukoencephalopathy to potential resolution.

Subjects and their families/caregivers should be warned of the risk of late neurotoxicity thru day 28 and told to seek immediate medical attention for any new symptoms of neurotoxicity. In addition, subjects should be advised not to drive or operate heavy machinery for the first month after discharge and/or until 1 month after complete resolution of neurotoxicity symptoms.

4.3 Criteria for Removal from Protocol Therapy and Off Study Criteria

4.3.1 Criteria for removal prior to CD19/CD22-CAR T cell infusion

Subjects will be taken off treatment and followed until effects of chemotherapy have reversed and all toxicities are resolved to Grade 1 or baseline for any of the following:

- ✓ General or specific changes in the subject's condition render the subject unacceptable for cell infusion on this study in the judgment of the investigator.
- ✓ Pregnancy in a female of child-bearing potential.
- ✓ Cells do not meet infusion release criteria.

Once toxicities and effects of chemotherapy resolve, subjects who are unable to undergo cell infusion will be removed from this study.

4.3.2 Criteria for removal from the option for a 2nd cell infusion:

Subjects will not be eligible for further therapy (2nd cell infusion) (and will be followed until off-study criteria are met) for the following:

- ✓ Dose limiting toxicity (DLT) after first infusion. The definition of DLT is in section **5.7.1.**
- ✓ Pregnancy in a female of child-bearing potential.
- ✓ No cells available for re-treatment.

4.3.3 Off-Study Criteria

- ✓ Subject withdrawal from protocol (in which case the reason will be documented, if possible)
- ✓ Subject unable to receive CD19/CD22-CAR T cell infusion and has recovered from all study induced toxicity.
- ✓ Death
- ✓ Conclusion of the 15 years of follow up, or subject enrolls in long-term follow up protocol for subjects receiving gene transfer.

4.3.4 Off-Study Procedure

Off study date and reason should be documented in the study CRFs.

5 SUBJECT STUDY PROCEDURES

5.1 Subject Screening

The screening period begins on the date the subject signs the IRB approved ICF and continues through date of enrollment. Procedures that are to be performed as part of the practice of medicine and which would be done whether or not study entry was contemplated, such as for diagnosis or treatment of a disease or medical condition, may be performed and the results subsequently used for determining study eligibility without first obtaining consent. On the other hand, informed consent must be obtained prior to initiation of any clinical screening procedures that are performed solely for the purpose of determining eligibility for research, i.e. withdrawal from medication (wash-out period). Only subjects who meet the eligibility criteria listed in section **3** (See Eligibility checklist) will be enrolled in the study. The date of enrollment is defined as the date triple review

eligibility sign off is completed to verify that the participant's eligibility is accurate, complete, and legible in source records.

All subjects will undergo the screening procedure, which includes a comprehensive history and physical exam performed by a study physician as outlined below. Imaging, bone marrow biopsy and aspiration, and organ specific studies will be performed as per institutional guidelines. The following screening tests must be performed within **28 days** prior to enrollment, **unless specified otherwise**.

(a) CD19 expression

Expression will be evaluated by immunohistochemistry or by flow cytometry in a CLIA approved laboratory and documentation at screening will be required (**testing is permitted to be conducted at any time since diagnosis, unless subject has received anti-CD19 targeted therapy such as Blinatumomab or CD19-CAR T cells, in which case the specimen must be collected after such therapy)**.

(b) Medical history

The subject's complete history through review of medical records and by interview will be collected and recorded. Concurrent medical signs and symptoms must be documented to establish baseline severities. A disease history, including the date of initial diagnosis and list of all prior treatment, responses, and duration of response to the prior treatment also will be recorded. A list of concurrent mediations current at time of enrollment will be recorded.

(c) Physical examination

A complete physical examination will be performed. The exam will include general appearance of the subject, height and weight, examination of the skin, eyes and ears, nose, throat, lungs, heart, abdomen, extremities, musculoskeletal system, and nervous system. Vital signs, including blood pressure, heart rate, respiratory rate, oxygen saturation and temperature will be recorded.

(d) Performance status (ECOG or Karnofsky)

(e) ECG

(f) Evaluation for HIV seropositivity to consist of ELISA and, if positive, confirmation by Western blot within the time requirements for autologous apheresis donation. The investigator, in the event of a positive finding, will make appropriate counseling available.

(g) Evaluation for Hepatitis B surface antigen (HbsAG) and anti-HCV antibodies, within the time requirements for autologous apheresis donation.

(h) Creatinine clearance: A measured 24 hour urine creatinine clearance test may be performed if the serum creatinine is elevated, and the measured value will be recorded in the CRF and may be used to qualify the subject for study participation.

(i) β-HCG pregnancy test on all women of child-bearing potential (within 28 days of enrollment)

(j) PT/PTT

(k) General Laboratory Tests: The following will be obtained during the screening process:

- ✓ Chemistries: (sodium, potassium, chloride, bicarbonate, BUN, creatinine, glucose, calcium, AST and/or ALT, alkaline phosphatase, bilirubin, albumin, total protein)
- ✓ Phosphorus and magnesium
- ✓ CBC with differential
- ✓ CRP and ferritin
- ✓ Urinalysis

(l) Additional Tests:

The PI may order additional tests in some subjects if needed to fully assess clinical status and obtain baseline results. Examples of such tests include: haptoglobin, soluble IL2R, immunoglobulin levels, and viral serology or PCR for cytomegalovirus (CMV), herpes simplex virus (HSV), varicella zoster virus (VZV), and Epstein-Barr virus (EBV).

(m) ECHO, MUGA or Cardiac MRI for LVEF and pericardial effusion assessment

- o If performed **within 180 days or after most recent anthracycline based treatment or mediastinal radiation therapy** (whichever is most recent) may be used for confirmation of eligibility

(n) Disease Evaluation (methods will be determined by investigator based on subjects' type and location of disease, not all are required on all subjects) within 28 days of enrollment:

- ✓ Imaging Studies appropriate to sites relevant to the subject's disease; subjects with DLBCL will undergo PET/CT, other imaging studies, such as MRI of the brain, will be performed as determined by investigator;
- ✓ Bone marrow aspirate/biopsy
- ✓ Lymph node biopsy
- ✓ Lumbar puncture (all subjects will undergo LP at baseline).

5.2 LEUKAPHERESIS FOR CELL ACQUISITION:

Subjects will undergo single day apheresis according to institutional standards with the goal of obtaining a minimum of 1 x 10^8 CD3+ T cells for transduction, unless PBMCs have been cryopreserved previously that meet the requirements for this study, in which case cryopreserved PBMCs may be used to generate CD19/CD22-CAR T cells on this study if they meet criteria outlined in the IND.

Target cell number will be based upon the expected expansion and planned cell doses of transduced T cells/kg. The target will be 14-20 L apheresis (target 2 x 10^9 mononuclear cells). Prophylactic intravenous $CaCl_2$ and $MgSO_4$ infusions may be administered by the apheresis clinical team per standard operating procedures. Institutional guidelines will be followed for venous access and apheresis procedures.

If, after enrollment and leukapheresis, the decision is made to not proceed with CD19/CD22-CAR T cell manufacturing, the collected apheresis product will be made available to the subject for alternate CAR-T cell production, if requested.

Eligibility criteria to undergo leukapheresis:

- Subjects must have no evidence of a clinically significant uncontrolled infection prior to leukapheresis
- Absolute lymphocyte count ≥ 150/uL within last 7 days
- No new signs or symptoms of kidney or liver dysfunction outside eligibility criteria within last 7 days
- Must not be pregnant

Corticosteroid therapy at a pharmacologic dose (≥ 5 mg/day of prednisone or equivalent doses of other corticosteroids) and other immunosuppressive drugs must be avoided for 7 days prior to leukapheresis.

The following procedures/requirements will occur on the leukapheresis collection day (unless otherwise specified) and as outlined in the section 9 Study Calendar:

- Vital signs, including blood pressure, heart rate, respiratory rate, oxygen saturation, and temperature
- Weight (day of leukapheresis or day before)
- Labs (within 7 days prior to leukapheresis)
  - Chemistry panel (sodium, potassium, chloride, bicarbonate, BUN, creatinine, glucose, calcium, AST and/or ALT, alkaline phosphatase, bilirubin, albumin, total protein)
  - Phosphorus and magnesium
  - CBC with differential
  - C-reactive protein (CRP)
  - HIV, HBV, and HCV testing
  - Pregnancy test (females of child-bearing potential only)
- Leukapheresis
- Adverse/Serious Adverse Event reporting
- Concomitant medications documentation

5.3 CONDITIONING CHEMOTHERAPY

Subjects will receive a conditioning lymphodepletion regimen consisting of cyclophosphamide and fludarabine in order to induce lymphocyte depletion and create an optimal environment for expansion of CD19/CD22-CAR T cells *in vivo*. Subjects will initiate conditioning chemotherapy with cyclophosphamide and fludarabine beginning on

Day -5 through Day -3. The 3-day conditioning chemotherapy regimen may be administered in an outpatient setting per investigator's discretion. The dose calculation for the chemotherapy administration (on Days -5 through -3) will be based on the height and weight measured on day -6. At the investigator's discretion, the timing of the 3-day regimen of conditioning lymphodepletion chemotherapy may be adjusted based on the best interest of the patient.

Adult patients in the outpatient setting will receive Home IV fluid infusion of Normal saline 150 mL/hour for 12-24 hours before cyclophosphamide and fludarabine. After final infusion of cyclophosphamide and fludarabine, the patient will receive another 1 liter Normal saline before the Home IV fluid infusion is removed and subject discharged home. Subjects should be instructed to drink plenty of liquids during and for 24 hours following the chemotherapy. In general, subjects should be kept well-hydrated but closely monitored to prevent fluid overload.

Routine anti-emetic prophylaxis and treatment should be employed. Corticosteroids may not be used (except for physiologic replacement).

The criteria for initiating the conditioning regimen is as follows:

Subjects must have

- no evidence of uncontrolled infection,
- no clinically significant cardiac dysfunction,
- serum creatinine must be < 2 x ULN,
- no acute neurological toxicity > Grade 1 (with the exception of peripheral sensory neuropathy)
- repeat disease evaluation, at the discretion of the investigator, if the subject received any antineoplastic therapy after enrollment (per Section **4.2.5**), if greater than 28 days has elapsed between date of enrollment and start of conditioning lymphodepletion chemotherapy regimen, or if investigator believes disease may have significantly advanced since enrollment.

Should an event exceed these criteria immediately prior to conditioning chemotherapy, conditioning chemotherapy must be delayed until the event resolves to ≤ Grade 1 or baseline.

The following procedures will be completed daily during Day -5 to Day -3, unless otherwise specified:

- Weight (Day -5 or -6 only)
- Vital signs, including blood pressure, heart rate, respiratory rate, oxygen saturation, and temperature
- Labs
    - Chemistry Panel (sodium, potassium, chloride, bicarbonate, BUN, creatinine, glucose, calcium, AST and/or ALT, alkaline phosphatase, bilirubin, albumin, total protein)
    - Phosphorus, magnesium, uric acid, and LDH (on day -5 only)
    - CBC with differential
    - CRP and ferritin (on day -5 or -6 only)

  - o Pregnancy test (females of child-bearing potential only), performed within 7 days prior to day -5
- Fludarabine and cyclophosphamide administration
- Correlative samples as outlined in Section 14.9, Appendix I: Correlative Sample Schedule.
- Adverse/Serious Adverse Event reporting
- Concomitant medications documentation

Each subject will receive the lymphodepleting regimen as follows:

Drug	Dose	Supportive Care	Days
Cyclophosphamide	500 mg/m^2 per day IV infusion over approximately 60 minutes, daily for 3 days.	IV pre-hydration prior to cyclophosphamide with 1 liter of 0.9% Sodium Chloride USP.	-5, -4, -3
Fludarabine	After conclusion of cyclophosphamide infusion, 30 mg/m^2 per day IV infusion in 50 mL of 0.9% sodium chloride over approximately 30 minutes, daily for 3 days.	Post fludarabine infusion, IV hydration with 1 liter of 0.9% Sodium Chloride USP.	-5, -4, -3

Hydration may be adjusted based on clinical need (e.g. over hydration or dehydration). There will be no dose adjustment of chemotherapy agents for weight considerations or abnormal lab values. If subjects are eligible for the trial, then the full dose of lymphodepletion agents will be administered.

5.4 Investigational Agent Administration and Research Procedures

5.4.1 Cell Processing:

Cellular Product: Autologous T cells; transduced with bivalent lentiviral vector (CD19/CD22.BB.z) Chimeric Antigen Receptor (CD19/CD22-CAR); following Fludarabine and Cyclophosphamide

Autologous CD19/CD22-CAR T cells will be generated from fresh or cryopreserved PBMCs under GMP conditions in the Stanford's Laboratory for Cell and Gene Medicine (LCGM) or MBI Sunnyvale Facility. Apheresis products from participating subjects will be received through and final formulated product distributed by the Stanford Bone Marrow Transplant – Cellular Therapeutics Facility (BMT-CTF).

Any prepared cells not required for infusion or for research or regulatory purposes will be cryopreserved by standard techniques and will be made available should the subject be

eligible for a second infusion as outlined in section **5.5**. Additional product in excess of that needed for infusion will be utilized for research.

Fresh or cryopreserved peripheral blood mononuclear cells (PBMC) (depending on the timing of apheresis relative to cell culture, subject condition and scheduling availability) will be enriched for T cells using a CD4/CD8 immunomagnetic bead enrichment at the Stanford's Stem Cell and Cellular Therapeutics Operations. They will then be activated by co-culture with immunomagnetic particles expressing anti-CD3 and anti-CD28 mAb. One day following activation, replication incompetent lentiviral vector particles containing the CD19/CD22.BB.z construct will be added to the culture for transduction. Cells will be incubated for approximately 7 days with IL-2 then harvested and cryopreserved for subsequent infusion. Cultures may be extended as needed for adequate cell growth. Cells will be required to meet standard release criteria including transduction efficiency ≥ 10%, T cell content ≥ 70%, sterility and minimum levels of LPS as well as no evidence for replication competent lentivirus. All procedures will take place using good manufacturing process guidelines.

The release criteria will be based upon analyses for each dose of CD19/CD22-CAR lenti-transduced autologous PBL and will include:

Test	Criteria
Cell viability[1]	≥ 70%
Cell number[1]	within 20% of planned dose level
% CAR+ cells[1]	≥ 10%
Endotoxin[1]	≤ 0.5 EU/mL
Mycoplasma[1]	≤ 10 CFU/mL
RCL[4]	Negative
Sterility testing[3]	Negative

[1]Performed on sample from final product immediately prior to infusion, results available at the time of infusion.

[3]Gram stain is performed on final product prior to infusion and is available at the time of infusion, cultures will be sent from the harvest (Day 7 or Day 13) product.

[4] Sample collected from the final product prior to infusion. Results will not be available before cells are infused into the subject.

5.4.1.1 Determination of product administration: fresh or cryopreserved

CD19/CD22-CAR T cells will be administered on Day 0. Cells may be cryopreserved after harvest according to Standard Operating Procedures in LCGM or MBI Sunnyvale Facility, formulated in CryoStor® CS5 (BioLife Solutions) in an appropriately-sized CryoMACS Freezing Bag, or administered fresh (depending on cell culture growth, patient condition, etc). If cells are cryopreserved, infusion will be scheduled when final sterility culture results are available unless the subject's clinical condition requires immediate intervention. In addition, unavoidable delays to the infusion date may include any of the following:

- Subject develops a health condition or infection prior to cell administration, such

that the infusion criteria in Section 5.4.2.1 cannot be met; or

- Schedule conflict arises (patient or patient's family, intervening holidays, within LCGM or MBI, etc.)

5.4.2 CD19/CD22-CAR T cell Infusion

5.4.2.1 Cell Infusion Criteria

Subjects will be hospitalized to receive treatment with CD19/CD22-CAR T cells and will remain hospitalized for a minimum of 7 days during the dose escalation trial phase. Beyond 7 days, patients will be discharged once all AEs have resolved to Grade 1 or better, and at the discretion of the treating physician. Subjects may be discharged with non-critical and clinically stable or slowly improving toxicities (e.g., renal insufficiency, cytopenias) even if > Grade 1, if deemed appropriate by the investigator. Subjects must meet the following criteria in order for cells to be infused (based on labs obtained within 24 hrs of cell infusion):

- CD19/CD22-CAR T cells must have met release criteria (section 5.4.1)
- Subject has no evidence of hemodynamic instability
- Subject has not developed a new requirement for supplemental oxygen therapy
- Subject has not developed symptoms concerning for new, systemic infection or condition that in the opinion of the PI may pose an unacceptable risk to the subject
- There is no evidence of clinically significant cardiac dysfunction, uncontrolled, significant tumor lysis syndrome, serum creatinine > 2 x ULN, and no acute neurological toxicity > Grade 1 (with the exception of peripheral sensory neuropathy).
- Subject must not be receiving systemic anti-microbials for the treatment of an active infection within 48 hours before CD19/CD22-CAR T cell administration (prophylactic use of anti-microbials are allowed). **Corticosteroid therapy at a pharmacologic dose (≥ 5 mg/day of prednisone or equivalent doses of other corticosteroids) and other immunosuppressive drugs must be avoided for 5 days prior to CD19/CD22-CAR T cell administration**.

If these criteria are not met, measures will be taken to resolve the underlying condition(s) and if successful cells may be infused up to 72 hours following the time of the planned infusion (if administering fresh cells). Otherwise, cells may be frozen and infused upon subject recovery.

Should an event not meet these criteria immediately prior to receiving CD19/CD22-CAR T cells, the cell infusion must be delayed until the event resolves. If the CD19/CD22-CAR T cells infusion is delayed > 2 weeks, conditioning chemotherapy must be repeated**, unless investigator deems this unnecessary**.

5.4.2.2 Premedications

All subjects will receive levetiracetam 10 mg/kg/dose orally twice a day (maximum dose of 750 mg/dose) beginning the day before cell infusion (Day -1).

Subjects at high risk of TLS, per investigator's discretion, will receive allopurinol 300 mg

orally once daily beginning the day prior to cell infusion (Day -1).

Subjects will receive the following medications 30-120 minutes prior to cell infusion:

- o Diphenhydramine 25-50 mg/dose PO or IV;
- o Acetaminophen 650 mg/dose PO.

5.4.2.3 Cell Infusion

Cells are delivered to the subject care unit by a staff member from BMT-CTF. Prior to infusion, the cell product identity label is double-checked by two authorized staff (MD or RN), an identification of the product and documentation of administration are entered in the subject's chart, as is done for blood banking protocols. Cell products should NOT be infused unless the product identification matches the subject's identification.

Cells are to be infused intravenously (IV) over 10-30 minutes (or as tolerated based on volume and/or DMSO toxicity) via non-filtered tubing, gently agitating the bag during infusion to prevent cell clumping. After infusing cells, rinse the infusion tubing and empty bag with a backflush of normal saline. Infuse the remaining cells using the backflush method. Documentation in the medical record should include the volume of cell infusion, the thaw start/stop time (if cells are cryopreserved), and cell product infusion time start/stop times.

5.5 EVALUATIONS AND FOLLOW UP

Subjects will be hospitalized prior to receipt of CD19/CD22-CAR T cells and will remain in the hospital for a minimum of 7 days during the dose escalation trial phase. Beyond 7 days, patients will be discharged once all AEs have resolved to Grade 1 or better. Subjects should remain hospitalized for ongoing cell-related fever, hypotension, hypoxia, or ongoing central neurological toxicity > Grade 1, or if deemed necessary by the treating investigator. Subjects may be discharged with non-critical and clinically stable or slowly improving toxicities (e.g., renal insufficiency, cytopenias) even if > Grade 1, if deemed appropriate by the investigator.

Given the possibility that a subject could develop CRS or neurotoxicity after discharge from the hospital, subjects will be asked to remain within commuting distance to the clinic for the first 28 days post cell infusion, and subjects and their family members/caregivers will be educated on potential symptoms such as fever, dyspnea, confusion, aphasia, dysphasia, somnolence, encephalopathy, ataxia, or tremor. If subjects develop these symptoms, they will be instructed to immediately contact the investigator or seek immediate medical attention.

During this post cell infusion period, the following procedures will be completed at the time points outlined in the Section **9** Study Calendar:

5.5.1 Evaluation Prior to Cell infusion

The following procedures will be completed prior to cell infusion:

Within 24 hours of CAR Infusion (unless otherwise specified):

- Complete physical exam, neurologic examination, weight and vital signs

- Laboratory evaluations
    - Chemistry Panel: (sodium, potassium, chloride, bicarbonate, BUN, creatinine, glucose, calcium, AST and/or ALT, alkaline phosphatase, bilirubin, albumin, total protein)
    - Magnesium
    - CBC with differential
    - CRP
    - TSH (within 28 days of lymphodepletion)
    - Urinalysis
- RCL blood sample (baseline measure, may be drawn any time prior to cell infusion)
- Correlative samples as outlined in Section **14.9**, Appendix I.
- Adverse/Serious Adverse Event reporting
- Concomitant medications documentation

5.5.2 Required monitoring during cell infusion:

Monitoring will include vital signs (temperature, blood pressure, heart rate, oxygen saturation, respiratory rate) prior to infusion, within 15 minutes after start of the infusion, and then 30 (± 10 min), 60 (± 10 min) and 120 (± 10 min) minutes after infusion.

- ✓ Supplemental oxygen will be available at the bedside.
- ✓ If an allergic or other acute reaction occurs, studies appropriate for investigation of a transfusion reaction will be performed (urinalysis, CBC, Coomb's test). Acute reactions will be treated according to institutional standards of care.

5.5.3 Evaluations Post cell Infusion until Day 27 (Day 0 to Day 27)

After completing CD19/CD22-CAR T cell infusion all subjects will be followed in the post treatment assessment period. Counting from Day 0 (CD19/CD22-CAR T cell infusion), subjects will undergo the following evaluations (during hospitalization or will return to the clinic), Daily until Day 14 and then twice per week until Day 27 (± 4 days):

- Brief targeted physical exam, as clinically indicated
- Neurologic exam
- Vital signs including blood pressure, heart rate, oxygen saturation, respiratory rate and temperature, every 6-8 hours during hospitalization, then with each visit after discharge
- ICE score evaluated every 8 hours (± 2 hours) and with any change in neurologic functioning during the initial hospitalization for cell infusion or daily after discharge until Day 14
- Labs performed daily until day 14 (±2 days) (unless otherwise specified) and then twice weekly or weekly until Day 27 (± 4 days) or as clinically indicated
    - Chemistry Panel (sodium, potassium, chloride, bicarbonate, BUN, creatinine, glucose, calcium, AST and/or ALT, alkaline phosphatase, bilirubin, albumin, total protein)

- CBC with differential
- Phosphorus, magnesium, uric acid, and LDH (daily while hospitalized and then day 14 and 21)
- CRP (daily while hospitalized and then day 14 and 21)
- IgG (every 3 to 12 weeks post infusion until Month 3 [± 2 weeks])

- Correlative samples as outlined in section **14.9**, Appendix I.
- Lymph node biopsy for patients with DLBCL for correlative studies performed once between Day 7 and Day 28 and at time of progression, if clinically feasible, in patients with lymphoma.
- Adverse/Serious Adverse Event reporting
- Concomitant medications documentation

Monitoring of CRP and LDH (only if LDH is elevated at baseline) levels may assist with the diagnosis and define the clinical course in regards to CRS/neurotoxicity. It is, therefore, recommended that CRP and LDH (if elevated at baseline) be monitored when lab specimens are drawn starting at Day 0 and continuing through hospitalization. In addition, lactate can be monitored as clinically indicated.

5.5.4 Evaluation on Day 28 (+/- 4 days)

All subjects will undergo the following evaluations on Day 28. Day 28 will be the primary evaluation time point for the secondary efficacy objective of subjects with ALL. Day 28 evaluation will NOT be the primary assessment time point for efficacy in subjects with DLBCL; secondary efficacy objective for subjects with DLCBL will be evaluated at the Month 3 evaluation.

- Physical exam
- Neurologic exam, performance status, weight, and vital signs (blood pressure, heart rate, oxygen saturation, respiratory rate and temperature)
- Labs
  - Chemistry Panel (sodium, potassium, chloride, bicarbonate, BUN, creatinine, glucose, calcium, AST and/or ALT, alkaline phosphatase, bilirubin, albumin, total protein)
  - CBC with differential
  - CRP and ferritin
- Correlative samples as outlined in section **14.9**, Appendix I.
- Adverse/Serious Adverse Event reporting
- Concomitant medications documentation
- Disease evaluations will be specific to the subject's type and location of disease and may include imaging studies, LP or bone marrow aspirate:
  - Imaging Studies if any are relevant to the subject's site of disease such as CT, PET, MRI, x-rays;
  - Lumbar puncture for collection of CSF samples at discretion of investigator for all subjects with CSF involvement.

- o Bone marrow aspirate: bone marrow aspiration will be requested for subjects presenting with bone marrow involvement prior to therapy, and/or if new abnormalities in the peripheral blood counts or blood smear cause clinical suspicion of bone marrow involvement after treatment.
- o Lymph node biopsy for diagnosis at time of progression in patients with lymphoma, if clinically feasible, a portion of the sample will be sent for correlative studies

On Day 28 (± 7 days), subjects may undergo a small volume apheresis (approx. one to two blood volumes) to obtain peripheral blood lymphocytes for correlative studies. Every attempt will be made to use an indwelling catheter or peripheral lines for the small volume apheresis. If this is not feasible and an apheresis specimen cannot be collected, peripheral blood should be collected into the appropriate tubes for correlative samples.

5.5.5 Evaluations of subjects who Do NOT received CD19/CD22-CAR T cells

The following procedures/assessments will be completed for subjects who are enrolled but do not receive CD19/CD22-CAR T cells, at the time points outlined in the Section **9** Study Calendar until disease progression and/or toxicities attributable to the study participation resolve to ≤ Grade 1 or stabilize:

- Disease assessment per standard of care
- Adverse/Serious Adverse Event reporting and concomitant medication documentation until 30 days after last procedure (e.g., leukapheresis, conditioning chemotherapy).

Should the subject fail to return to the clinic for a scheduled protocol specific visit, sites will need to make 2 attempts by a combination of telephone and mail to contact the subject. Sites must document both attempts to contact the subject. If a subject does not respond within 1 month after the second contact the subject will be considered lost to follow-up and no additional contact will be required.

5.5.6 Post Treatment Assessment Period (after Day 28) after CD19/CD22-CAR T cells

5.5.6.1 Schedule

After Day 28, subjects who received CD19/CD22-CAR T cells will return to the clinic at the following intervals:

- Month 2 (± 1 week)
- Month 3 (± 1 week) (Efficacy evaluation in subjects with DLBCL who received CD19/CD22-CAR T cells)

Should a subject fail to return to the clinic for a scheduled protocol specific visit, sites will need to make 2 attempts by a combination of telephone and mail to contact the subject. Sites must document both attempts to contact the subject. If a subject does not respond within 1 month after the second contact the subject will be considered lost to follow-up and no additional contact will be required. Should a subject proceed to other therapies,

the patient would undergo a final evaluation for the study and proceed to long term follow up of gene therapy per section 5.5.7, at the discretion of the principal investigator. Additional correlative sample collection is at the discretion of the principal investigator.

5.5.6.2 Evaluations

The following procedures will be completed for subjects as outlined in the Section **9** Study Calendar:

- Physical and neurological exams, vital signs (blood pressure, heart rate, respiratory rate, oxygen saturation, temperature, weight) and performance status
- Laboratory evaluations
    - Chemistry Panel (sodium, potassium, chloride, bicarbonate, BUN, creatinine, glucose, calcium, AST and/or ALT, alkaline phosphatase, bilirubin, albumin, total protein)
    - CBC with differential
    - IgG (every 3 to 12 weeks post infusion until Month 3 [± 2 weeks]).
- Disease evaluations will occur only on Month 3 visit and be specific to the subject's type and location of disease and may include imaging studies, LP or bone marrow aspirate/biopsy:
    - Imaging Studies if any are relevant to the subject's site of disease such as CT, PET, MRI, x-rays; all subjects with lymphoma will undergo PET/CT.
    - Lumbar puncture for collection of CSF samples at discretion of investigator for all subjects with CSF involvement.
    - Bone marrow aspirate: will be requested for subjects presenting with bone marrow involvement prior to therapy, and/or if new abnormalities in the peripheral blood counts or blood smear cause clinical suspicion of bone marrow involvement after treatment.
    - Lymph node biopsy for diagnosis at time of progression in patients with lymphoma, a portion of the sample will be sent for correlative studies
- RCL blood sample at Month 3 visit only, sent to Indiana University Gene Therapy Testing Laboratory
- Correlative samples as outlined in section **14.9**, Appendix I.

  At Month 3 (± 1 month), subjects may undergo a small volume apheresis (approx. one to two blood volumes) to obtain peripheral blood lymphocytes for correlative studies. Every attempt will be made to use an indwelling catheter or peripheral lines for the small volume apheresis. If this is not feasible and an apheresis specimen cannot be collected, peripheral blood should be collected into the appropriate tubes for correlative samples.

- Adverse/Serious Adverse Event reporting
- Concomitant medications documentation

5.5.7 Long Term Follow-up Period

At any time during the post treatment assessment period, if a subject did not respond to treatment (i.e., did not achieve a CR or PR) or progresses following a response and is

either not eligible for re-treatment or chooses not to pursue re-treatment or proceeds to alternative therapy, the subject will proceed directly to the Month 3 visit and subsequently be followed in Long Term Follow Up for survival, subsequent therapy, long term gene therapy follow up, and at the discretion of the investigator, completion of correlative sample collection. A PMBC sample (for anti-CD19/CD22-CAR T cells, etc) and lymph node biopsy should be collected at the time of progression, prior to starting any subsequent anticancer therapy, at the discretion of the principal investigator.

5.5.7.1 Long Term Follow-up Schedule

Subjects who received CD19/CD22-CAR T cells will begin the long term follow-up period after they have completed the Month 3 visit (whether they have responded to treatment or went straight to the Month 3 visit due to disease progression)

- Every 3 months (± 1 month) through Month 12
- Every 6 – 12 months (± 2 months) for 5 years
- Beginning with year 6 (± 3 months), subjects will return to the clinic or be contacted by phone, e-mail or mail one time annually for up to 15 years.

5.5.7.2 Evaluations

The following procedures will be completed for all subjects who are enrolled and received CD19/CD22-CAR T cells, at the time points outlined in section **5.5.7.1** and section **9** Study Calendar:

- Physical exams to include vital signs and performance status
    - ✓ For Gene Therapy annual evaluations should specifically document any new malignancies, new incidence or exacerbation of a pre-existing neurologic disorder, new incidence or exacerbation of a prior rheumatologic or other autoimmune disorder, new incidence of a hematologic disorder; and other factors that may be relevant to the feasibility and scientific value of the long-term follow up observations (i.e. exposure to other cancer causing agents).
- Disease evaluations will be performed if applicable (i.e. patient responding to protocol therapy) and may be performed by referring physician. Evaluations will be specific to the subject's type and location of disease and may include imaging studies, LP or bone marrow aspirate:
    - o Imaging Studies if any are relevant to the subject's site of disease such as CT, PET, MRI, x-rays; all subjects with lymphoma will undergo PET/CT.
    - o Lumbar puncture for collection of CSF samples at discretion of investigator for all subjects with CSF involvement.
    - o Bone marrow aspirate: will be requested for subjects presenting with bone marrow involvement prior to therapy, and/or if new abnormalities in the peripheral blood counts or blood smear cause clinical suspicion of bone marrow involvement after treatment.
    - o Lymph node biopsy for diagnosis at time of progression, a portion of the sample will be sent for correlative studies

If subject's disease has not progressed by Month 24, disease assessments will continue to be performed per standard of care.

- RCL blood sample at Month 6 (± 1 month) and 12 (± 2 months), sent to Indiana University Gene Therapy Testing Laboratory
    - After month 12, RCL blood samples will be collected annually for 5 years and banked if all RCL tests in first year were negative
- Laboratory evaluations
    - CBC with differential
    - Correlative studies as per section **14.9**, Appendix I

      At 3 (± 1 month), 6 (± 1 month), 9 (± 1 month) and 12 (± 2 months) months subjects may undergo a small volume apheresis (approx. one to two blood volumes) to obtain peripheral blood lymphocytes for correlative studies. Every attempt will be made to use an indwelling catheter or peripheral lines for the small volume apheresis. If this is not feasible and an apheresis specimen cannot be collected, peripheral blood should be collected into the appropriate tubes for correlative samples.
    - Lymph node biopsy for correlative studies to be performed at time of progression (this biopsy is conducted for diagnostic purposes related to progression with sample sent for research).
- Targeted Adverse/Serious Adverse Event reporting (for 24 months, until disease progression, or until subject proceeds to alternate therapy; whichever occurs first)
    - Including neurological, hematological, infections, autoimmune disorders, and secondary malignancies
- Targeted concomitant medication documentation (for 24 months, until disease progression, or until subject proceeds to alternate therapy; whichever occurs first)
    - Including gammaglobulins, immunosuppressive drugs, anti-infectives, and vaccinations

A PMBC sample (for CD19/CD22-CAR T cells, etc) and lymph node biopsy (in patients with lymphoma) should be collected at the time of progression, prior to starting any subsequent anticancer therapy.

5.5.7.3 Annual Questionnaire Follow Up (after 5 years)

Once a subject has completed 5 years of follow up without evidence of delayed adverse events secondary to genetically engineered cellular therapy product, the following evaluations will be conducted annually for the subsequent 10 years (± 2 months):

The subject will be contacted (in clinic or via phone or written questionnaire) to evaluate for development of delayed adverse events (See questionnaire in **Section 14.7,** Appendix G).

- If vector modified cells were detected in the blood during the previous visit, then blood for persistence of vector modified cells will be collected and tested until negative.
- Subjects are requested to inform the study research team of any changes to e-mail (if consented to use e-mail), phone, and address. E-mails will be sent to subjects via the Secure Email to ensure securely and confidentially over an SSL/encrypted connection.
- Distribution and collection of questionnaires
  - A draft letter to subject's primary doctor is provided in section **14.8,** Appendix H. This letter should be sent to the local health care provider via mail, fax or e-mail at the time the subject is referred back to the care of the local physician or with any change in primary doctors. E-mails will be sent to subjects via the Secure Email to ensure securely and confidentially over an SSL/encrypted connection.
  - Subjects will be sent a request for information (section **14.7**, Appendix G) and questionnaire annually.
    - If there is no response within 1 month, the study coordinator will follow up with a telephone call to request the information. The questionnaire may be completed with the subject responses over the phone at this time.
    - If there is no response to the telephone call, the letter and questionnaire (section **14.7**, Appendix G) should be sent to the subject via FedEx, signature required.
    - Each attempt to contact the subject and outcome must be documented in the medical record.

5.6 OPTION FOR ADDITIONAL DOSE(S) OF CD19/CD22-CAR T CELLS

On the day cells are infused, remaining cells that have been produced above and beyond the number of cells needed for a subject's dose level will be cryopreserved using standard techniques. Cells will be reserved for 2 or 3 additional doses and remaining cells will be released for research. Subjects will have the option for additional infusions of CD19/CD22-CAR T cells (including preparative chemotherapy regimen, toxicity assessment and research blood sampling) if the following criteria are met:

5.6.1 Eligibility Criteria for subsequent cell infusions:

- ✓ Cell infusion of CD19/CD22-CAR T cells has been deemed safe (enrollment to the first dose cohort (1 x 10^6 cells/kg) dose cohort has been completed without 2 subjects experiencing DLTs, and the resulting safety data has been submitted to the FDA for review.
- ✓ Response to previous infusion. Subjects who had a PR, or SD with clinical benefit may elect to receive another infusion of cells. Subjects that initially

had a CR may only receive a second dose if evaluable disease recurs. Clinical benefit is indicated by an improvement in the subject's health status (e.g., decreased transfusion requirement, improved cytopenias, decrease in number of blasts not sufficient to reach a PR, improved performance status or quality of life, etc.).

- ✓ At least 60 days have passed since the previous cell infusion.
- ✓ Circulating levels of CD19/CD22-CAR T cells must be < 5% by flow cytometry.
- ✓ Any toxicity (regardless of causality) after the previous CD19/CD22-CAR T cell infusion must resolve such that subjects meet all the initial eligibility criteria as outlined in sections **3.1** and **3.2**.
- ✓ An adequate number of cryopreserved CD19/CD22-CAR T cells or an adequate number of apheresis product sufficient to generate an additional dose of CD19/CD22-CAR T cells must be available.

The cell dose (based on CAR transduced cells) for the second infusion shall not be greater than the current dose level completed or the MTD if this has been determined.

As a rule, subjects who incurred DLT after receiving the first cell infusion will not be eligible to receive additional cell infusions unless IRB, APB and FDA approval is granted on a case by case basis.

5.6.2 Procedures for Additional Doses

- Subjects may receive additional antineoplastic and lymphodepleting chemotherapy prior to the second infusion of CD19/CD22-CAR T cells.
- On the day of infusion, the cellular product will be thawed and administered IV immediately (as per institutional guidelines) at a rate of approximately 10-15 ml/min or as tolerated based on volume status and/or DMSO toxicity.
- Any subject who receives subsequent doses of CD19/CD22-CAR T cells will NOT be evaluable for toxicity purposes of this study as they will be beyond the 28 day observation period for DLTs after their first cell infusion. However post-infusion monitoring will be the same as for the 1st infusion and all toxicities, including secondary reactions, will be recorded and reported. If two or more subjects develop Grade 4 toxicity at any time following the second CD19/CD22-CAR T cell infusion that is felt to be possibly, probably or likely related to the CD19/CD22-CAR T cells, then second infusions will be paused pending discussion with the FDA, IRB and APB regarding continuing second infusions as part of the experimental regimen.

5.6.2.1 Gene Therapy Follow Up

If a subject receives more than one cellular protocol, the timing of gene therapy long term evaluations is restarted with each subsequent gene therapy administration. For example, a subject receives gene therapy and has undergone long term follow up for 1.5 years, develops progressive disease and is treated with a second gene therapy product. That subject

will begin the blood sample collection at 3, 6 and 12 months (± 1 month) post cell administration and continue for a total of 15 years of follow up from the LAST gene therapy administration.

5.7 Phase 1 Dosing Procedures

Adverse events that are considered disease-related (not suspected of relationship to CD19/CD22-CAR T cells) will not be considered dose-limiting toxicities. Only those AEs suspected to be related to pre-infusion chemotherapy and/or CD19/CD22-CAR T cells (any component of the treatment regimen) will be used in the definition of DLT. Toxicities occurring after initiation of the chemotherapy preparative regimen but prior to CD19/CD22-CAR T cell infusion, will primarily be attributable to the chemotherapy administration or disease, if not extraneous causes. After cell infusion, toxicities will be evaluated for temporal and causal relationship to chemotherapy versus cell infusion. Some symptoms may overlap and attribution will not be clearly definable, in which case, toxicities will be attributed as possibly related to both preparative regimen and cell infusion. Toxicities will be attributed to the T cells if: 1) they were NOT present before T cell infusion; OR 2) they increase in grade in temporal association with the T cell infusion; AND 3) they are not clearly explained by other factors.

Subjects who are fully assessable are those that have completed the conditioning lymphodepletion chemotherapy regimen and received the CD19/CD22-CAR T cell infusion. Chemotherapy-related toxicities experienced by subjects who are unable to receive CD19/CD22-CAR T cells (See section **13.7.2** for the definition of inevaluable subjects) *will not* be considered in the definition of DLT.

See section **7.2** Causality for definitions of 'suspected'. The definition of DLT in these studies uses NCI's Common Terminology Criteria for Adverse Events (CTCAEv4.03).

5.7.1 Definition of DLT:

Adverse events that are at least possibly related to the treatment regimen (preparative chemotherapy and/or CD19/CD22-CAR T cells) with onset within the first 28 days following CD19/CD22-CAR T cell infusion will be considered DLTs as follows:

- CRS toxicity Grade 4 in severity, or Grade 3 in severity for greater than 7 days.
- Infusion reactions ≥ Grade 2 in severity lasting more than 24 hours.
- Grade 4 neurotoxicity or neurotoxicity Grade 3 for greater than 7 days (severity as defined in **Section 14.2.4** Neurologic Recommended Treatments by Grade).
- Any other Grade 3 or greater, non-hematological toxicity will be considered a DLT with the following **exceptions**:
  - Tumor lysis syndrome, including associated abnormalities (e.g., electrolytes, uric acid, renal function);
  - Grade 3 diarrhea that resolves to ≤ Grade 2 within 4 days;
  - Grade 3 low electrolyte levels that are correctable and asymptomatic, Grade 3 hypoalbuminemia;

        - Hypocalcemia toxicity grade should be assigned based on the calcium level corrected for degree of hypoalbuminemia according to the following (or comparable) formula: For every albumin decrease of 1 gm/dL a total calcium increase of 0.2 mmol/L is to be made;
    - Abnormal coagulation parameters in subjects on anticoagulant therapy or with pre-existing coagulopathy;
    - Grade 3 transaminase, alkaline phosphatase, bilirubin or other liver function test elevation, provided there is resolution to ≤ Grade 2 or baseline within 14 days. Grade 4 transaminitis lasting < 72 hours will also not be considered DLT;
    - Grade 3 or 4 fever lasting ≤ 7 days, as this is common and expected with CAR therapy and may be a biomarker for an effective immunotherapeutic regimen;
    - Grade 3 or 4 infection or neutropenic fever unless subjects have normal blood counts at baseline, and infection is not considered likely related to cyclophosphamide/fludarabine and relationship to CD19/CD22-CAR T cells is suspected (Note: Grade 4 infection uncontrolled for > 7 days will be considered DLT.);
    - Toxicities occurring within 24 hours post cell infusion related to cell infusion, (including Grade 3 and 4 allergic reaction) that are reversible to a Grade 2 or less within 8 hours with up to two doses of acetaminophen 15 mg/kg/dose (to a max adult dose of 650 mg) or up to two doses of diphenhydramine 1 mg/kg up (to a max adult dose of 50 mg);
    - Grade 3 nausea and/or anorexia.
- A Grade 2 non-hematologic toxicity of > 30 days duration will constitute a DLT (with the exceptions noted above).
- In patients with history of prior SCT, any histologically proven acute GVHD Grade 3 or higher within 30 days of receiving the CD19/CD22-CAR T cells will be considered DLT.

As noted in section **2.5.4** CRS will be graded according to a revised grading system (Lee 2014). See section **14.3** Appendix C. Adverse events attributed to CRS will be mapped to the overall CRS grading assessment for the determination of DLT.

5.7.2 Definition of Maximal Tolerated Dose (MTD)

The MTD is a dose level immediately below the level at which the enrollment is stopped due to a DLT, as explained specifically below:

- If **more than one subject** in the first three subjects included in a dose level experience DLT as defined above, MTD will have been exceeded.
- If DLT develops in **one** of the 3 subjects included in a cohort, the cohort will be then expanded up to six:

- If 2 or more of these 6 included subjects develop DLT, the MTD will have been exceeded.

If no additional subject develops a DLT, the MTD will not have been exceeded and the next dose level can be administered after the 28-day safety assessment period of the last subject at this dose level.

If MTD is exceeded at any dose level, three subjects will be added to the immediate lower dose level, unless it has been previously expanded to six. If less than 2 of 6 subjects develop DLT at that level, it will be defined as the MTD.

5.7.3 Dose Escalation

Cell dose will be body weight-based. In subjects with BMI equal to or less than 30, the dose of CD19/CD22-transduced T cells for each cohort will be based on doses calculated using actual body weight. Subjects with BMI greater than 30 may have doses calculated using ideal body weight (IBW) plus 50% of the difference between actual weight and IBW (see section **14.4**, Appendix D for calculation).

There will be a Phase 1 dose-escalation design with three dose cohorts. The number of CD19/CD22-CAR 1s transferred for each dose level cohort will be:

**Dose Escalation Schedule**	
**Dose Level**	**Dose of CD19/CD22-CAR T cells**
Cohort Level -1	3×10^5 transduced T cells/kg (± 20%)
Cohort Level 1	1×10^6 transduced T cells/kg (± 20%)
Cohort Level 2	3×10^6 transduced T cells/kg (± 20%)
Cohort Level 3	1×10^7 transduced T cells/kg (± 20%)

Each dose cohort will initially include a minimum of 3 subjects. When the cell culture duration is reduced from 9 days to 7 days, 3 additional subjects will be enrolled to the currently enrolling dose level to evaluate safety of the younger cell product, with 14 days between each subject infusion to evaluate safety.

A four (4) week (28 days) safety assessment period will follow regimen completion (defined as infusion of CD19/CD22-CAR T cells) of the first subject in each dose cohort. Subsequent subjects in that cohort and subsequent cohorts may be treated after a one week (7 day) safety assessment period. Four weeks (28 days) must elapse after completion of cell infusion in the final subject in each dose cohort to allow for safety assessment before treating subjects on the next higher dose cohort level. Therefore, treatment in the next higher dose cohort will not proceed to a higher dose level until all subjects have been treated in the prior dose cohort and the last subject treated on the completed dose cohort has been observed for at least 28-days after infusion of CD19/CD22-CAR T cells. If more than one DLT occurs in the first dose level, (2 out of 6) the dose will be de-escalated to Dose level -1 (3×10^5 transduced T cells/kg (± 20%)) for safety evaluation.

If cell growth limitations preclude administration of the number of cells targeted for the assigned cohort level, the subject will receive as many cells as possible and the subject will be enrolled in the appropriate cohort for the number of cells infused, allowing for an additional two subjects to be enrolled per cohort due to cell growth limitations. If a DLT occurs in an additional subject (beyond the first 3 enrolled) entered at a lower dose due to cell growth limitations, accrual will continue at the previously planned dose level for subsequent subjects. If a minimum of $1x10^5$/kg CAR-transduced T cells cannot be obtained for infusion, the subject will still receive the cell infusion, as the effective dose of cells is not well defined, and the subject will be evaluable for feasibility but not analyzed for MTD, although toxicities will be assessed and reported separately. If the 3rd dose level is completed without DLT, an MTD may not be determined. This will be considered the 'highest cell dose' studied, and will be the dose level that will be studied further in the expansion cohort. Alternatively, if no toxicity or clinical activity is observed after completion of the 3rd dose cohort, consideration may be given to adding additional dose cohorts in a protocol amendment.

If cell growth limitations preclude administration of the number of cells targeted for the assigned cohort level in 3 subjects (out of 6) in a dose cohort, that dose will be considered not feasible as there will be inadequate number of subjects in that cohort to evaluate safety. Enrollment to that dose, and any higher doses will cease. This will be considered the 'highest cell dose' studied, and will be the dose level that will be studied further in the expansion cohort.

Dose escalation will follow the rules outlined in the Table below.

**Number of Subjects with DLT at a Given Dose Level**	**Escalation Decision Rule**
0 out of 3	Enter up to 3 subjects at the next dose level. If 0 out of 3 occur, dose may escalate.
≥ 2	Dose escalation will be stopped. This dose level will be declared the maximally administered dose (highest dose administered). Up to three (3) additional subjects will be entered at the next lowest dose level if only 3 subjects were treated previously at that dose.
1 out of 3	If DLT develops in the first subject at Dose Level 1, the dose will be de-escalated to Dose Level -1 (3×10^5 transduced T cells/kg (± 20%)), and accrual to Dose Level -1 will proceed to evaluate safety. If DLT develops in any subject at Dose Level -1, accrual will be temporarily stopped while consultation with the NCI IRB and FDA

	occurs.<br><br>If DLT develops in one of the 3 subjects at Dose Levels 2 or 3, the cohort will be expanded to 6 subjects. If no additional subjects develop DLT, MTD will not have been exceeded and the next dose level can be administered after the four week safety assessment period of the last subject at this dose level.<br><br>If 1 or more of this group suffer DLT, then dose escalation is stopped, and this dose is declared the maximally tolerated dose. Up to three (3) additional subjects will be entered at the next lowest dose level if only 3 subjects were treated previously at that dose.
≤ 1 out of 6 at highest dose level below the maximally administered dose	This is the MTD and is generally the recommended phase 2 dose. At least 6 subjects must be entered at this dose level.

5.7.4 Dose Expansion Cohort

To gain further experience with the safety, feasibility and clinical activity of the CD19/CD22-CAR T cells, the MTD (or highest cell dose studied) will be expanded (i.e., will include those subjects treated at MTD during the dose escalation phase) in two groups of subjects (1. Subjects with DLBCL and 2) Subjects with ALL) to obtain additional information regarding the toxicity and efficacy of this investigational therapy.

6 INVESTIGATIONAL AGENT AND COMMERCIAL DRUG INFORMATION

6.1 AUTOLOGOUS T CELLS TRANSDUCED WITH LENTIVIRAL VECTOR (CD19/CD22.BB.Z) CHIMERIC ANTIGEN RECEPTOR (CD19/CD22-CAR); FOLLOWING FLUDARABINE AND CYCLOPHOSPHAMIDE

6.1.1 Description

Cell therapy production will be conducted according to the SOPs in Stanford's Stem Cell and Cellular Therapeutics Operations and must meet the requirements for a GMP facility. The bi-specific CD19/CD22-CAR replication incompetent lentiviral vector has been manufactured by Lentigen Technology Incorporated.

Fresh or cryopreserved peripheral blood mononuclear cells (PBMC) (depending on the timing of apheresis relative to cell culture, subject condition and scheduling availability) will be enriched for T cells using a CD4/CD8 immunomagnetic bead enrichment at Stanford's Stem Cell and Cellular Therapeutics Operations. They will then be activated by co-culture with immunomagnetic particles expressing anti-CD3 and anti-CD28 mAb. One day following activation, replication incompetent lentiviral vector particles containing

the CD19/CD22.BB.z construct will be added to the culture for transduction. Cells will be incubated for approximately 7 days with IL-2 then harvested and administered fresh or frozen for subsequent infusion. Cells will be required to meet standard release criteria including transduction efficiency ≥ 10%, T cell content ≥ 70%, sterility and minimum levels of LPS as well as no evidence for replication competent lentivirus. All procedures will take place using good manufacturing process guidelines.

6.1.2 Stability:

Stability testing is ongoing.

6.1.3 Administration

Guidance on administration of CD19/CD22-CAR T cells is specified in section **5.4.2.**

6.1.4 Toxicities

In addition to standard transfusion types of reactions (chills, fever, rigors), the toxicities specific to CAR T cell therapy are described in detail in section **2.5**. Every effort will be made to mitigate the risk of these toxicities with the intensive monitoring plans outlined in section **5** and the supportive care measures outlined in section **4.2**. With growing experience with CAR therapy world-wide, the risk of severe neurotoxicity is increasingly better recognized—most notable with the recent occurrences of fatal neurotoxicity. With the incorporation of mandatory anti-seizure prophylaxis and prospective monitoring of neurologic function, we plan to rigorously monitor and evaluate for any neurotoxicity, with a plan to treat more severe neurotoxicity with steroids to mitigate the symptoms.

6.2 FLUDARABINE

6.2.1 Description:

(Please refer to package insert for complete product Information) Fludarabine phosphate is a synthetic purine nucleoside that differs from physiologic nucleosides in that the sugar moiety is arabinose instead of ribose or deoxyribose. Fludarabine is a purine antagonist antimetabolite.

6.2.2 How Supplied:

It will be purchased by the institution’s Pharmacy Department from commercial sources. Fludarabine is supplied in a 50 mg vial as a fludarabine phosphate powder in the form of a white, lyophilized solid cake.

6.2.3 Stability:

Following reconstitution with 2 mL of sterile water for injection to a concentration of 25 mg/ml, the solution has a pH of 7.7. The fludarabine powder is stable for at least 18 months at 2-8^{0}C; when reconstituted, fludarabine is stable for at least 16 days at room temperature. Because no preservative is present, reconstituted fludarabine will typically be administered within 8 hours. Specialized references should be consulted for specific compatibility information. Fludarabine is dephosphorylated in serum, transported

intracellularly and converted to the nucleotide fludarabine triphosphate; this 2-fluoro-ara-ATP molecule is thought to be required for the drug's cytotoxic effects. Fludarabine inhibits DNA polymerase, ribonucleotide reductase, DNA primase, and may interfere with chain elongation, and RNA and protein synthesis.

6.2.4 Storage:

Intact vials should be stored refrigerated (2-8°C).

6.2.5 Administration:

Fludarabine is administered as an IV infusion in an appropriate solution over 30 minutes for 3 days: Day -5, -4, -3. To prevent undue toxicity the dose will be based on BSA (30 mg/m^2/dose).

6.2.6 Toxicities:

At doses of 30 mg/m^2/day for 3 days, the primary side effect is myelosuppression; however, thrombocytopenia is responsible for most cases of severe and life-threatening hematologic toxicity. Serious opportunistic infections have occurred in subjects treated with fludarabine. Hemolytic anemia has been reported after one or more courses of fludarabine with or without a prior history of a positive Coomb's test; fatal hemolytic anemia has been reported. In addition, bone marrow fibrosis has been observed after fludarabine therapy. Other common adverse effects include malaise, fever, chills, fatigue, anorexia, nausea and vomiting, and weakness. Irreversible and potentially fatal central nervous system toxicity in the form of progressive encephalopathy, blindness, and coma is only rarely observed at the currently administered doses of fludarabine. More common neurologic side effects at the current doses of fludarabine include weakness, pain, malaise, fatigue, paresthesia, visual or hearing disturbances, and sleep disorders. Adverse respiratory effects of fludarabine include cough, dyspnea, allergic or idiopathic interstitial pneumonitis. Tumor lysis syndrome has been rarely observed in fludarabine treatment of CLL.

6.3 CYCLOPHOSPHAMIDE

(Refer to FDA-approved package insert for complete product information)

6.3.1 Description:

Cyclophosphamide is a nitrogen mustard-derivative alkylating agent. Following conversion to active metabolites in the liver, cyclophosphamide functions as an alkyating agent; the drug also possesses potent immunosuppressive activity. The serum half-life after IV administration ranges from 3-12 hours; the drug and/or its metabolites can be detected in the serum for up to 72 hours after administration.

6.3.2 How Supplied:

Cyclophosphamide will be obtained from commercially available sources by the institution's Pharmacy Department.

6.3.3 Stability:

Following reconstitution as directed with sterile water for injection, cyclophosphamide is stable for 24 hours at room temperature or 6 days when kept at 2-8^{0}C.

6.3.4 Administration:

It will be diluted in an appropriate solution and infused over one hour. The dose will be based on the subject's body weight, at 500 mg/m^2/dose for three days: Day -5, -4, -3.

6.3.5 Toxicities:

Hematologic toxicity occurring with cyclophosphamide usually includes leukopenia and thrombocytopenia. Anorexia, nausea and vomiting, rash and alopecia occur, especially after high-dose cyclophosphamide; diarrhea, hemorrhagic colitis, infertility, and mucosal and oral ulceration have been reported. Sterile hemorrhagic cystitis occurs in about 20% of subjects; severity can range from microscopic hematuria to extensive cystitis with bladder fibrosis. Although the incidence of hemorrhagic cystitis associated with cyclophosphamide appears to be lower than that associated with ifosfamide, mesna (sodium 2-mercaptoethanesulfonate) has been used prophylactically as a uroprotective agent in subjects receiving cyclophosphamide. Prophylactic mesna is not effective in preventing hemorrhagic cystitis in all subjects. Subjects who receive high dose cyclophosphamide may develop interstitial pulmonary fibrosis, which can be fatal. Hyperuricemia due to rapid cellular destruction may occur, particularly in subjects with hematologic malignancy. Hyperuricemia may be minimized by adequate hydration, alkalinization of the urine, and/or administration of allopurinol. If allopurinol is administered, subjects should be watched closely for cyclophosphamide toxicity (due to allopurinal induction of hepatic microsomal enzymes). At high doses, cyclophosphamide can result in a syndrome of inappropriate antidiuretic hormone secretion; hyponatremia with progressive weight gain without edema occurs. At high doses, cyclophosphamide can result in cardiotoxicity. Deaths have occurred from diffuse hemorrhagic myocardial necrosis and from a syndrome of acute myopericarditis; in such cases, congestive heart failure may occur within a few days of the first dose. Other consequences of cyclophosphamide cardiotoxicity include arrhythmias, potentially irreversible cardiomyopathy, and pericarditis. Other reported adverse effects of cyclophosphamide include headache, dizziness, and myxedema; faintness, facial flushing, and diaphoresis have occurred following IV administration. Mesna (sodium 2-mercaptoethanesulphonate; given by IV injection) is a synthetic sulfhydryl compound that can chemically interact with urotoxic metabolites of cyclophosphamide (acrolein and 4-hydroxycyclophosphamide) to decrease the incidence and severity of hemorrhagic cystitis, but is not a required premedication for this study.

6.4 ACETAMINOPHEN (TYLENOL):

Will be given as a pre-medication. This agent will be provided by the institution's pharmacy. Please refer to the package insert for complete pharmaceutical information on this product.

6.5 DIPHENHYDRAMINE (BENADRYL):

Will be given as a pre-medication IV or orally. This agent will be provided by the institution's Pharmacy Department. Please refer to the package insert for complete pharmaceutical information on this product.

6.6 ANTIMICROBIAL PROPHYLAXIS

Subjects will receive appropriate antimicrobial prophylaxis (e.g., Bactrim for PCP and acyclovir for HSV and VZV prophylaxis) during fludarabine/cyclophosphamide treatment and for a minimum of 6 months following treatment. This agent will be provided by the institution's Pharmacy Department. Please refer to the package insert for complete pharmaceutical information on this product.

6.7 LEVETIRACETAM (KEPPRA):

All subjects will receive levetiracetam 500 mg or 750 mg orally twice a day (maximum dose of 750 mg/dose) beginning the day before cell infusion. For cohort 3, at the onset of ≥ Grade 2 neurotoxicity, levetiracetam should be increased from a dose of 750 mg (PO or IV) BID to a dose of 1000mg (PO or IV) BID. Levetiracetam should continue > 750 mg BID thru day 28 once neurotoxicity develops. This agent will be provided by the institution's Pharmacy Department. Please refer to the package insert for complete pharmaceutical information on this product.

6.8 ALLOPURINOL

Will be given at the investigator's discretion as a pre-medication to prevent Tumor Lysis Syndrome dosed 300 mg orally once daily. This agent will be provided by the institution's pharmacy. Please refer to the package insert for complete pharmaceutical information on this product.

7 ADVERSE EVENTS AND REPORTING PROCEDURES

7.1 POTENTIAL ADVERSE EVENTS

7.1.1 CD19/CD22-CAR T cells

Because these cells have not been previously administered in humans, there may be unanticipated adverse events.

Section **2.5** and section **6** discuss the potential risks of this investigational therapy based on previous studies with this or similar preparative regimens or cell products, including the risks of chemotherapy, risk of autoimmunity, risk of neurotoxicity, risk of the gene therapy component, and risk of cytokine storm. Transient, reversible mild effects have been observed with the administration of fresh cells, including chills, fever, rigors, diaphoresis, anorexia, nausea, diarrhea, headache and myalgias. General guidance for treatment of the most common toxicities are included in section **4.2**.

7.1.2 Risk of Apheresis:

Apheresis is a safe procedure that is routinely performed in healthy adults. Participants will be closely monitored and procedures to minimize risks and prevent side effects are incorporated into all aspects of the protocol. The institutions have broad expertise to adequately manage side effects. The potential risks of apheresis in this trial are as follows:

a. The most common side effects of apheresis are pain and bruising at IV sites. A central venous catheter may be required. Possible side effects include pain, bleeding, bruising, infection, thrombosis, vascular perforation.

b. During apheresis, mild side effects from citrate anticoagulant are common and include chills, numbness and tingling ("pins and needles"), anxiety, muscle cramps, and nausea. More serious side effects due to citrate-induced hypocalcemia are uncommon and include low blood pressure, seizures, weakness, and tetany. Citrate reactions rapidly resolve when the collection is slowed down or stopped. Prophylactic IV $CaCl_2$ and $MgSO_4$ infusions may be administered to subjects deemed to be at high risk of citrate toxicity. Risks of parenteral calcium and magnesium include extravasation necrosis and cardiovascular effects including bradycardia and blood pressure changes. However, side effects are unlikely given the low rate of infusion and use of large bore catheters for apheresis.

c. Transient mild thrombocytopenia is common after apheresis, but bleeding is unlikely.

d. Dilutional anemia occurs during apheresis, but this is unlikely to be clinically significant.

e. Side effects of blood draws include pain and bruising, lightheadedness, and rarely, fainting.

7.2 Adverse Event Definitions

7.2.1 Adverse Event

An adverse event is defined as any reaction, side effect, or untoward event that occurs during the course of the clinical trial associated with the use of a drug in humans, whether or not the event is considered related to the treatment or clinically significant. For this study, AEs will include events reported by the subject, as well as clinically significant abnormal findings on physical examination or laboratory evaluation. A new illness, symptom, sign or clinically significant laboratory abnormality or worsening of a pre-existing condition or abnormality is considered an AE. .

All AEs, including clinically significant abnormal findings on laboratory evaluations, regardless of severity, will be followed until return to baseline or stabilization of event. Serious adverse events that occur more than 30 days after the last administration of investigational agent/intervention and have an attribution of at least possibly related to the agent/intervention should be recorded and reported as per this Section.

An abnormal laboratory value will be considered a reportable, recordable AE if the

laboratory abnormality is characterized by any of the following:

- Results in discontinuation from the study therapy
- Is associated with clinical signs or symptoms
- Requires treatment or any other therapeutic intervention
- Is associated with death or another serious adverse event, including hospitalization.
- Is judged by the Investigator to be of significant clinical impact
- Is a hematologic abnormality, including WBCs, hemoglobin, neutrophils, lymphocytes, and platelets that constitutes a change in grade from baseline (i.e. worsens from a baseline Grade 1 to Grade 2, etc)

If any abnormal laboratory result is considered clinically significant, the investigator will provide details about the action taken with respect to the test drug and about the subject's outcome.

7.2.2 Suspected adverse reaction

Suspected adverse reaction means any adverse event for which there is a reasonable possibility that the investigational therapy caused the adverse event. For the purposes of IND safety reporting, 'reasonable possibility' means there is evidence to suggest a causal relationship between the investigational therapy and the adverse event. A suspected adverse reaction implies a lesser degree of certainty about causality than adverse reaction, which means any adverse event caused by a drug.

7.2.3 Unexpected adverse reaction

An adverse event or suspected adverse reaction is considered "unexpected" if it is not listed in the protocol or informed consent documents or is not listed at the specificity or severity that has been observed; or is not consistent with the risk information described in the general investigational plan or elsewhere in the current application. "Unexpected" also refers to adverse events or suspected adverse reactions that are mentioned in the protocol or informed consent documents as occurring with a class of drugs or as anticipated from the pharmacological properties of the drug, but are not specifically mentioned as occurring with the particular drug under investigation.

7.2.4 Serious

An Unanticipated Problem or Protocol Deviation is serious if it meets the definition of a Serious Adverse Event or if it compromises the safety, welfare or rights of subjects or others.

7.2.5 Serious Adverse Event

An adverse event or suspected adverse reaction is considered serious if in the view of the investigator or the sponsor, it results in any of the following:

- Death,
- A life-threatening adverse drug experience

- In-patient hospitalization or prolongation of existing hospitalization [Note: Hospitalizations related to scheduling issues (e.g. evening or weekend evaluations) and convenience considerations do not constitute a serious adverse event.]
- Persistent or significant incapacity or substantial disruption of the ability to conduct normal life functions
- A congenital anomaly/birth defect.
- Important medical events that may not result in death, be life-threatening, or require hospitalization may be considered a serious adverse drug experience when, based upon appropriate medical judgment, they may jeopardize the subject or subject and may require medical or surgical intervention to prevent one of the outcomes listed in this definition.

7.2.6 Disability

A substantial disruption of a person's ability to conduct normal life functions.

7.2.7 Life-threatening adverse drug experience

Any adverse event or suspected adverse reaction that places the subject or subject, in the view of the investigator or sponsor, at immediate risk of death from the reaction as it occurred, i.e., it does not include a reaction that had it occurred in a more severe form, might have caused death.

7.2.8 Protocol Deviation

Any change, divergence, or departure from the IRB-approved research protocol.

7.2.9 Non-compliance

The failure to comply with applicable IRB requirements, FDA or other regulatory requirements for the protection of human research subjects.

7.2.10 Unanticipated Problem

Any incident, experience, or outcome that:

- Is unexpected in terms of nature, severity, or frequency in relation to

  (a) the research risks that are described in the IRB-approved research protocol and informed consent document, or other study documents, and

  (b) the characteristics of the subject population being studied; **AND**
- Is related or possibly related to participation in the research; **AND**
- Suggests that the research places subjects or others at a *greater risk of harm* (including physical, psychological, economic, or social harm) than was previously known or recognized.

7.3 Adverse Event Reporting

Both Serious and Non-Serious Adverse Events will be clearly noted in source documentation and listed on study specific Case Report Forms (CRFs). The Investigator or designee will assess each Adverse Event (AE) to determine whether it is unexpected according to the Informed Consent, Protocol Document, or related to the investigation. All Serious Adverse Events (SAEs) will be tracked until resolution or until 30 days after the last dose of the study treatment, whichever is later.

Adverse events will be recorded and reported from the start of the conditioning lymphodepletion chemotherapy and conclude 30 days after the last dose of study treatment. Prior to start of conditioning lymphodepletion chemotherapy, record only unexpected serious adverse events considered related or possibly related to study procedures. Deaths occurring after the 30 day window due to disease progression or causes unrelated to study interventions will not be recorded as an SAE, but will be documented as a study outcome as per Section 13.5. In the event that a subject requires disease directed therapy after undergoing leukapheresis but prior to commencing the preparative regimen, these adverse events and any adverse events unrelated to participation in this study will not be collected or reported.

In addition, any **suspected** adverse events that occur after 30 days, during the initial 5 years of follow up, will be recorded and reported (section 5.5.6). Suspected adverse events occurring in subjects who received cell therapy that are potentially related to the gene therapy nature of this study will be reported at the time of their occurrence during 15 year follow up (section **5.5.7**).

7.3.1 Stanford Reporting

SAEs CTCAE Grade 3 and above, and all subsequent follow-up reports will be reported to the Stanford Cancer Institute Data and Safety Monitoring Committee (DSMC) and APB using the study specific CRF regardless of the event's relatedness to the investigation. Following review by the DSMC, events meeting the IRB definition of 'Unanticipated Problem' will be reported to the IRB and APB using eProtocol within 10 working days of DSMC review, or within 5 working days for deaths or life-threatening experiences.

7.3.2 Reporting of IND Safety Reports

Only IND Safety Reports that meet the definition of an unanticipated problem will need to be reported to the IRB and APB.

7.4 IND Sponsor Reporting Criteria

The Investigator must **immediately** report to the sponsor, using the mandatory MedWatch form 3500a, any serious adverse event, whether or not considered drug related, including those listed in the protocol or informed consent documents and must include an assessment of whether there is a reasonable possibility that the drug caused the event.

Study endpoints that are serious adverse events (e.g. all-cause mortality) must be reported in accordance with the protocol unless there is evidence suggesting a causal

relationship between the study therapy and the event (e.g. death from anaphylaxis). In that case, the investigator must immediately report the death to the sponsor.

Events will be submitted to Sponsor Dr. Crystal Mackall, at:

Crystal Mackall, M.D.
Professor Pediatrics & Medicine
Associate Director, Stanford Cancer Institute
265 Campus Dr G3141A, MC5456
Stanford, CA 94305
650-725-9670

7.4.1 Reporting Pregnancy

- ✓ Maternal exposure

If a female subject becomes pregnant during the course of the study while she still has detectable CD19/CD22-CAR T cells in peripheral blood, the study treatment should be discontinued immediately and the pregnancy reported to the Sponsor and the Stanford regulatory authorities. The potential risk of exposure of the fetus to the investigational agent(s) or chemotherapy agents (s) should be documented in box B5 of the MedWatch form "Describe Event or Problem".

Pregnancy itself is not regarded as an AE unless there is a suspicion that the study treatment under study may have interfered with the effectiveness of a contraceptive medication. However, as subjects who become pregnant on study risk intrauterine exposure of the fetus to agents which may be teratogenic, the Sponsor is requesting that pregnancy should be reported in an expedited manner as **Grade 3** "***Pregnancy, puerperium and perinatal conditions* - *Other (pregnancy*)**" under the ***Pregnancy, puerperium and perinatal conditions*** SOC.

Congenital abnormalities or birth defects and spontaneous miscarriages should be reported and handled as SAEs. Elective abortions without complications should not be handled as AEs. The outcome of all pregnancies (spontaneous miscarriage, elective termination, ectopic pregnancy, normal birth, or congenital abnormality) should be followed up and documented.

If any pregnancy occurs within 4 months of last dose of conditioning lymphodepletion chemotherapy or while CD19/CD22-CAR T cells are detectable in peripheral blood, then the investigator should inform the Sponsor within 1 day, i.e., immediately, but **no later than 24 hours** of when he or she becomes aware of it.

The designated Sponsor representative will work with the investigator to ensure that all relevant information is provided to the Sponsor within 1 to 5 calendar days for SAEs and within 30 days for all other pregnancies.

The same timelines apply when outcome information is available.

- ✓ Paternal exposure

Male subjects should refrain from fathering a child or donating sperm for 4 months after the last dose of conditioning lymphodepletion chemotherapy and while CD19/CD22-CAR

T cells are detectable in peripheral blood.

Pregnancy of the subject's partner is not considered to be an AE. However, the outcome of all pregnancies (spontaneous miscarriage, elective termination, ectopic pregnancy, normal birth, or congenital abnormality) occurring from the date of the first dose until 4 months after the last dose should, if possible, be followed up and documented.

7.5 FDA Reporting Criteria

7.5.1 IND Safety Reports to the FDA (Refer to 21 CFR 312.32)

The Sponsor will be responsible for reporting to the FDA any unexpected fatal or life-threatening suspected adverse reactions as soon as possible but no later than 7 calendar days of initial receipt of the information using the MedWatch Form 3500a. All CRS events ≥ Grade 3 in severity and all neurotoxicity events ≥ Grade 3 in severity will be submitted to IND 017484 on a MedWatch Form 3500A in an expedited fashion.

7.5.2 FDA Annual Reports (Refer to 21 CFR 312.33)

The Sponsor will submit the Annual Report to the FDA according to 21CFR§312.33, and any associated FDA correspondences regarding the IND annual report.

7.5.3 Serious Adverse Event Reporting on Cell Therapy Products to the FDA

A sample from all products that are non-conforming or do not meet release specifications will be used to conduct an out of specification investigation and the remainder either disposed of according to our facility biohazardous material disposal SOP or the FDA will be contacted by the manufacturing team to determine whether the product is suitable for infusion. The manufacturing facility will report all products manufactured including those that did not meet release criteria or were otherwise not infused in the annual IND report to the FDA.

All HCT/P deviations involving 351 cell products will be reported using MedWatch From FDA3500A according to FDA publication "Guidance for Industry: MedWatch Form FDA 3500A: Mandatory Reporting of Adverse Reactions Related to Human Cells, Tissues, and Cellular and Tissue-Based Products (HCT/Ps) available at: http://www.fda.gov/BiologicsBloodVaccines/GuidanceComplianceRegulatoryInformation/Guidances/Tissue/ucm074000.htm.

7.5.4 Action Plan for Positive Results on Cell Product Safety Testing:

In the unlikely event that a positive sterility test or mycoplasma test result is obtained after distribution of a cell product that has been administered fresh (not cryopreserved), or after administration of the product to the subject, the following steps will be initiated IMMEDIATELY:

a. Clinical Laboratory personnel will notify the manufacturing facility of the test results.
b. The manufacturing facility personnel will notify the Principal Investigator at 650-723-0822. The Sponsor will also be immediately notified. Both will be updated with any

substantive changes, including the final report on the identification and sensitivity from the positive sterility test. The manufacturing facility QS Director or designee will determine the need for quality improvement based on the nature and extent of the incident.

c. If the manufacturing facility is unable to reach Investigator within 15 minutes, contact inpatient attending physician caring for the subject on the hospital service via hospital page with direct communication.. NOTE: The Sponsor; Principal Investigator; and/or designee will contact the attending physician, who will determine the extent of the work-up of a positive culture in consultation with appropriate infectious disease consultants, as well as determine an appropriate action treatment plan.

d. The Investigator/attending physician will discuss the positive results with the subject, and specify the clinical therapy, antibiotic regimen and/or monitoring plan.

e. A contaminated sample of a product that has been administered to a subject will be handled in the same fashion as a Grade 4/5 toxicity. The Principal Investigator will be responsible for notifying the IRB and APB via an Unanticipated Problem (UP) report within 5 working days, and the Sponsor will notify the FDA via an expedited 7-day IND Safety Report.

In addition to the above, appropriate Safety reporting will be done as per the manufacturing facility's SOP for Sterility Testing. A sample of each product is retained by Quality Systems and will be sent to the Microbiology Laboratory for repeat testing and speciation. An Out-of-Specification (OOS) Investigation will be conducted by the Quality Systems staff of the manufacturing laboratory including root cause analysis, review of viable environmental monitoring results collected at the time of manufacturing on personnel, equipment and reagents. Whether or not attribution is established, a formal Corrective and Preventive Action plan will be issued by the manufacturing facility Quality Systems staff and appropriate remediation will be performed including retraining of manufacturing personnel, elimination of any contaminated reagents and re-cleaning of the production facility followed by viable microbiological monitoring to establish effectiveness of cleaning.

8 CORRELATIVE/SPECIAL STUDIES

CAR T cell therapy targeting a single tumor associated antigen has mediated striking remissions in B cell leukemia and lymphoma. The clinical experience to date has demonstrated variable patterns of tumor remission and CAR T cell efficacy and persistence. In subjects treated with singular CD19 specific or CD22 specific CAR T cells, relapse patterns have included both respective CD19 or CD22 negative disease or relapsed disease with persistent, variable surface expression of the targeted antigen. The complex interplay of tumor, T cell and intrinsic CAR properties that influence these outcomes are not well understood. We aim to utilize this safety study as an opportunity to collect correlative data that will permit extensive study of both the B cell and T cell compartment prior to and following CAR T cell therapy. We aim to integrate multi-dimensional technologies to permit complex analyses of the apheresis product, the CAR T cell product pre-infusion and *in vivo* expanded CAR T cells following antigen

encounter. We additionally aim to investigate properties of B cell tumors that render them susceptible to CAR T cell cytotoxicity and study physiologic and malignant B cell remodeling under the pressure of multi-targeted CAR therapy.

Overall goal of study correlatives:

- Explore the T cell and tumor properties that influence CAR T cell efficacy and persistence and permit disease relapse

Specific Aims:

- Assess the impact of T cell subset composition as delineated using flow cytometry, mass cytometry and ATAC-Seq on CAR T cell expansion and persistence
- Where possible, use TCR sequencing to fate map cells contained in the apheresis of manufactured product to persistent CD19/CD22-CAR T cells as an exploratory aim to identify subsets with a greater likelihood of T cell persistence in the setting of adoptive cell therapy.
- Characterize antigen expression on relapsed B-ALL and B cell lymphoma following multi-specific CAR-mediated targeting
- Utilize the CAR-mediated arrest of B cell maturation to gain insight on physiologic and pathophysiologic B cell development, maturation arrest and alternative survival pathways in normal and malignant B cells.
- Explore the level CD19/CD22 surface expression and CD19/CD22 site density on leukemic blasts, when feasible and correlate with clinical response to CAR T cells.
- To establish the utility of chromatin structure and epigenomic technology to characterize CAR T cell therapies.

8.1 SAMPLE COLLECTION SCHEDULE

8.1.1 Study Correlatives

The samples to be collected and schedule for sample collection is detailed in section **14.9**, Appendix I and in the Study Laboratory Manual. Every effort will be made to collect correlative samples as described in Section **14.9** Appendix I, but changes in tests, schedules, blood volume limitations and/or conduct of apheresis may be modified based on schedule conflicts, holidays, patient schedules, patient's condition, etc., and such variations will be noted but will NOT constitute a protocol deviation given the exploratory nature of the correlative testing.

8.1.2 Monitoring Gene Therapy Trials: Persistence, and RCL, and Toxicity:

Sample collection, storage and testing of the following will be performed on this protocol. Please refer to sections **14.6, 14.7, 14.8** (Appendices F, G and H) for Gene Therapy Long Term Follow Up.

8.1.2.1 Monitoring Persistence

Due to the nature of these studies, it is possible that expansion of specific T-cell clones will be observed as tumor reactive T-cells proliferate in response to tumor antigens.

Therefore, care will be taken to track T-cell persistence both immunologically and molecularly (See section **14.9**, Appendix I) by flow cytometry using anti-CD19/CD22 anti-idiotype antibody.

8.1.2.2 Safety Assessment Testing for Replication Competent Lentivirus in Lentiviral Vector Based Gene Therapy Products during Subject Follow up

In compliance with OSP and FDA's ***Guidance for Industry: Gene Therapy Clinical Trials – Observing Participants for Delayed Adverse Events (2006)***, subjects who have received at least one dose of a genetically engineered cellular therapy will be evaluated for long term safety and occurrence of adverse events according to the requirements established by FDA guidance and the NIH Guidelines for Research Involving Recombinant or Synthetic Nucleic Acid Molecules (NIH Guidelines) (2016).

8.1.2.2.1 Replication-competent Lentivirus Evaluations

Detection of RCL: Blood samples will undergo analysis at Indiana University Gene Therapy Testing Laboratory (IU GTTL) for detection of RCL by detection of p24 antigen and reverse transcriptase activity (Product Enhanced Reverse Transcriptase [PERT]) prior to cell infusion (at baseline) and at 3, 6, and 12 months (± 4 weeks) post cell administration (see **Section 14.6**). Samples sent to IU VPF MUST be drawn on Monday, Tuesday, Wednesday or Thursday, and shipped on ice priority overnight. The lab cannot accept samples on the weekends. If all samples in the first year are negative, annual blood samples will be archived for the next 4 years (± 1 month) (for a total of 5 years after cellular therapy) with a brief clinical history conducted annually. If any post-treatment samples are positive, further analysis of the RCL and more extensive subject follow-up will be undertaken, in consultation with the FDA.

If a subject dies or develops neoplasms during this follow up, efforts will be made to assay a biopsy sample for RCL.

8.1.2.3 Persistence of genetically engineered transduced cells

The procedures and methodologies for testing persistence of genetically engineered transduced cells will be specified in section **14.9**, Appendix I.

If any subject has more than 5% persistence of gene transduced cells at Month 6, samples will be subjected to flow cytometry using anti-CD19 anti-idiotype antibody that would allow the identification of clonality of persisting gene transduced cells. Such techniques may include analysis of BV chain expression, T cell cloning or LAM-PCR. If a predominant or monoclonal T cell clone derived from gene transduced cells is identified during the follow-up, the integration site and sequence will be identified and subsequently analyzed against human genome database to determine whether the sequences are associated with any known human cancers. If a predominant integration site is observed, the T cell cloning or LAM-PCR test will be used at an interval of no more than 3 months after the first observation to see if the clone persists or is transient. In all instances where monoclonality is persistent and particularly in instances where there is expansion of the clone, regardless of whether or not the sequence is known to be associated

with a known human cancer, the subject should be monitored closely for signs of malignancy, so that treatment, if available, may be initiated early.

8.2 Blood Drawing Limits for Research Purposes

8.2.1 Adults

The maximum amount of blood that may be drawn per day from adult subjects is 2.5% of total blood volume (192-200 mL). The volume to be drawn per day for research purposes will comply with institutional policy. Subjects may undergo a small volume apheresis (approx. one to two blood volumes) in lieu of standard blood draw to obtain peripheral blood lymphocytes for correlative studies including Day 28 (± 1 week), and 3 (± 1 month), 6 (± 1 month), 9 (± 1 month), and 12 (± 2 months) months.

9 STUDY CALENDAR

			Preparative Regimen and Cell Infusion			Post Treatment Assessment				Long Term Follow-Up		
**Procedure**	**Screening[a]**	**Apheresis**	**Day -5, Day -4, Day -3**	**Prior to cell infusion (< 24 h)**	**Day 0**	**Daily, Day 1 to Day 14 (± 2 d)**	**Twice weekly, Day 15 to Day 27 (± 4 d)**	**Day 28 (± 4 d)**	**Monthly, Month 2-3 (±2 weeks)**	**Month 6 (±1 mo.) 9, (±1 mo) 12 (±2 mo) q6-12 month s to 5 years**	**Annually, Year 6 to 15**	**At Disease Progression**
History (at screening) and PE	X			X	X	X	X	X	X	X		
Vital signs	X	X	X	X	X[h]	X[h]	X[h]	X[h]	X	X		
Neurologic exam				X		X[j]	X[j]	X[j]	X	X		
Performance Status	X							X	X	X		
Height (screening and Day -6-only) and weight	X	X	X	X				X	X			
Labs[c]												
• CBC with diff	X	X[e]	X	X		X	X	X	X	X		
• PT/PTT	X											
• Chemistries	X[z]	X[e]	X	X		X	X	X	X			
• Phosphorus	X	X[e]	X[w]			X[k]	X[k]					
• Magnesium	X	X[e]	X[w]	X		X[k]	X[k]					
• CRP	X	X[e]	X[w]	X		X[k]	X[k]	X				
• Ferritin	X		X[w]					X				
• Uric acid and LDH			X[w]			X[k]	X[k]					
• TSH				X[x]								
• CD19 staining by IHC or flow cytometry	X[s]											X
• HIV, HbsAG, anti-HCV	X[y]	X[e]										
• IgG level									X[p]			
• Urine analysis	X			X								
• Pregnancy test (females of child-bearing potential only)	X	X[e]	X[e]									
• RCL blood sample				X[aa]					X[aa]	X[aa]		
ECG	X											

			Preparative Regimen and Cell Infusion			Post Treatment Assessment				Long Term Follow-Up		
**Procedure**	**Screening[a]**	**Apheresis**	**Day -5, Day -4, Day -3**	**Prior to cell infusion (< 24 h)**	**Day 0**	**Daily, Day 1 to Day 14 (± 2 d)**	**Twice weekly, Day 15 to Day 27 (± 4 d)**	**Day 28 (± 4 d)**	**Monthly, Month 2-3 (±2 weeks)**	**Month 6 (±1 mo.) 9, (±1 mo) 12 (±2 mo) q6-12 month s to 5 years**	**Annually, Year 6 to 15**	**At Disease Progression**
ECHO, MUGA or cardiac MRI	X[u]											
Correlative Research Studies	Correlative samples outlined in Section **14.9**, Appendix I											
• Leukapheresis		X[f]						X[l]	X[l]	X[l]		
Disease Evaluation												
• PET/CT chest, abdomen, pelvis; brain MRI (or other appropriate imaging)	X[n]		X[n]					X[n]	X[n]	X[n]		
• Lumbar puncture	X[d,n]							X[n]	X[n]	X[n]		
• Bone marrow aspirate	X[n]		X[n]					X[n]	X[n]	X[n]		
• MRD blood sample	X[n]							X[n]	X[n]	X[n]		
• Lymph node biopsy	X[t]					X[m]	X[m]	X[m]	X[m]			X[m]
Treatment Regimen												
• Fludarabine			X[g]									
• Cyclophosphamide			X[g]									
• Levetiracetam				X	X[v]	X[v]	X[v]	X[v]				
• CD19/CD22-CAR T cells infusion					X[i]							
Response Evaluation								X[o]	X[o]	X[o]	X[q]	X[q]
Adverse Events[b]		X	X	X	X	X	X	X	X	X[r]		X[r]
Concomitant Medications[b]	X	X	X	X	X	X	X	X	X	X[r]		X[r]

a: Within 28 days prior to enrollment, unless otherwise specified.
b: Monitor continuously during reporting period.
c: Laboratory evaluation to include; Chemistries: Sodium (Na), Potassium (K), Chloride (Cl), Total CO2 (bicarbonate), Creatinine, Glucose, Urea nitrogen (BUN), Albumin, Calcium total, Alkaline Phosphatase, ALT/GPT and/or AST/GOT, Bilirubin, Total Protein); creatinine clearance may be performed if the serum creatinine is elevated. The PI may order additional tests in some subjects if needed to fully assess clinical status and obtain baseline results. Examples of such tests include: haptoglobin, soluble IL2R, immunoglobulin levels, and viral serology or PCR for cytomegalovirus (CMV), herpes simplex virus (HSV), varicella zoster virus (VZV), and Epstein-Barr virus (EBV), C-reactive peptide.

d: For all subjects at baseline
e: Within 7 days prior to leukapheresis procedure and Day -5
f: For cell acquisition for product development. This may be skipped if subject has cryopreserved cells that meet IND criteria.
g: Adult patients will receive prophylactic continuous home IV fluid infusion of Normal saline 150ml/hour for 12-24 hours before and throughout cyclophosphamide and fludarabine treatment. IVF is removed after final infusion of Flu CY and patient receives another 1 liter IVF before discharge home.
h: Vital signs (blood pressure, heart rate, respiratory rate, pulse oximetry, temperature) collected on Day 0: prior to infusion, once during infusion, and then 30, 60 and 120 minutes after infusion; then Day 1-28: every 6-8 hours during hospitalization and with each visit after discharge.
i: Premedications will be provided as described in protocol. Subject may be offered additional CAR T cell treatments if enough cells were manufactured from the initial preparation, the subject experienced at least partial response to the first infusion, and the subject meets all eligibility criteria. Subsequent infusions will follow the same procedures as the first.
j: Neurologic exam daily and ICE score every 8 hours (± 2 h) during hospitalization or up to Day 14, then with each visit after discharge
k: Daily while hospitalized and then Day 14 (± 2 days) and 21 (± 2 days). LDH testing only required if elevated prior to lymphodepleting chemotherapy
l: Subjects may undergo a small volume apheresis (approx. one to two blood volumes) in lieu of standard blood draw to obtain peripheral blood lymphocytes for correlative studies including Day 28 (±7 days), and 3 (± 1 month) , 6 (± 1 month) , 9 (± 1 month) , and 12 (± 2 months) months.
m: Lymph node biopsy will be obtained from lymphoma subjects once between Day 7 and Day 28, if clinically feasible. A PMBC sample (for CD19/CD22-CAR T cells, etc) and lymph node biopsy should be collected at the time of progression, prior to starting any subsequent anticancer therapy, if clinically feasible.
n: Subjects with lymphoma will undergo PET/CT; subjects with leukemia will undergo bone marrow biopsy at Day 28 and Month 3, 6, 9, 12, then q6-12 months. Disease evaluations may be repeated if >28 days passes between enrollment and start of lymphodepletion chemotherapy or if bridging anti-neoplastic therapy is used post-enrollment (per **Section 4.2.5**). Other methodologies including lumbar puncture, x-rays, MRI, MRD, etc determined by investigator based on sites and type of disease; consistency throughout protocol evaluations is necessary. Day 28 will be considered the response evaluation for subjects with ALL. Month 3 will be considered the response evaluation for subjects with DLBCL. If subject's disease has not progressed by Month 24, disease assessments will continue at standard of care frequency.
o: If no response (i.e., progressive disease) by Day 28 (patients with ALL) or month 3 (patients with lymphoma), then no further close clinical follow-up required and subject will be followed as per section **5.5.7**. Otherwise, subject will be monitored by physical exams, disease evaluations, vitals, CBC with diff, Chemistries, and IgG levels until relapse or subject proceeds to other therapies at investigator's discretion; at which time long-term follow-up will proceed as per section **5.5.7**
p: Every 3-12 weeks post infusion until Month 3.
q: After 5 years, health status data will be obtained from surviving subjects via telephone contact or mailed questionnaires. The long term follow up period for lentiviral vectors is 15 years. Persistence of CAR gene transduced cells in the blood: prior to cells, 1 week, 4-6 weeks, 3 and 6 months post cell infusion
r: Targeted adverse/serious adverse event reporting and concomitant medication collection until Month 24, disease progression, or start of alternate therapy (whichever occurs first).
s: CD19 requirement can be met since diagnosis, but must be re-demonstrated if patient has received targeted anti-CD19 therapy, i.e. Blinatumomab or CD19-CAR T cells.
t: Lymph node diagnostic material is required, but not within 28 days of enrollment.
u: Testing performed within 180 days or after most recent anthracycline based treatment may be used for confirmation of eligibility
v: Continue until end of neurotoxicity or at investigator discretion.
w: Day-5 or Day -6 only
x: Within 28 days of starting lymphodepletion.
y: Within time requirements for autologous apheresis donation.
z: Perform creatinine clearance if the serum creatinine elevated
aa: RCL samples collected and sent to IU GTTL: prior to cell infusion; and at 3 (± 1 month), 6 (± 1 month), and 12 months (± 2 months) post cell infusion. Subsequent RCL blood samples will be stored annually for 4 additional years if all RCL in first year negative.

10 MEASUREMENTS

10.1 PRIMARY OUTCOME MEASURES

10.1.1 Primary Objectives:

1. Determine the feasibility of producing CD19/CD22-CAR T cells meeting the established release criteria

The primary endpoint feasibility is defined by the rate of successful manufacture and expansion of the CD19/CD22-CAR T cells to satisfy the targeted dose level and meet the required release specifications outlined in the Certificate of Analysis (COA).

In addition to aiming to evaluate up to 6 subjects at a given dose level with respect to toxicity, the number of subjects which can successfully manufacture the targeted dose number will be determined. Subjects will be enrolled on a given dose level until adequate subjects are enrolled to produce the correct number of cells for safety evaluation at that dose level. Specifically, dose escalation will proceed as long as 3 or more of the first 3 to 6 subjects in a dose level are able to produce adequate cells for evaluation. Primary Objective:

2. Assess the safety of administering escalating doses of autologous CD19/CD22-CAR T cells that can be feasibly produced to meet established release specifications in children and young adults with B cell ALL or lymphoma following a cyclophosphamide/fludarabine conditioning regimen. The following dose escalation will be used:

   - Dose Level -1: 3×10^5 transduced T cells/kg (± 20%)
   - Dose Level 1: 1×10^6 transduced T cells/kg (± 20%)
   - Dose Level 2: 3×10^6 transduced T cells/kg (± 20%)
   - Dose Level 3: 1×10^7 transduced T cells/kg (± 20%)

The endpoint for Safety of CD19/CD22-CAR T cells is evidenced by the incidence and severity of dose limiting toxicities (DLTs) (i.e. laboratory abnormalities, changes in vital signs, and changes in physical examination) following chemotherapy preparative regimen and infusion of CD19/CD22-CAR T cell, recorded and graded according to the Common Terminology Criteria for Adverse Events (CTCAE) Version 4.03 at three dose levels until the maximum tolerated dose (MTD) is determined. If Dose level 3 can be feasibly manufactured and is administered in up to 6 subjects without evidence of DLT or efficacy, consideration will be given to amending the trial to examine higher dose levels.

10.2 SECONDARY OUTCOME MEASURES

10.2.1 Secondary Objective: Evaluate whether CD19/CD22-CAR T cells can mediate antitumor effects in adults with B cell malignancies.

The secondary endpoints for this study are defined as the ability to achieve a clinical response after administration of CD19/CD22-CAR T cells in these subject populations (Subjects with DLCBL and subjects with ALL):

10.2.1.1 Response Criteria

-The response rate as measured by Response Criteria for Lymphoma (Refer to section 14.5 Appendix E Response Criteria):

- Complete Response (CR)
- Partial Response (PR)
- No Response or Stable Disease (SD)
-Progressive Disease (PD)

-The response rate as measured by Response Criteria for ALL (See Appendix (*Modified from: Cheson BD, et al.)

- Complete Response (CR)
- Partial Response (PR)
- Hematological Activity
- Stable Disease (SD)
-Progressive Disease (PD)

10.3 Exploratory Objectives

- ✓ Evaluate the frequency of CD22+ expression on lymphoma cells, and determine site density when possible.
- ✓ Analyze alterations in early B cell development induced by immune pressure exerted via CD19/CD22-CAR T cells.
- ✓ Evaluate whether subjects receiving CD19/CD22-CAR T cells relapse with loss or diminished expression of CD19 and/or CD22, when feasible.
- ✓ Measure persistence of CD19/CD22-CAR T cells in the blood, bone marrow and CSF, and explore correlations between CD19/CD22-CAR T cell properties and CAR T cell efficacy and persistence.

11 REGULATORY CONSIDERATIONS\

11.1 Institutional Review of Protocol

The protocol, the proposed informed consent and all forms of participant information related to the study (e.g. advertisements used to recruit participants) will be reviewed and approved by the Stanford IRB and Stanford Cancer Institute Scientific Review Committee (SRC). Any changes made to the protocol will be submitted as a modification and will be approved by the IRB prior to implementation. The Protocol Director will disseminate the protocol amendment information to all participating investigators.

11.2 Data and Safety Monitoring Plan

11.2.1 Clinical Team

The clinical research team will meet on a regular basis during dose escalation and when subjects are being actively treated on the trial to discuss cell manufacturing, toxicities, eligibility questions, trial accrual, and treatment needs. Decisions about dose level

enrollment and dose escalation if applicable will be made based on the toxicity data from prior subjects. Members from cell processing facilities will join as needed.

All data will be collected in a timely manner and reviewed by the investigator or a lead associate investigator. Adverse events will be reported as required above. Any safety concerns, new information that might affect either the ethical and or scientific conduct of the trial, or protocol deviations will be immediately reported to the IRB, DSMC, APB and to the Sponsor.

The principal investigator at each site will review adverse event and response data on each subject to ensure safety and data accuracy. The principal investigator will personally conduct or supervise the investigation and provide appropriate delegation of responsibilities to other members of the research staff.

Data Safety Monitoring Committee (DSMC) The Stanford Cancer Center Data and Safety Monitoring Committee (DSMC) will be the monitoring entity for this study. The DSMC will audit study-related activities to determine whether the study has been conducted in accordance with the protocol, local standard operating procedures, FDA regulations, and Good Clinical Practice (GCP). In addition, the DSMC will regularly review serious adverse events, adverse events, and protocol deviations associated with the research to ensure the protection of human subjects. Results of the DSMC audit will be communicated to the IRB and APB and the appropriate regulatory authorities at the time of continuing review, or in an expedited fashion, as needed.

11.3 Data Management Plan

Case Report Forms (CRFs) are printed or electronic documents designed to record all protocol-related information on each trial participant. CRFs should summarize the clinical findings and observations necessary to ensure safety of participants on the study, and to document the study outcomes. Data will be entered into the Stanford database, a cloud based electronic data capture system.

All data will be kept secure. Personal identifiers will not be used when collecting and storing data. An enrollment log will be maintained in the regulatory binder/file which is the only location of personal identifiers with unique subject identification number.

12 COLLABORATIVE AGREEMENTS

Collaborative research agreement exists for conduct of correlative studies with Adaptive Biosciences, Inc. This collaborative agreement is disclosed in the informed consent document.

13 STATISTICAL CONSIDERATIONS

13.1 Statistical Design

A formal statistical analysis plan (SAP) will be prepared and finalized before database lock for the final analysis for the study report. The SAP will provide details regarding the definition of analysis subjects (populations), analysis variables, and analysis methodology to meet all study objectives.

The principle and key elements of the SAP are provided as follows:

- In general, safety and efficacy data will be summarized with descriptive statistics, including means, standard deviations, medians, minimums and maximums for continuous variables, the number of subjects and percent in each category for categorical variables.
- Data from each individual will be tabulated as appropriate. Efficacy and safety endpoints will be tabulated by dose cohort and time point.

13.1.1 Primary Objective:

Assess the safety of administering escalating doses of autologous CD19/CD22-CAR engineered T cells that can be feasibly produced to meet established release specifications in children and young adults with B cell ALL or lymphoma following a cyclophosphamide/fludarabine conditioning regimen. The following dose escalation will be used:

- Dose Level -1: 3×10^5 transduced T cells/kg (± 20%)
- Dose Level 1: 1×10^6 transduced T cells/kg (± 20%)
- Dose Level 2: 3×10^6 transduced T cells/kg (± 20%)
- Dose Level 3: 1×10^7 transduced T cells/kg (± 20%)

The endpoint for Safety of CD19/CD22-CAR T cells is evidenced by the incidence and severity of dose limiting toxicities (DLTs) (i.e. laboratory abnormalities, changes in vital signs, and changes in physical examination) following chemotherapy preparative regimen and infusion of CD19/CD22-CAR T cells, recorded and graded according to the Common Terminology Criteria for Adverse Events (CTCAE) Version 4.03 at three dose levels until the maximum tolerated dose (MTD) is determined. If Dose level 3 can be feasibly manufactured and is administered in up to 6 subjects without evidence of DLT or efficacy, consideration will be given to amending the trial to examine higher dose levels.

The dose escalation procedure follows a 3+3 design as described in Section 5.7.3**Error! Reference source not found.**, beginning with dose level 1, based on the DLT count in each cohort, with escalation also constrained by the feasibility of producing the doses called for. The purpose of the design is not to invert the dose-toxicity curve at a target DLT rate, but rather to proceed with appropriate caution to dose level 3, which we expect will be reached without observing any DLT. Safety monitoring will continue throughout the study in the expanded cohort of up to 30 subjects at the final MTD (or highest dose tested).

13.1.2 Primary Objective:

In addition to evaluating up to 6 subjects at a given dose level with respect to toxicity, the number of subjects which can successfully manufacture the targeted dose number will be determined. Subjects will be enrolled on a given dose level until adequate subjects are enrolled to produce the correct number of cells for safety evaluation at that dose level.

13.2 Safety Endpoints

All subjects who receive experimental treatment (conditioning lymphodepletion

chemotherapy regimen and CD19/CD22-CAR T cell infusion) will be analyzed for safety and efficacy.

Subjects not treated for any reason will be included in the disposition tabulation but will be excluded from the safety and efficacy analysis.

The safety and tolerability of CD19/CD22-CAR T cell regimen will be assessed by:

- Suspected adverse events, and
- Suspected serious adverse events

As evidenced by:

  - Changes in clinical laboratory tests (clinical chemistry, hematology, etc).
  - Changes in vital signs (blood pressure, pulse, respiratory rate and body temperature).
  - Changes in physical exams. Signs and symptoms assessed may require additional testing as clinically indicated such as ECG, PFT, radiographic studies, etc.
  - Subject reported signs and symptoms

Safety data will be analyzed per standard methods and interpreted descriptively for each dose cohort. Safety data will be summarized for each dose cohort separately and for all dose cohorts combined. Adverse events will be assessed using the CTCAE version 4.03 for type and severity of event. Serious Adverse Events will be summarized for each dose cohort and for all dose cohorts combined. Reasons for discontinuation of study therapy will be tabulated.

Laboratory testing includes hematology, serology, serum chemistry, and urinalysis. Baseline laboratory testing will be those results obtained prior to initiating the conditioning lymphodepletion chemotherapy regimen. The study will utilize local lab for all clinical laboratory testing. Laboratory data will be tabulated based on the following result class.

- Normal: result is within the local lab normal range
- Abnormal: result is either higher or lower than the normal range

All abnormal values will be assessed for clinical significance; clinical significance will be captured in the case report form.

Vital signs collected immediately prior to receiving study drug will be the baseline vital signs. Observed vital sign values and change from baseline in vital signs at each visit will be summarized without formal statistical testing.

Vital sign result may also be tabulated based on the following result class.

- Normal: result is within the normal range
- Abnormal: result is either higher or lower than the normal range

All abnormal values will be assessed for clinical significance; clinical significance will be captured in the case report form. Number and percent of subjects within each result class will be tabulated by time point for each vital sign.

Findings of physical examinations will be tabulated by dose cohorts without formal statistical analysis.

13.3 Protocol Safety Rules

The study will be halted pending discussions with the FDA, IRB and APB if any of the following conditions are met:

a. DLT occurs in dose -1 cohort.
b. Development of EBV lymphoma or polyclonal lymphoproliferative disease (PLPD).
c. Any Grade 5 event at least possibly related to the research regimen.

13.4 Feasibility endpoint

Feasibility will be defined as the successful manufacturing of CD19/CD22-CAR T cells that meet established release criteria to satisfy the targeted dose level. Although we anticipate reaching the targeted cell dose during manufacture, feasibility of manufacturing cells remains a primary objective in this subject population. Subjects will be enrolled on a given dose level until adequate subjects are enrolled to produce the correct number of cells for safety evaluation at that dose level. Specifically, dose escalation will proceed as long as 3 or more of the first 3 to 6 subjects in a dose level are able to produce adequate cells for evaluation. For example, this might mean that 6 to 9 subjects will need to be enrolled at a dose level to result in 6 for the safety evaluation. However, if less than 3 of 6 subjects at a given dose level are able to have adequate cells produced, evaluation of that level and beyond for safety and feasibility will not take place. If cell growth limitations preclude administration of the targeted cohort cell dose, the subject will receive as many cells as possible, and be considered part of the lower dose cohort. If a minimum of 1.0×10^5 CD19/CD22-CAR-transduced T cells per kg cannot be obtained for infusion, the subject may be treated but will not be evaluable for toxicity or response, but will be considered a feasibility failure. Specifically, if after the first 6 subjects have been enrolled at a given dose level, more than 3 are unable to have adequate CD19/CD22-CAR T cells produced (that meet COA for infusion), accrual to that dose level will stop and the dose escalation phase of the study will also end, since the upper 90% one-sided confidence interval about 3/6 is 79.9%; thus, it would be unlikely that the true feasibility rate is 80% or greater for a given, which would be desirable. The evaluation of subjects in the expansion cohort will take place using the highest dose level at which feasibility, as well as safety, was identified. In the expansion cohorts, the fraction which are able to manufacture the targeted dose level will also be monitored, and beginning with the 6th subject in an expansion cohort, if at any point fewer than half of the enrolled subjects are able to manufacture an acceptable level of cells, the accrual to the expansion cohort will end.

13.5 Efficacy Endpoint

- ✓ Evaluate whether CD19/CD22-CAR T cells can mediate clinical activity in two cohorts: adults with B-ALL or DLBCL.

Although a secondary endpoint, efficacy will be important in determining if a phase 2 study is warranted. While the feasibility and safety aspects can be addressed in the pooled sample, the efficacy analysis requires two separate cohorts, each of 30 subjects, due to between-disease differences in the efficacy of the implied historical control.

DLBCL cohort: A futility analysis will be conducted when 15 subjects at the MTD (or highest dose studied) have reached the month 3 PET imaging and/or bone marrow assessment (which is the primary efficacy endpoint for DLBCL), and if 5 or fewer subjects have a CR or PR, further enrollment to the cohort will stop, with the upper 80% Confidence Limit of 0.476 not reaching the 0.48 minimum overall response rate expected for the "competing" treatment with active therapy such as autologous HCT for chemotherapy or alternative CAR-T therapies. Assuming no futility stopping, no failure of manufacturing the required doses, and no DLT observed, the study will reach a maximum size of 30 DLBCL subjects treated at dose 3.

The precision of estimate of the probability of dichotomous outcomes (as measured by 90% half-widths of two-sided 90% confidence intervals) relating to evidence for efficacy is no worse than 0.17, under the above conditions.

Overall survival (OS) and progression-free survival (PFS) will be assessed by dose cohort. Progression-free survival (PFS) will be measured from the start of the preparative regimen until the documentation of disease progression or death due to any cause, whichever occurs first. Overall survival (OS) will be determined as the time from the start of the preparative regimen until death.

B-ALL cohort: A futility analysis will be conducted when 15 subjects at the MTD (or highest dose studied) have reached Day 28 PET imaging and/or bone marrow assessment (which is the primary efficacy endpoint for B-ALL) and if 6 or fewer subjects have a CR or PR, further enrollment to the cohort will stop, with the upper 80% Confidence Limit of 0.542 not reaching the 0.60 minimum overall response rate expected for the "competing" treatment with active therapy such as chemotherapy, transplantation or alternative CAR-T therapies. Assuming no futility stopping, no failure of manufacturing the required doses, and no DLT observed, the study will reach a maximum size of 30 ALL subjects treated at dose 3.

The precision of estimate of the probability of dichotomous outcomes (as measured by 90% half-widths of two-sided 90% confidence intervals) relating to evidence for efficacy is no worse than 0.17, under the above conditions.

Overall survival (OS) and progression-free survival (PFS) will be assessed by dose cohort. Progression-free survival (PFS) will be measured from the start of the preparative regimen until the documentation of disease progression or death due to any cause, whichever occurs first. Overall survival (OS) will be determined as the time from the start of the preparative regimen until death.

13.6 Exploratory Analysis

a) Evaluate whether subjects receiving CD19/CD22-CAR T cells relapse with loss or diminished expression of CD19 and/or CD22.

b) Measure persistence of CD19/CD22-CAR T cells in the blood, bone marrow and CSF, and explore correlations between CD19/CD22-CAR T cell properties and CAR T cell efficacy and persistence.

c) Analyze alterations in early B cell development induced by immune pressure exerted via anti-CD19/CD22-CAR T cells.

13.6.1 Persistence of CD19/CD22-CAR T cell Analyses

✓ Measure persistence of adoptively-transferred CD19/CD22-CAR T cells in the blood and, where possible, the bone marrow and CSF of subjects.

Peripheral blood, bone marrow aspirate, and CSF will be collected when available and separately analyzed for the presence of CD19/CD22-CAR T cells. The percentage of all CD3+ cells in a sample that are positive by flow cytometry for CD19/CD22-CAR containing T cells will be analyzed and reported as time from T cell infusion.

13.6.2 To establish the utility of chromatin structure and epigenomic technology to characterize CAR T cell therapies.

To achieve this exploratory objective the investigators will attempt to establish parameters for how best to utilize the technology in CAR research to:

1. establish basis for blood therapeutic monitoring;
2. derive blood biomarkers for prediction of the safety and efficacy of CAR cell therapy; and
3. develop metrics for CAR-T product release criteria that can be used during the manufacturing of the product.

Blood samples will be collected for testing according to Section **14.9**, Appendix I.

13.7 Sample Size

13.7.1 Accrual estimates

Given the number of subjects seeking out adoptive cell therapy trials the rate limiting factor at this time is the time and labor intensive nature of manufacturing the CD19/CD22-CAR T cells. We anticipate enrollment of 2-4 subjects per month given the number of subjects with B-ALL and DLBCL treated in Stanford Cancer Therapy Clinics.

13.7.2 Sample size justification

The primary objectives of this study are safety and feasibility. A minimum of 3 subjects and a maximum of 9 subjects will be enrolled at each dose level to evaluate toxicity of study therapy and feasibility of manufacturing cells. With Amendment 1 the culture time for generating CD19/CD22-CAR T cells is shortened to approximately 7 days. To ensure safety, 3 additional subjects will be treated at dose level 1 with cells generated with the

shortened culture time (21 total in order to determine a safe and feasible dose). Up to 6 subjects may be replaced for inevaluable subjects (subjects enrolled who cannot receive cells due to concomitant illness, or unforeseen circumstances). The MTD or highest dose evaluated (if MTD is not reached) will be expanded in two cohorts (subjects with DLBCL and subjects with ALL) to a minimum of 15 and a maximum of 30 subjects per cohort to conduct a preliminary evaluation of efficacy. Therefore a minimum of 24 subjects and a maximum of 90 subjects may be enrolled to meet study endpoints.

14 APPENDICES

14.1 APPENDIX A: PERFORMANCE STATUS CRITERIA

**PERFORMANCE STATUS CRITERIA** *Karnofsky and Lansky performance scores are intended to be multiples of 10.*					
**ECOG (Zubrod)**		**Karnofsky**		**Lansky**	
**Score**	**Description**	**Score**	**Description**	**Score**	**Description**
0	Fully active, able to carry on all pre-disease performance without restriction.	100%	Normal, no complaints, no evidence of disease.	100%	Fully active, normal.
		90%	Able to carry on normal activity; minor sighs of symptoms of disease.	90%	Minor restrictions in physically strenuous activity.
1	Restricted in physically strenuous activity but ambulatory, able to carry out light or sedentary work, e.g., light housework, office work.	80%	Able to carry on normal activity with effort; some signs or symptoms of disease.	80%	Active, but tires more quickly.
		70%	Cares for self, unable to carry on normal activity or do active work.	70%	Both greater restriction of, and less time spent in, play activities.
2	Ambulatory and capable of all self-care but unable to carry out any work activities. Up and about more than 50% of waking hours.	60%	Requires occasional assistance but is able to care for most of own needs.	60%	Up and around, but minimal active play; keeps busy with quieter activities.
		50%	Requires considerable assistance and frequent medical care.	50%	Gets dressed, but lies around much of the day; no active play; able to participate in quiet play and activities.
3	Capable of only limited self-care, confined to bed or chair more than 50% of waking hours	40%	Disabled; requires special care and assistance.	40%	Mostly in bed; participates in quiet activities.
		30%	Severely disabled; hospitalization indicated, although death not imminent.	30%	In bed; needs assistance even for quiet play.
4	Completely disabled. Cannot carry on any self-care. Totally confined to a bed or chair	20%	Very ill; hospitalization necessary; active supportive treatment required.	20%	Often sleeping; play entirely limited to very passive activities.
		10%	Moribund, fatal process progressing rapidly	10%	No play; does not get out of bed
5	Dead	0%	Patient expired	0%	Unresponsive; Dead

14.2 Appendix B: Guidelines for Toxicity Assessment and Management – Dose Expansion Cohort

14.2.1 Guidelines for Grading Suspected Cytokine Release Syndrome according to ASBMT CRS Consensus Grading

Organ toxicities associated with CRS may be graded according to CTCAE v5.0 but they do not influence CRS grading.

**CRS Parameter**	**Grade 1**	**Grade 2**	**Grade 3**	**Grade 4**
Fever†	Temp ≥ 38 °C	Temp ≥ 38 °C	Temp ≥ 38 °C	Temp ≥ 38 °C

With

Hypotension	None	Not requiring vasopressors	Requiring a vasopressor (excluding vasopressin)	Requiring multiple vasopressors (excluding vasopressin)

And/or§

Hypoxia	None	Requiring low-flow nasal cannula* or blow-by	Requiring high-flow nasal cannula*, facemask, nonrebreather mask, or Venturi mask	Requiring positive pressure (e.g. CPAP, BiPAP, intubation, mechanical ventilation)

† Fever is defined as temperature ≥ 38° C not attributable fo any other cause. In patients who have CRS then receive anti-pyretics or anti-cytokine therapy such as tocilizumab or steroids, fever is no long required to grade subsequent CRS severity. In this case, CRS grading is driven by hypotension and/or hypoxia.

§ CRS grade is determined by the more severe event: hypotension or hypoxia not attributable to any other cause. For example, a patient with a temperature of 39.5 ° C, hypotension requiring one vasopressor and hypoxia requiring low-flow nasal cannula is classified as having Grade 3 CRS.

* Low-flow nasal cannula is defined as oxygen delivered at ≤ 6 liters/minute. Low flow also includes blow-by oxygen delivery, sometimes used in pediatrics. High-flow nasal cannula is defined as oxygen delivered at > 6 liters/minute.

14.2.2 Guidelines for Treating Suspected Cytokine Release Syndrome

Grade 1	*Symptoms are not life threatening and require symptomatic treatment only*<br>Treatment:<br>✓ Assess for infection<br>✓ Treat fever and neutropenia if present, monitor fluid balance, antipyretics, analgesics as needed.
Grade 2	*Symptoms require and respond to moderate intervention*<br>Treatment:<br>✓ As above for Grade 1<br>✓ Monitor organ function closely (incl. cardiac, respiratory, renal, neurologic, liver)
Grade 3<br>or Grade 2 with co-morbidity	*Symptoms require and respond to aggressive intervention*<br>Treatment:<br>✓ As above for Grade 2<br>✓ Consider tocilizumab with or without corticosteroids<br>✓ Consider vasopressors, oxygen supplementation
Grade 4	*Life-threatening symptoms*<br>Treatment:<br>✓ As above<br>✓ Positive pressure airway assistance or Mechanical ventilation<br>✓ Multiple vasopressors or high dose vasopressors

High dose vasopressor doses shown in Table 2.

Tocilizumab is administered at a dose of 4-8 mg/kg infused over 1 hour (not to exceed 800 mg).

Table 2. High-Dose Vasopressors (all doses are required for ≥ 3 hours)

Norepinephrine monotherapy	≥ 0.2 mcg/kg/min
Dopamine monotherapy	≥ 10 mcg/kg/min
Phenylephrine monotherapy	≥ 200 mcg/min
Epinephrine monotherapy	≥ 0.1 mcg/kg/min
If on vasopressin	[1]Vasopressin + NE equivalent of ≥ 0.1mcg/kg/min
If on combination vasopressors (not vasopressin)	[1]Norepinephrine equivalent of ≥ 20 mcg/kg/min

[1]*VASST Trial Vasopressor Equivalent Equation:*

Norepinephrine equivalent dose = [norepinephrine (mcg/min)] + [dopamine (mcg/kg/min) ÷ 2] + [epinephrine (mcg/min)] + [phenylephrine (mcg/min) ÷10]

14.2.3 Encephalopathy assessment tools for grading Immune effector Cell-Associated Neurotoxicity Syndrome (ICANS)

14.2.3.1 Immune Effector Cell-associated Encephalopathy (ICE) Assessment

Directions: Answer whether each task was performed correctly (Not Done/Yes/No). If you answer YES put 1 in the Score column; If you answer NO (or Not Done) put 0 in the Score column.

Tasks	Performed correctly?	Score
**Orientation**		
1. What is the current year?		**0**
2. What is the current month?		**0**
3. What is the current city?		**0**
4. What hospital are you in?		**0**
**Naming**		
5. Name this object (*point to an object in the room)*		**0**
6. Name this object (*point to an object in the room)*		**0**
7. Name this object (*point to an object in the room)*		**0**
**Following commands**		
8. Show me (insert object, e.g. 2 fingers) or Close your eyes and stick out your tongue.		**0**
**Writing**		
9. Write a simple sentence (*provide paper and pencil)*		**0**
**Attention**		
10. Count backwards from 100 in 10's.		**0**
**Total Score**		

<u>Scoring ICE</u>

No impairment: Score 10

Grade 1 ICANS: Score 7-9

Grade 2 ICANS: Score 3-6

Grade 3 ICANS: Score 0-2

Grade 4 ICANS: Score 0 due to subject unarousable and unable to perform ICE assessment.

14.2.3.2 ASBMT Immune effector Cell-Associated Neurotoxicity Syndrome (ICANS) Consensus Grading for Adults

Neurotoxicity Domain	Grade 1	Grade 2	Grade 3	Grade 4
**ICE Score^**	7-9	3-6	0-2	0 (patient is unarousable and unable to perform ICE)
**Depressed level of consciousness□**	Awakens spontaneously	Awakens to voice	Awakens only to tactile stimulus	Patient is unarousable or requires vigorous or repetitive tactile stimuli to arouse. Stupor or coma
**Seizure**	N/A	N/A	Any clinical seizure focal or generalized that resolves rapidly; or<br>Non-convulsive seizures on EEG that resolve with intervention	Life-threatening prolonged seizure (> 5 min); or<br>Repetitive clinical or electrical seizures without return to baseline in between.
**Motor findings§**	N/A	N/A	N/A	Deep focal motor weakness such as hemiparesis or paraparesis
**Raised ICP / Cerebral edema**	N/A	N/A	Focal/local edema on neuroimaging#	Diffuse cerebral edema on neuroimaging;<br>Decerebrate or decorticate posturing; or Cranial nerve VI palsy; or Papilledema; or Cushing's triad

ICANS grade is determined by the most severe event (ICE score, level of consciousness, seizure, motor findings, raised ICP/cerebral edema) not attributable to any other cause. For example, a patient with and ICE score of 3 who has a generalized seizure is classified as having a Grade 3 ICANS.

^A patient with an ICE score of 0 may be classified as having Grade 3 ICANS if the patient is awake with global aphasia. But a patient with an ICE score of 0 may be classified as having a Grade 4 ICANS if the patient is unarousable.

□Depressed level of consciousness should be attributable to no other cause (e.g. no sedating medication).

§Tremors and myoclonus associated with immune effector cell therapies may be graded according to CTCAE v5.0 but they do not influence ICANS grading.

#Intracranial hemorrhage with or without associated edema is not considered a neurotoxicity feature and is excluded from ICANS grading. It may be graded according to CTCAE v5.0.

ICE: Immune effector Cell-associated Encephalopathy; **ICP:** Intracranial pressure; **EEG:** electroencephalogram

14.2.4 Neurologic Recommended Treatments by Grade

Grading Assessment	Recommended Treatments *
**Grade 1:**	☐ Notify MD<br>☐ Neurologic Consult<br>☐ Review for causes of confusion, i.e. polypharmacy, and avoid CNS depressive meds<br>☐ Neuro checks Q 2-4 h while awake<br>☐ Institute Aspiration precautions<br>☐ Ensure IV access<br>☐ EEG or ☐ MRI of brain<br>☐ Consider increasing dose of Keppra to 20 mg/kg, consider alternative anti-epileptics<br>☐ Consider tocilizumab in NS 8 mg/kg (if ≥ 30 kg) or 12 mg/kg (if < 30kg), IV if concurrent CRS (grade 2 or 3)
**Grade 2:**	☐ Include those above for Grade 1<br>☐ Elevate HOB > 30°<br>☐ VS Q 2-4h, notify MD immediately for bradycardia, hypertension or irregular respirations.<br>☐ Brain MRI (with or without contrast)<br>☐ Cardiac telemetry and continuous pulse oximetry<br>☐ Consider increasing Keppra dose to 1 GM IV BID<br>☐ Consider Steroids Dexamethasone 10mg q 6-12 hours (methylprednisolone 1 mg/kg IV Q12h; OR dexamethasone 0.1 mg/kg IV Q6h) or ICANS without CRS
**Grade 3:**	☐ Include those above for Grade 1&2<br>☐ Corticosteroids (☐ methyprednisolone 30 mg/kg IV Q24h X 3) for ICANS without CRS<br>☐ Tocilizumab at a dose of 8 mg/kg ≥ 30 kg or 12 mg/kg < 30 kg, infused over 1 hour - not to exceed 800 mg,<br>☐ ICU transfer if not previously done<br>☐ Evaluate for papilledema and treat elevated ICP if present<br>☐ Consider IT hydrocortisone[a]<br>☐ Assess need for airway protection
**Grade 4:**	☐ Include all those above<br>☐ Airway support and/or Mechanical ventilation, and all other supportive care measures<br>☐ Give high dose steroids: methylprednisolone 1 g or 30 mg/kg, daily x3 (MAX 1000 mg) with taper<br>☐ Evaluate for status epilepticus and treat accordingly

[a] IT hydrocortisone dosing will be calculated by patient age as follows [from Rieselbach, RE, et al. Subarachnoid distribution of drugs after lumbar injection; NEJM. 1962 Dec 20; 267: 1273-8.]:

Patient Age (years)	Doses (MTX/Hydrocortisone/ARAC)	Recommended Volume	10% CSF Volume	CSF Volme*
9 or greater	15 mg/ 15 mg / 30 mg	5-10 mL	13 mL	130 ± 30 mL (adults)

* **Please NOTE**: these are only recommendations and actual physician's plan of care must consider patient conditions, comorbidities, neurologist recommendations, etc.

14.3 Appendix C: Guidelines for Grading and Management of Suspected Cytokine Release Syndrome and Neurotoxicity – Dose Escalation Cohort

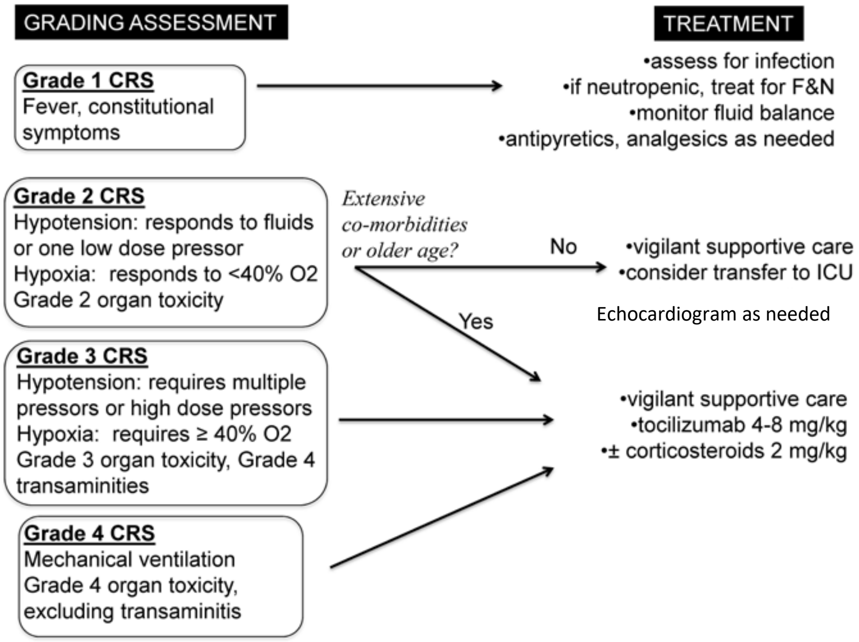

Table 1: Cytokine Release Syndrome Revised Grading System and Treatment

Grade 1	*Symptoms are not life threatening and require symptomatic treatment only*<br>e.g. fever, nausea, fatigue, headache, myalgias, malaise<br>Treatment :<br>✓ Assess for infection<br>✓ Treat fever and neutropenia if present, monitor fluid balance, antipyretics, analgesics as needed.
Grade 2	*Symptoms require and respond to moderate intervention*<br>-oxygen requirement < 40% or<br>-hypotension responsive to fluids or low dose[1] of one vasopressor or<br>-Grade 2 organ toxicity<br>Treatment :<br>✓ As above for Grade 1<br>✓ Monitor organ function closely (incl. cardiac, respiratory, renal, neurologic, liver)
Grade 3 or Grade 2 with co-morbidity	*Symptoms require and respond to aggressive intervention*<br>-oxygen requirement ≥ 40% or<br>-hypotension requiring high dose[1] or multiple vasopressors or<br>-Grade 3 organ toxicity or Grade 4 transaminitis<br>Treatment :<br>✓ As above for Grade 2<br>✓ Consider tocilizumab with or without corticosteroids<br>✓ Consider vasopressors, oxygen supplementation
Grade 4	*Life-threatening symptoms*<br>-requirement for ventilator support or<br>-Grade 4 organ toxicity (excluding transaminitis)<br>Treatment :

	✓ As above<br>✓ Mechanical ventilation<br>✓ Multiple vasopressors or high dose vasopressors

[1]high dose vasopressor doses shown in Table 2.

Suggested tocilizumab dose is 8 mg/kg (or subjects ≥ 30 kg) or 12 mg/kg (for subjects < 30 kg), not to exceed 800 mg per infusion, infused over 1 hour.

Table 2. High-Dose Vasopressors (all doses are required for ≥ 3 hours)

Norepinephrine monotherapy	≥ 0.20 mcg/kg/min
Dopamine monotherapy	≥ 10 mcg/kg/min
Phenylephrine monotherapy	≥ 200 mcg/kg/min
Epinephrine monotherapy	≥ 0.10 mcg/kg/min
If on vasopressin	[1]Vasopressin + NE equivalent of ≥ 0.10mcg/kg/min
If on combination vasopressors (not vasopressin)	[1]Norepinephrine equivalent of ≥ 0.20 mcg/kg/min

[1]*<u>VASST Trial Vasopressor Equivalent Equation:</u>*
Norepinephrine equivalent dose = [norepinephrine (mcg/min)] + [dopamine (mcg/kg/min) ÷ 2] + [epinephrine (mcg/min)] + [phenylephrine (mcg/min) ÷10]

Table 3: Neurotoxicity Assessment and Management Guidance

Neurotoxicity Grading assessment (CTCAE 4.03)	Treatment	Evaluation
**Grade 1: (consider CRES score)**<br>Examples include:<br>• Somnolence-mild drowsiness or sleepiness<br>• Confusion-mild disorientation<br>• Encephalopathy- mild limiting of ADL<br>• Dysphasia-not impairing ability to communicate<br>• Brief partial seizure; no loss of consciousness	☐ Notify MD<br>☐ Avoid CNS depressive meds<br>☐ Aspiration precautions<br>☐ Ensure IV access<br>☐ Consider increasing dose of Keppra, consider alternative antiepileptics<br>☐ Consider tocilizumab in NS 8 mg/kg IV if concurrent CRS grade 2-3 (see CRS Assessment)	Neuro checks Q 2-4 h, wake for neuro checks Neurologic Consult<br>EEG or MRI of brain<br>Review for causes of confusion, i.e. polypharmacy<br>Additional work up as clinically indicated.
**Grade 2: (consider CRES score)**<br>Examples include:<br>• Somnolence-moderate, limiting instrumental ADL<br>• Confusion-moderate disorientation, limiting instrumental ADL<br>• Encephalopathy- limiting	Include those above for Grade 1<br>☐ Vigilant supportive care<br>☐ Elevate HOB > 30°<br>☐ Consider ICU transfer if associated with grade ≥ 2 CRS	Brain MRI<br>Evaluation of CSF<br>Consider EEG as clinically indicated<br>Cardiac telemetry and continuous pulse oximetry<br>VS Q 2-4h, notify MD

instrumental ADL<br>• Dysphasia-moderate impairing ability to communicate spontaneously<br>• Brief generalized seizure	☐ Increase levetiracetam dose from 1 GMBID<br>☐Consider Steroids Dexamthasone 10mg q 6-12 hours	immediately for bradycardia, hypertension or irregular respirations.<br>Q4 hour neurological checks (wake the subject)
**Grade 3:**<br>Examples include:<br>• Somnolence-obtundation or stupor<br>• Confusion-severe disorientation, limiting self-care ADL<br>• Encephalopathy- limiting self-care ADL<br>• Dysphasia-severe receptive or expressive characteristics, impairing ability to read, write or communicate intelligibly<br>• Multiple seizures despite medical intervention<br>• Weakness limiting self-care ADL; disabling<br>• Complete bowel / bladder incontinence	Include those above for Grade 1<br>☐ Tocilizumab 8 mg/kg IV over 1 hour (not to exceed 800 mg), q 4-6 hours if symptoms have not stabilized or improved within 12-24 hours<br>Corticosteroids (☐ methyprednisolone 1 mg/kg IV Q12h; OR ☐ dexamethasone 10 mg IV Q6h) for CRES without CRS<br>Strongly consider transfer to ICU	As above for Grade 1 & 2<br>Evaluate for papilledema and treat elevated ICP if present
**Grade 4:**<br>Examples include:<br>• Life-threatening consequences<br>• Urgent intervention indicated<br>• Mechanical ventilation<br>• Life-threatening; prolonged repetitive seizures	As above plus:<br>Give methylprednisolone 1 g daily x3<br>Taper methylprednisolone 250 mg BID x2 days,<br>125 mg BID x2 days and 60 mg BID x2 days<br>Mechanical ventilation and other supportive measures	As above plus:<br>Evaluate for status epilepticus and treat accordingly

14.4 Appendix D: Calculation of Weight for Cell Dose Calculation in Morbidly Obese Candidates

Formulation for deriving the weight to be used in targeting cell doses in morbidly obese cell candidates.

1. **Definition**

Obesity is defined as a BMI > 30.

BMI = wgt (kg) / [hgt (M)]2

2. **Calculation of ideal body weight** is performed using the standard, published formula:

Male: 50 + 2.3(Hgt - 60) where Hgt is in inches, and the result is expressed in kg.

ex. The ideal weight of a 5'10" male = 50 + 2.3(10) = 73 kg.

Female: 45.5 + 2.3(Hgt - 60), where height is in inches, and the result is in kg.

3. **Calculation of the "practical weight."**

Calculate the midway point, halfway between the actual and ideal body weights (ie the average of the two numbers). This is the "practical weight" to be used in calculating the targeted cell dose.

4. **Example**:

Subject's actual weight = 143 kg.

Subject' s actual height 173 cm = 69 in

BMI = 48

IBW formula = 50 + 2.3(9) = 70.7 kg

Midway point between 70.0 and 143 = 107 kg.

The weight we would use in targeting cell dose is 107 kg.

1 dose by weight with adjustment:
- IBW + 50%(Weight-IBW)
- Practical body weight = (IDW + actual BW)/2

2 Formula

IBW (men)

- 52 kg + 1.9 kg/inch above 5 feet
- 50 kg + 2.39 (height in inches – 60)

IBW (women)

- 49 kg + 1.7 kg/inch above 5 feet
- 45.5 kg + 2.39 (height in inches – 60)

14.5 APPENDIX E: RESPONSE CRITERIA

For the purposes of this study, subjects should be re-evaluated for response as outlined in Section 9. In addition to a baseline scan, confirmatory scans should also be obtained at least 4 weeks following initial documentation of objective response.

14.5.1 Response Criteria Lymphoma (From: Cheson BD, et al : Recommendations for Initial Evaluation, Staging, and Response Assessment of Hodgkin and Non-Hodgkin Lymphoma: The Lugano Classification. J Clin Oncol 2014 ; 32 :3059-3067)

National Comprehensive Cancer Network®

NCCN Guidelines Version 1.2017
B-cell Lymphomas

NCCN Guidelines Index
Table of Contents
Discussion

LUGANO RESPONSE CRITERIA FOR NON-HODGKIN'S LYMPHOMA

PET should be done with contrast-enhanced diagnostic CT and can be done simultaneously or at separate procedures.

Response	Site	PET-CT (Metabolic response)	CT (Radiologic response)[d]
Complete response	Lymph nodes and extralymphatic sites	Score 1, 2, or 3[a] with or without a residual mass on 5 point scale (5-PS)[b,c]	All of the following:<br>Target nodes/nodal masses must regress to ≤1.5 cm in longest transverse diameter of a lesion (LDi)<br>No extralymphatic sites of disease
	Non-measured lesion	Not applicable	Absent
	Organ enlargement	Not applicable	Regress to normal
	New Lesions	None	None
	Bone Marrow	No evidence of FDG-avid disease in marrow	Normal by morphology; if indeterminate, and flow cytometry IHC negative
Partial response	Lymph nodes and extralymphatic sites	Score 4 or 5[b] with reduced uptake compared with baseline. No new or progressive lesions.<br>At interim these findings suggest responding disease.<br>At end of treatment these findings may indicate residual disease.	All of the following:<br>≥50% decrease in SPD of up to 6 target measurable nodes and extranodal sites<br>When a lesion is too small to measure on CT, assign 5mm x 5mm as the default value.<br>When no longer visible, 0x0 mm<br>For a node >5mm x 5mm, but smaller than normal, use actual measurement for calculation
	Non-measured lesion	Not applicable	Absent/normal, regressed, but no increase
	Organ enlargement	Not applicable	Spleen must have regressed by >50% in length beyond normal
	New Lesions	None	None
	Bone Marrow	Residual uptake higher than uptake in normal marrow but reduced compared with baseline (diffuse uptake compatible with reactive changes from chemotherapy allowed). If there are persistent focal changes in the marrow in the context of a nodal response, consider further evaluation with biopsy, or an interval scan.	Not applicable

Reprinted with permission. © 2014 American Society of Clinical Oncology. All rights reserved. Cheson B, Fisher R, Barrington S, et al. Recommendations for Initial Evaluation, Staging and Response Assessment of Hodgkin and Non-Hodgkin Lymphoma – the Lugano Classification. J Clin Oncol 2014;32:3059-3067.

Footnotes on NHODG-C 3 of 3

Continued on next page

Note: All recommendations are category 2A unless otherwise indicated.
Clinical Trials: NCCN believes that the best management of any patient with cancer is in a clinical trial. Participation in clinical trials is especially encouraged.

Version 1.2017, 12/07/16 © National Comprehensive Cancer Network, Inc. 2016, All rights reserved. The NCCN Guidelines® and this illustration may not be reproduced in any form without the express written permission of NCCN®.

NHODG-C
1 OF 3

LUGANO RESPONSE CRITERIA FOR NON-HODGKIN'S LYMPHOMA

PET should be done with contrast-enhanced diagnostic CT and can be done simultaneously or at separate procedures.

Response	Site	PET-CT (Metabolic response)	CT (Radiologic response)[d]
No response or stable disease	Target nodes/nodal masses, extranodal lesions	Score 4 or 5[b] with no significant change in FDG uptake from baseline at interim or end of treatment. No new or progressive lesions	<50% decrease from baseline in SPD of up to 6 dominant, measurable nodes and extranodal sites; no criteria for progressive disease are met
	Non-measured lesion	Not applicable	No increase consistent with progression
	Organ enlargement	Not applicable	No increase consistent with progression
	New Lesions	None	None
	Bone Marrow	No change from baseline	Not applicable
Progressive disease	Individual target nodes/nodal masses<br>Extranodal lesions	Score 4 or 5[b] with an increase in intensity of uptake from baseline<br>and/or<br>New FDG-avid foci consistent with lymphoma at interim or end-of-treatment assessment[e]	Requires at least one of the following<br>PPD progression:<br>An individual node/lesion must be abnormal with:<br>LDi >1.5 cm and<br>Increase by ≥50% from PPD nadir and<br>An increase in LDi or SDi from nadir<br>0.5 cm for lesions ≤2 cm<br>1.0 cm for lesions >2 cm<br>In the setting of splenomegaly, the splenic length must increase by >50% of the extent of its prior increase beyond baseline. If no prior splenomegaly, must increase by at least 2 cm from baseline<br>New or recurrent splenomegaly
	Non-measured lesion	None	New or clear progression of preexisting nonmeasured lesions
	New Lesions	New FDG-avid foci consistent with lymphoma rather than another etiology (eg, infection, inflammation). If uncertain regarding etiology of new lesions, biopsy or interval scan may be considered[e]	Regrowth of previously resolved lesions<br>A new node >1.5 cm in any axis<br>A new extranodal site >1.0 cm in any axis; if <1.0 cm in any axis, its presence must be unequivocal and must be attributable to lymphoma<br>Assessable disease of any size unequivocally attributable to lymphoma
	Bone Marrow	New or recurrent FDG-avid foci	New or recurrent involvement

Reprinted with permission. © 2014 American Society of Clinical Oncology. All rights reserved. Cheson B, Fisher R, Barrington S, et al. Recommendations for Initial Evaluation, Staging and Response Assessment of Hodgkin and Non-Hodgkin Lymphoma – the Lugano Classification. J Clin Oncol 2014;32:3059-3067.

Footnotes on NHODG-C 3 of 3

Continued on next page

Note: All recommendations are category 2A unless otherwise indicated.
Clinical Trials: NCCN believes that the best management of any patient with cancer is in a clinical trial. Participation in clinical trials is especially encouraged.

Version 1.2017, 12/07/16 © National Comprehensive Cancer Network, Inc. 2016, All rights reserved. The NCCN Guidelines® and this illustration may not be reproduced in any form without the express written permission of NCCN®.

NHODG-C
2 OF 3

LUGANO RESPONSE CRITERIA FOR NON-HODGKIN'S LYMPHOMA

Footnotes

[a]Score 3 in many patients indicates a good prognosis with standard treatment, especially if at the time of an interim scan. However, in trials involving PET where de-escalation is investigated, it may be preferable to consider score 3 as an inadequate response (to avoid under-treatment).
[b]See PET Five Point Scale (5-PS).
[c]It is recognized that in Waldeyer's ring or extranodal sites with high physiological uptake or with activation within spleen or marrow, e.g. with chemotherapy or myeloid colony stimulating factors, uptake may be greater than normal mediastinum and/or liver. In this circumstance, CMR may be inferred if uptake at sites of initial involvement is no greater than surrounding normal tissue even if the tissue has high physiological uptake.
[d]FDG-avid lymphomas should have response assessed by PET-CT. Diseases that can typically be followed with CT alone include CLL/SLL and marginal zone lymphomas.
[e]False-positive PET scans may be observed related to infectious or inflammatory conditions. Biopsy of affected sites remains the gold standard for confirming new or persistent disease at end of therapy.

PET Five Point Scale (5-PS)

1 **No uptake above background**
2 **Uptake ≤ mediastinum**
3 **Uptake > mediastinum but ≤ liver**
4 **Uptake moderately > liver**
5 **Uptake markedly higher than liver and/or new lesions**
X **New areas of uptake unlikely to be related to lymphoma**

SPD – sum of the product of the perpendicular diameters for multiple lesions
LDi – Longest transverse diameter of a lesion
SDi – Shortest axis perpendicular to the LDi
PPD – Cross product of the LDi and perpendicular diameter

Measured dominant lesions – Up to 6 of the largest dominant nodes, nodal masses and extranodal lesions selected to be clearly measurable in 2 diameters. Nodes should preferably be from disparate regions of the body, and should include, where applicable, mediastinal and retroperitoneal areas. Non-nodal lesions include those in solid organs, e.g., liver, spleen, kidneys, lungs, etc, gastrointestinal involvement, cutaneous lesions of those noted on palpation.
Non-measured lesions – Any disease not selected as measured, dominant disease and truly assessable disease should be considered not measured. These sites include any nodes, nodal masses, and extranodal sites not selected as dominant, measurable or which do not meet the requirements for measurability, but are still considered abnormal. As well as truly assessable disease which is any site of suspected disease that would be difficult to follow quantitiatively with measurement, including pleural effusions, ascites, bone lesions, leptomeningeal disease, abdominal masses and other lesions that cannot be confirmed and followed by imaging.

Reprinted with permission. © 2014 American Society of Clinical Oncology. All rights reserved. Cheson B, Fisher R, Barrington S, et al. Recommendations for Initial Evaluation, Staging and Response Assessment of Hodgkin and Non-Hodgkin Lymphoma – the Lugano Classification. J Clin Oncol 2014;32:3059-3067.

Note: All recommendations are category 2A unless otherwise indicated.
Clinical Trials: NCCN believes that the best management of any patient with cancer is in a clinical trial. Participation in clinical trials is especially encouraged.

Version 1.2017, 12/07/16 © National Comprehensive Cancer Network, Inc. 2016, All rights reserved. The NCCN Guidelines® and this illustration may not be reproduced in any form without the express written permission of NCCN®.

NHODG-C
3 OF 3

14.5.2 Response Criteria for ALL

*Modified from: Cheson BD, et al. Revised recommendations of the International Working Group for diagnosis, standardization of response criteria, treatment outcomes, and reporting standards for therapeutic trials in acute myeloid leukemia. J Clin Oncol 2003;21:4642-4649

Bone Marrow Classification

	**%** blasts (at least 200 cells counted)
M1	< 5%
M2	5 - 25%
M3	> 25%

- Complete Response (CR)
  1. M1 marrow, absence of peripheral blasts (morphologic), absence of extramedullary sites of disease, peripheral blood neutrophil count ≥ 1,000/μL and platelet count ≥ 100,000/μL. This parameter will be the requisite criterion for CR. The following additional parameters will be reported as exploratory findings.
  2. Morphologic CR with incomplete blood count recovery (CRi): Above CR criteria without specified blood counts.
  3. Cytogenetic CR (CR_{cyto}): In addition to above CR criteria, reversion to normal karyotype for those with previously detected cytogenetic abnormality.
  4. Molecular CR (CR_{molec}): In addition to above CRc criteria, normalization of previously detected molecular cytogenetic abnormality.
- Partial Response (PR)
  1. M2 marrow and a decrease in the percentage of marrow blasts by at least 50%, absence of peripheral blasts (morphologic), absence of extramedullary sites of disease.
- Hematological Activity (HA)

  Does not meet the criteria for CR or PR with any of the following**:**
  1. At least a 50% decrease in the percentage of marrow blasts
  2. At least a 50% decrease in the absolute peripheral blast count
  3. Improvement of the peripheral blood neutrophil count to ≥ 1,000/μL or platelet count to ≥ 100,000/μL
- Stable Disease (SD)
  1. Does not meet the criteria for CR, PR, HA, or PD

- Progressive Disease (PD)
  1. Worse marrow classification (i.e., M status) with at least a 50% increase in the percentage of marrow blasts.

     Or
  2. No change in marrow classification (i.e., M status), but a 50% or greater increase in absolute peripheral blast count or extent of extramedullary disease
- CNS Classification

  CNS is the most common site of extramedullary disease in ALL. The following table lists the CNS disease classification. This classification should be used when evaluating the subject’s overall response to treatment.

CNS Classification

	CSF Cell Count and Cytology
CNS 1	0 blasts on cytospin
CNS 2	< 5/µl WBCs, cytospin positive for blasts<br>or<br>Traumatic spinal tap with ≥ 10/µL RBCs, WBC ≥ 5/µL, cytospin positive for blasts but negative by Steinherz/Bleyer algorithm
CNS 3	≥ 5/µl WBCs, cytospin positive for blasts<br>or<br>Traumatic spinal tap with ≥ 10/µL RBCs, cytospin positive for blasts, and positive Steinherz/Bleyer algorithm*

***Steinherz/Bleyer algorithm method of evaluating traumatic lumbar punctures:**

If the subject has leukemic cells in the peripheral blood and the lumbar puncture is traumatic and contains ≥ 5 WBC/µL and blasts, the following algorithm should be used to distinguish between CNS2 and CNS3 disease:

CSF WBC/RBC > 2X Blood WBC/RBC

14.6 APPENDIX F: MONITORING GENE THERAPY TRIALS: REPLICATION COMPETENT LENTIVIRUS (RCL)

Subject Testing for RCL PCR

Subject blood samples will be obtained at the following time points:

1. Prior to cell infusion
2. 3 months(± 1 month)
3. 6 months (± 1 month)
4. 12 months (± 2 months)
5. Archive samples annually thereafter for an additional 4 years (± 2 months), if previous samples have been negative.

All Samples are scheduled from the most recent cell infusion, and should proceed even after disease progression.

Procedures:

Samples will be collected from subjects returning to clinic or, at investigator discretion, may be collected by a local medical provider. Samples will be shipped directly to Indiana University GTTL, as described in Stanford Center for Cancer Cell Therapy SOP 'Remote RCR/RCL Collection'. Procedures in brief include:

1. 4 mL blood sample collected in EDTA tube (lavender/purple top). If necessary, sample may be collected as 2 x 2 mL EDTA tubes.
2. Sample will be packaged according to biohazardous shipping regulation (UN 3373, 'Diagnostic Specimens') and shipped on ice priority overnight to Indiana University GTTL.

   Attn: Lisa Duffy
   IU GTTL
   980 West Walnut St, R3-C668
   Indianapolis, IN 46202-5188
   Phone: 317-274-0323

   a. 'NGVB Sample Submission Form' and 'Sample Submission Form Master Agreements' must accompany sample for proper processing
   b. Sample shipment must occur Monday-Thursday, as IU GTTL is unable to accept samples over the weekend or holidays
3. Notify IU GTTL representatives by email when sample delivery is expected.

Document Retention

1. RCL Reports from Indiana University will be sent to the Sponsor Investigator or designated representative; who will distribute the results. Groups requesting RCL results notification include:
   a. Principal Investigator and co-investigators
   b. IND Sponsor, Crystal Mackall
   c. CCT-Clinical Research Operations & Procedures (CCT-CROP) [regulatory support]
   d. CCT Manufacturing team
2. Hard copies will be retained in the subject's research record or other suitable study file in a locked file cabinet.

14.7 Appendix G: Draft Letter and Questionnaire to Subjects for Long Term Follow-up for Delayed Adverse Events

[date]

[name and address]

Dear [subject name],

You have participated in a clinical research study that requires that the study doctors and nurses monitor your health for 15 years. In addition to the annual visits you will be attending, **we would like for you to report certain events listed below to your study doctor or nurse if they occur**:

1. Your doctor tells you that you have been diagnosed with any new type of cancer, including blood disorders such as leukemia or lymphoma (this would be separate from your cancer diagnosis).
2. You develop loss of feeling in any part of your body, especially hands and feet; you develop a loss of control of any body part (arms, legs…); you have a seizure; you experience memory loss. In addition, if you experience a worsening of any of the symptoms listed, please contact your study nurse or doctor. These types of symptoms are called neurological disorders. If your primary doctor or specialist tells you that you have developed neurological symptoms, contact your study doctor or nurse.
3. You develop arthritis or autoimmune disease, or worsening of any previously experienced arthritis or autoimmune disease which you were experiencing prior to participation in the study. If you are experiencing symptoms of arthritis or have been told by your doctor that you have an autoimmune disease, contact your study doctor or nurse.

Please complete the attached questionnaire and return it in the Fed-Ex envelope to the study coordinator.

If you experience any of the events listed above during the upcoming year, please contact your study physician or the study nurse listed below as soon as you can. They may ask you questions about your health and will record your symptoms/disease and then monitor your health if they decide that it is necessary. When you call, please mention that you participated in a gene therapy clinical trial at the < put your institution here> . Your subject identification number under this protocol is (#XXX).

Study Coordinator:
Name
Address
Phone Email

If you have any questions about this letter or the follow up procedures for the study itself, please do not hesitate to contact the above study nurse.

Thank you for your continued participation in our clinical research study. Best regards,

[study coordinator]

Questionnaire to Subjects for Long Term Follow-up for Delayed Adverse Events

Subject Identification [put subject study number here]

Within the past year, have you:

1. **Had any problems with your health?** ☐ YES ☐ NO

If Yes, please explain:____

____

2. **Required any hospitalizations?** ☐ YES ☐ NO

If Yes, please describe when and the reason:__________________________

____

____

3. **Seen any healthcare provider?** ☐ YES ☐ NO

If Yes, please describe when and the reason:__________________________

____

____

4. **Started on any new medications?** ☐ YES ☐ NO

If Yes, please list: ____

____

5. **Developed any new conditions or illnesses?** ☐ YES ☐ NO

If Yes, please describe:____

____

____

Please share any other new health concerns or problems:

____

____

When you have completed this questionnaire, please return it to:

Study Coordinator:
Name
Address Phone
Fax Email

A pre-addressed stamped envelope has been enclosed for your convenience, if you choose to mail this questionnaire. We will also accept faxed or e-mailed completed questionnaires as well.

Thank you very much for your participation.

14.8 Appendix H: Physician (Local Medical Provider) Letter

[date]

[name and address]

Dear [physician name],

Your subject [subject name] has participated in a clinical research study that requires 15 year monitoring for adverse events. To aid in reporting adverse events that are possible related to the clinical research study, we are asking the subjects on our research study to designate a primary care or infectious disease physician that may help in the monitoring and reporting of adverse events. Your subject has designated you. **If upon any of your visits with your subject, any of the following events are reported or discovered, please contact the study nurse or physician as soon as possible**:

1. New malignancies
2. New incidence or exacerbation of a pre-existing neurologic disorder
3. New incidence or exacerbation of a prior rheumatologic or other autoimmune disorder
4. New incidence of a hematologic disorder.

If your subject experiences any of these events, please contact the study coordinator below as soon as you can so that they can record the event and then monitor your subject's health if necessary. When you call, please mention that the subject has participated in a gene therapy clinical trial in the < designate location, institution and investigator> .

Study Coordinator
Name
Address
Phone
Email

If you have any questions about this letter or the study itself, please do not hesitate to contact the above study nurse.

Thank you for your support in helping us to monitor for delayed adverse events. Best regards,

14.9 APPENDIX I: CORRELATIVE SAMPLE SCHEDULE

Correlative Sample Collection Matrix

mL	type	purpose	Apheresis	Baseline (prior to LD chemo)	D0	D3 (± 2 d)	D7 (± 2 d)	D10 (± 2 d)	D14 (± 2 d)	D21 (± 4 d)	D28 (± 7 d)	M2 (± 2 w)	M3 (± 4 w)	M4 (± 2 w)	M5 (± 2 w)	M6 (± 1 m)	M9 (± 1 m)	M12 (± 2 m)	At Progression
**Apheresis Product**																			
	collected by manufacturer		X																
	collected by Stanford										X[1]		X[1]			X[1]	X[1]	X[1]	
**CAR T cell Product**																			
	collected by manufacturer	baseline measurements for correlatives			X														
**PBMCs**																			
10	Green top (Heparin)	Flow panel (CAR-FACS)[4]		X		X	X	X	X	X	X	X	X	X	X	X	X	X	X
10	Green top (Heparin)	CyTOF[4]		X		X	X	X	X	X	X	X	X	X	X	X	X	X	X
10	Lavender top (EDTA)	CAR qRT-PCR[4]			X	X	X[2]	X	X	X	X	X	X[2]	X	X	X[2]	X	X[2]	X[2]
10	Lavender top (EDTA)	TCR sequencing/ CAR-T cell fate		X			X						X			X		X	X
5	Lavender top (EDTA)	ATAC-Seq		X							X		X			X		X	X
10	Green top (Heparin)	Sample banking			X	X	X	X	X	X	X	X	X	X	X	X	X	X	X
**Plasma**																			
4	Lavender top (EDTA)	Cytokines - Luminex (HIMC)[4]			X	X	X	X	X	X	X								
4	Lavender top (EDTA)	MRD - DLBCL			X		X		X	X	X	X	X			X	X	X	
**Bone Marrow Aspirate**																			
2	Green top (Heparin)	Flow panel (CAR-FACS)[4]		X							X		X			X			X
5	Green top (Heparin)	CyTOF[4]		X							X		X			X			X
5	Lavender top (EDTA)	TCR sequencing/ CAR-T cell fate		X							X		X			X			X
5	Lavender top (EDTA)	ATAC-Seq		X							X		X			X			X
2	Green top (Heparin)	Sample banking		X							X		X			X			X
**Lymph Node Biopsy**																			
	CT guided: 4 cores collected in RPMI			X[3]							X[3]								X[3]
	CT guided: 4 cores collected in hydrothermacil			X[3]							X[3]								X[3]
	CT guided: 4 tubes FNA product			X[3]							X[3]								X[3]
	Not CT guided: 6 tubes FNA product			X[3]							X[3]								X[3]
**Lumbar Puncture/CSF**																			
1	Plastic Falcon tube			X							X[5]								X
**Total Blood Volume per visit**																			
				35	28	44	48	44	48	48	53	44	49	40	40	49	44	49	45

Notes
1. A small volume apheresis (1-2 blood volumes) may be collected in lieu of PBMC blood tubes listed below
2. Sample collected used for both CAR qRT-PCR and TCR sequencing
3. Either CT guided or not CT guided will be collected. One sample between Day 7 to 28 at peak CAR activity
4. Stop further collection if CAR is undetectable
5. Collect at any time of neurotoxicity and at Day 28 (for subjects with CNS at baseline), at discretion of investigator

15 REFERENCES

1. Kantarjian, H.M., et al., *Defining the course and prognosis of adults with acute lymphocytic leukemia in first salvage after induction failure or short first remission duration.* Cancer, 2010. **116**(24): p. 5568-74.
2. Tavernier, E., et al., *Outcome of treatment after first relapse in adults with acute lymphoblastic leukemia initially treated by the LALA-94 trial.* Leukemia, 2007. **21**(9): p. 1907-14.
3. Gokbuget, N., et al., *Adult patients with acute lymphoblastic leukemia and molecular failure display a poor prognosis and are candidates for stem cell transplantation and targeted therapies.* Blood, 2012. **120**(9): p. 1868-76.
4. Gisselbrecht, C., et al., *Salvage regimens with autologous transplantation for relapsed large B-cell lymphoma in the rituximab era.* J Clin Oncol, 2010. **28**(27): p. 4184-90.
5. Davila, M.L., et al., *Efficacy and toxicity management of 19-28z CAR T cell therapy in B cell acute lymphoblastic leukemia.* Sci Transl Med, 2014. **6**(224): p. 224ra25.
6. Maude, S.L., et al., *Chimeric antigen receptor T cells for sustained remissions in leukemia.* N Engl J Med, 2014. **371**(16): p. 1507-17.
7. Lee, D.W., et al., *T cells expressing CD19 chimeric antigen receptors for acute lymphoblastic leukaemia in children and young adults: a phase 1 dose-escalation trial.* Lancet, 2015. **385**(9967): p. 517-28.
8. Kochenderfer, J.N., et al., *Chemotherapy-refractory diffuse large B-cell lymphoma and indolent B-cell malignancies can be effectively treated with autologous T cells expressing an anti-CD19 chimeric antigen receptor.* J Clin Oncol, 2015. **33**(6): p. 540-9.
9. Long, A.H., et al., *4-1BB costimulation ameliorates T cell exhaustion induced by tonic signaling of chimeric antigen receptors.* Nat Med, 2015. **21**(6): p. 581-90.
10. Grupp, S.A., et al., *Chimeric antigen receptor-modified T cells for acute lymphoid leukemia.* N Engl J Med, 2013. **368**(16): p. 1509-18.
11. Gardner, R., et al., *Acquisition of a CD19-negative myeloid phenotype allows immune escape of MLL-rearranged B-ALL from CD19 CAR-T-cell therapy.* Blood, 2016. **127**(20): p. 2406-10.
12. Nitschke, L., *The role of CD22 and other inhibitory co-receptors in B-cell activation.* Curr Opin Immunol, 2005. **17**(3): p. 290-7.
13. Nitschke, L., *CD22 and Siglec-G: B-cell inhibitory receptors with distinct functions.* Immunol Rev, 2009. **230**(1): p. 128-43.
14. Nitschke, L., *CD22 and Siglec-G regulate inhibition of B-cell signaling by sialic acid ligand binding and control B-cell tolerance.* Glycobiology, 2014. **24**(9): p. 807-17.
15. Bendall, S.C., et al., *Single-cell trajectory detection uncovers progression and regulatory coordination in human B cell development.* Cell, 2014. **157**(3): p. 714-25.
16. Tedder, T.F., J.C. Poe, and K.M. Haas, *CD22: a multifunctional receptor that regulates B*

lymphocyte survival and signal transduction. Adv Immunol, 2005. **88**: p. 1-50.

17. Olejniczak, S.H., et al., *A quantitative exploration of surface antigen expression in common B-cell malignancies using flow cytometry.* Immunol Invest, 2006. **35**(1): p. 93-114.
18. Raponi, S., et al., *Flow cytometric study of potential target antigens (CD19, CD20, CD22, CD33) for antibody-based immunotherapy in acute lymphoblastic leukemia: analysis of 552 cases.* Leuk Lymphoma, 2011. **52**(6): p. 1098-107.
19. Haso, W., et al., *Anti-CD22-chimeric antigen receptors targeting B-cell precursor acute lymphoblastic leukemia.* Blood, 2013. **121**(7): p. 1165-74.
20. Shah, N.N., et al., *Characterization of CD22 expression in acute lymphoblastic leukemia.* Pediatr Blood Cancer, 2015. **62**(6): p. 964-9.
21. Chevallier, P., et al., *Simultaneous study of five candidate target antigens (CD20, CD22, CD33, CD52, HER2) for antibody-based immunotherapy in B-ALL: a monocentric study of 44 cases.* Leukemia, 2009. **23**(4): p. 806-7.
22. Polson, A.G., et al., *Anti-CD22-MCC-DM1: an antibody-drug conjugate with a stable linker for the treatment of non-Hodgkin's lymphoma.* Leukemia, 2010. **24**(9): p. 1566-73.
23. Huang, J., et al., *Diagnostic usefulness of aberrant CD22 expression in differentiating neoplastic cells of B-Cell chronic lymphoproliferative disorders from admixed benign B cells in four-color multiparameter flow cytometry.* Am J Clin Pathol, 2005. **123**(6): p. 826-32.
24. Cheson, B.D., et al., *Recommendations for initial evaluation, staging, and response assessment of Hodgkin and non-Hodgkin lymphoma: the Lugano classification.* J Clin Oncol, 2014. **32**(27): p. 3059-68.
25. Smith, M.A., et al., *Declining childhood and adolescent cancer mortality.* Cancer, 2014. **120**(16): p. 2497-506.
26. Muffly, L. and R.A. Larson, *Improving outcomes in childhood T-cell acute lymphoblastic leukemia: promising results from the Children's Oncology Group incorporating nelarabine into front-line therapy.* Transl Pediatr, 2012. **1**(2): p. 120-2.
27. Day, C.A., et al., *Development of immunity following financial incentives for hepatitis B vaccination among people who inject drugs: A randomized controlled trial.* J Clin Virol, 2016. **74**: p. 66-72.
28. Fielding, A.K., et al., *Outcome of 609 adults after relapse of acute lymphoblastic leukemia (ALL); an MRC UKALL12/ECOG 2993 study.* Blood, 2007. **109**(3): p. 944-50.
29. Fielding, A.K. and A.H. Goldstone, *Allogeneic haematopoietic stem cell transplant in Philadelphia-positive acute lymphoblastic leukaemia.* Bone Marrow Transplant, 2008. **41**(5): p. 447-53.
30. Topp, M.S., et al., *Safety and activity of blinatumomab for adult patients with relapsed or refractory B-precursor acute lymphoblastic leukaemia: a multicentre, single-arm, phase 2 study.* Lancet Oncol, 2015. **16**(1): p. 57-66.
31. Topp, M.S., et al., *Phase II trial of the anti-CD19 bispecific T cell-engager blinatumomab shows hematologic and molecular remissions in patients with relapsed or refractory B-*

precursor acute lymphoblastic leukemia. J Clin Oncol, 2014. **32**(36): p. 4134-40.

32. Braig, F., et al., *Resistance to anti-CD19/CD3 BiTE in acute lymphoblastic leukemia may be mediated by disrupted CD19 membrane trafficking.* Blood, 2017. **129**(1): p. 100-104.
33. Fisher, S.G. and R.I. Fisher, *The epidemiology of non-Hodgkin's lymphoma.* Oncogene, 2004. **23**(38): p. 6524-34.
34. Martelli, M., et al., *Diffuse large B-cell lymphoma.* Crit Rev Oncol Hematol, 2013. **87**(2): p. 146-71.
35. Siegel, R., D. Naishadham, and A. Jemal, *Cancer statistics, 2012.* CA Cancer J Clin, 2012. **62**(1): p. 10-29.
36. Coiffier, B., et al., *CHOP chemotherapy plus rituximab compared with CHOP alone in elderly patients with diffuse large-B-cell lymphoma.* N Engl J Med, 2002. **346**(4): p. 235-42.
37. Pfreundschuh, M., et al., *CHOP-like chemotherapy with or without rituximab in young patients with good-prognosis diffuse large-B-cell lymphoma: 6-year results of an open-label randomised study of the MabThera International Trial (MInT) Group.* Lancet Oncol, 2011. **12**(11): p. 1013-22.
38. Coiffier, B., et al., *Long-term outcome of patients in the LNH-98.5 trial, the first randomized study comparing rituximab-CHOP to standard CHOP chemotherapy in DLBCL patients: a study by the Groupe d'Etudes des Lymphomes de l'Adulte.* Blood, 2010. **116**(12): p. 2040-5.
39. Friedberg, J.W., *Relapsed/refractory diffuse large B-cell lymphoma.* Hematology Am Soc Hematol Educ Program, 2011. **2011**: p. 498-505.
40. Larouche, J.F., et al., *Lymphoma recurrence 5 years or later following diffuse large B-cell lymphoma: clinical characteristics and outcome.* J Clin Oncol, 2010. **28**(12): p. 2094-100.
41. Van Den Neste, E., et al., *Outcomes of diffuse large B-cell lymphoma patients relapsing after autologous stem cell transplantation: an analysis of patients included in the CORAL study.* Bone Marrow Transplant, 2017. **52**(2): p. 216-221.
42. Colosia, A., et al., *Clinical efficacy and safety in relapsed/refractory diffuse large B-cell lymphoma: a systematic literature review.* Clin Lymphoma Myeloma Leuk, 2014. **14**(5): p. 343-355 e6.
43. Crump, M., Neelapu, S.S., Farooq, U., Van Den Neste, E., et al. . *Outcomes in refractory aggressive diffuse large b-cell lymphoma (DLBCL): Results from the international SCHOLAR-1 study*. in *ASCO Annual Meeting*. 2016. Chicago, IL: Journal of Clinical Oncology.
44. Costa, L.J., Reddy, N., Maddocks, K.J., Epperla, N., Karmali, R., Bachanova, V., Umyarova, E., Zhou, Z., Glenn, M., et al. *Primary Failure Diffuse Large B Cell Lymphoma: Early Autologous or Donor Hematopoietic Cell Transplantation Not Effective in Patients with Ultra-High Risk Features*. in *American Society of Hematology.* 2016. San Diego, CA: American Society of Hematology.
45. Wang, K., G. Wei, and D. Liu, *CD19: a biomarker for B cell development, lymphoma diagnosis and therapy.* Exp Hematol Oncol, 2012. **1**(1): p. 36.
46. Kochenderfer, J.N., et al., *Eradication of B-lineage cells and regression of lymphoma in a*

patient treated with autologous T cells genetically engineered to recognize CD19. Blood, 2010. **116**(20): p. 4099-102.

47. Kalos, M., et al., *T cells with chimeric antigen receptors have potent antitumor effects and can establish memory in patients with advanced leukemia.* Sci Transl Med, 2011. **3**(95): p. 95ra73.

48. Kochenderfer, J.N., et al., *B-cell depletion and remissions of malignancy along with cytokine-associated toxicity in a clinical trial of anti-CD19 chimeric-antigen-receptor-transduced T cells.* Blood, 2012. **119**(12): p. 2709-20.

49. Mackall, C.L., T.J. Fry, and R.E. Gress, *Harnessing the biology of IL-7 for therapeutic application.* Nat Rev Immunol, 2011. **11**(5): p. 330-42.

50. Lee, D.W., et al., *Current concepts in the diagnosis and management of cytokine release syndrome.* Blood, 2014. **124**(2): p. 188-95.

51. Weber, J., et al., *A phase I trial of intravenous interleukin-6 in patients with advanced cancer.* J Immunother Emphasis Tumor Immunol, 1994. **15**(4): p. 292-302.

52. Sriaroon, P. and M. Ballow, *Immunoglobulin Replacement Therapy for Primary Immunodeficiency.* Immunol Allergy Clin North Am, 2015. **35**(4): p. 713-30.

53. Spadaro, G., et al., *Intravenous versus subcutaneous immunoglobulin replacement in secondary hypogammaglobulinemia.* Clin Immunol, 2016. **166-167**: p. 103-4.

54. Borowitz, M.J., et al., *Clinical significance of minimal residual disease in childhood acute lymphoblastic leukemia and its relationship to other prognostic factors: a Children's Oncology Group study.* Blood, 2008. **111**(12): p. 5477-85.

55. Borowitz, M.J., et al., *Prognostic significance of minimal residual disease in high risk B-ALL: a report from Children's Oncology Group study AALL0232.* Blood, 2015. **126**(8): p. 964-71.

56. Pui, C.H., et al., *Clinical utility of sequential minimal residual disease measurements in the context of risk-based therapy in childhood acute lymphoblastic leukaemia: a prospective study.* Lancet Oncol, 2015. **16**(4): p. 465-74.

57. Topp, M.S., et al., *Targeted therapy with the T-cell-engaging antibody blinatumomab of chemotherapy-refractory minimal residual disease in B-lineage acute lymphoblastic leukemia patients results in high response rate and prolonged leukemia-free survival.* J Clin Oncol, 2011. **29**(18): p. 2493-8.

58. Grupp, S.A., Maude, S.L., Shaw, P.A., et al. *Durable Remissions in Children with Relapsed/Refractory ALL Treated with T Cells Engineered with a CD19-Targeted Chimeric Antigen Receptor (CTL019).* in *American Society of Hematology (ASH).* 2015. Orlando, FL.

59. Sotillo, E., et al., *Convergence of Acquired Mutations and Alternative Splicing of CD19 Enables Resistance to CART-19 Immunotherapy.* Cancer Discov, 2015. **5**(12): p. 1282-95.

60. Raetz, E.A., et al., *Chemoimmunotherapy reinduction with epratuzumab in children with acute lymphoblastic leukemia in marrow relapse: a Children's Oncology Group Pilot Study.* J Clin Oncol, 2008. **26**(22): p. 3756-62.

61. Raetz, E.A., et al., *Re-induction chemoimmunotherapy with epratuzumab in relapsed acute lymphoblastic leukemia (ALL): Phase II results from Children's Oncology Group (COG) study*

ADVL04P2. Pediatr Blood Cancer, 2015. **62**(7): p. 1171-5.

62. Chevallier, P., et al., *Vincristine, dexamethasone and epratuzumab for older relapsed/refractory CD22+ B-acute lymphoblastic leukemia patients: a phase II study*. Haematologica, 2015. **100**(4): p. e128-31.

63. Kantarjian, H.M., et al., *Inotuzumab Ozogamicin versus Standard Therapy for Acute Lymphoblastic Leukemia*. N Engl J Med, 2016. **375**(8): p. 740-53.

64. Kreitman, R.J., et al., *Phase I trial of anti-CD22 recombinant immunotoxin moxetumomab pasudotox (CAT-8015 or HA22) in patients with hairy cell leukemia*. J Clin Oncol, 2012. **30**(15): p. 1822-8.

65. Wayne, A.S., et al., *Anti-CD22 immunotoxin RFB4(dsFv)-PE38 (BL22) for CD22-positive hematologic malignancies of childhood: preclinical studies and phase I clinical trial*. Clin Cancer Res, 2010. **16**(6): p. 1894-903.

66. Chevallier, P., et al., *(90)Y-labelled anti-CD22 epratuzumab tetraxetan in adults with refractory or relapsed CD22-positive B-cell acute lymphoblastic leukaemia: a phase 1 dose-escalation study*. Lancet Haematol, 2015. **2**(3): p. e108-17.

67. Xiao, X., et al., *Identification and characterization of fully human anti-CD22 monoclonal antibodies*. MAbs, 2009. **1**(3): p. 297-303.

68. Neelapu, S.S., Frederick L. Locke, Nancy L. Bartlett, Lazaros Lekakis, David Miklos, Caron A. Jacobson, Ira Braunschweig, Olalekan Oluwole, Tanya Siddiqi, Yi Lin, John Timmerman, Patrick J. Stiff, Jonathan Friedberg, Ian Flinn, Andre Goy, Mitchell Smith, Abhinav Deol, Umar Farooq, Peter McSweeney, Javier Munoz, Irit Avivi, Januario E. Castro, Jason R. Westin, Julio C. Chavez, Armin Ghobadi, Krishna V. Komanduri, Ronald Levy, Eric D. Jacobsen, Patrick Reagan, Adrian Bot, John M. Rossi, Lynn Navale, Yizhou Jiang, Jeff S. Aycock, Meg Elias, Jeff Wiezorek and William Y. Go, *Kte-C19 (anti-CD19 CAR T Cells) Induces Complete Remissions in Patients with Refractory Diffuse Large B-Cell Lymphoma (DLBCL): Results from the Pivotal Phase 2 Zuma-1*, in *Blood*. 2016, American Society of Hematology: San Diego, CA.

69. tephen J. Schuster, J.S., Sunita Dwivedy Nasta, Elise A. Chong, Nicole Winchell, Daniel J. Landsburg, David L. Porter, Anthony R. Mato, Honore T Strauser, April M. Schrank-Hacker, Mariusz A Wasik, Simon Francis Lacey, Jan Joseph Melenhorst, Anne Chew, Jens Hasskarl, Katherine T. Marcucci, Bruce L Levine and Carl H June. *Treatment with Chimeric Antigen Receptor Modified T Cells Directed Against CD19 (CTL019) Results in Durable Remissions in Patients with Relapsed or Refractory Diffuse Large B Cell Lymphomas of Germinal Center and Non-Germinal Center Origin, "Double Hit" Diffuse Large B Cell Lymphomas, and Transformed Follicular to Diffuse Large B Cell Lymphomas*. in *ASH*. 2016. San Diego CA.

70. Brudno, J.N., et al., *Allogeneic T Cells That Express an Anti-CD19 Chimeric Antigen Receptor Induce Remissions of B-Cell Malignancies That Progress After Allogeneic Hematopoietic Stem-Cell Transplantation Without Causing Graft-Versus-Host Disease*. J Clin Oncol, 2016. **34**(10): p. 1112-21.

71. Wang, X., et al., *Phase 1 studies of central memory-derived CD19 CAR T-cell therapy following autologous HSCT in patients with B-cell NHL*. Blood, 2016. **127**(24): p. 2980-90.

72. Turtle, C.J., et al., *Immunotherapy of non-Hodgkin's lymphoma with a defined ratio of CD8+ and CD4+ CD19-specific chimeric antigen receptor-modified T cells.* Sci Transl Med, 2016. **8**(355): p. 355ra116.

73. Abramson, J.S., Palomba, L., Gordon, L.I., Lunning, M., Arnason, J., Forero-Torres, A., et al. *IMMUNOTHERAPY WITH THE CD19-DIRECTED CAR T-CELL PRODUCT JCAR017 RESULTS IN HIGH COMPLETE RESPONSE RATES IN RELAPSED OR REFRACTORY B-CELL NON-HODGKIN LYMPHOMA.* in *American Society of Hematology*. 2016. San Diego, CA: American Society of Hematology.

74. Tran, K.Q., et al., *Minimally cultured tumor-infiltrating lymphocytes display optimal characteristics for adoptive cell therapy.* J Immunother, 2008. **31**(8): p. 742-51.

75. Itzhaki, O., et al., *Establishment and large-scale expansion of minimally cultured "young" tumor infiltrating lymphocytes for adoptive transfer therapy.* J Immunother, 2011. **34**(2): p. 212-20.

76. Dudley, M.E., et al., *CD8+ enriched "young" tumor infiltrating lymphocytes can mediate regression of metastatic melanoma.* Clin Cancer Res, 2010. **16**(24): p. 6122-31.

77. Besser, M.J., et al., *Clinical responses in a phase II study using adoptive transfer of short-term cultured tumor infiltration lymphocytes in metastatic melanoma patients.* Clin Cancer Res, 2010. **16**(9): p. 2646-55.

78. Ghassemi, S., et al., *203. Shortened T Cell Culture with IL-7 and IL-15 Provides the Most Potent Chimeric Antigen Receptor (CAR)-Modified T Cells for Adoptive Immunotherapy.* Molecular Therapy. **24**: p. S79.

79. Maude, S.L., et al., *CD19-targeted chimeric antigen receptor T-cell therapy for acute lymphoblastic leukemia.* Blood, 2015. **125**(26): p. 4017-23.

80. Jahr, S., et al., *DNA fragments in the blood plasma of cancer patients: quantitations and evidence for their origin from apoptotic and necrotic cells.* Cancer Res, 2001. **61**(4): p. 1659-65.

81. Roschewski, M., et al., *Circulating tumour DNA and CT monitoring in patients with untreated diffuse large B-cell lymphoma: a correlative biomarker study.* Lancet Oncol, 2015. **16**(5): p. 541-9.

82. Arnold, A., et al., *Immunoglobulin-gene rearrangements as unique clonal markers in human lymphoid neoplasms.* N Engl J Med, 1983. **309**(26): p. 1593-9.

83. Logan, A.C., et al., *Immunoglobulin and T cell receptor gene high-throughput sequencing quantifies minimal residual disease in acute lymphoblastic leukemia and predicts post-transplantation relapse and survival.* Biol Blood Marrow Transplant, 2014. **20**(9): p. 1307-13.

84. Logan, A.C., et al., *Minimal residual disease quantification using consensus primers and high-throughput IGH sequencing predicts post-transplant relapse in chronic lymphocytic leukemia.* Leukemia, 2013. **27**(8): p. 1659-65.

85. Lee, D.W., et al., *ASBMT Consensus Grading for Cytokine Release Syndrome and Neurological Toxicity Associated with Immune Effector Cells.* Biol Blood Marrow Transplant, 2018.

86. Neelapu SS, Tummala S, Kebriaei P, et al, *Chimeric antigen receptor T-cell therapy – assessment and management of toxicities*. Nat Rev Clin Oncol, 2018. **15**: p.47-62.
