# Supplementary material for: CAR T cells with dual targeting of CD19 and CD22 in adult patients with recurrent or refractory B cell malignancies: a phase 1 trial
Source: Nat Med. 2021 Jul 26;27(8):1419–31. doi: 10.1038/s41591-021-01436-0 (PMC8363505; doi:10.1038/s41591-021-01436-0)
Supplement: Supplementary file 2 — Reporting Summary [file 41591_2021_1436_MOESM2_ESM.pdf]

nature portfolio

Corresponding author(s): Dr. Crystal Mackall

Last updated by author(s): Jun 4, 2021

Reporting Summary

Nature Portfolio wishes to improve the reproducibility of the work that we publish. This form provides structure for consistency and transparency in reporting. For further information on Nature Portfolio policies, see our Editorial Policies and the Editorial Policy Checklist.

Statistics

For all statistical analyses, confirm that the following items are present in the figure legend, table legend, main text, or Methods section.

n/a	Confirmed	
☐	☒	The exact sample size (*n*) for each experimental group/condition, given as a discrete number and unit of measurement
☐	☒	A statement on whether measurements were taken from distinct samples or whether the same sample was measured repeatedly
☐	☒	The statistical test(s) used AND whether they are one- or two-sided<br>*Only common tests should be described solely by name; describe more complex techniques in the Methods section.*
☒	☐	A description of all covariates tested
☐	☒	A description of any assumptions or corrections, such as tests of normality and adjustment for multiple comparisons
☐	☒	A full description of the statistical parameters including central tendency (e.g. means) or other basic estimates (e.g. regression coefficient) AND variation (e.g. standard deviation) or associated estimates of uncertainty (e.g. confidence intervals)
☐	☒	For null hypothesis testing, the test statistic (e.g. *F*, *t*, *r*) with confidence intervals, effect sizes, degrees of freedom and *P* value noted<br>*Give P values as exact values whenever suitable.*
☒	☐	For Bayesian analysis, information on the choice of priors and Markov chain Monte Carlo settings
☒	☐	For hierarchical and complex designs, identification of the appropriate level for tests and full reporting of outcomes
☒	☐	Estimates of effect sizes (e.g. Cohen's *d*, Pearson's *r*), indicating how they were calculated

Our web collection on statistics for biologists contains articles on many of the points above.

Software and code

Policy information about availability of computer code

Data collection	BC FACs Diva and BD LSRII for flow cytometry
Data analysis	Statistics: R version 3.6.2 or GraphPad Prism 8, Flow cytometry FloJo v 8 and CytoBank. Isopeak 2.8.0.0 was used for single cell cytokine analysis.R code was written to perform basic analyses and do not entail a custom code or algorithm.

For manuscripts utilizing custom algorithms or software that are central to the research but not yet described in published literature, software must be made available to editors and reviewers. We strongly encourage code deposition in a community repository (e.g. GitHub). See the Nature Portfolio guidelines for submitting code & software for further information.

Data

Policy information about availability of data

All manuscripts must include a data availability statement. This statement should provide the following information, where applicable:

- Accession codes, unique identifiers, or web links for publicly available datasets
- A description of any restrictions on data availability
- For clinical datasets or third party data, please ensure that the statement adheres to our policy

All requests for raw and analyzed data will be reviewed by the corresponding author to determine if the request if subject to any intellectual property of confidentiality considerations. Patient-related data not included in the paper were generated as part of clinical trials and may be subject to patient confidentiality. Any data and materials that can be shared will be released via a Material Transfer Agreement. Source data for Figures 1-4 and Extended Figures 1-7 are provided with the manuscript. CD19-22 Bispecific CAR sequence is in the patent application: U.S. Provisional Patent Application No. 62/135,442 filed March 19, 2015, and which is entitled "DUAL SPECIFIC ANTI-CD22-ANTI-CD19CHIMERIC ANTIGEN RECEPTORS" and the amino acid sequence is provided in Supplementary Figure 2.

March 2021

Field-specific reporting

Please select the one below that is the best fit for your research. If you are not sure, read the appropriate sections before making your selection.

☒ Life sciences ☐ Behavioural & social sciences ☐ Ecological, evolutionary & environmental sciences

For a reference copy of the document with all sections, see nature.com/documents/nr-reporting-summary-flat.pdf

Life sciences study design

All studies must disclose on these points even when the disclosure is negative.

Sample size	All infused with CD19-22.BB.z patients were included in analysis of clinical outcomes. Axi-cel patients with available IHC and/or quantitative flow cytometry were included for analysis. No formal sample size calculation was made.
Data exclusions	No data were excluded
Replication	All experiments reported in the paper were from patient samples. Data from Extended Figure 7A was performed with samples from a healthy donor and run successfully in technical triplicate.
Randomization	This was a Phase I to test safety and tolerability and therefore randomization was not performed. Standard of care axi-cel patients were not randomized.
Blinding	No blinding was performed in this Phase I trial.

Reporting for specific materials, systems and methods

We require information from authors about some types of materials, experimental systems and methods used in many studies. Here, indicate whether each material, system or method listed is relevant to your study. If you are not sure if a list item applies to your research, read the appropriate section before selecting a response.

Materials & experimental systems

n/a	Involved in the study	
☐	☒	Antibodies
☐	☒	Eukaryotic cell lines
☒	☐	Palaeontology and archaeology
☒	☐	Animals and other organisms
☐	☒	Human research participants
☐	☒	Clinical data
☒	☐	Dual use research of concern

Methods

n/a	Involved in the study	
☒	☐	ChIP-seq
☐	☒	Flow cytometry
☒	☐	MRI-based neuroimaging

Antibodies

Antibodies used	All antibodies used, manufacturer, part number, respective fluorochromes and dilutions are listed in the methods section in: Panel 1, Antibody Index for Phenotyping and Exhaustion Profiling, Panel 2, and Panel 3.
Validation	All commercially-available antibodies are validated and routinely tested as described in the manufacturers product information (BD Biosciences, Biolegend, Miltenyi and Thermo Fisher). The one non-commerically available antibody is CD19 anti-Id-DL650/APC which was validated for specificity in Jena et. al, PLOSOne 2013., We have conjugated, validated, and titrated in our lab to determine the appropriate volume to use for staining. We also conduct stability testing on this reagent to ensure the quality. We compared antibody-specific staining to isotype, FMO, or no staining control samples and we use the same lot numbers of antibody when possible in our panels. Primary antibodies have been titrated to determine the optimal stain concentration for each test. This data is included in Supplemental Figure 3.

Eukaryotic cell lines

Policy information about cell lines

Cell line source(s)	NALM6 from NCI (Terry Fry) - lines were CRISPR/gene edited to create NALM6 CD22KO, NALM6 CD19KO, and NALM6 DO KO lines
Authentication	STR DNA profiling of all cell lines is conducted by Genetica Cell Line testing once per year
Mycoplasma contamination	All stock NALM6 vials were tested for mycoplasma and found to be negative.

Commonly misidentified lines (See ICLAC register)	No commonly misidentified cell lines were used according to the ICLAC register.

Human research participants

Policy information about studies involving human research participants

Population characteristics	Two disease cohorts were treated in this Phase I trial. Patients with aggressive large B-cell lymphoma relapsed or refractory after 2 lines of therapy including DLBCL, PMBCL, and large cell lymphoma transformed from follicular or other indolent lymphomas were eligible. Patients with B-acute lymphoblastic leukemia relapsed or refractory to 2 lines of therapy were eligible. Patient characteristics are described in Results, Table 1 A and B and Methods. Standard of care axi-cel patients consisted of patients with aggressive large cell lymphoma relapsed or refractory after 2 lines of therapy.
Recruitment	Patients were not actively recruited and were enrolled on a first-come first-serve basis. The trial patients reflect our overall patient composition both in ethnicity and age range at our academic medical center.
Ethics oversight	The Phase I study as well as the Clinical Bio-repository was governed by the Institutional Review Board of the Stanford Cancer Center

Note that full information on the approval of the study protocol must also be provided in the manuscript.

Clinical data

Policy information about clinical studies

All manuscripts should comply with the ICMJE guidelines for publication of clinical research and a completed CONSORT checklist must be included with all submissions.

Clinical trial registration	Clinicaltrials.gov NCT03233854
Study protocol	The full protocol is included in the Supplementary Materials
Data collection	All data was collected between September 11,2017 and June 15, 2020 at Stanford Cancer Center
Outcomes	In the Methods we included the following: Primary outcomes were feasibility of manufacture of CD19-22.BB.Z-CAR as well as safety of CD19-22.BB.Z-CAR. Secondary outcomes included efficacy of CD19-22.BB.Z-CAR to induce clinical response at 3 months for LBCL and 1 month for B-ALL. Exploratory objectives included the rate of CD19 negative relapse after CD19-22.BB.Z-CAR.

Flow Cytometry

Plots

Confirm that:

☒ The axis labels state the marker and fluorochrome used (e.g. CD4-FITC).

☒ The axis scales are clearly visible. Include numbers along axes only for bottom left plot of group (a 'group' is an analysis of identical markers).

☒ All plots are contour plots with outliers or pseudocolor plots.

☒ A numerical value for number of cells or percentage (with statistics) is provided.

Methodology

Sample preparation	Up to 1 million CART cells or tumor cells from the Prodigy, cell culture, or patient samples were washed in FACS Buffer (lX PBS+ 2% FBS), labeled in 100 ul of FACS buffer containing the relevant antibody cocktail, and incubated at 4C in the dark for 30 minutes. Samples were washed in 2-4 ml of FACS buffer prior to running on instrument. For ICS data, samples included both an extracellular stain and wash, followed by an intracellular stain and wash, as indicated in the methods.
Instrument	BD LSRFortessa X-20 or BDFACS Lyric
Software	FACSDiva for collection and FlowJo for Analysis
Cell population abundance	Cell sorting was not performed
Gating strategy	Debris was gated out and samples were gated on FSC/SSC lymphocyte gate, on singlets using FSC-A/FSC-H, viable cells or CD3 cells as applicable for the figure. Beads were used for compensation and FMO controls were used in multi-color panels to assist with gating strategies. In some instances, healthy donor CART were run in parallel as a control and to check batch normalization.

☒ Tick this box to confirm that a figure exemplifying the gating strategy is provided in the Supplementary Information.
